# Regioselective [2 + 2 + 2] Alkyne Cyclotrimerizations to Hexasubstituted Benzenes: Syntheses of Fomajorin D and Fomajorin S

**DOI:** 10.1021/acs.joc.4c00224

**Published:** 2024-04-29

**Authors:** Amir Tavakoli, Gregory B. Dudley

**Affiliations:** C. Eugene Bennett Department of Chemistry, West Virginia University, Morgantown, West Virginia 26505, United States

## Abstract

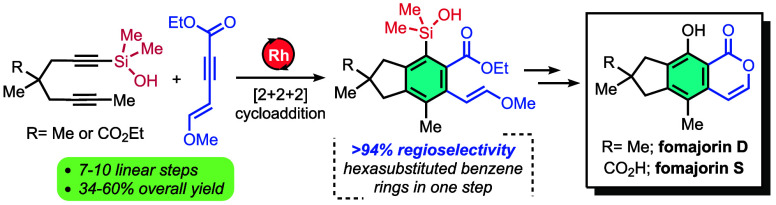

Hexasubstituted benzenoids are prepared by regioselective bimolecular [2 + 2 + 2] alkyne cyclotrimerizations of diynes with alkynes. These convergent and efficient benzannulations are directed toward and lead to the first total syntheses of the illudalane sequiterpenes fomajorin D and S, in 10 and 7 steps, respectively, from commercially available dimedone. Control experiments suggest that hydrogen bonding may play a role in preorganizing the diyne and alkyne coupling partners for establishing the desired regioselectivity, but other factors are likely involved in the selective formation of other regioisomers.

## Introduction

**Figure 1:**
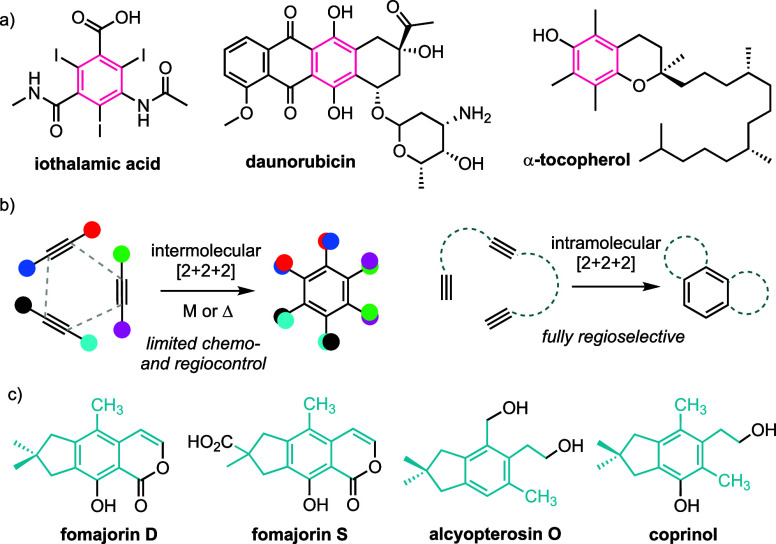
(a) Hexasubstituted benzenes for modern medicine; (b) chemo- and regioselectivity challenges in Reppe-type alkyne cyclotrimerizations; (c) representative illudalane sesquiterpenes.

Benzene rings are an order of magnitude more common than any other ring system in small molecule drugs,^1^ but highly substituted benzenoids are underrepresented in drug discovery, prompting a call to action.^2^ In principle, dense functionalization around a rigid, robust, and privileged scaffold creates myriad opportunities in molecular design. In practice, however, ≥75% of benzene-derived active pharmaceutical ingredients (APIs) feature simple mono-, *ortho*-, and/or *para*-substituted benzenes, reflecting their comparative ease of synthesis by substitution chemistry. In contrast, hexasubstituted benzenoids comprise only 2% of FDA-approved APIs, and only 0.5% of APIs are hexasubstituted benzenoids *not* structurally related to one of three natural products: iothalamic acid, α-tocopherol, and daunorubicin (Figure 1a). Methodology for making highly substituted benzene rings is needed to advance synthetic chemistry into new structure space, with hexasubstituted benzenoids posing a uniquely important and unmet challenge.

Convergent Reppe^3^-type [2 + 2 + 2] alkyne cyclotrimerizations are conceptually ideal in this regard, but regiocontrol is a major challenge with no general solution.^4^ Most synthetic applications of [2 + 2 + 2] cyclotrimerizations are unimolecular (e.g., tethered triynes) and/or involve symmetrical substrates that avoid any question of regiochemistry (Figure 1b).^4c^ We recently identified regioselective bimolecular [2 + 2 + 2] cyclotrimerizations of 3-pentynol with neopentylene-tethered (NPT) 1,6-diynes^5^ to produce pentasubstituted benzene rings of interest for the synthesis of certain alcyopterosins and coprinol.^6^ These observations encouraged us to target hexasubstituted benzene rings, specifically with a focus on the natural product fomajorin D (and fomajorin S, Figure 1c). Our working hypothesis for the observed regioselectivity is that noncovalent interactions (e.g., hydrogen bonding) between alkyne partners guide their orientation around the catalyst, although direct interactions with cationic rhodium (Rh) have been invoked previously.^7,8^

Fomajorin D and fomajorin S are illudalane sesquiterpenes^9^ from the basidiomycete fungus *Heterobasidion annosum*, also known as *Fomes annosus*.^10^ This destructive forest pathogen causes annosus root rot, a conifer disease having a negative economic impact on the order of $800 M annually in Europe, with parallel impacts to the United States forestry industry and across the northern hemisphere.^11^ Methanolic extracts of *H. annosum* are reported to induce apoptosis in cancer cells,^12^ although nothing has been reported on the biological activities of the fomajorins specifically.

**Scheme 1:**
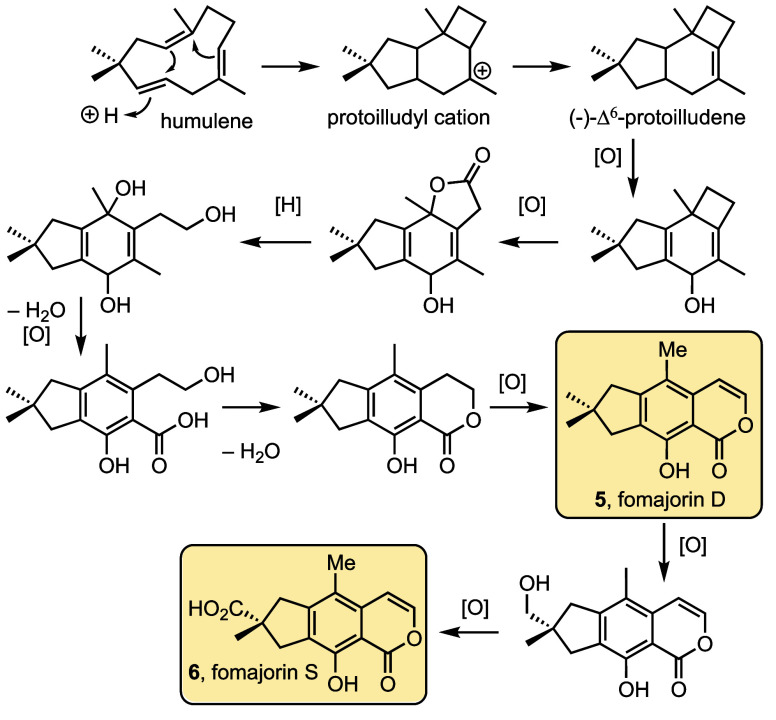
Biosynthetic Pathways Postulated for Fomajorin D and S

Scheme 1 outlines postulated biosynthetic pathways leading to fomajorins D and S,^10^ although this complex biosynthesis is difficult to replicate in vitro.^13^ In addition to the hexasubstituted benzenoid core, these isocoumarin natural products are of note for the lack of substituents on the ene-lactone ring.^10^ Our ongoing interest in the synthesis of bioactive illudalanes^14^ made fomajorin D an appealing focal point of strategic efforts to gain improved access to highly substituted benzenoids for drug discovery.

**Scheme 2:**
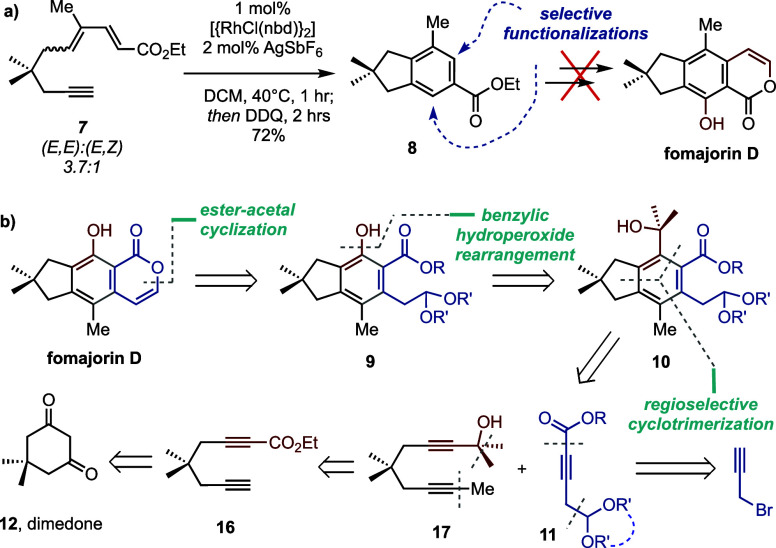
(a) Initial and (b) Revised Retrosynthetic Analyses of Fomajorin D

Our initial approach to fomajorin D focused on selective functionalization of Indane **8**,^15^ which is available by cycloisomerization/oxidation of 1,6-enyne **7** (Scheme 2a).^16^ This route was ultimately abandoned because of the compounding strategic and tactical limitations of substitution reactions for making penta- and hexasubstituted benzenes. Meanwhile, developments in regioselective cyclotrimerizations cited above support a hypothetical assembly of hexasubstituted benzene **10** in one step from two acyclic precursors of similar complexity (**11** and **17**, Scheme 2b).

Our revised retrosynthetic analysis thus results with identifying ester acetal **9** as a direct precursor to the ene-lactone fomajorin D. Phenol **9** was envisioned to arise by benzylic hydroperoxide oxidation^17^ of aryldimethyl carbinol **10**, which is the aspirational product of a regioselective [2 + 2 + 2] cyclotrimerization of diyne dimethylcarbinol **17** with an alkynoate acetal **11**. Based on prior observations, we hypothesized that hydrogen bonding between the alcohol functionality on diyne **17** and the ester of alkyne **11** would bias regioselectivity in the desired manner. As discussed herein, we ultimately arrived at alternative cyclotrimerizations offering complementary regioselectivities, one of which leading to the first chemical synthesis of fomajorin D. The same strategy was then adapted for the synthesis of fomajorin S.

## Results and Discussion

**Scheme 3:**
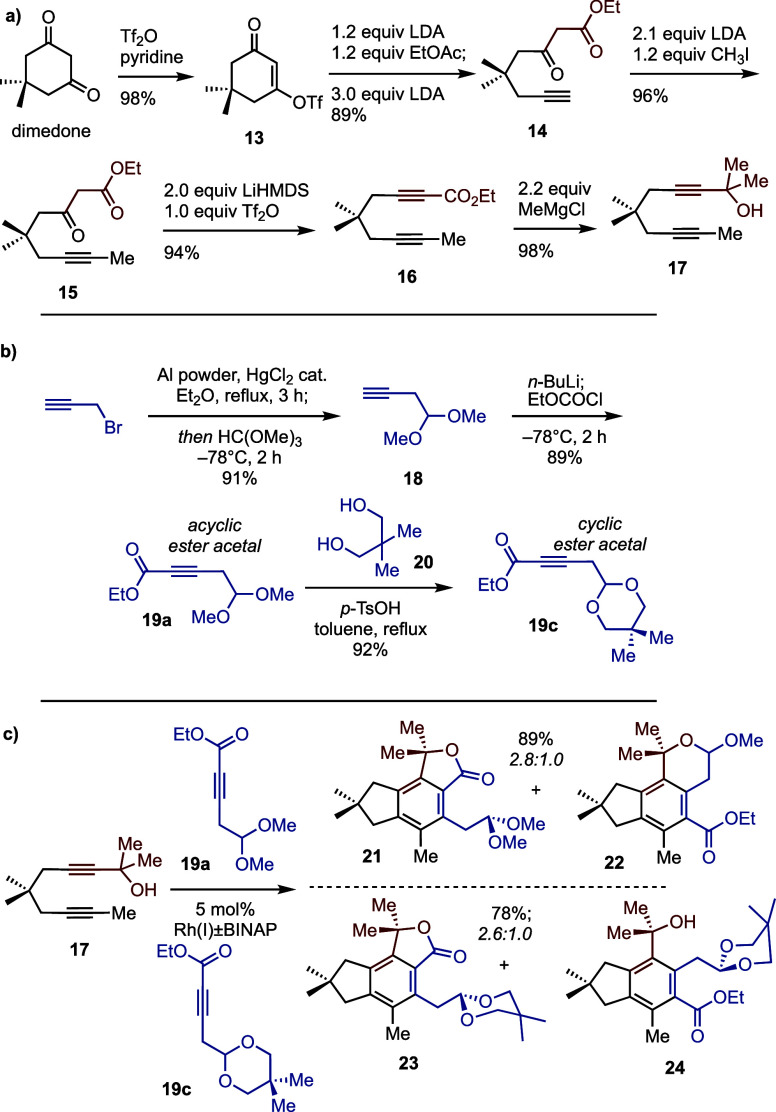
(a) Preparation of Diyne Dimethyl Carbinol **17**; (b) Preparation of Acyclic (**19a**) and Cyclic (**19c**) Alkynyl Acetal Partners; (c) Initial Attempts at Regioselective Cyclotrimerization Using Diyne Dimethylcarbinol **17**

Our synthesis began with the preparation of diyne **17** via the addition/fragmentation methodology previously established in our lab.^5,18^ Our general approach involves triflation of dimedone to vinylogous acyl triflate (VAT) **13** (Scheme 3a). The ring-opening addition/fragmentation of VAT **13** then generates β-keto ester **14**, whose terminal alkyne was selectively methylated to give β-keto ester **15** in excellent yield. With the first internal alkyne in place, dehydration of β-keto ester **15** was achieved by two equivalents of LiHMDS and one equivalent of Tf_2_O.^19,20^ Finally, methylation using MeMgCl at 0 °C furnished diyne dimethylcarbinol **17**.

Scheme 3b depicts the preparation of internal monoalkyne partners **19a** and **19c** (cf. **11**, Scheme 2b) starting from propargyl bromide. Alkynyl acetal **18** was first generated by adding trimethyl orthoformate to a solution of an in situ-generated propargylaluminum reagent. The terminal alkyne was then metalated for reaction with ethyl chloroformate to give alkynyl ester acetal **19a**. We also prepared cyclic acetal **19c** by acid-catalyzed condensation with neopentylene glycol (**20**).

With our first set of coupling partners (**19a** and **19c**) in hand, we turned to the Rh-catalyzed cyclotrimerization reactions. The active Rh(BINAP)BF_4_ catalyst was prepared by hydrogenation of commercially available Rh(cod)_2_BF_4_ in the presence of (±)-BINAP, and coupling reactions of diyne carbinol **17** with alkynes **19a** and **19c** were then examined. These reactions produced mixtures that modestly favored the desired regiochemistry (<3:1, Scheme 3c) but with additional cyclizations that are complicating but not necessarily surprising for this densely functionalized system. The lactones with the desired connectivity (**21** and **23**) proved resistant to hydroperoxide oxidations aimed at installing the desired phenol (cf. **9**, Scheme 2b) using various combinations of protic and/or Lewis acids (e.g., HCl or BF_3_·OEt_2_) and peroxide sources (e.g., H_2_O_2_, oxone). The various attempts yielded only unreacted starting materials, oxidation of the acetal to the corresponding carboxylic acid, and/or general decomposition.

**Scheme 4:**
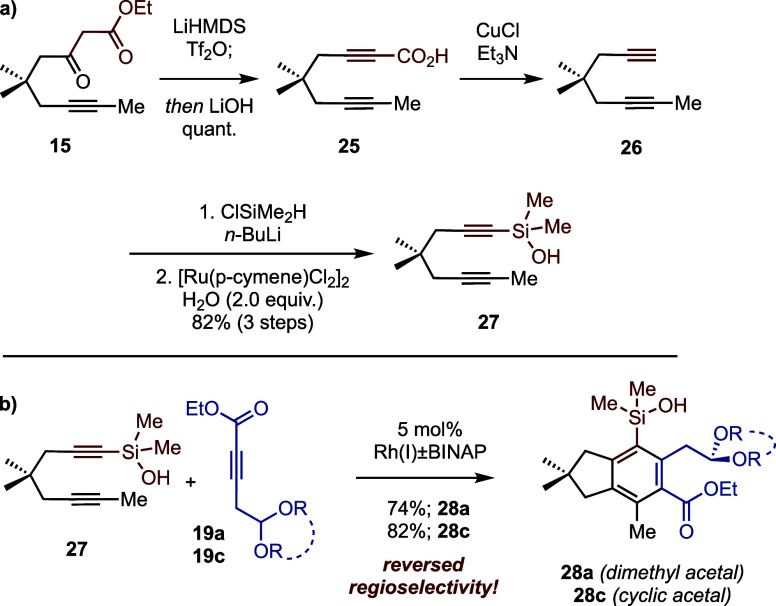
(a) Preparation of Diyne Dimethylsilanol; (b) Cycloterimerization of Diyne Dimethylsilanol and Alkynyl Acetal Partners

The modest regioselectivities and complications associated with the lactone led us to consider the analogous silanol (**27**, Scheme 4a). We reasoned that the silicon (Si) atom would ameliorate lactonization while still enabling intramolecular noncovalent interactions that hypothetically control regioselective cyclotrimerization. We thus prepared diyne dimethylsilanol **27** in 82% overall yield from β-keto ester **15** by dehydration and in situ saponification to diyne acid **25**, followed by Cu-catalyzed decarboxylation (to **26**), base-mediated dimethylsilylation, and Ru-catalyzed silane oxidation to furnish diyne **27**. Remarkably, and in contrast to diyne carbinol **17**, cyclotrimerizations of diyne silanol **27** with alkynes **19a** and **19c** gave single hexasubstituted benzene products in good yields but favoring the undesired regioisomers (**28a** and **28c**).^21^ Although not productive for the synthesis of fomajorin D, these results may offer clues into the nature of substrate-controlled cyclotrimerizations.^22^

**Scheme 5:**
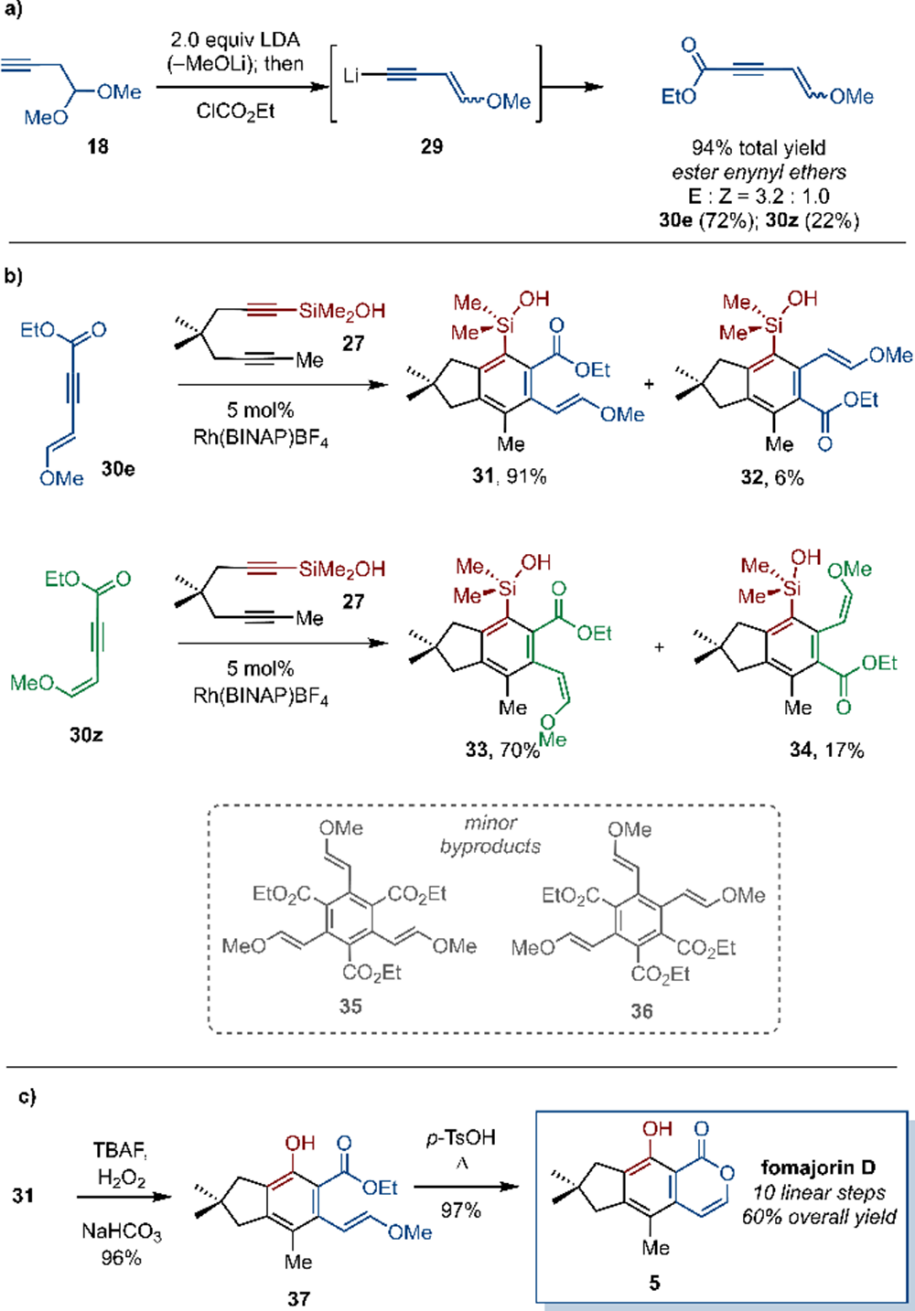
(a) Preparation of Enynyl Ethers **30e** and **30z**; (b) Cyclotrimerization of Diyne Dimethylsilanol and E-Ester Enynyl Ether; (c) Final Two Steps for Fomajorin D Synthesis

The observed differences in regioselectivity between carbinol **17** and silanol **27** with alkynes **19** are presumably functions of the differences between carbon and silicon atoms. Considering the longer bonds, greater electrophilicity, and enhanced acidity of silanols along with the observed change in regioselectivity, we considered that silanol **27** pre-engage preferentially with the oxygens of the acetal group as opposed to the ester of **19**. If so, then eliminating the acetal might reorient coupling in favor of the desired regioisomer. Indeed, this proved to be the case (cf. Scheme 5).

To test this new hypothesis aimed at biasing the monoalkyne coupling partner for the desired regioselectivity, we designed new internal alkynes in which methanol is formally removed^23^ from acetal **19a** in favor of stereoisomeric vinyl ethers (Scheme 5a). We considered that the resulting enynes would provide enhanced partial negative charge on the ester carbonyl for greater basicity, with the remaining methoxy group being correspondingly less effective as an intermolecular hydrogen bond acceptor (especially in the case of *E*-isomer **30e**). The push–pull nature of substituents on the alkyne may also lengthen and weaken the alkyne π-bond, perhaps rendering it more reactive to (cyclo)addition reactions.

The preparation of enynes **30e** and **30z** was achieved by treating alkynyl acetal **18** with two equivalents of LDA and then trapping the presumed acetylide intermediate (**29**) with ethyl chloroformate. The desired alkynes **30e** and **30z** were obtained in a 3.2:1 ratio (94% combined yield, Scheme 5a), separated by chromatography, and independently subjected to cyclotrimerization with silanol **27** (Scheme 5b). Gratifyingly, the desired regioselectivity was observed in both cases. *E*-isomer **30e** gave better regioselectivity for hexasubstituted benzene **31**—94:6 over **32** as estimated by ^1^H NMR spectroscopy—and a 97% combined yield (91% estimated yield of **31**). Byproducts **35** and **36** (ca. 1:1) from self-cyclotrimerization of enyne **30e** were also identified in the crude reaction mixture; these are easily purged chromatographically, and 2.2 equiv of **30e** allows for complete consumption of **27**. The parallel reaction of *Z*-isomer **30z** with **27** was less regioselective, providing an 80:20 mixture of regioisomers **33** and **34** in 87% combined yield.

Arylsilanol **31** was laboriously separated from isomer **32** by column chromatography and carried forward through two high-yielding steps to fomajorin D (Scheme 5c). Alternatively, mixtures of **31** and **32** can be carried forward to the same effect. Tamao-Fleming^24^ oxidation with TBAF and H_2_O_2_ gave rise to phenol **37** in 96% yield from **31**, and cyclization and elimination under acidic conditions then crafted the isocoumarin core and delivered fomajorin D in 97% yield. To our knowledge, this is the first synthesis of fomajorin D, accomplished here in 10 linear steps and 60% overall yield from dimedone.

**Scheme 6:**
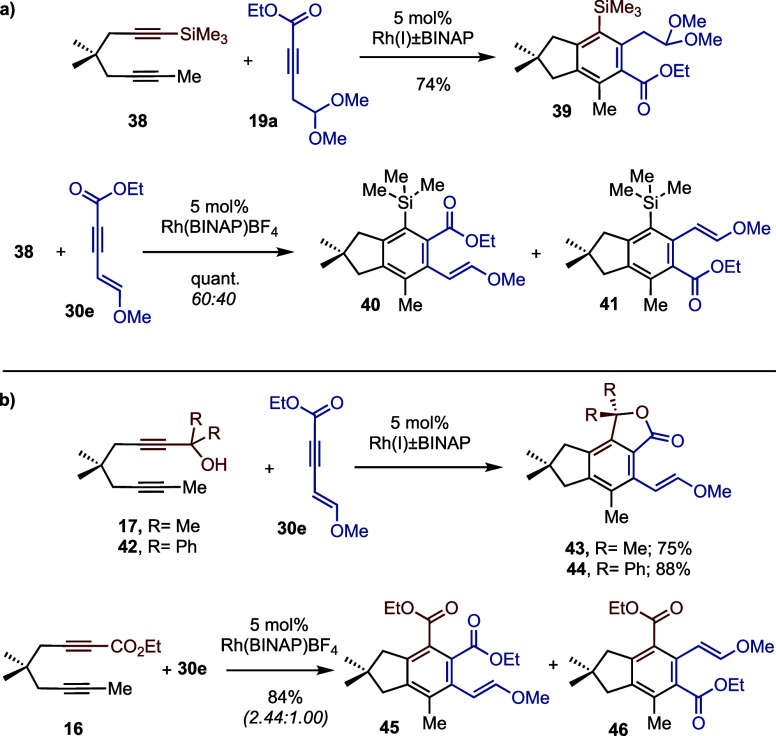
(a, b) Control Cyclotrimerization Experiments Using Diyne Trimethylsilane **33** and Other Diynes

Control experiments provide preliminary insights into the observed cyclotrimerizations and raise additional questions for further study. TMS-diyne **38** reacted regioselectively with alkyne **19a** (Scheme 6a), suggesting that hydrogen bonding was not critical for the undesired (in this context) regioselectivity^22^ (cf. Scheme 4). On the other hand, TMS-diyne **38** failed to react regioselectivity with enyne **30e**, which suggests that the hydroxyl functionality plays an important role in setting the desired regiochemistry (cf. **27** + **30e** → **31**, Scheme 5b). The importance of the hydroxyl functionality in setting the desired regiochemistry is reinforced by regioselective cyclotrimerizations of **30e** with carbinols **17** and **42**, but not with ester **16** (Scheme 6b).^25^

Diyne dimethylsilanol **27** and monoalkyne **30e** thus stand as our preferred combination for the synthesis of fomajorin D, and these other examples of divergent regiocontrol provide important leads for future methodology.

**Scheme 7:**
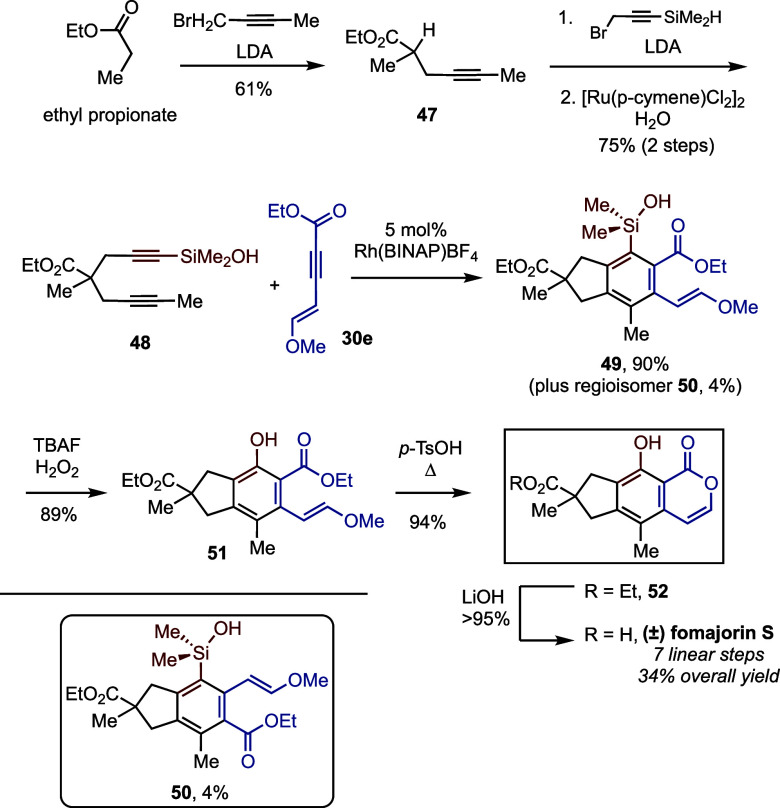
Synthesis of (±)-Fomajorin S

Finally, we adapted our strategy to the synthesis of (±)-fomajorin S (Scheme 7). Diyne dimethylsilanol **48** was prepared by sequential *bis*-propargylation of ethyl propionate followed by silane oxidation as before. Cyclotrimerization of **48** with alkyne **30e** gave the desired hexasubstituted arylsilanol **49** in 90% yield, along with only 4% of undesired regioisomer **50**. Tamao oxidation of silanol **49** furnished phenol **51** in 89% yield, and the subsequent acidic cyclization gave fomajorin S ethyl ester (**52**) in 94% yield. From there, fomajorin S was obtained in quantitative yield by LiOH-mediated hydrolysis.

## Conclusions

Hexasubstituted benzene derivatives are prepared here by regioselective bimolecular [2 + 2 + 2] alkyne cyclotrimerizations. These convergent and efficient benzannulations were directed toward and led to the first total syntheses of the illudalanes fomajorin D and fomajorin S, in 10 and 7 steps, respectively, from commercially available dimedone. Hexasubstituted benzenoids are underrepresented in drug discovery. The observations and preliminary methodology identified here can open new regions of chemical space for medicinal chemistry, including especially for the opportunities to develop the pharmacological potential of the fomajorins and other illudalanes.

## Data Availability

The data underlying this study are available in the published article and its Supporting Information.

## Supporting Information Available

Experimental procedures, characterization data, and NMR spectra of the synthesized compounds (PDF)
