# Supplementary material for: Regioselective [2 + 2 + 2] Alkyne Cyclotrimerizations to Hexasubstituted Benzenes: Syntheses of Fomajorin D and Fomajorin S
Source: J Org Chem. 2024 Apr 29;89(10):6847–52. doi: 10.1021/acs.joc.4c00224 (PMC11110065; doi:10.1021/acs.joc.4c00224)
Supplement: Supplementary file 1 — jo4c00224_si_001.pdf [file jo4c00224_si_001.pdf]

Supporting Information

Regioselective [2+2+2] Alkyne Cyclotrimerizations to Hexasubstituted Benzenes: Syntheses of Fomajorin D and Fomajorin S

Amir Tavakoli and Gregory B. Dudley*

Email: gregory.dudley@mail.wvu.edu

C. Eugene Bennett Department of Chemistry, West Virginia University, Morgantown, West Virginia 26505, United States.

Contents

General methods……………………………………………………………………………S2

General procedure A for the diyne-alkyne cyclotrimerization reactions………………………..S2

References……………………………………………………………………………....S16

NMR Spectra…………………………………………………………………………... S17

Experimental:

General methods. The following general experimental methods apply to all procedures reported herein unless otherwise stated. All reactions were conducted in oven-dried glassware under an atmosphere of nitrogen using anhydrous solvents. Dimedone and all other chemicals were purchased from commercial sources (e.g., Sigma-Aldrich) and used as received. ***Caution!*** Although no safety problems were experienced in the course of this work, standard safety precautions should be employed for all work with LiHMDS. Syringes were used in all protocols requiring the transfer of a liquid reactant of solvent unless otherwise stated. Tetrahydrofuran (THF), toluene, and dichloromethane (DCM) were dried under a column of molecular sieves in an argon atmosphere. A Schleck line was used in all reactions to purge reaction vessels and provide an inert, nitrogen atmosphere. Column chromatography was performed using a Biotage Isolera One automated flash column system. Yields are reported as isolated yields considered to be ≥95% pure by ^{1}H NMR following flash chromatography. All new compounds were characterized using a JEOL 400 spectrometer to conduct ^{1}H and ^{13}C NMR spectroscopy in $CDCl_3$ (≥99.8 atom % D, contains 0.03% (v/v) TMS) purchased from Cambridge Isotope Laboratories. Chemical shifts (δ) are reported in units (ppm) referenced to 0.0 ppm of TMS in the ^{1}H spectrum and 77.0 ppm of $CDCl_3$ in the ^{13}C spectrum. Coupling constants (J) are reported in Hertz (Hz). Mass spectrometry was performed on a Thermo Scientific Q Exactive Plus Hybrid Quadrupole-Orbitrap and recorded using electrospray ionization (ESI).

General procedure A for the diyne-alkyne cyclotrimerization reactions: To a flask/vial was added stir bar, (±)-BINAP (5 mol%) and $[Rh(cod)_2]BF_4$ (5 mol%) precatalyst. A septum cap was attached, and the flask was evacuated and slowly back-filled with nitrogen before DCM (0.1 M with respect to the diyne) was added. The nitrogen line was removed, and a hydrogen-filled balloon was inserted through the cap into the flask. Another needle was inserted, and the flask was purged with hydrogen for 5 minutes and the needle was removed. The homogeneous light orange solution was stirred at room temperature under the atmosphere of hydrogen gas for 1 hour, at which point the active $Rh(BINAP)BF_4$ catalyst was assumed to have formed (the solution turned dark red). The resulting mixture was concentrated by rotary evaporation, evacuated, backfilled with nitrogen, and DCM (0.4 M) and alkyne partner (1.5-2.2 equiv) were then added respectively. Immediately after, a solution of diyne (1.0 equiv) in DCM (0.2 M) was added slowly over 1 hour at room temperature using a syringe pump. For ester enynylether **30e**, a solution of it was slowly added over 1 hour simultaneously with the diyne because of its increased reactivity. The resulting solution was stirred for an additional 1 hour or until the diyne had been fully consumed based on TLC. The solution was then concentrated by rotary evaporation, and the crude was loaded onto silica gel. Purification by flash column chromatography on silica gel (5–40% EtOAc/Hexanes) furnished the corresponding adducts.

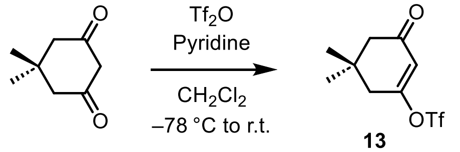

13

5,5-dimethyl-3-oxocyclohex-1-en-1-yl trifluoromethanesulfonate (13). Dimedone (2.80 g, 20.0 mmol, 1.0 equiv) and pyridine (3.2 mL, 40 mmol, 2.0 equiv) were dissolved in DCM (100 mL, 0.2 M), and the resulting clear solution was stirred at –78 °C for 10 minutes, followed by adding Tf_2O (4.0 mL, 24.0 mmol, 1.2 equiv) via syringe over a period of 15 minutes. The reaction mixture was stirred for 20 minutes in a –78 °C bath, then for 20 minutes in a 0 °C bath, and finally for 30 minutes without external cooling. The reaction was quenched using 1.0 M aqueous HCl solution (40 mL), and the resulting mixture was extracted with Et_2O (3×25 mL). The organic layers were combined, washed with saturated aqueous $NaHCO_3$ (50 mL) and brine (50 mL), dried over Na_2SO_4, filtered, and concentrated by rotary evaporation. The resulting crude red oil was purified by silica gel flash chromatography (5-20% EtOAc/Hexanes) to afford 5.32 g of VAT **13** as a colorless oil (98% yield), R_f=0.46 (20% EtOAc/Hexanes). This compound was found to be 97% pure by qNMR using 1,4-dimethoxybenzene as the internal standard. The characterization data matched the previous reports.[1] The product was indefinitely stable when stored in freezer (–23 °C).**^{1}H NMR** (400 MHz, $CDCl_3$): δ 6.07 (s, 1H), 2.56 (d, J = 1.5 Hz, 2H), 2.32 (s, 2H), 1.14 (s, 6H) ppm; **^{13}C{^{1}H}NMR** (100 MHz, $CDCl_3$): δ 197.5, 166.0, 118.3 (q, J = 319.8 Hz), 50.5, 42.3, 33.4, 28.0 ppm; **^{19}F NMR** (376 MHz, $CDCl_3$): δ –73.44 ppm.

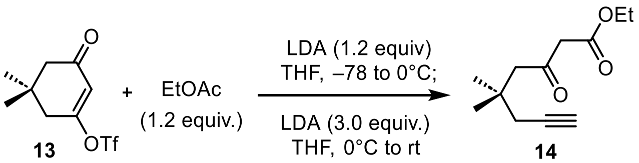

13 **14**

Ethyl 5,5-dimethyl-3-oxo-7-octynoate (14). A 25-mL pear-shaped flask was charged with 7.5 mL THF, diisopropyl amine (0.85 mL, 6.00 mmol, 1.2 equiv), and cooled to –78 °C for 10 minutes. A solution of *n*-BuLi (3.80 mL, 1.6 M in hexanes, 6.00 mmol, 1.2 equiv) was added to the flask over 5 minutes and the mixture was stirred at –78 °C for 30 minutes to make a 0.5 M LDA solution. A solution of EtOAc (0.59 mL, 6.00 mmol, 1.2 equiv) in 4 mL THF was then added dropwise over a 15-minute period to the LDA solution which was then stirred at –78 °C for 30 minutes. The prepared solution of enolate **5** (6.00 mmol, 1.2 equiv) was transferred to a solution of VAT **4** (1.40 g, 5.00 mmol, 1.0 equiv) in THF (20 mL, 0.25 M) at –78 °C via cannula over ca. 5 minutes. The mixture was then stirred at –78 °C for 10 min, and at 0 °C for 10 min. A solution of freshly prepared LDA (15.0 mmol, 3.0 equiv) was added to the flask via canula over 10 minutes and the ice bath was removed. The mixture was allowed to stir at room temperature for 1 hour before quenching with a sat. NH_4Cl solution (40 mL). The layers were separated, and the aqueous layer was repeatedly extracted with diethyl ether (3×20 mL). The organic layers were combined, washed with brine, dried over MgSO4, filtered, and concentrated by rotary evaporation to afford a red oil. The crude product was purified by silica gel flash chromatography (1- 6% EtOAc/Hexanes) to afford 933 mg β-keto ester **14** (89%) as a light-yellow oil, R_f = 0.53 (20% EtOAC/Hexanes). The characterization data matched the previous reports.[2] **^{1}H NMR** (400 MHz, $CDCl_3$): δ 4.20 (q, *J* = 7.2 Hz, 2H), 3.44 (s, 2H), 2.59 (s, 2H), 2.28 (d, *J* = 2.6 Hz, 2H), 2.01 (t, *J* = 2.6 Hz, 1H), 1.29 (t, *J* = 7.2 Hz, 3H), 1.10 (s, 6H) ppm; **^{13}C{^{1}H}NMR** (100 MHz, $CDCl_3$): δ 202.0, 167.2, 91.6, 81.9, 70.6, 61.3, 51.5, 50.9, 45.8, 33.4, 31.1, 26.9, 14.1 ppm; **HRMS** (ESI): [M – H]$^-$ calculated for $C_{12}H_{17}O_3^-$: 209.1183; found: 209.1178.

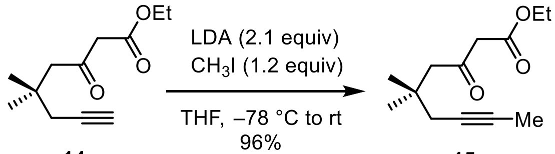

14 **15**

ethyl 5,5-dimethyl-3-oxonon-7-ynoate (15). *n*-BuLi (1.6 M in hexanes, 5.9 mL, 9.52 mmol, 2.1 equiv) was added dropwise to a solution of diisopropylamine (1.3 mL, 9.52 mmol, 2.1 equiv) in THF (18 mL, 0.25 M) at −78 °C. The resulting solution was stirred at this temperature for 30 minutes. A solution of β-keto ester **14** (953 mg, 4.53 mmol, 1.0 equiv) in THF (4.0 mL) was then added dropwise over 15 minutes. The resulting mixture was stirred at −78 °C for 30 minutes followed by dropwise addition of iodomethane (0.34 mL, 5.44 mmol, 1.2 equiv) at −78 °C. The reaction mixture was allowed to warm up to room temperature overnight. The reaction was quenched by the addition of saturated NH_4Cl solution (25 mL). The aqueous layer was further extracted with ether (3×10 mL). The combined organic layers were dried over anhydrous MgSO4, filtered, and concentrated under vacuum to afford a light yellow oil which was found to be pure by ^{1}H NMR analysis and used for the next step without further purification (975 mg, 96%), R_f= 0.55 (20% EtOAC/Hexanes).

^{1}H NMR (400 MHz, $CDCl_3$) δ 12.11 (s, 1H-*enol*), 4.98 (s, 1H-*enol*), 4.19 (q, *J* = 7.1 Hz, 2H-*keto*), 4.18 (q, *J* = 7.1 Hz, 2H-*enol*), 3.43 (s, 2H), 2.54 (s, 2H), 2.16 (q, *J = 2.61* Hz, 2H-*keto*), 2.11 (q, *J = 2.61* Hz, 2H-*enol*), 1.80 (t, *J = 2.54* Hz, 3H-*enol*), 1.79 (t, *J = 2.54* Hz, 3H-*keto*), 1.28 (t, *J = 7.1* Hz, 3H-*enol*), 1.27 (t, *J = 7.1* Hz, 3H-*keto*), 1.05 (s, 6H-*keto*), 1.02 (s, 6H-*enol*) ppm; **^{13}C{^{1}H}NMR** (100 MHz, $CDCl_3$) δ 202.4, 177.2, 172.8, 167.3, 91.6, 78.0, 76.6, 61.4, 60.1, 51.9, 51.2, 46.0, 34.6, 34.0, 32.6, 32.0, 27.3, 27.1, 14.4, 14.2, 3.6. ppm; **HRMS** (ESI): [M – H]$^-$ calculated for $C_{13}H_{19}O_3^-$: 223.1340; found: 223.1335.

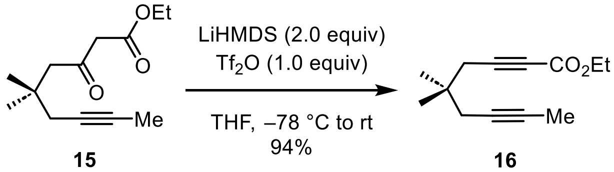

15 **16**

ethyl 5,5-dimethylnona-2,7-diynoate (16). By analogy to the published procedure[2], a round bottom flask was charged with dry THF (52 mL, 0.25 M) and LiHMDS (26 mL, 1.0 M/THF, 26 mmol, 2.0 equiv). The solution was then cooled to −78 ° followed by the addition of a solution of β-keto ester **15** (3.04 g, 13 mmol, 1.0 equiv) over 10 minutes. After stirring for 1 hour, triflic anhydride (2.2 mL, 13 mmol, 1.0 equiv) was added slowly over 15 minutes. The reaction was then stirred overnight slowly warming to room temperature and quenched with saturated NH_4Cl solution (50 mL). The aqueous layer was extracted with ether (3×20 mL), and the combined organic layers were combined and dried with anhydrous MgSO4. The red crude oil was purified by flash column chromatography (1-10% EtOAc/Hexanes) to give **16** (2.54 g, 94%), Rf = 0.61 (20% EtOAc/Hexanes).

^{1}H NMR (400 MHz, $CDCl_3$): δ 4.22 (q, *J* = 7.15 Hz, 2H), 2.34 (s, 2H), 2.14 (q, *J* = 2.52 Hz, 2H), 1.80 (t, *J* = 2.52 Hz, 3H), 1.31 (t, *J* = 7.15 Hz, 3H), 1.07 (s, 6H) ppm; **^{13}C{^{1}H}NMR** (100 MHz, $CDCl_3$): δ 157.8, 87.3, 78.0, 76.0, 75.0, 61.8, 34.4, 31.7, 30.9, 26.5, 14.1, 3.5 ppm; **HRMS** (ESI): $[M + H]^+$ calculated for $C_{13}H_{19}O_2^+$: 207.1380; found: 207.1377.

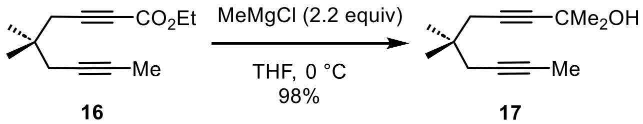

16 **17**

2,6,6-trimethyldeca-3,8-diyn-2-ol (17) MeMgCl (3.0 M in THF, 3.6 mL, 10.7 mmol, 2.2 equiv) was added dropwise to a solution of diyne **16** (1.00 g, 4.85 mmol, 1.0 equiv) in THF (15 mL, 0.32 M) at 0 °C. After stirring for 3 hours at 0 °C, the reaction was diluted with 15 mL diethyl ether and 15 mL of water and quenched with dropwise addition of 1.0 M HCl solution (11 mL). The aqueous layer was repeatedly extracted with diethyl ether (3×15 mL). The combined organic layers were dried over MgSO4 and evaporated under reduced pressure. The colorless crude product was purified by flash column chromatography (5-20% EtOAc/Hexanes) to give **17** (913 mg, 98%) as a colorless oil, R_f= 0.48 (20% EtOAc/Hexanes).

^{1}H NMR (400 MHz, $CDCl_3$) δ 2.16 (s, 2H), 2.10 (q, *J* = 2.5 Hz, 2H), 1.87 (s, 1H), 1.79 (t, *J* = 2.5 Hz, 3H), 1.50 (s, 6H), 1.01 (s, 6H) ppm; **^{13}C{^{1}H}NMR** (100 MHz, $CDCl_3$) δ 87.2, 80.4, 77.6, 76.7, 65.5, 34.2, 31.9, 31.6, 31.2, 26.4, 3.6 ppm; **HRMS** (ESI): $[M + Na]^+$ calculated for $C_{13}H_{20}ONa^+$: 215.1406; found: 215.1407.

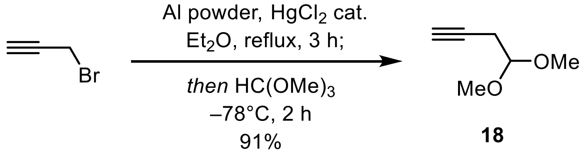

18

4,4-dimethoxybut-1-yne (18). Following the published procedure[3], A solution of propargyl bromide (11.7 mL, 150 mmol, 1.5 equiv) in diethyl ether (15 mL) was added to a refluxing suspension of aluminium powder (6.33 g, 230 mmol, 2.3 equiv) and $HgCl_2$ (360 mg, 1.30 mmol, 0.013 equiv) in diethyl ether (100 mL, 1 M) over 2 hours. The grey suspension was kept under reflux using heating block for 1 hour and cooled to room temperature and then –78 °C. Trimethyl orthoformate (11.1 mL, 100 mol, 1.0 equiv) was added neat to the reaction mixture over 20 minutes. After 2 hours at –78 °C, the reaction was allowed to warm up to room temperature. Water (70 mL) and 1 M aqueous NaOH (20 mL) were added and the mixture was stirred for 15 minutes at room temperature. The resultant insoluble materials were removed by suction filtration using a pad of Celite, and the separated aqueous layer was extracted with Et_2O (3×40 mL). The combined organic layers were washed with brine, dried over MgSO4, and concentrated by rotary evaporation. The resulting light red oil was purified by column chromatography on silica gel (10−20% Et_2O/pentane) to give **18** (10.4 g, 91%) as a light yellow oil, R_f=0.47 (20% EtOAc/Hexanes)**.**

^{1}H NMR (400 MHz, $CDCl_3$) δ 4.55 (t, *J* = 5.6 Hz, 1H), 3.37 (s, 6H), 2.53 (dd, *J* = 5.6, 2.7 Hz, 2H), 2.03 (t, *J* = 2.7 Hz, 1H) ppm. **^{13}C{^{1}H}NMR** (100 MHz, $CDCl_3$) δ 102.4, 79.4, 70.2, 53.6, 23.8 ppm; **HRMS** (ESI): $[M + Na]^+$ calculated for $C_6H_{10}O_2Na^+$: 137.0578; found: 137.0576.

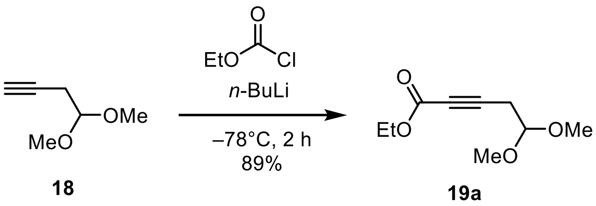

ethyl 5,5-dimethoxypent-2-ynoate (19a). To a solution of alkynyl acetal (500 mg, 4.38 mmol, 1.0 equiv) in tetrahydrofuran (25 mL, 0.18 M) was added *n*-BuLi (3.0 mL, 1.6 M/Hexanes, 1.1 equiv) dropwise at –78 °C over 10 minutes at which point the solution turned purple. After 30 minutes, ethylchloroformate (1.3 mL, 13.1 mmol, 3.0 equiv) was added dropwise. The mixture was stirred at –78 °C for another 2 hours and quenched by saturated NH_4Cl (25 mL) and extracted with Et_2O (3×10 mL). The combined organic layer was washed with brine, dried over MgSO4, filtered and concentrated in vacuo to afford a crude oil. The crude product was purified by column chromatography on silica gel (2−20% EtOAc/Hexanes) to give **19a** (726 mg, 89%) as a colorless oil, R_f = 0.32 (20% EtOAc/Hexanes).

^{1}H NMR (400 MHz, $CDCl_3$) δ 4.60 (t, *J* = 5.6 Hz, 1H), 4.21 (q, *J* = 7.1 Hz, 2H), 3.38 (s, 6H), 2.67 (d, *J* = 5.6 Hz, 2H), 1.30 (t, *J* = 7.1 Hz, 3H) ppm; **^{13}C{^{1}H}NMR** (100 MHz, $CDCl_3$) δ 153.7, 101.6, 83.9, 74.6, 62.0, 53.8, 24.3, 14.1 ppm; **HRMS** (ESI): $[M + Na]^+$ calculated for $C_9H_{14}O_4Na^+$: 209.0784; found: 209.0785.

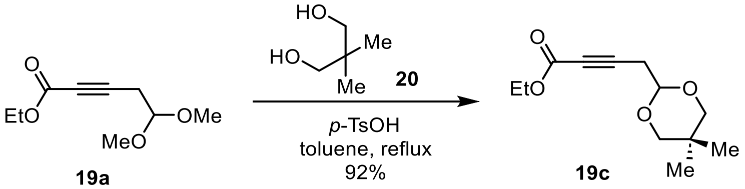

ethyl 4-(5,5-dimethyl-1,3-dioxan-2-yl)but-2-ynoate (19c). To a solution of 3.48 g (18.7 mmol, 1.0 equiv) **19a** in 19 mL of toluene (1.0 M) was added 2,2-dimethylpropane-1,3-diol (2.36 g, 22.4 mmol, 1.2 equiv) and 108 mg of *p*-TsOH (3 mol%). The mixture was refluxed at 110 °C in a heating block for 2 hours. Toluene was evaporated under reduced pressure and the residue was diluted with 20 mL of dichloromethane. To the resulting solution was added 20 mL of sat. $NaHCO_{3(aq)}$ and the aqueous layer was extracted two times with dichloromethane. The combined DCM layers were dried over Na_2SO_4, filtered and concentrated in vacuo. The crude product was purified by column chromatography on silica gel (2−20% EtOAc/Hexanes) to give **19c** (3.89 g, 92%) as a colorless oil, R_f = 0.63 (20% EtOAc/Hexanes).

^{1}H NMR (400 MHz, $CDCl_3$) δ 4.64 (t, *J* = 5.1 Hz, 1H), 4.21 (q, *J* = 7.1 Hz, 2H), 3.63 (d, *J* = 11.3 Hz, 2H), 3.45 (d, *J* = 11.3 Hz, 2H), 2.70 (d, *J* = 5.1 Hz, 2H), 1.29 (t, *J* = 7.1 Hz, 3H), 1.19 (s, 3H), 0.73 (s, 3H) ppm; **^{13}C{^{1}H}NMR** (100 MHz, $CDCl_3$) δ 153.7, 98.7, 83.6, 77.3, 74.7, 62.0, 30.2, 26.0, 23.0, 21.8, 14.1 ppm; **HRMS** (ESI): $[M + Na]^+$ calculated for $C_{12}H_{18}O_4Na^+$: 249.1097; found: 249.1094.

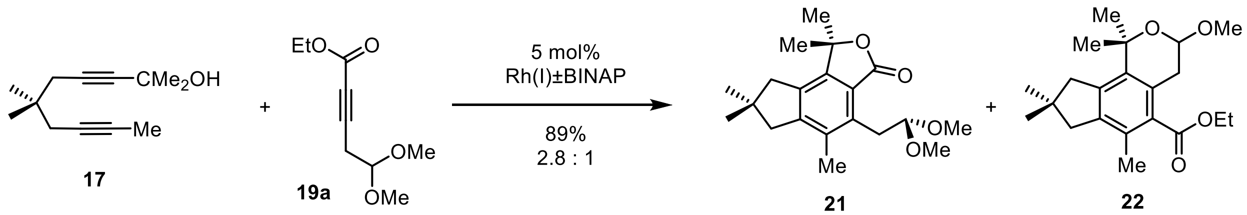

According to general procedure A, a solution of diyne **17** (179 mg, 0.930 mmol, 1.0 equiv) in 3.5 mL DCM was added slowly over 1 h to the solution of 5 mol% active catalyst and alkyne **19a** (381 mg, 2.05 mmol, 2.2 equiv) in 2.0 mL DCM. A separable mixture of compounds **21** (202 mg, white solid), R_f = 0.33 (20% EtOAc/Hexanes) and **22** (75 mg, colorless oil), R_f = 0.45 (20% EtOAc/Hexanes) were obtained (89% combined yield).

4-(2,2-dimethoxyethyl)-1,1,5,7,7-pentamethyl-1,6,7,8-tetrahydro-3H-indeno[4,5-c]furan-3-one (21).

^{1}H NMR (400 MHz, $CDCl_3$) δ 4.57 (t, J = 5.7 Hz, 1H), 3.42 (d, J = 5.7 Hz, 2H), 3.37 (s, 6H), 2.78 (s, 2H), 2.74 (s, 2H), 2.27 (s, 3H), 1.61 (s, 6H), 1.20 (s, 6H) ppm; **^{13}C{^{1}H}NMR** (100 MHz, $CDCl_3$) δ 170.6, 150.7, 148.7, 135.7, 135.0, 133.5, 121.5, 106.6, 83.3, 54.9, 47.6, 45.4, 40.0, 31.8, 29.3, 25.9, 16.1 ppm; **HRMS** (ESI): $[M + Na]^+$ calculated for $C_{20}H_{28}O_4Na^+$: 355.1880; found: 355.1854.

ethyl 6-(2,2-dimethoxyethyl)-7-(2-hydroxypropan-2-yl)-2,2,4-trimethyl-2,3-dihydro-1H-indene-5-carboxylate (22).

^{1}H NMR (400 MHz, $CDCl_3$) δ 4.81 (t, J = 5.3 Hz, 1H), 4.37 (q, J = 7.1 Hz, 2H), 3.50 (s, 3H), 2.85 (d, J = 15.4 Hz, 1H), 2.82 (d, J = 5.3 Hz, 2H), 2.75 (d, J = 15.4 Hz, 1H), 2.63 (d, J = 15.7 Hz, 1H), 2.57 (d, J = 15.7 Hz, 1H), 2.14 (s, 3H), 1.60 (s, 3H), 1.57 (s, 3H), 1.37 (t, J = 7.1 Hz, 3H), 1.18 (s, 3H), 1.09 (s, 3H) ppm; **^{13}C{^{1}H}NMR** (100 MHz, $CDCl_3$) δ 170.4, 142.6, 139.6, 136.5, 133.1, 129.2, 126.4, 96.4, 76.4, 61.2, 55.8, 49.8, 45.9, 39.6, 33.4, 29.4, 28.7, 28.7, 26.8, 16.6, 14.4 ppm; **HRMS** (ESI): $[M + Na]^+$ calculated for $C_{21}H_{30}O_4Na^+$: 369.2036; found: 369.2033.

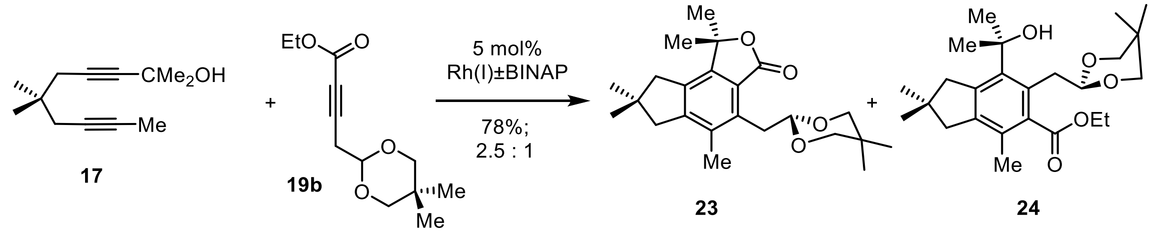

17 19b 23 24

4-((5,5-dimethyl-1,3-dioxan-2-yl)methyl)-1,1,5,7,7-pentamethyl-1,6,7,8-tetrahydro-3H-indeno[4,5-c]furan-3-one (23). ethyl 6-((5,5-dimethyl-1,3-dioxan-2-yl)methyl)-7-(2-hydroxypropan-2-yl)-2,2,4-trimethyl-2,3-dihydro-1H-indene-5-carboxylate (24). According to general procedure A, a solution of diyne **17** (73.0 mg, 0.380 mmol, 1.0 equiv) in 2 mL DCM was added slowly over 1 h to the solution of 5 mol% active catalyst and alkyne **19b** (189 mg, 0.835 mmol, 2.2 equiv) in 1.0 mL DCM. Purification furnished 113 mg white solid containing an inseparable mixture of compounds **23** and **24** (78% combined yield) which was estimated by ^{1}H NMR to be a 2.6:1.0 mixture of **23**:**24**, R_f = 0.37 (20% EtOAc/Hexanes).

^{1}H NMR (400 MHz, $CDCl_3$) δ 5.01 (s, 1H-***24***), 4.78 (t, J = 5.6 Hz, 1H-***23***), 4.74 (t, J = 6.1 Hz, 1H-***24***), 4.39 (q, J = 7.2 Hz, 2H-***24***), 3.60 (d, J = 11.3 Hz, 2H-***24***), 3.54 (d, J = 11.3 Hz, 2H-***23***), 3.45 (d, J = 5.6 Hz, 2H-***23***), 3.42 (d, J = 11.3 Hz, 2H-***24***), 3.36 (d, J = 11.3 Hz, 2H-***23***), 2.86 (s, 2H-***24***), 2.77 (s, 2H-***23***), 2.75 (s, 2H-***23***), 2.58 (s, 2H-***24***), 2.31 (s, 3H-***23***), 2.10 (s, 3H-***24***), 1.61 (s, 6H-***23***), 1.57 (s, 6H-***24***), 1.38 (t, J = 7.1 Hz, 3H-***24***), 1.19 (s, 6H-***23***), 1.20 (s, 3H-***24***), 1.21 (s, 3H-***23***), 0.70 (s, 3H-***24***), 0.67 (s, 3H-***23***) ppm; **^{13}C{^{1}H}NMR** (100 MHz, $CDCl_3$) δ 171.7, 170.5, 150.6, 148.7, 143.8, 143.2, 141.4, 137.0, 136.1, 134.4, 133.6, 128.1, 127.0, 121.8, 102.7, 102.4, 77.4, 77.3, 75.7, 61.3, 52.5, 47.7, 45.8, 45.5, 40.0, 39.3, 36.0, 33.0, 30.3, 30.3, 29.8, 29.3, 28.4, 25.9, 23.2, 23.1, 22.0, 21.8, 16.6, 16.2, 14.4 ppm; **HRMS** (ESI): $[M + H]^+$ calculated for $C_{23}H_{33}O_4^+$ and $[M + Na]^+$ calculated for $C_{25}H_{38}O_5Na^+$: 373.2373 and 441.2611; found: 373.2372 and 441.2610.

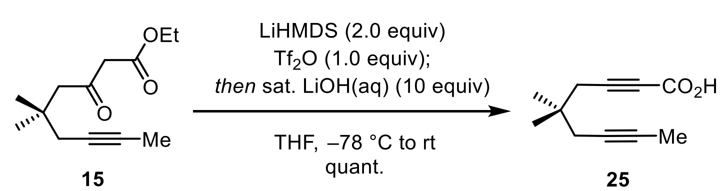

15 25

5,5-dimethylnona-2,7-diynoic acid (25). A round bottom flask was charged with dry THF (52 mL, 0.25 M) and LiHMDS (26 mL, 1.0 M/THF, 26 mmol, 2.0 equiv). The solution was then cooled to −78 °C followed by the addition of a solution of β-keto ester **15** (3.04 g, 13 mmol, 1.0 equiv) over 10 minutes. After stirring for 1 hour, triflic anhydride (2.2 mL, 13 mmol, 1.0 equiv) was added slowly over 15 minutes. The reaction was then stirred overnight slowly warming to room temperature (12 h). A solution of sat. LiOH(aq) (26 mL, ~10 eqiuv) was then added to the reaction mixture at room temperature and stirring was continued for an additional 12 h. The reaction was diluted with water (20 mL) and acidified with 3 M HCl solution (50 mL) until pH~5. The aqueous layer was extracted with EtOAc (3×25 mL), and the combined organic layers were combined and dried with anhydrous Na_2SO4. The solvent was evaporated

and the crude yellow oil was purified by flash column chromatography (10-30% EtOAc/Hexanes) to give **25** (2.32 g, quant) , $R_f = 0.35$ (20% EtOAc/Hexanes).

^{1}H NMR (400 MHz, $CDCl_3$): δ 2.38 (s, 2H), 2.14 (q, *J* = 2.48 Hz, 2H), 1.80 (t, *J* = 2.48 Hz, 3H), 1.08 (s, 6H) ppm; **^{13}C{^{1}H}NMR** (100 MHz, $CDCl_3$): δ 157.8, 90.7, 78.2, 75.8, 74.33, 34.4, 31.7, 26.5, 3.5 ppm; **HRMS** (ESI): $[M-H]^-$ calculated for $C_{11}H_{13}O_2^-$: 177.0916; found: 177.0917.

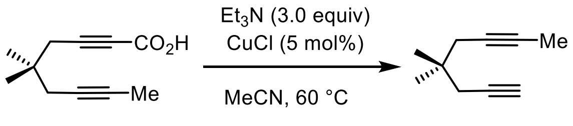

25 26

4,4-dimethylocta-1,6-diyne (26). Diyne acid **25** (2.47 g, 13.86 mmol, 1.0 equiv) and CuCl (67.0 mg, 5 mol%) were added to a round bottom flask. The flask was sealed with a rubber stopper, evacuated and backfilled with nitrogen for three times. MeCN (70 mL, 0.2 M) and Et_3N (5.8 mL, 41.6 mmol, 3.0 equiv) were added respectively, and the reaction was heated at 60 °C in a heating block for 2 hours under positive nitrogen pressure until no remaining starting material was observed by TLC. The reaction mixture was cooled to room temperature and partitioned between 1.0 M HCl (50 mL) and pentane (100 mL). The aqueous layer was extracted with pentane (3×30 mL), and the combined organic layers were washed with brine, dried over $MgSO_4$, and filtered through a pad of silica gel. Since the product is relatively volatile, the resulting clear solution was partially concentrated by rotary evaporation in an ice bath to give a solution of the corresponding diyne mono methyl **26** in approximately 5 mL of pentane, R_f=0.24 (100% Hexanes). This crude product in pentane was used for the next step without further purification.

^{1}H NMR (400 MHz, $CDCl_3$): δ 2.19 (d, *J* = 2.67 Hz, 2H), 2.13 (q, *J* = 2.57 Hz, 2H), 2.00 (t, *J* = 2.67 Hz, 1H), 1.80 (t, *J* = 2.57 Hz, 3H), 1.04 (s, 6H) ppm; **^{13}C{^{1}H}NMR** (100 MHz, $CDCl_3$): δ 82.3, 77.5, 76.4, 70.0, 33.9, 31.4, 30.8, 26.3, 3.50 ppm.

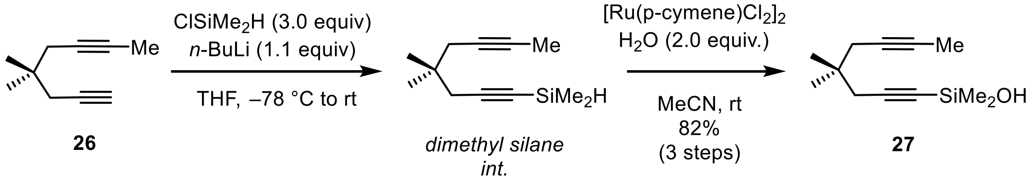

26 *dimethyl silane int.* 27

(4,4-dimethylocta-1,6-diyn-1-yl)dimethylsilanol (27)

To the 100-mL round-bottom flask containing the previous solution of diyne **26** in 5 mL of pentane was added THF (28 mL, 0.5 M). The resulting solution was cooled to −78 °C. To the solution was slowly added *n*-BuLi (8.7 mL, 1.6 M/hexane, 13.9 mmol, 1.0 equiv) and stirred for 1 h at the same temperature. Chlorodimethylsilane (4.8 mL, 41.6 mmol, 3.0 equiv) was added and the solution was allowed to warm to room temperature over 3 hours. The reaction mixture was quenched with 40 mL of water and extracted with pentane (3×20 mL). The combined organics were washed with brine and sat. $NaHCO_3$, dried over MgSO4, and filtered through a pad of silica gel. The volatile solvents were evaporated in vacuo to provide a colorless oil which was found to be pure and consistent with the intermediate diyne dimethylsilane by ^{1}H NMR analysis: **^{1}H NMR** (400 MHz, $CDCl_3$) δ 2.22 (d, *J* = 1.3 Hz, 2H), 2.11 (q, *J* = 2.6 Hz, 2H), 1.79 (t, *J* = 2.6 Hz, 3H), 1.03 (s, 6H), 0.22 (d, *J* = 3.8 Hz, 6H) ppm. This crude product was diluted with acetonitrile (55 mL, 0.25 M) and water (0.50 mL, 27.7 mmol, 2.0 equiv) and $[RuCl_2(p\text{-cymene})]_2$ (170 mg, 2 mol%) were added respectively. The reaction mixture was stirred at room temperature and under open air for 1 hour until bubbling ceased. Acetonitrile was removed in vacuo and the crude residue was purified by flash column chromatography (5-20% EtOAc/Hexanes) to give **27** (2.37 g, 82% over 3 steps) as a colorless oil, $R_f = 0.39$ (20% EtOAc/Hexanes).

^{1}H NMR (400 MHz, $CDCl_3$) δ 2.21 (s, 2H), 2.11 (q, *J* = 2.5 Hz, 2H), 2.01 (s, 1H), 1.79 (t, *J* = 2.5 Hz, 3H), 1.03 (s, 6H), 0.24 (s, 6H) ppm; **^{13}C{^{1}H}NMR** (100 MHz, $CDCl_3$) δ 105.5, 85.7, 77.8, 76.6, 34.2, 32.2, 31.6, 26.4, 3.6, 1.8 ppm; **HRMS** (ESI): $[M + Na]^+$ calculated for $C_{12}H_{20}OSiNa^+$: 231.1176; found: 231.1177.

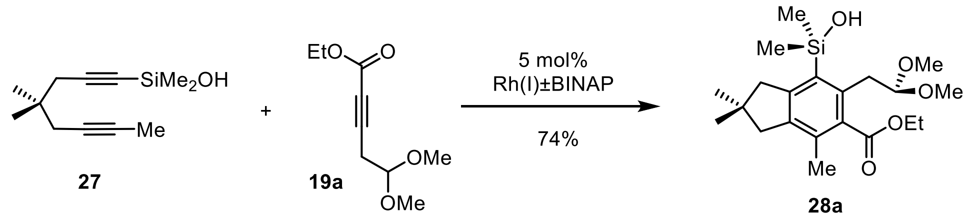

ethyl 6-(2,2-dimethoxyethyl)-7-(hydroxydimethylsilyl)-2,2,4-trimethyl-2,3-dihydro-1H-indene-5-carboxylate (28a). According to general procedure A, a solution of diyne **27** (71.0 mg, 0.341 mmol, 1.0 equiv) in 2.5 mL DCM was added slowly over 1 h to the solution of 5 mol% active catalyst and alkyne **19a** (140 mg, 0.750 mmol, 2.2 equiv) in 1.4 mL DCM. Single regioisomer **28a** (100 mg, 74% yield) was obtained after purification as a white solid, $R_f = 0.33$ (20% EtOAc/Hexanes).

^{1}H NMR (400 MHz, $CDCl_3$) δ 4.62 (t, $J = 5.6$ Hz, 1H), 4.59 (s, 1H), 4.39 (q, $J = 7.2$ Hz, 2H), 3.33 (s, 6H), 3.25 (br s, 2H), 2.73 (s, 2H), 2.59 (s, 2H), 2.14 (s, 3H), 1.38 (t, $J = 7.1$ Hz, 3H), 1.11 (s, 6H), 0.35 (s, 6H) ppm; **^{13}C{^{1}H}NMR** (100 MHz, $CDCl_3$) δ 171.6, 149.5, 141.1, 135.9, 134.6, 134.4, 131.3, 106.2, 61.3, 54.2, 50.6, 45.6, 39.5, 36.7, 28.8, 16.9, 14.4, 2.6 ppm; **HRMS** (ESI): $[M + Na]^+$ calculated for $C_{21}H_{34}O_5SiNa^+$: 417.2068; found: 417.2064.

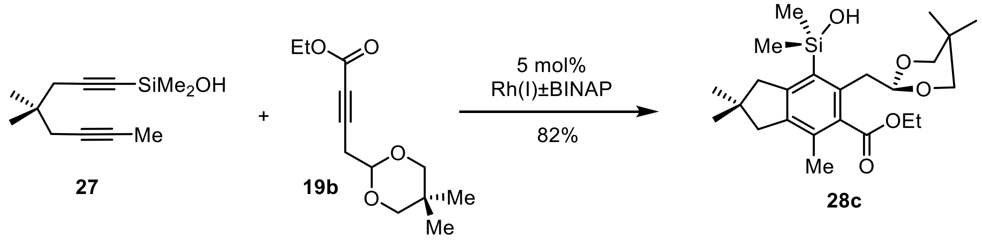

ethyl 6-((5,5-dimethyl-1,3-dioxan-2-yl)methyl)-7-(hydroxydimethylsilyl)-2,2,4-trimethyl-2,3-dihydro-1H-indene-5-carboxylate (28c). According to general procedure A, a solution of diyne **27** (50.0 mg, 0.240 mmol, 1.0 equiv) in 1.5 mL DCM was added slowly over 1 h to the solution of 5 mol% active catalyst and alkyne **19b** (119 mg, 0.528 mmol, 2.2 equiv) in 1.0 mL DCM. Single regioisomer **28c** (85.0 mg, 82% yield) was obtained after purification as a white solid, $R_f = 0.44$ (20% EtOAc/Hexanes).

^{1}H NMR (400 MHz, $CDCl_3$) δ 4.81 (s, 1H), 4.72 (t, $J = 5.5$ Hz, 1H), 4.39 (q, $J = 7.1$ Hz, 2H), 3.61 (d, $J = 11.4$ Hz, 2H), 3.42 (d, $J = 11.3$ Hz, 2H), 3.30 (s, 2H), 2.75 (s, 2H), 2.60 (s, 2H), 2.15 (s, 3H), 1.38 (t, $J = 7.1$ Hz, 3H), 1.21 (s, 3H), 1.12 (s, 6H), 0.71 (s, 3H), 0.36 (s, 6H) ppm; **^{13}C{^{1}H}NMR** (100 MHz, $CDCl_3$) δ 171.5, 149.5, 141.3, 135.2, 134.6, 131.4, 103.2, 77.4, 61.3, 50.7, 45.7, 39.4, 37.9, 30.3, 28.9, 23.0, 21.8, 16.9, 14.4, 2.6 ppm; **HRMS** (ESI): $[M + Na]^+$ calculated for $C_{24}H_{38}O_5SiNa^+$: 457.2381; found: 457.2375.

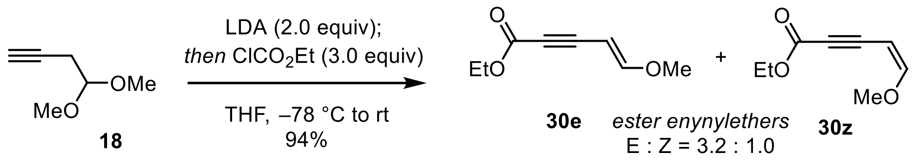

n-BuLi (1.6 M in hexanes, 2.5 mL, 4.00 mmol, 2.0 equiv) was added dropwise to a solution of diisopropylamine (0.57 mL, 4.00 mmol, 2.0 equiv) in THF (8.0 mL) at −78 °C. The resulting solution was stirred at this temperature for 0.5 h. A solution of alkyne **18** (228 mg, 2.00 mmol, 1.0 equiv) in THF (2.0 mL) was then added dropwise over 10 minutes. The resulting mixture was stirred at −78 °C for 1 h followed by dropwise addition of ethylchloroformate (0.6 mL, 6.00 mmol, 3.0 equiv) at −78 °C. The reaction mixture was allowed to warm up to room temperature over 3 h. The reaction was quenched by the addition of saturated NH_4Cl solution (15 mL). The aqueous layer was further extracted with ether (3×10 mL). The combined organic layers were dried over anhydrous MgSO4, filtered, and concentrated under vacuum to afford a red oil. Purification by flash column chromatography (1-10% EtOAc/Hexanes) furnished 266 mg of the *E*-isomer (**30e**), $R_f = 0.34$ (20% EtOAc/Hexanes) and 83 mg of the *Z*-isomer (**30z**), $R_f = 0.26$ (20%

EtOAc/Hexanes) as light yellow oils (94% combined yield). These products were indefinitely stable when stored in fridge (2 °C).

ethyl (E)-5-methoxypent-4-en-2-ynoate (30e)

^{1}H NMR (400 MHz, $CDCl_3$) δ 7.16 (d, J = 12.7 Hz, 1H), 4.91 (d, J = 13.0 Hz, 1H), 4.22 (q, J = 7.1 Hz, 2H), 3.67 (s, 3H), 1.30 (t, J = 7.2 Hz, 3H); **^{13}C{^{1}H}NMR** (100 MHz, $CDCl_3$) δ 164.2, 154.5, 85.7, 82.3, 81.0, 61.8, 57.4, 14.2 ppm; **HRMS** (ESI): $[M + H]^+$ calculated for $C_8H_{11}O_3^+$: 155.0703; found: 155.0700.

ethyl (Z)-5-methoxypent-4-en-2-ynoate (30z)

^{1}H NMR (400 MHz, $CDCl_3$) δ 6.54 (d, J = 6.3 Hz, 1H), 4.61 (d, J = 6.3 Hz, 1H), 4.22 (q, J = 7.1 Hz, 2H), 3.84 (s, 3H), 1.30 (t, J = 7.1 Hz, 3H) ppm; **^{13}C{^{1}H}NMR** (100 MHz, $CDCl_3$) δ 162.0, 154.4, 84.6, 82.5, 82.5, 61.9, 61.4, 14.2 ppm; **HRMS** (ESI): $[M + H]^+$ calculated for $C_8H_{11}O_3^+$: 155.0703; found: 155.0700.

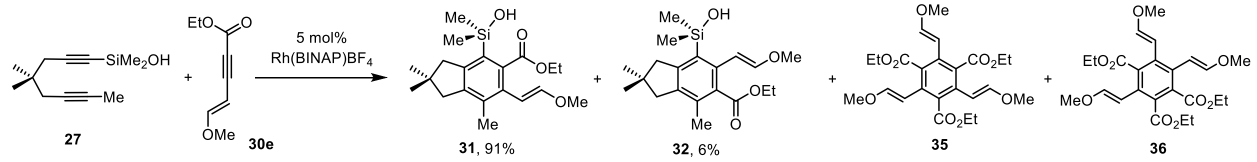

According to general procedure A, a solution of diyne **27** (47.7 mg, 0.229 mmol, 1.0 equiv) and alkyne **30e** (77.6 mg, 0.504 mmol, 2.2 equiv) in 3.0 mL DCM was added slowly over 1 h to the solution of 5 mol% active catalyst. After purification, an inseparable mixture of compounds **31** and **32** (81.0 mg, 97% yield) were obtained as a thick colorless oil which was estimated by ^{1}H NMR to be a 94:6.0 mixture of **31**:**32,** R_f = 0.28 (20% EtOAc/Hexanes). A mixture containing 15.0 mg benzene derivatives **35** and **36** were also generated as a result of partially self-cyclotrimerization of reactive alkyne **19a**, R_f = 0.1 (20% EtOAc/Hexanes). The extent of cyclotrimerization depends on the duration of the reaction and the excess alkyne can be fully consumed.

ethyl (E)-4-(hydroxydimethylsilyl)-6-(2-methoxyvinyl)-2,2,7-trimethyl-2,3-dihydro-1H-indene-5-carboxylate (31).

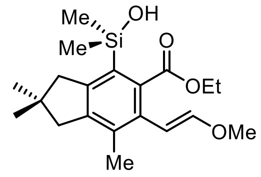

31

^{1}H NMR (400 MHz, $CDCl_3$) δ 6.43 (d, J = 13.1 Hz, 1H), 5.70 (d, J = 13.0 Hz, 1H), 4.27 (q, J = 7.2 Hz, 2H), 3.65 (s, 3H), 2.79 (s, 2H), 2.63 (s, 2H), 2.17 (s, 3H), 1.33 (t, J = 7.2 Hz, 3H), 1.13 (s, 6H), 0.38 (s, 6H) ppm; **^{13}C{^{1}H}NMR** (100 MHz, $CDCl_3$) δ 173.4, 150.6, 146.8, 143.7, 138.4, 135.2, 130.2, 129.0, 101.0, 61.3, 56.3, 50.2, 46.5, 39.4, 29.1, 17.1, 14.2, 1.9 ppm; **HRMS** (ESI): $[M + Na]^+$ calculated for $C_{20}H_{30}O_4SiNa^+$: 385.1806; found: 385.1803.

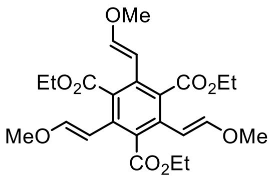

35

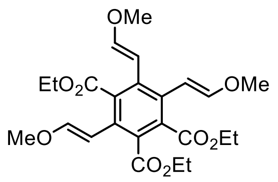

36

^{1}H NMR (400 MHz, $CDCl_3$) 6.72 – 6.42 (m, 3H), 5.87 – 5.54 (m, 3H), 4.28 (q, J = 7.2 Hz, 6H), 3.64 (s, 6H), 3.60 (s, 3H), 1.31 (m, 9H) ppm; **^{13}C{^{1}H}NMR** (100 MHz, $CDCl_3$) δ 168.9, 168.5, 167.8, 152.1, 152.0, 151.9, 136.3, 134.8, 133.5, 131.8, 130.4, 130.2, 100.9, 100.6, 99.3, 61.7, 61.4, 56.5, 56.3, 14.3, 14.2, 14.1 ppm; **HRMS** (ESI): $[M + Na]^+$ calculated for $C_{24}H_{30}O_9Na^+$: 485.1782; found: 485.1760.

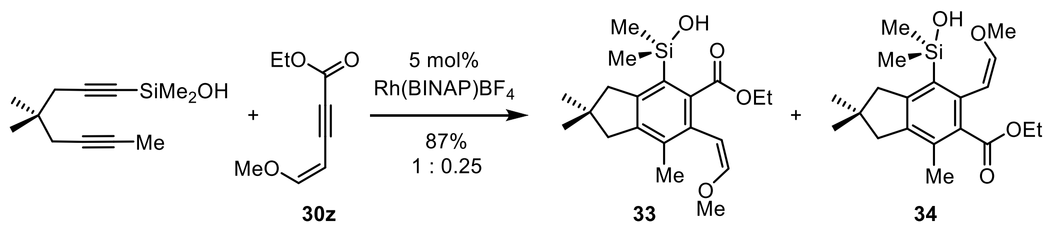

According to general procedure A, a solution of diyne **27** (95.0 mg, 0.456 mmol, 1.0 equiv) and alkyne **30z** (141 mg, 0.912 mmol, 2.0 equiv) in 2.5 mL DCM was added slowly over 1 h to the solution of 5 mol% active catalyst in 1.0

mL DCM. After purification an inseparable mixture of compounds **33** and **34** (143 mg, 87% yield) which was estimated by ^{1}H NMR to be a 1.00:0.25 mixture of **33**:**34,** R_f = 0.45 (30% EtOAc/Hexanes).

ethyl (Z)-4-(hydroxydimethylsilyl)-6-(2-methoxyvinyl)-2,2,7-trimethyl-2,3-dihydro-1H-indene-5-carboxylate (33).

^{1}H NMR (400 MHz, $CDCl_3$) δ 6.10 (d, J = 6.8 Hz, 1H), 5.30 (d, J = 6.8 Hz, 1H), 4.29 (q, J = 7.2 Hz, 2H), 3.61 (s, 3H), 2.79 (s, 2H), 2.65 (s, 2H), 2.32 (s, 1H), 2.15 (s, 3H), 1.35 (t, J = 7.2 Hz, 3H), 1.14 (s, 6H), 0.37 (s, 6H) ppm; **^{13}C{^{1}H}NMR** (100 MHz, $CDCl_3$) δ 173.0, 147.7, 146.7, 143.7, 137.9, 135.1, 129.6, 128.7, 102.4, 61.2, 59.9, 50.2, 46.5, 39.2, 29.1, 17.1, 14.1, 1.9 ppm; **HRMS** (ESI): [M + Na]$^+$ calculated for $C_{20}H_{30}O_4SiNa^+$: 385.1806; found: 385.1803.

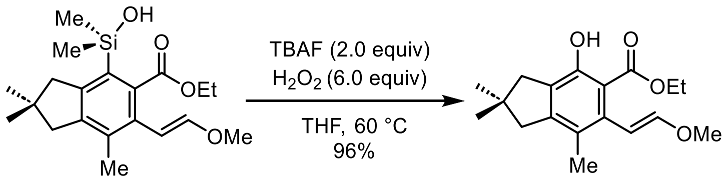

31 **37**

ethyl (E)-4-hydroxy-6-(2-methoxyvinyl)-2,2,7-trimethyl-2,3-dihydro-1H-indene-5-carboxylate (37). To a stirred solution of silanol **31** (103 mg, 0.283 mmol, 1.0 equiv) in 1.5 mL THF (0.2 M) was added TBAF (0.57 mL, 0.56 mmol, 2.0 equiv) and 50% H_2O_2 (116 mg, 1.70 mmol, 6.0 equiv). The reaction mixture was stirred at 60 °C in a heating block for 24 h before cooling down to room temperature and concentrating by rotary evaporation. The crude residue was purified by flash column chromatography (3-10% EtOAc/Hexanes) to give **37** (83 mg, 96%) as a colorless thick oil, Rf = 0.65 (20% EtOAc/Hexanes).

^{1}H NMR (400 MHz, $CDCl_3$) δ 10.54 (s, 1H), 6.18 (d, J = 13.1 Hz, 1H), 5.93 (d, J = 13.5 Hz, 1H), 4.35 (q, J = 7.2 Hz, 2H), 3.69 (s, 3H), 2.75 (s, 1H), 2.69 (s, 2H), 2.11 (s, 3H), 1.37 (t, J = 7.2 Hz, 3H), 1.17 (s, 6H) ppm; **^{13}C{^{1}H}NMR** (100 MHz, $CDCl_3$) δ 172.1, 155.7, 150.4, 149.0, 135.0, 128.6, 125.1, 111.9, 103.7, 61.3, 56.1, 48.3, 44.3, 39.4, 29.5, 17.1, 14.3 ppm; **HRMS** (ESI): [M – H]$^-$ calculated for $C_{18}H_{23}O_4^-$: 303.1602; found: 303.1612.

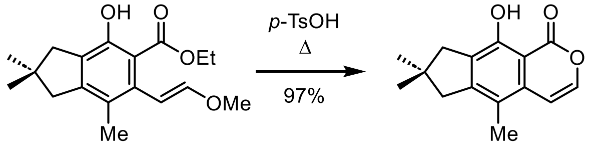

37 **5**

9-hydroxy-7-(hydroxymethyl)-5,7-dimethyl-7,8- dihydrocyclopenta[g]isochromen-1(6H)-one (5). To a solution of **37** (55.0 mg, 0.181 mmol, 1.0 equiv) in toluene (4.6 mL, 0.04 M) was added p-TsOH.H_2O (69.0 mg, 0.361 mmol, 2.0 equiv) and the solution was refluxed in a heating block for 24 h. The solution was cooled down to rt and diluted with DCM and transferred to an evaporating flask. The volatiles were removed in vacuo and the crude residue was loaded onto silica gel and chromatographed using a 5-20% EtOAc/Hexanes eluent to afford **5** as a white solid (43 mg, 97%), Rf = 0.45 (20% EtOAc/Hexanes).

^{1}H NMR (400 MHz, $CDCl_3$) δ 11.07 (s, 1H), 7.19 (d, J = 5.8 Hz, 1H), 6.62 (d, J = 5.8 Hz, 1H), 2.81 (s, 2H), 2.78 (s, 2H), 2.23 (s, 3H), 1.19 (s, 6H) ppm; **^{13}C{^{1}H}NMR** (100 MHz, $CDCl_3$) δ 167.1, 155.9, 154.4, 142.6, 133.4, 129.3, 119.4, 105.9, 105.6, 48.2, 44.0, 39.7, 29.2, 14.4 ppm; **HRMS** (ESI): [M – H]$^-$ calculated for $C_{15}H_{15}O_3^-$: 243.1027; found: 243.1034.

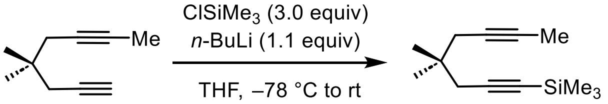

26 **38**

(4,4-dimethylocta-1,6-diyn-1-yl)trimethylsilane (38). To a solution of diyne **26** (309 mg, 2.30 mmol, 1.0 equiv) in 5 mL of THF (0.5 M) at −78 °C was slowly added LDA (2.3 mL, 1.0 M/THF, 2.30 mmol, 1.0 equiv) and stirred for 1 h at the same temperature. Chlorotrimethylsilane (0.90 mL, 6.90 mmol, 3.0 equiv) was added and the solution was allowed to warm to room temperature over 3 hours. The reaction mixture was quenched with 10 mL of water and extracted with pentane (3×10 mL). The combined organics were washed with brine, dried over MgSO4, and filtered through a pad of silica gel. The filtrate was concentrated by rotary evaporation to provide **38** as a colorless oil which was found to be pure by ^{1}H NMR (403 mg, 85%), R_f= 0.75 (20% EtOAc/Hexanes).

^{1}H NMR (400 MHz, $CDCl_3$) δ 2.20 (s, 2H), 2.11 (q, *J* = 2.6 Hz, 2H), 1.80 (t, *J* = 2.6 Hz, 3H), 1.03 (s, 6H), 0.15 (s, 9H) ppm; **^{13}C{^{1}H}NMR** (100 MHz, $CDCl_3$) δ 105.4, 86.6, 77.6, 76.8, 34.2, 32.5, 31.6, 26.3, 3.6, 0.3 ppm; **HRMS** (ESI): $[M + Na]^+$ calculated for $C_{13}H_{22}SiNa^+$: 229.1383; found: 229.1378.

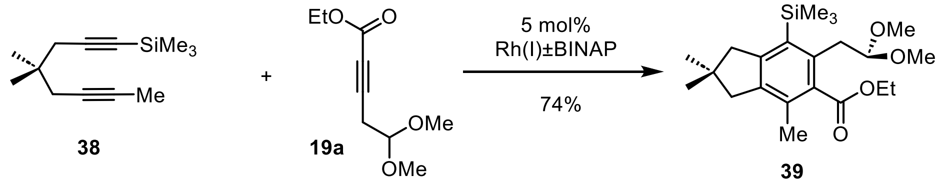

ethyl 6-(2,2-dimethoxyethyl)-2,2,4-trimethyl-7-(trimethylsilyl)-2,3-dihydro-1H-indene-5-carboxylate (39). According to general procedure A, a solution of diyne **38** (50 mg, 0.242 mmol, 1.0 equiv) in 1.5 mL DCM was added slowly over 1 h to the solution of 5 mol% active catalyst and alkyne **19a** (100 mg, 0.533 mmol, 2.2 equiv) in 1.2 mL DCM. Single regioisomer **39** (70.3 mg, 74% yield) was obtained after purification as a colorless oil, R_f = 0.55 (20% EtOAc/Hexanes).

^{1}H NMR (400 MHz, $CDCl_3$) δ 4.38 (q, *J* = 7.1 Hz, 2H), 4.38 (t, *J* = 5.6 Hz, 1H), 3.24 (s, 6H), 3.08 (d, *J* = 5.6 Hz, 2H), 2.82 (s, 2H), 2.59 (s, 2H), 2.14 (s, 3H), 1.39 (t, *J* = 7.1 Hz, 3H), 1.12 (s, 6H), 0.36 (s, 9H) ppm; **^{13}C{^{1}H}NMR** (100 MHz, $CDCl_3$) δ 171.6, 151.0, 140.8, 136.7, 134.7, 134.0, 131.0, 107.2, 61.1, 54.8, 50.9, 45.6, 39.5, 37.5, 28.7, 16.9, 14.4, 3.2 ppm; **HRMS** (ESI): $[M + Na]^+$ calculated for $C_{22}H_{36}O_4SiNa^+$: 415.2275; found: 415.2265.

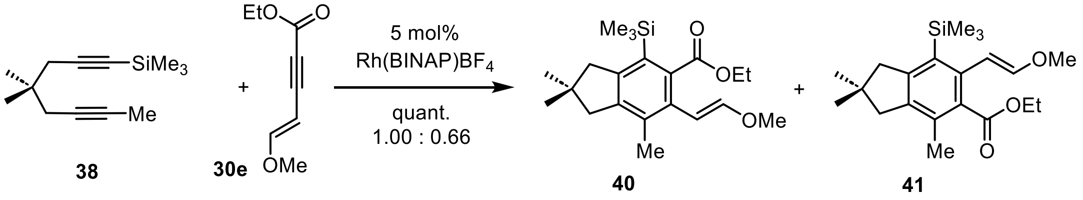

According to general procedure A, a solution of diyne **38** (100 mg, 0.484 mmol, 1.0 equiv) and alkyne **30e** (112 mg, 0.726 mmol, 1.5 equiv) in 4.0 mL DCM was added slowly over 1 h to the solution of 5 mol% active catalyst in 1.5 mL DCM. After purification an inseparable mixture of compounds **40** and **41** (173 mg, quant.) which was estimated by ^{1}H NMR to be a 1.00:0.66 mixture of **40**:**41**, R_f = 0.61 (20% EtOAc/Hexanes).

ethyl (E)-6-(2-methoxyvinyl)-2,2,4-trimethyl-7-(trimethylsilyl)-2,3-dihydro-1H-indene-5-carboxylate (40) and ethyl (E)-6-(2-methoxyvinyl)-2,2,7-trimethyl-4-(trimethylsilyl)-2,3-dihydro-1H-indene-5-carboxylate (41). Colorless oil

^{1}H NMR (400 MHz, $CDCl_3$) δ 6.43 (d, *J* = 13.1 Hz, 1H-**major**), 6.32 (d, *J* = 13.0 Hz, 1H-minor), 5.91 (d, *J* = 13.0 Hz, 1H-**minor**), 5.66 (d, *J* = 13.1 Hz, 1H-**major**), 4.31 (q, *J* = 7.2 Hz, 1H-**minor**), 4.25 (q, *J* = 7.2 Hz, 2H-**major**), 3.64 (s, 3H-**major**), 3.62 (s, 3H-**minor**), 2.81 (s, 2H-**major**), 2.80 (s, 2H-**minor**), 2.62 (s, 2H-**major**), 2.60 (s, 2H-**minor**), 2.16 (s, 3H-**major** and **minor**), 1.34 (t, *J* = 7.2 Hz, 3H-**minor**), 1.32 (t, *J* = 7.2 Hz, 3H-**major**), 1.14 (s, 6H-**major**), 1.13 (s, 6H-**minor**), 0.30 (s, 9H-**minor**), 0.27 (s, 9H-**major**) ppm; **^{13}C{^{1}H}NMR** (100 MHz, $CDCl_3$) δ 171.9, 171.2, 150.8, 150.4, 150.0, 147.9, 143.3, 141.0, 139.0, 136.9, 134.7, 134.1, 133.4, 131.0, 129.8, 128.5, 105.0, 100.7, 60.9, 56.2, 56.1, 50.8, 50.6, 46.4, 45.8, 39.4, 39.1, 29.1, 29.0, 17.2, 16.8, 14.4, 14.2, 3.0, 1.0. ppm; **HRMS** (ESI): $[M + Na]^+$ calculated for $C_{21}H_{32}O_3SiNa^+$: 383.2013; found: 383.2006.

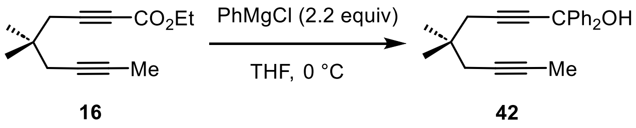

5,5-dimethyl-1,1-diphenylnona-2,7-diyn-1-ol (42). PhMgCl (2.0 M in THF, 7.7 mL, 15.4 mmol, 2.2 equiv) was added dropwise to a solution of diyne **16** (1.44 g, 7.00 mmol, 1.0 equiv) in THF (20 mL, 0.35 M) at 0 °C. After stirring for 3 hours at 0 °C, the reaction was diluted with 20 mL diethyl ether and 30 mL of half saturated solution of NH_4Cl. The aqueous layer was repeatedly extracted with diethyl ether (3×15 mL). The combined organic layers were dried over MgSO4 and evaporated under reduced pressure. The colorless crude product was purified by flash column chromatography (3-10% EtOAc/Hexanes) to give **42** (2.04 g, 92%) as a colorless oil, R_f= 0.57 (20% EtOAc/Hexanes).

^{1}H NMR (400 MHz, $CDCl_3$) δ 7.62 (m, 4H), 7.36 – 7.21 (m, 6H), 2.71 (s, 1H), 2.35 (s, 2H), 2.15 (q, *J* = 2.6 Hz, 2H), 1.81 (t, *J* = 2.6 Hz, 3H), 1.07 (s, 6H) ppm; **^{13}C{^{1}H}NMR** (100 MHz, $CDCl_3$) δ 145.6, 128.3, 127.7, 126.1, 86.2, 85.1, 77.8, 76.6, 74.7, 34.5, 31.8, 31.5, 26.7, 3.7. ppm; **HRMS** (ESI): $[M + Na]^+$ calculated for $C_{23}H_{24}ONa^+$: 339.1719; found: 339.1708.

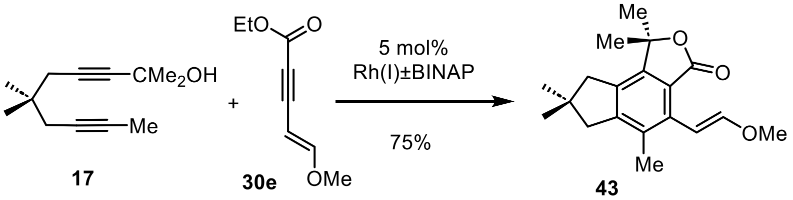

(E)-4-(2-methoxyvinyl)-1,1,5,7,7-pentamethyl-1,6,7,8-tetrahydro-3H-indeno[4,5-c]furan-3-one (43). According to general procedure A, a solution of diyne **17** (120 mg, 0.624 mmol, 1.0 equiv) and alkyne **30e** (191 mg, 1.25 mmol, 2.0 equiv) in 5.0 mL DCM was added slowly over 1 h to the solution of 5 mol% active catalyst in 1.6 mL DCM. Single regioisomer **43** (140 mg, 75% yield) was obtained as a white solid, R_f = 0.30 (20% EtOAc/Hexanes).

^{1}H NMR (400 MHz, $CDCl_3$) δ 6.90 (d, *J* = 13.3 Hz, 1H), 6.45 (d, *J* = 13.3 Hz, 1H), 3.78 (s, 3H), 2.78 (s, 2H), 2.74 (s, 2H), 2.29 (s, 3H), 1.60 (s, 6H), 1.20 (s, 6H) ppm; **^{13}C{^{1}H}NMR** (100 MHz, $CDCl_3$) δ 170.4, 153.4, 151.0, 149.2, 134.4, 132.3, 132.2, 120.2, 98.5, 82.6, 56.3, 47.7, 45.4, 40.1, 29.2, 25.8, 17.6 ppm; **HRMS** (ESI): $[M + Na]^+$ calculated for $C_{19}H_{24}O_3Na^+$: 323.1618; found: 323.1609.

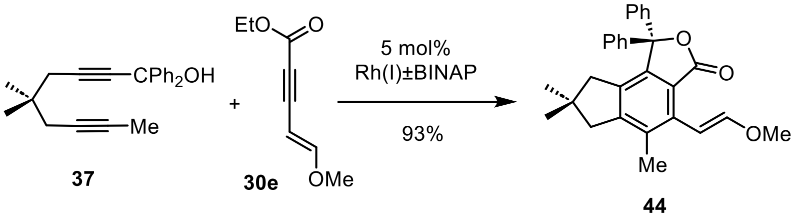

(E)-4-(2-methoxyvinyl)-5,7,7-trimethyl-1,1-diphenyl-1,6,7,8-tetrahydro-3H-indeno[4,5-c]furan-3-one (44). According to general procedure A, a solution of diyne **42** (73.0 mg, 0.231 mmol, 1.0 equiv) and alkyne **30e** (55.0 mg, 0.346 mmol, 1.5 equiv) in 2.0 mL DCM was added slowly over 1 h to the solution of 5 mol% active catalyst in 0.6 mL DCM. Single regioisomer **44** (86.0 mg, 88% yield) was obtained as a white solid, R_f = 0.46 (20% EtOAc/Hexanes).

^{1}H NMR (400 MHz, $CDCl_3$) δ 7.36 – 7.14 (m, 10H), 6.90 (d, *J* = 13.2 Hz, 1H), 6.48 (d, *J* = 13.2 Hz, 1H), 3.76 (s, 3H), 2.65 (s, 2H), 2.34 (s, 2H), 2.28 (s, 3H), 1.04 (s, 6H) ppm; **^{13}C{^{1}H}NMR** (100 MHz, $CDCl_3$) δ 170.6, 153.7, 151.6, 146.8, 139.7, 135.1, 135.0, 133.0, 128.5, 128.4, 128.2, 120.2, 98.4, 89.8, 56.4, 47.6, 46.4, 40.3, 28.7, 17.7. ppm; **HRMS** (ESI): $[M + Na]^+$ calculated for $C_{29}H_{28}O_3Na^+$: 447.1931; found: 447.1915.

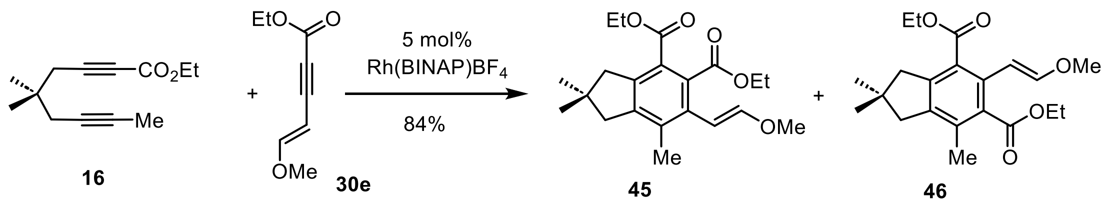

According to general procedure A, a solution of diyne **16** (100 mg, 0.485 mmol, 1.0 equiv) and alkyne **30e** (mg, 0.97 mmol, 2.0 equiv) in 4.0 mL DCM was added slowly over 1 h to the solution of 5 mol% active catalyst in 1.0 mL DCM. A separable mixture of compounds **45** (105 mg), $R_f = 0.31$ (20% EtOAc/Hexanes) and **46** (43 mg), $R_f = 0.38$ (20% EtOAc/Hexanes) were obtained (84% combined yield) as thick colorless oils.

diethyl (E)-5-(2-methoxyvinyl)-2,2,7-trimethyl-2,3-dihydro-1H-indene-4,6-dicarboxylate (46).

^{1}H NMR (400 MHz, $CDCl_3$) 6.48 (d, J = 13.0 Hz, 1H), 5.66 (d, J = 13.1 Hz, 1H), 4.31 (q, J = 7.2, 2H), 4.30 (q, J = 7.2, 2H), 3.65 (s, 3H), 3.00 (s, 2H), 2.67 (s, 2H), 2.19 (s, 3H), 1.33 (t, J = 7.2 Hz, 3H), 1.33 (t, J = 7.2 Hz, 3H), 1.14 (s, 6H) ppm; **^{13}C{^{1}H}NMR** (100 MHz, $CDCl_3$) δ 170.1, 167.1, 151.3, 145.0, 143.2, 137.9, 134.0, 131.5, 123.0, 99.9, 61.2, 61.2, 56.3, 49.1, 46.9, 39.1, 29.2, 17.5, 14.3, 14.3 ppm; **HRMS** (ESI): $[M + Na]^+$ calculated for $C_{21}H_{28}O_5Na^+$: 383.1829; found: 383.1823.

diethyl (E)-6-(2-methoxyvinyl)-2,2,7-trimethyl-2,3-dihydro-1H-indene-4,5-dicarboxylate (45).

^{1}H NMR (400 MHz, $CDCl_3$) 6.51 (d, J = 13.0 Hz, 1H), 5.95 (d, J = 13.0 Hz, 1H), 4.33 (q, J = 7.2 Hz, 2H), 4.31 (q, J = 7.2 Hz, 2H), 3.61 (s, 3H), 2.81 (s, 2H), 2.65 (s, 2H), 2.18 (s, 3H), 1.34 (t, J = 7.2, 3H), 1.33 (t, J = 7.2, 3H), 1.14 (s, 6H) ppm; **^{13}C{^{1}H}NMR** (100 MHz, $CDCl_3$) δ 170.1, 168.8, 150.7, 143.5, 141.9, 133.5, 132.9, 130.3, 127.7, 101.0, 61.2, 60.9, 56.2, 47.9, 46.5, 39.4, 29.2, 16.9, 14.4, 14.4 ppm; **HRMS** (ESI): $[M + Na]^+$ calculated for $C_{21}H_{28}O_5Na^+$: 383.1829; found: 383.1823.

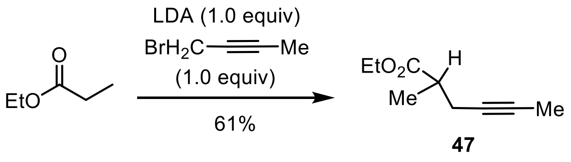

(±) ethyl 2-methylhex-4-ynoate (47). A solution of ethyl propionate (3.71 g, 36.0 mmol, 1.0 equiv) in 20 mL THF was slowly added over 15 minutes to a solution of LDA (38 mL, 1.0 M/THF, 1.05 equiv) in 72 mL THF at −78 °C. After 1 h at −78 °C, 1-bromo-2-butyne (4.98 g, 36 mmol, 1.0 equiv) was added dropwise and the solution was allowed to slowly warm up to room temperature overnight. The reaction was quenched by half saturated solution of NH_4Cl and the aqueous layer was extracted three times with diethyl ether. The combined organic layers were washed with brine, dried over $MgSO_4$, and concentrated by rotary evaporation. The crude volatile liquid was purified by flash column chromatography on silica gel (10% EtOAc/Hexanes) to afford 3.38 g **47** as a light-yellow oil (61% yield), $R_f = 0.58$ (20% EtOAc/Hexanes). Characterization data matched the previous report[4].

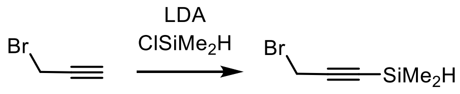

(3-bromoprop-1-yn-1-yl)dimethylsilane. To a solution of propargyl bromide (736 mg, 6.00 mmol, 1.0 equiv) in 12 mL of THF (0.5 M) at −78 °C was slowly added LiHMDS (6.0 mL, 1.0 M/THF, 6.00 mmol, 1.0 equiv) and stirred for 1 h at the same temperature. Dimethylchlorosilane (1.3 mL, 12.0 mmol, 2.0 equiv) was added and stirring was continued for 1 h and the reaction was complete based on TLC. The reaction mixture was quenched with 20 mL of half saturated NH_4Cl, and extracted with pentane (3×10 mL). The combined organics were washed with brine, dried over MgSO4, and filtered through a pad of silica gel. The filtrate was concentrated by rotary evaporation to an approximate volume of 3 mL and used for the next step without further purification, $R_f = 0.53$ (100% EtOAc/Hexanes).

[1]**H NMR** (400 MHz, $CDCl_3$) δ 4.14 (ts, *J* = 3.8, 1.1 Hz, 1H), 3.91 (d, *J* = 1.1 Hz, 2H), 0.25 (d, *J* = 3.8 Hz, 6H) ppm; **^{13}C{^{1}H}NMR** (100 MHz, $CDCl_3$) δ 101.4, 89.5, 14.5, -3.2 ppm.

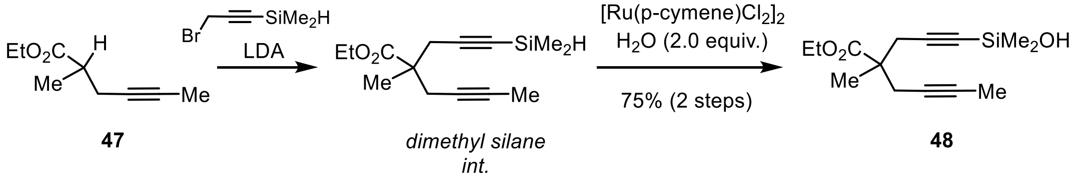

(±) ethyl 2-(3-(hydroxydimethylsilyl)prop-2-yn-1-yl)-2-methylhex-4-ynoate (48). A solution of alkyne **47** (427 mg, 3.00 mmol, 1.0 equiv) in 3.0 mL THF was slowly added over 15 minutes to a solution of LDA (3.3 mL, 1.0 M/THF, 1.1 equiv) in 20 mL THF at −84 °C (EtOAC/Liq. N_2). After 30 mins at −84 °C, the previously made solution of (3-bromoprop-1-yn-1-yl)dimethylsilane XX (6.00 mmol, 2.0 equiv) was added dropwise. The reaction was allowed to warm up slowly to room temperature overnight and quenched by a half saturated solution of NH_4Cl and the aqueous layer was extracted three times with diethyl ether. The combined organic layers were washed with brine, dried over $MgSO_4$, and concentrated by rotary evaporation. The resulting crude oil was diluted with acetonitrile (12 mL, 0.25 M) and water (0.11 mL, 6.00 mmol, 2.0 equiv) and $[RuCl_2(p\text{-cymene})]_2$ (37 mg, 2 mol%) were added respectively. The reaction mixture was stirred at room temperature and under open air for 1 hour until bubbling ceased. Acetonitrile was removed in vacuo and the crude residue was purified by flash column chromatography (5-40% EtOAc/Hexanes) to give **48** (600 mg, 75% over two steps) as a colorless oil, R_f = 0.27 (20% EtOAc/Hexanes).

[1]**H NMR** (400 MHz, $CDCl_3$) δ 4.17 (q, *J* = 7.1 Hz, 2H), 2.59 (s, 1H), 2.58 (s, 1H), 2.48 (q, *J* = 2.6 Hz, 1H), 2.47 (q, *J* = 2.6 Hz, 1H), 1.76 (t, *J* = 2.6 Hz, 2H), 1.30 (s, 3H), 1.26 (t, *J* = 7.1 Hz, 3H), 0.26 (s, 6H) ppm; **^{13}C{^{1}H}NMR** (100 MHz, $CDCl_3$) δ 175.1, 103.4, 86.4, 78.4, 74.9, 61.1, 45.8, 28.2, 27.6, 22.0, 14.3, 3.6, 1.7 ppm; **HRMS** (ESI): $[M + Na]^+$ calculated for $C_{14}H_{22}O_3SiNa^+$: 289.1230; found: 289.1221.

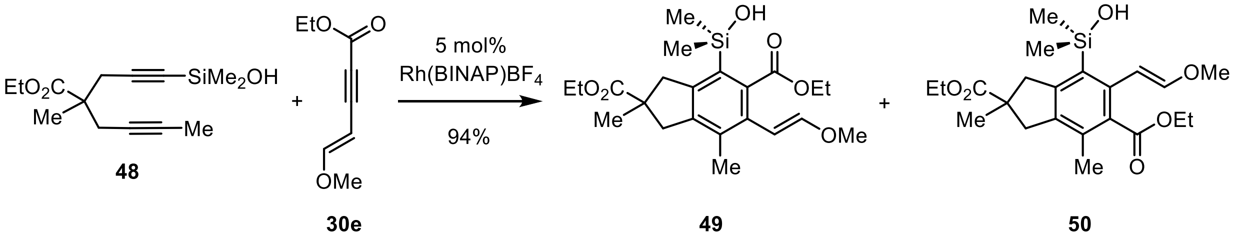

According to general procedure A, a solution of diyne **48** (100 mg, 0.375 mmol, 1.0 equiv) and alkyne **30e** (116 mg, 0.752 mmol, 2.0 equiv) in 3.0 mL DCM was added slowly over 1 h to the solution of 5 mol% active catalyst in 1.5 mL. After purification an inseparable mixture of compounds **49** and **50** (148 mg, 94% combined yield) which was estimated by ^{1}H NMR to be a 96:4.0 mixture of **49**:**50,** Rf = 0.34 (20% EtOAc/Hexanes)

(±) diethyl (E)-4-(hydroxydimethylsilyl)-6-(2-methoxyvinyl)-2,7-dimethyl-2,3-dihydro-1H-indene-2,5-dicarboxylate (49).

[1]**H NMR** (400 MHz, $CDCl_3$) 6.42 (d, *J* = 13.0 Hz, 1H), 5.69 (d, *J* = 13.0 Hz, 1H), 4.27 (q, *J* = 7.2 Hz, 2H), 4.16 (q, *J* = 7.1 Hz, 2H), 3.65 (s, 3H), 3.53 (d, *J* = 16.0 Hz, 1H), 3.37 (d, *J* = 16.2 Hz, 1H), 2.91 (d, *J* = 16.0 Hz, 1H), 2.75 (d, *J* = 16.2 Hz, 1H), 2.33 (s, 1H), 2.19 (s, 3H), 1.35 (s, 3H), 1.33 (t, *J* = 7.2 Hz, 3H), 1.26 (t, *J* = 7.1 Hz, 3H), 0.40 (s, 6H) ppm; **^{13}C{^{1}H}NMR** (100 MHz, $CDCl_3$) δ 177.6, 173.1, 150.8, 144.7, 141.7, 138.9, 135.2, 130.7, 129.1, 100.7, 61.4, 61.0, 56.4, 49.2, 46.1, 42.7, 25.3, 17.1, 14.3, 14.1, 1.9, 1.8 ppm; **HRMS** (ESI): $[M + Na]^+$ calculated for $C_{22}H_{32}O_6SiNa^+$: 443.1860; found: 443.1846.

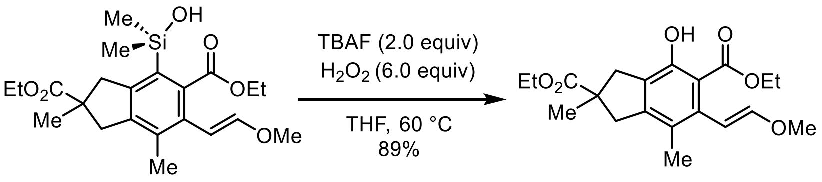

49 **51**

(±) diethyl (E)-4-hydroxy-6-(2-methoxyvinyl)-2,7-dimethyl-2,3-dihydro-1H-indene-2,5-dicarboxylate (51). To a stirred solution of silanol **49** (220 mg, 0.523 mmol, 1.0 equiv) in 2.6 mL THF (0.2 M) was added TBAF (1.0 mL, 1.0 M/THF, 1.05 mmol, 2.0 equiv) and 50% H_2O_2 (213 mg, 3.14 mmol, 6.0 equiv). The reaction mixture was stirred at 60 °C in a heating block for 24 h. The solution was cooled down to room temp and diluted with 5 mL of ether followed by adding 10 mL half saturated NH_4Cl solution. The phases were separated, and the aqueous phase was extracted with Et_2O (3×10 mL) and the combined organic extracts were dried over MgSO4 and concentrated in vacuo. The crude residue was purified by flash column chromatography (3-10% EtOAc/Hexanes) to give **51** (169 mg, 89%) as a colorless thick oil.

^{1}H NMR (400 MHz, $CDCl_3$) δ 10.60 (s, 1H), 6.18 (d, *J* = 13.1 Hz, 1H), 5.92 (d, *J* = 13.1 Hz, 2H), 4.35 (q, *J* = 7.2 Hz, 2H), 4.17 (q, *J* = 7.2 Hz, 2H), 3.69 (s, 3H), 3.44 (d, *J* = 16.8 Hz, 1H), 3.41 (d, *J* = 16.6 Hz, 1H), 2.90 (d, *J* = 16.6 Hz, 1H), 2.77 (d, *J* = 16.8 Hz, 1H), 2.13 (s, 3H), 1.37 (s, 3H), 1.36 (t, *J* = 7.2 Hz, 3H), 1.27 (t, *J* = 7.2 Hz, 3H) ppm; **^{13}C{^{1}H}NMR** (100 MHz, $CDCl_3$) δ 177.6, 172.0, 155.7, 149.1, 148.2, 135.7, 126.6, 124.9, 112.3, 103.5, 61.4, 61.0, 56.1, 48.8, 44.6, 41.1, 26.0, 17.1, 14.3, 14.3 ppm; **HRMS** (ESI): $[M + Na]^+$ calculated for $C_{20}H_{26}O_6Na^+$: 385.1622; found: 385.1610.

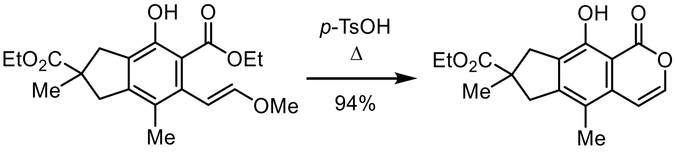

51 **52**

(±) thyl 9-hydroxy-5,7-dimethyl-1-oxo-1,6,7,8-tetrahydrocyclopenta[g]isochromene-7-carboxylate (52). To a solution of **51** (34.0 mg, 0.094 mmol, 1.0 equiv) in 2.0 mL toluene (0.04 M) was added *p*-TsOH.H_2O (36.0 mg, 0.188 mmol, 2.0 equiv) and the reaction was refluxed in a heating block for 20 h. The solution was cooled down to rt and diluted with DCM and transferred to an evaporating flask. The volatiles were removed in vacuo and the crude residue was loaded onto silica gel and chromatographed using a 5-20% EtOAc/Hexanes eluent to afford **52** as a white solid (27.0 mg, 94%), R_f = 0.34 (20% EtOAc/Hexanes).

^{1}H NMR (400 MHz, $CDCl_3$) 11.09 (s, 1H), 7.20 (d, *J* = 5.8 Hz, 1H), 6.63 (d, *J* = 5.8 Hz, 1H), 4.19 (q, *J* = 7.1 Hz, 2H), 3.54 (d, *J* = 17.0 Hz, 1H), 3.46 (d, *J* = 16.8 Hz, 1H), 2.98 (d, *J* = 16.8 Hz, 1H), 2.88 (d, *J* = 17.0 Hz, 1H), 2.25 (s, 3H), 1.39 (s, 3H), 1.28 (t, *J* = 7.1 Hz, 3H) ppm; **^{13}C{^{1}H}NMR** (100 MHz, $CDCl_3$) δ 177.2, 167.0, 156.0, 152.1, 142.9, 133.9, 127.2, 119.3, 106.3, 105.5, 61.1, 49.1, 44.4, 40.8, 25.7, 14.4, 14.3 ppm; **HRMS** (ESI): $[M + Na]^+$ calculated for $C_{17}H_{18}O_5Na^+$: 325.1046; found: 325.1044.

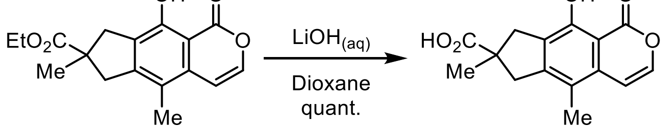

52 **6,** ± fomajorin S

(±) fomajorin S (6). In a 2-dram vial containing 22.0 mg of fomajorin S ethyl ester **52** (0.073 mmol, 1.0 equiv) was added 0.6 mL 1,4-dioxane and 0.6 mL 1.0 M NaOH at room temperature. The resulting homogenous solution was stirred at room temperature for 1 h until the starting material was fully hydrolyzed based on TLC. Water (3.0 mL) and

ethyl acetate (3.0 mL) was added to the vial. The organic layer was separated, and the water layer was acidified by dropwise addition of 1 M HCl until pH=5-6 at which point the solution turned cloudy. The aqueous layer was extracted with EtOAc (3×2 mL) and the combined organic layers were washed with brine, dried over Na_2SO_4, and filtered through a fritted glass followed by rotary evaporation to remove solvent. The white solid residue was determined to be pure by ^{1}H NMR (20.0 mg, quant), R_f = 0.2 (50% EtOAc/Hexanes).

^{1}H NMR (400 MHz, DMSO-d6) δ 10.99 (s, 1H), 7.52 (d, *J* = 5.8 Hz, 1H), 6.85 (d, *J* = 5.8 Hz, 1H), 3.39 (d, *J* = 35.8 Hz, 1H), 3.36 (d, *J* = 35.8 Hz, 1H), 2.85 (d, *J* = 33.7 Hz, 1H), 2.81 (d, *J* = 33.1 Hz, 1H), 2.21 (s, 3H), 1.31 (s, 3H) ppm; **^{13}C{^{1}H}NMR** (100 MHz, DMSO-d6) δ 178.1, 166.3, 154.6, 152.4, 143.9, 133.6, 126.6, 119.4, 105.3, 105.3, 48.3, 43.8, 40.2, 25.1, 13.9. ppm; **HRMS** (ESI): $[M + H]^+$ calculated for $C_{15}H_{15}O_5^+$: 275.0914; found: 275.0912.

References:

[1] Tavakoli, A.; Dudley, G. B. Synthesis of 4,4-dimethyl-1,6-heptadiyne and other neo-pentylene-tethered (NPT) 1,6-diynes. *J. Org. Chem.*, 2022, **87**, 5773–5784.

[2] Maity, P.; Lepore, S. D. Selective One-Pot Synthesis of Allenyl and Alkynyl Esters from β-Ketoesters. *J. Org. Chem.* **2009**, *74*, 158–162.

[3] Lagoutte, R.; Serba, C.; Abegg, D.; Hoch, D. G.; Adibekian, A.; Winssinger, N. Divergent synthesis and identification of the cellular targets of deoxyelephantopins. *Nat. Commun.* **2016**, *7*, 12470.

[4] Kramp, G. J.; Kim, M.; Gais, H-J; Vermeeren, C. Fully Stereocontrolled Total Syntheses of the Prostacyclin Analogues 16*S*-Iloprost and 16*S*-3-Oxa-Iloprost by a Common Route, Using Alkenylcopper-Azoalkene Conjugate Addition, Asymmetric Olefination, and Allylic Alkylation *J. Am. Chem. Soc.* **2005**, *127*, 17910.

^{1}H NMR - 400 MHz, Chloroform-d

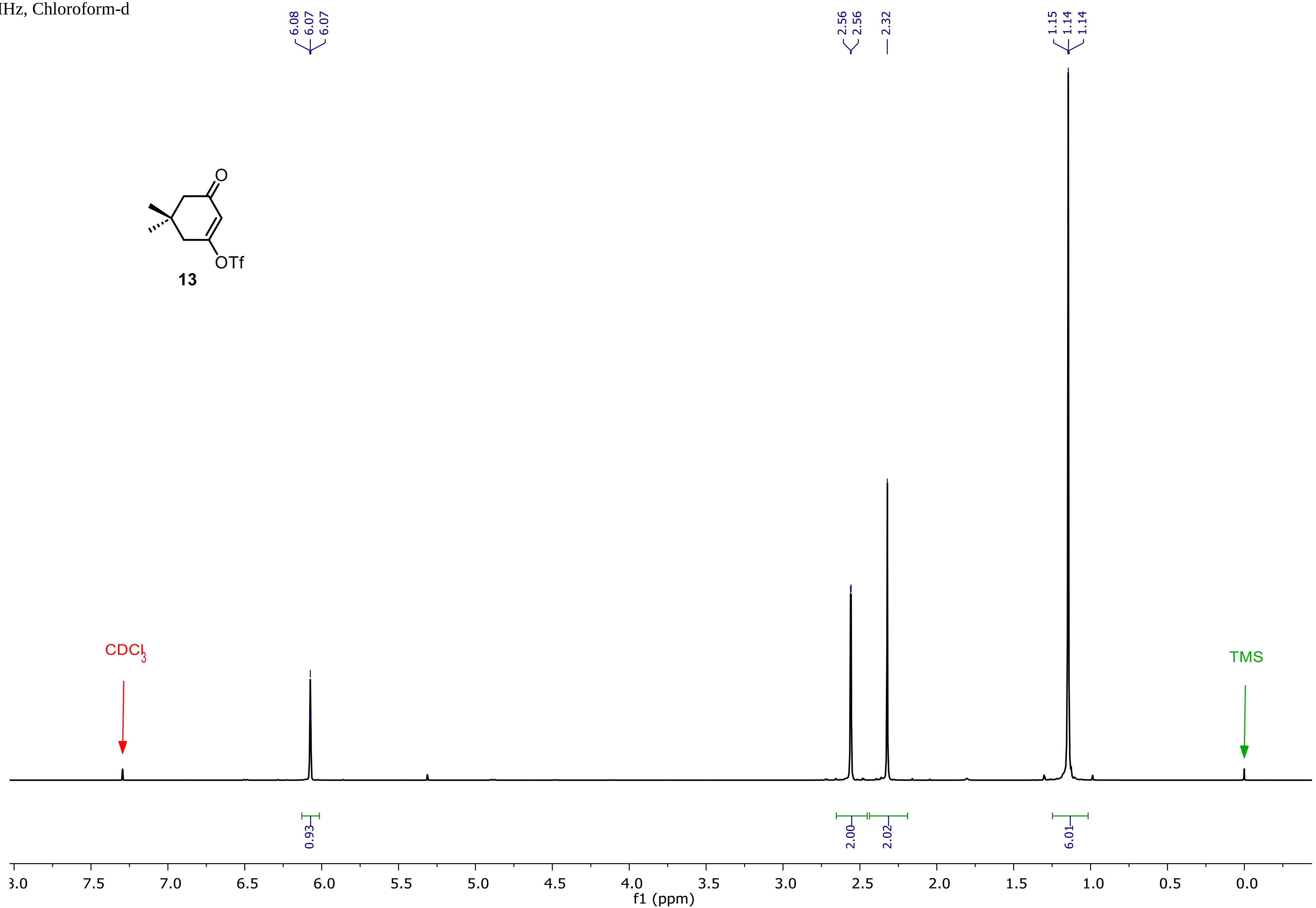

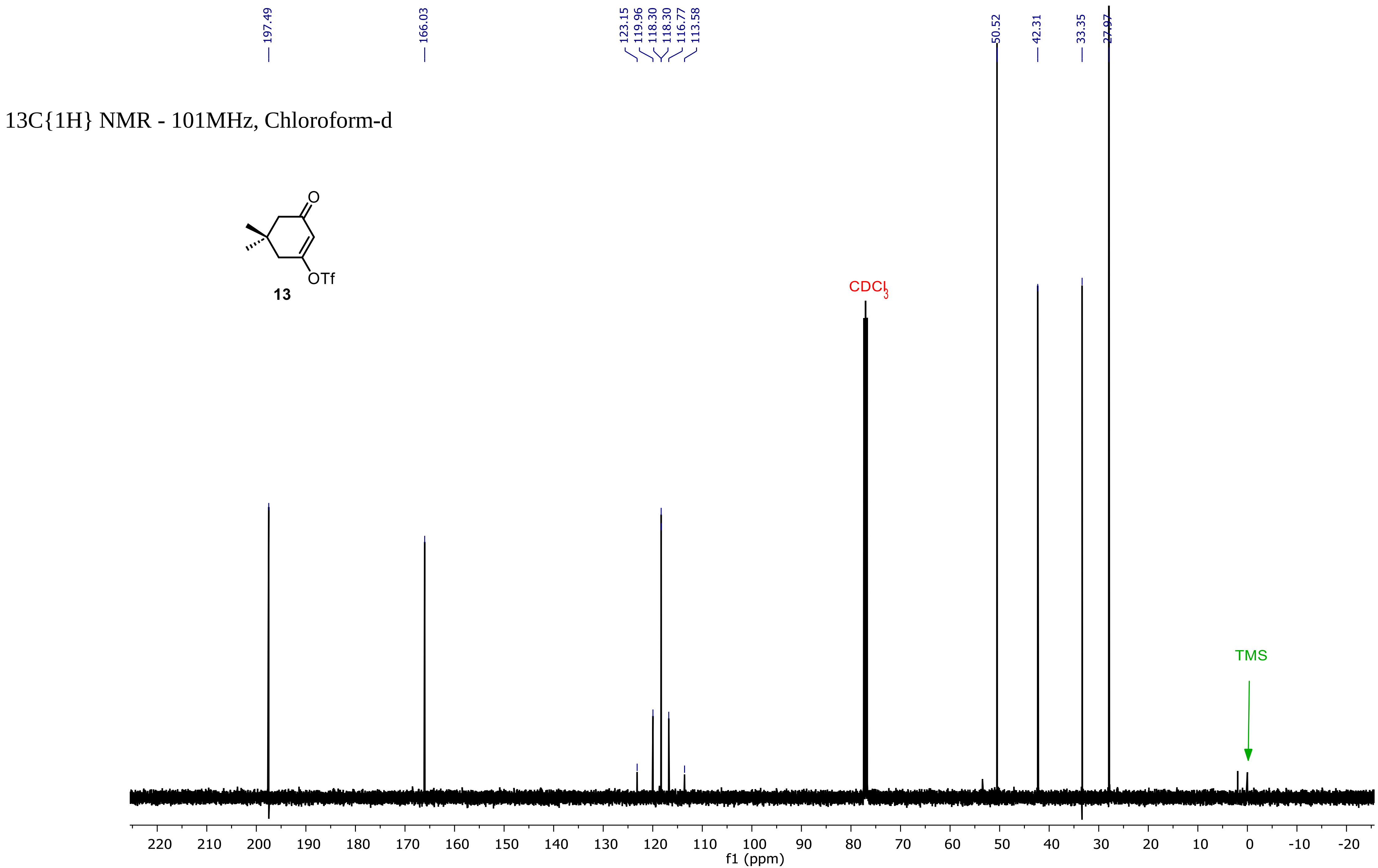

13C{1H} NMR - 101MHz, Chloroform-d
197.49
166.03
123.15
119.96
118.30
118.30
116.77
113.58
50.52
42.31
33.35
27.97
O
OTf
13
CDCl3
TMS
220 210 200 190 180 170 160 150 140 130 120 110 100 90 80 70 60 50 40 30 20 10 0 -10 -20
f1 (ppm)

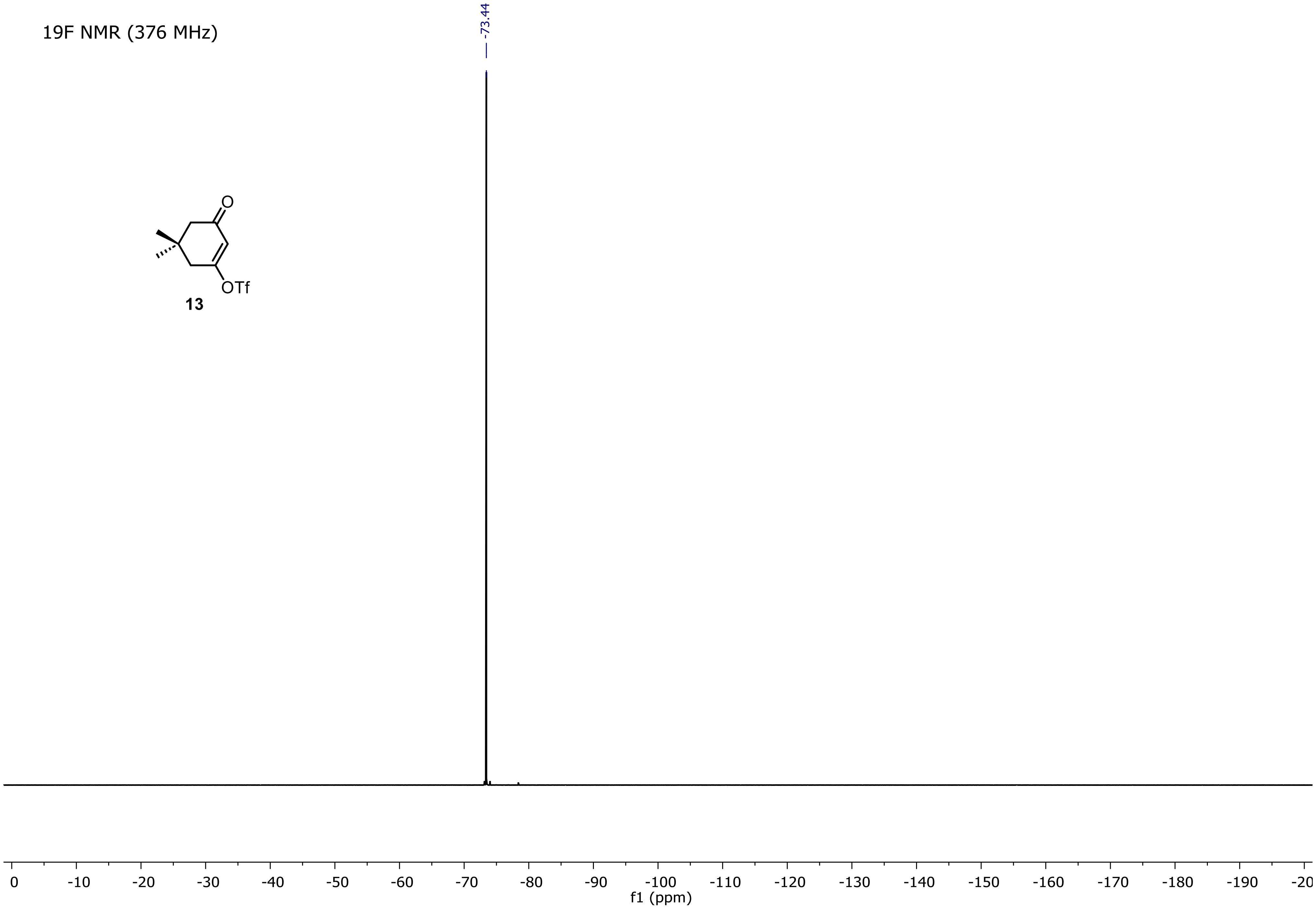
19F NMR (376 MHz)
-73.44
O
OTf
13
0
-10
-20
-30
-40
-50
-60
-70
-80
-90
-100
-110
-120
-130
-140
-150
-160
-170
-180
-190
-20
f1 (ppm)

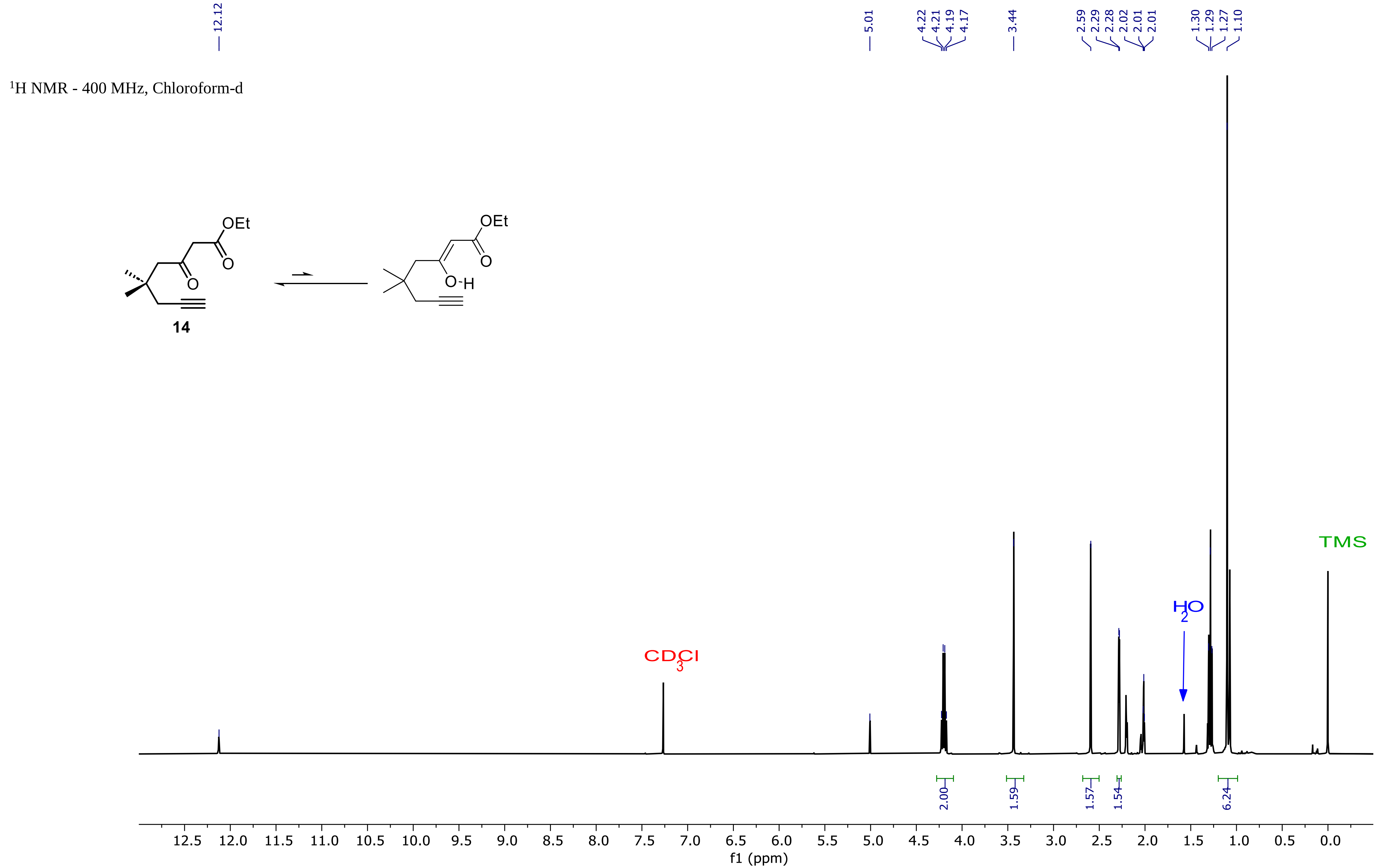

^{1}H NMR - 400 MHz, Chloroform-d
12.12
5.01
4.22
4.21
4.19
4.17
3.44
2.59
2.29
2.28
2.02
2.01
2.01
1.30
1.29
1.27
1.10
OEt
O
O
14
OEt
O
O-H
CDCl3
H2O
TMS
2.00
1.59
1.57
1.54
6.24
12.5 12.0 11.5 11.0 10.5 10.0 9.5 9.0 8.5 8.0 7.5 7.0 6.5 6.0 5.5 5.0 4.5 4.0 3.5 3.0 2.5 2.0 1.5 1.0 0.5 0.0
f1 (ppm)

13C{1H} NMR - 101MHz, Chloroform-d

The extra minor signals are related to enol tautomer.

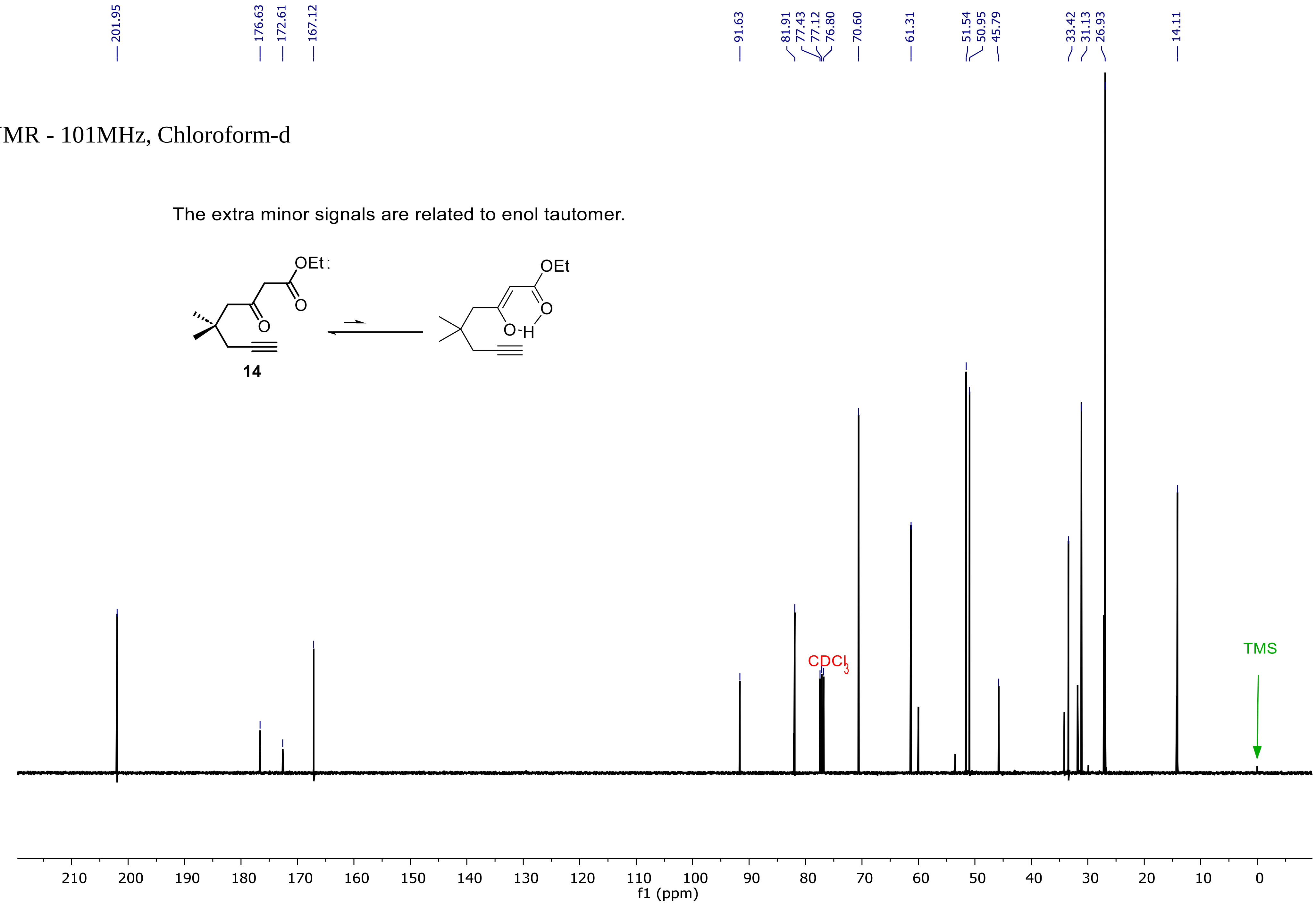

1H NMR - 400 MHz, Chloroform-d

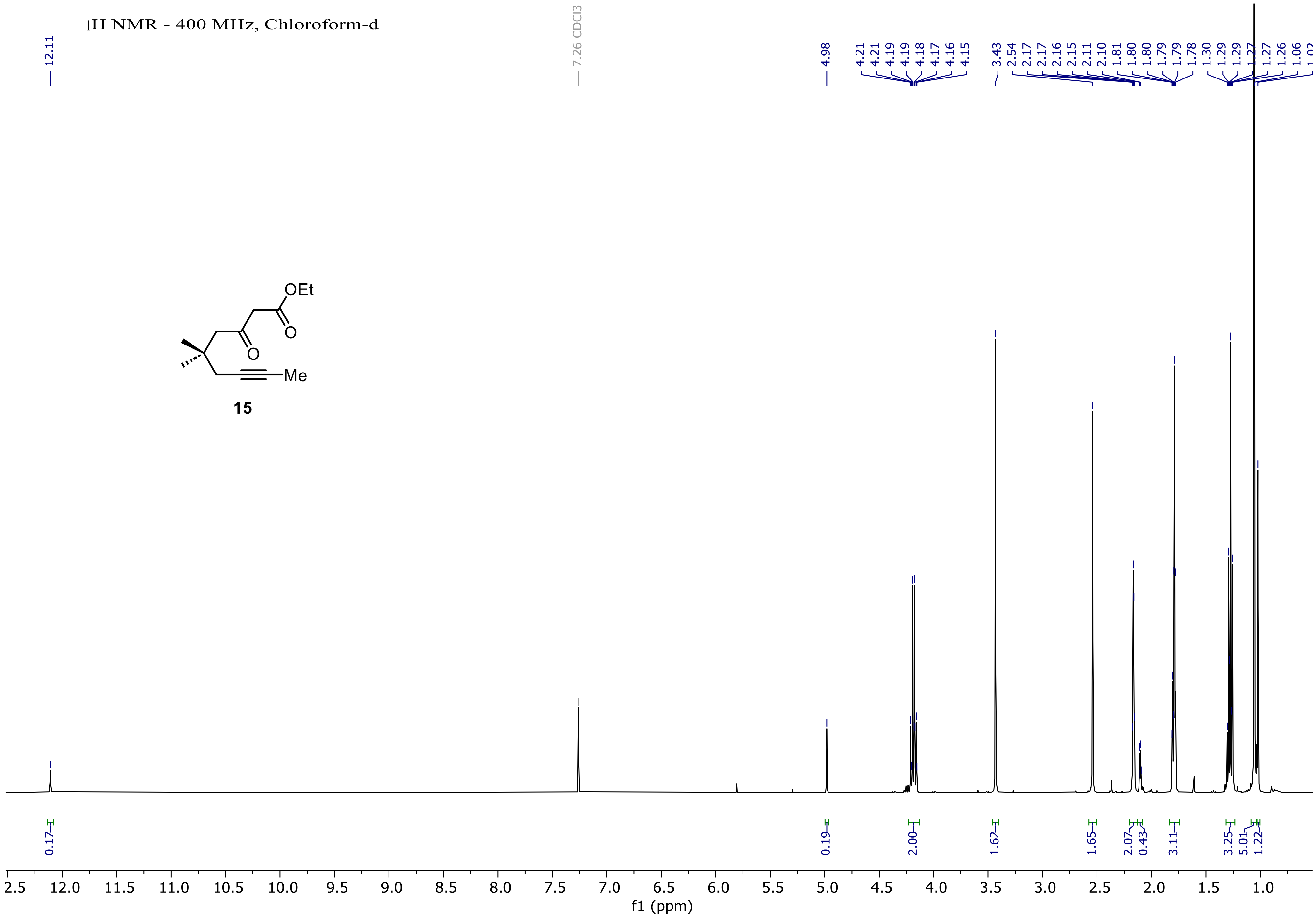

13C{1H} NMR - 101MHz, Chloroform-d

The extra minor signals are related to enol tautomer.

15

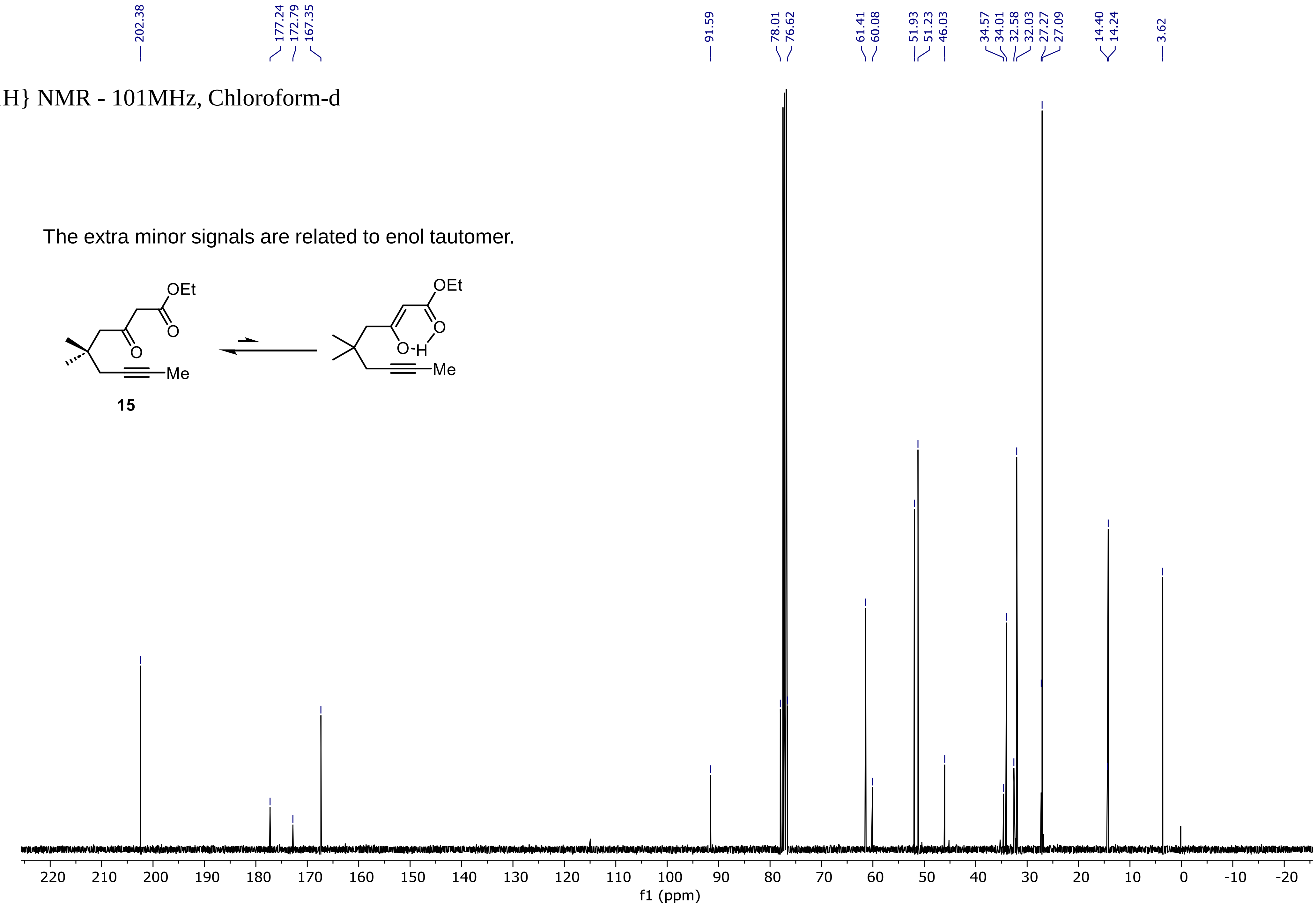

^{1}H NMR - 400 MHz, Chloroform-d

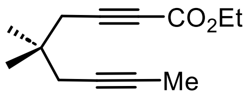

16

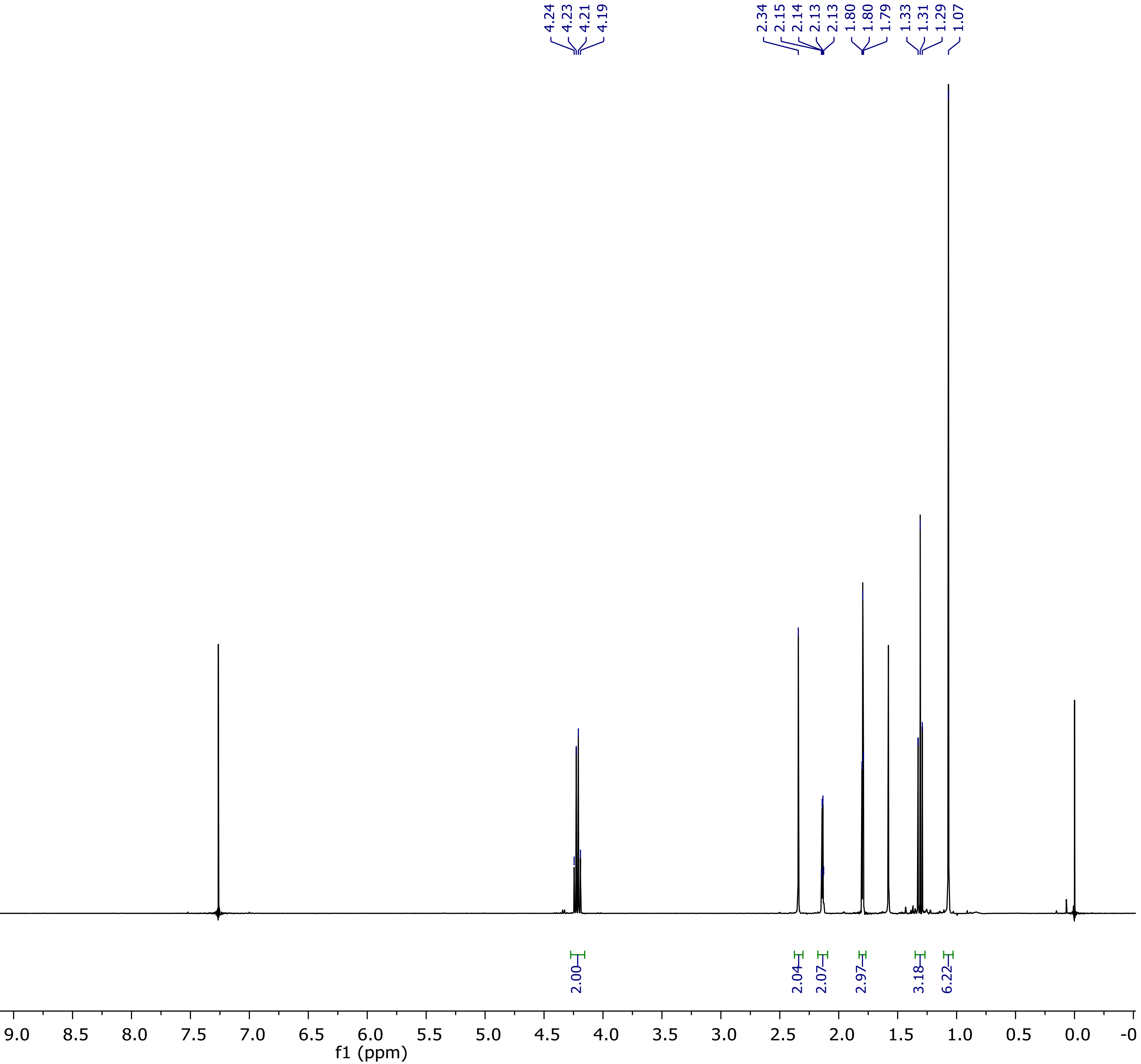

13C{1H} NMR - 101MHz, Chloroform-d

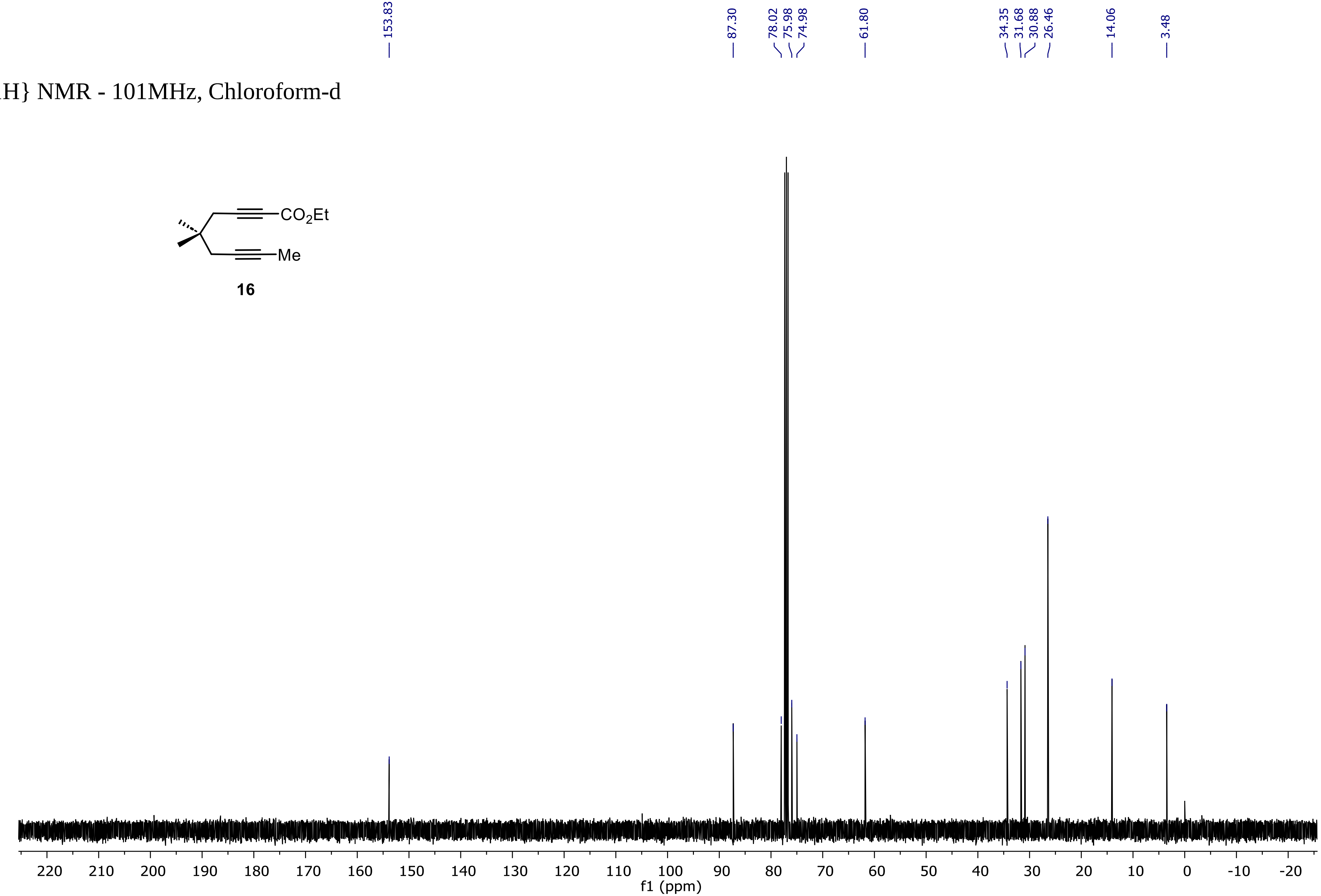

1H NMR - 400 MHz, Chloroform-d

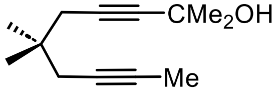

17

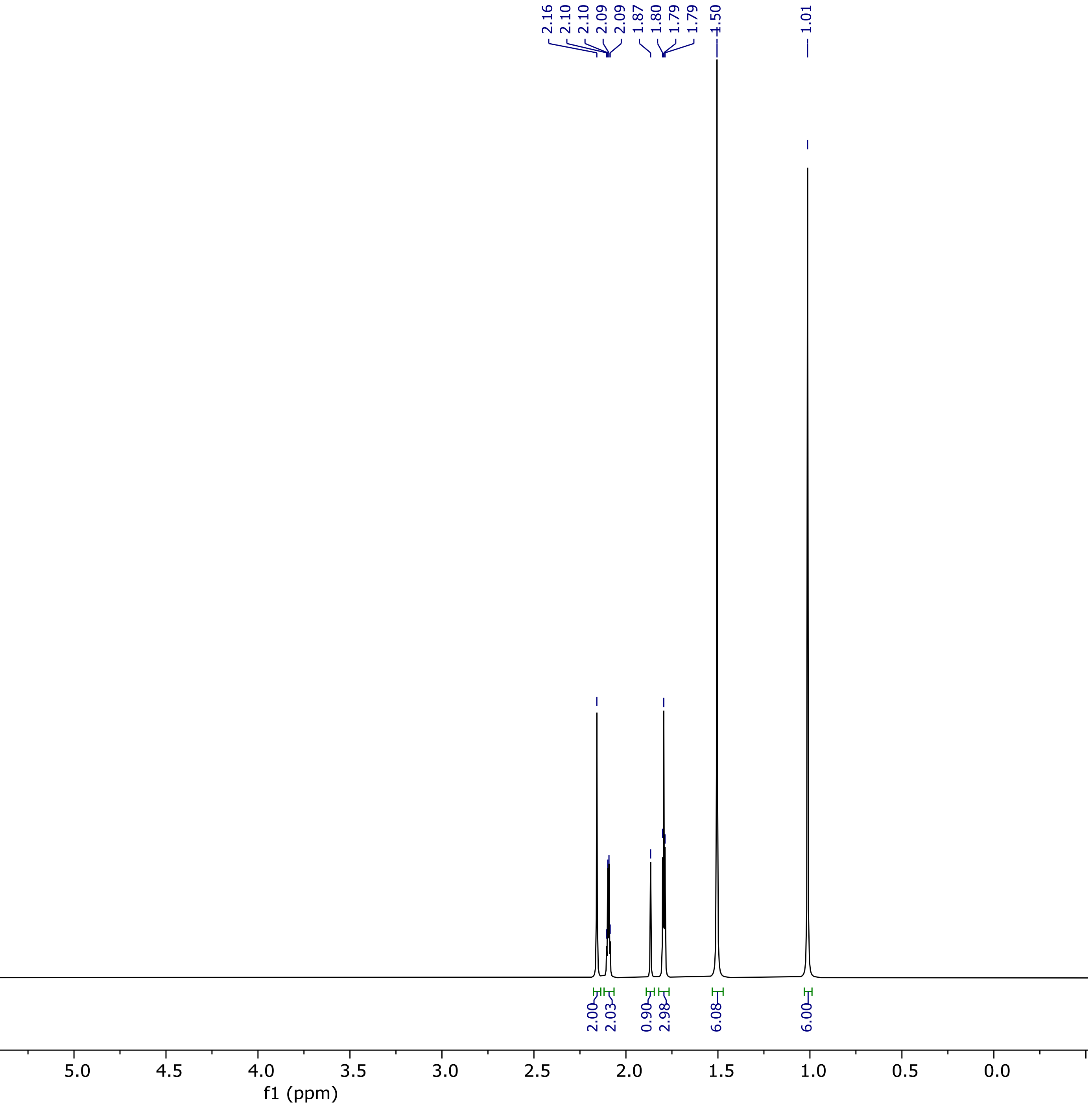

13C{1H} NMR - 101MHz, Chloroform-d

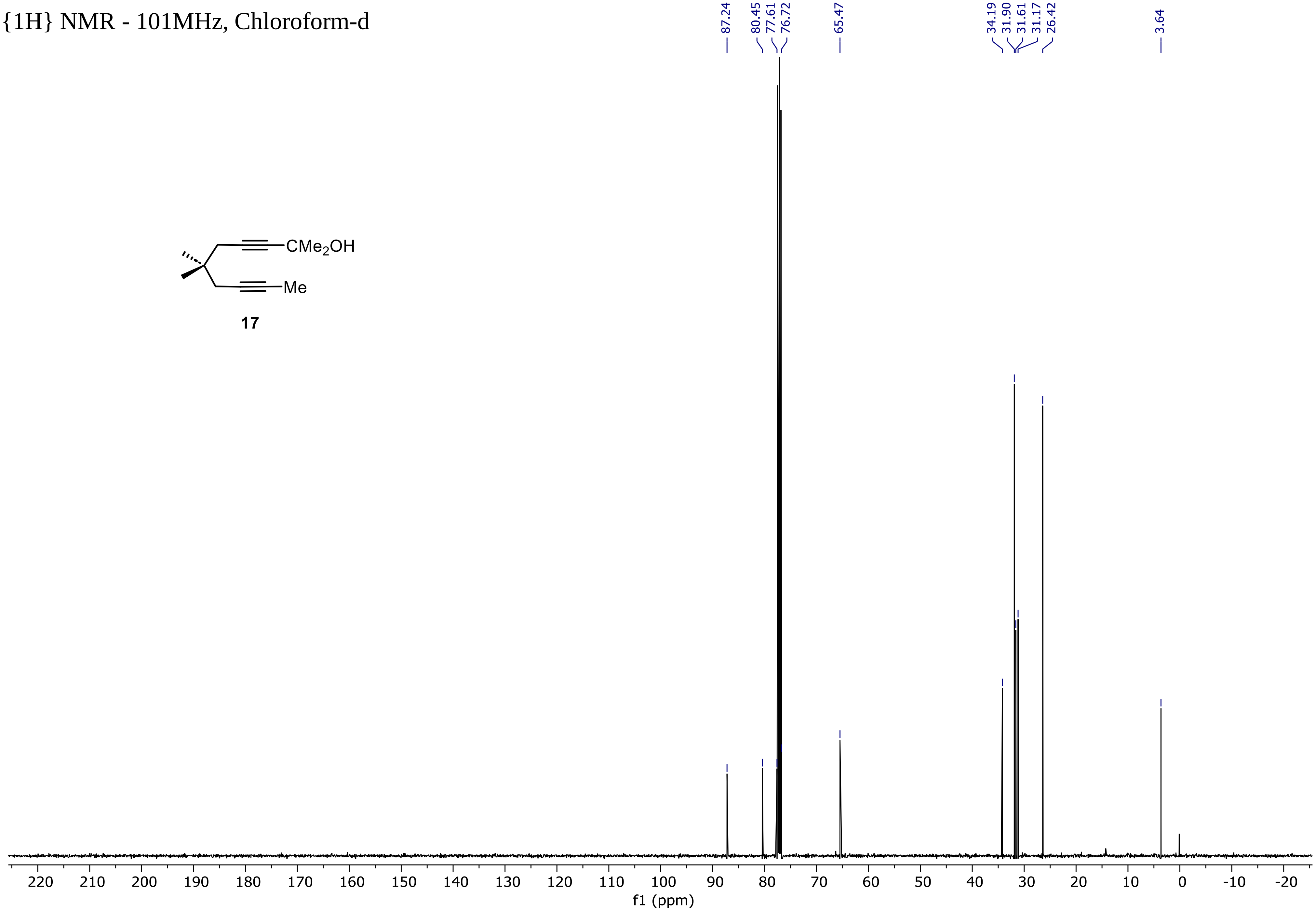

17

1H NMR - 400 MHz, Chloroform-d

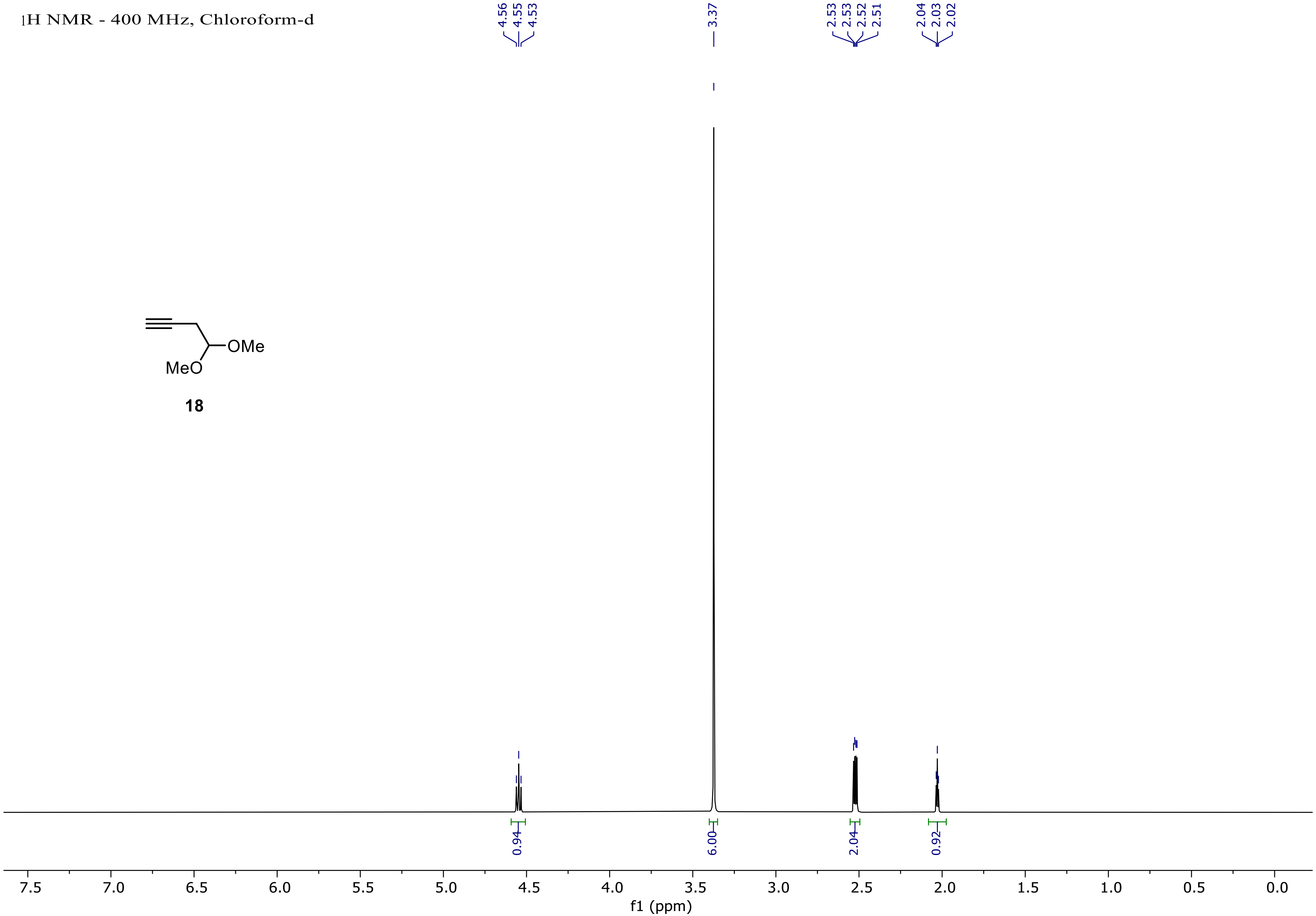

13C{1H} NMR - 101MHz, Chloroform-d

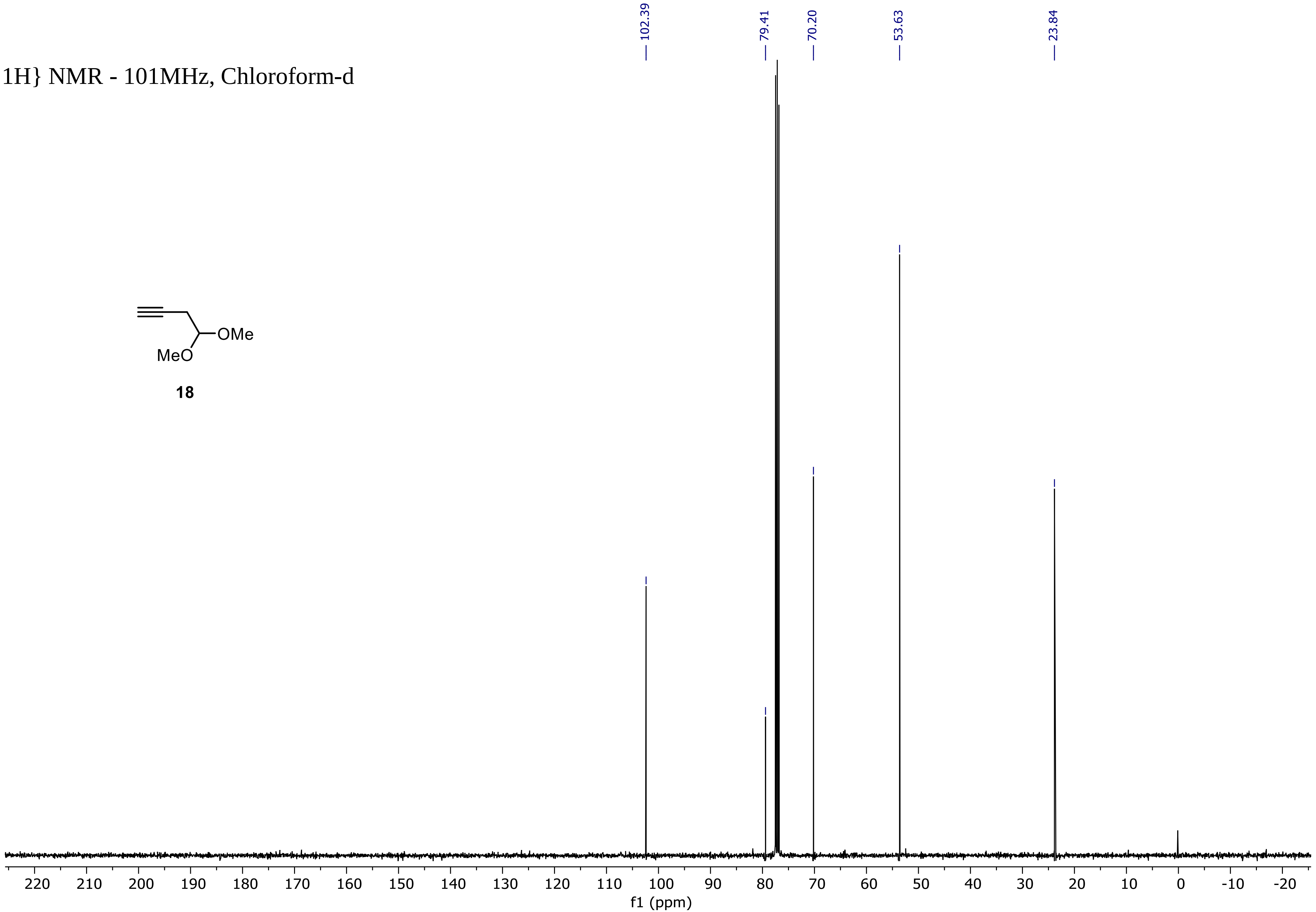

1H NMR - 400 MHz, Chloroform-d

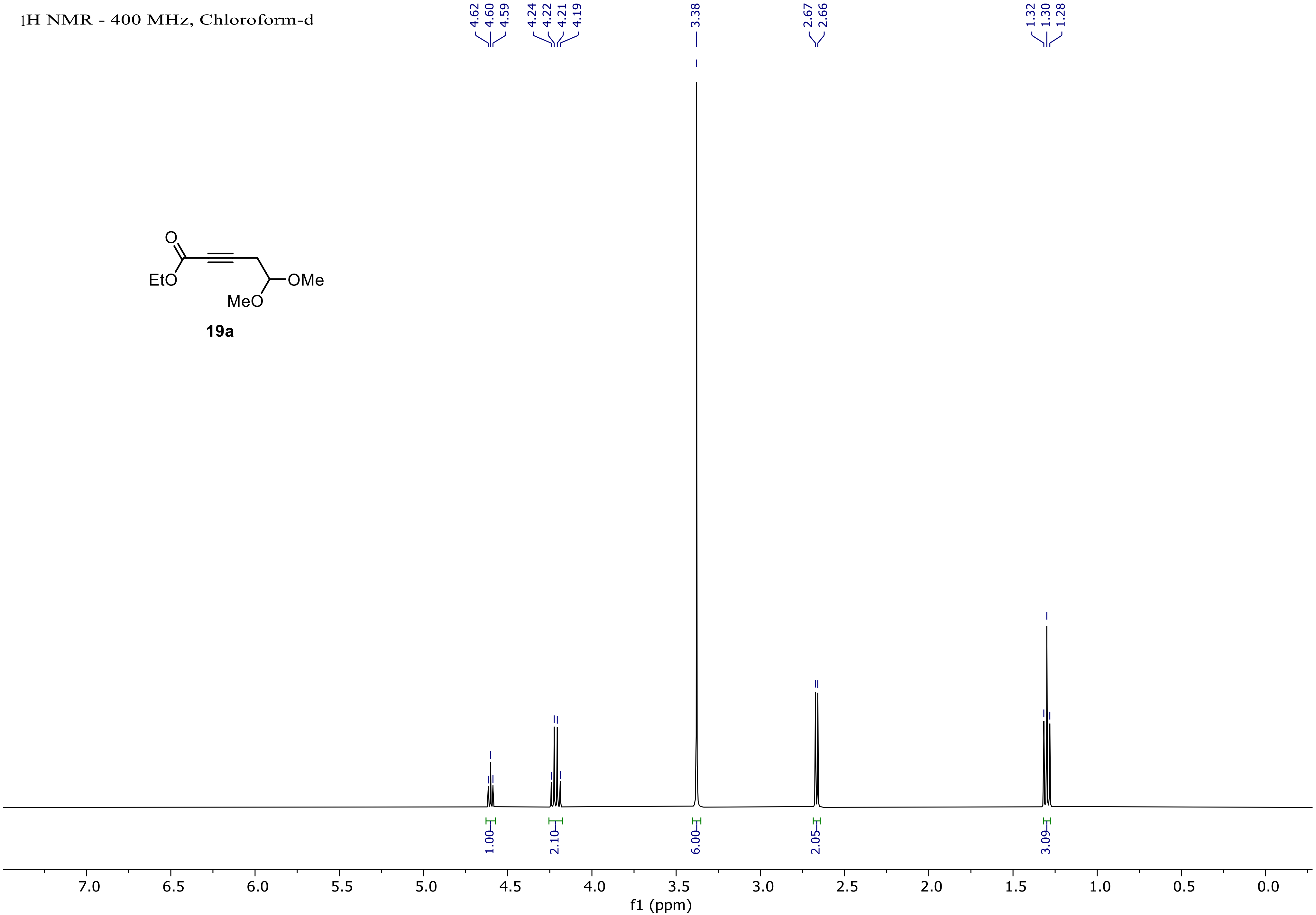

19a

13C{1H} NMR - 101MHz, Chloroform-d

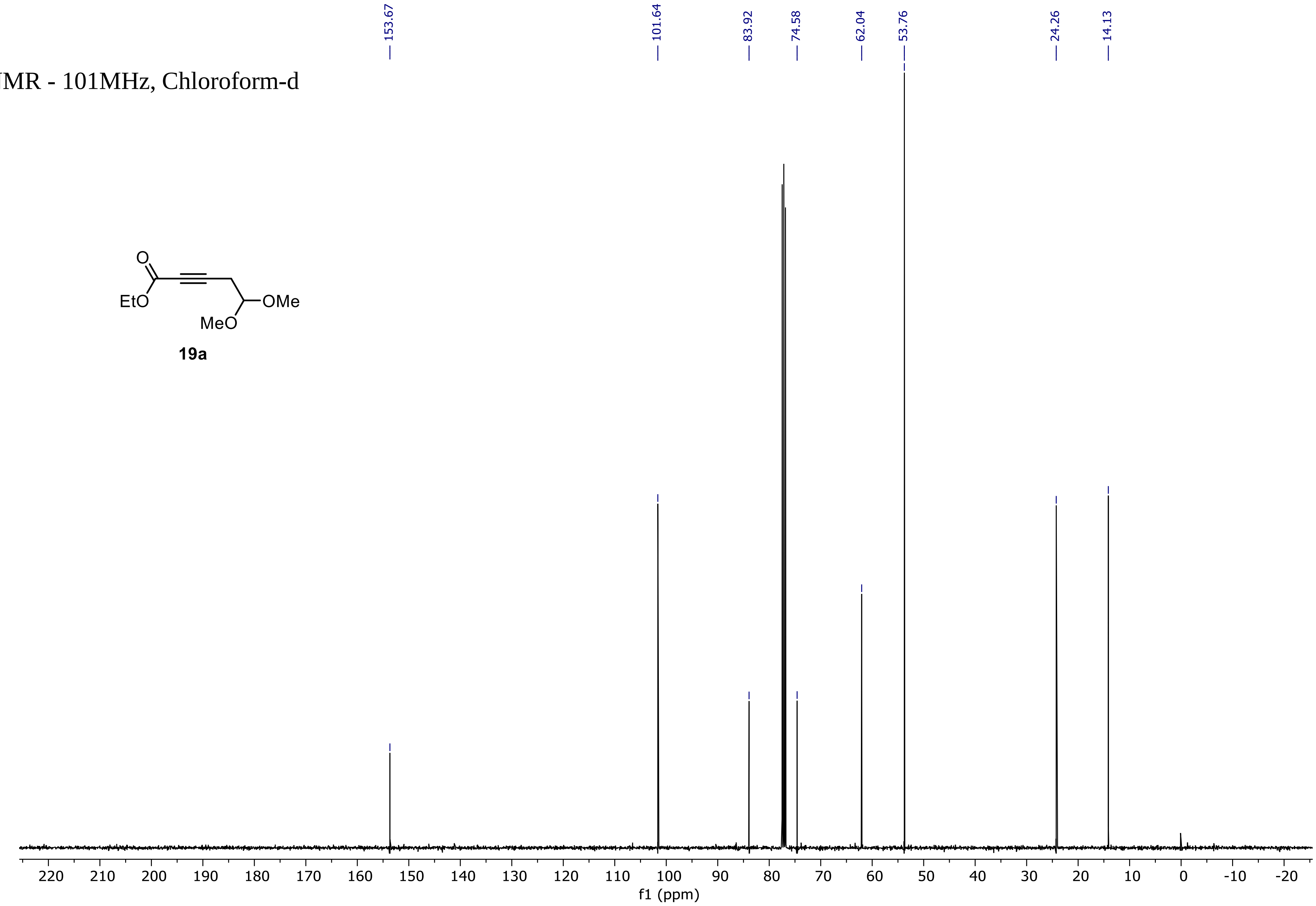

19a

1H NMR - 400 MHz, Chloroform-d

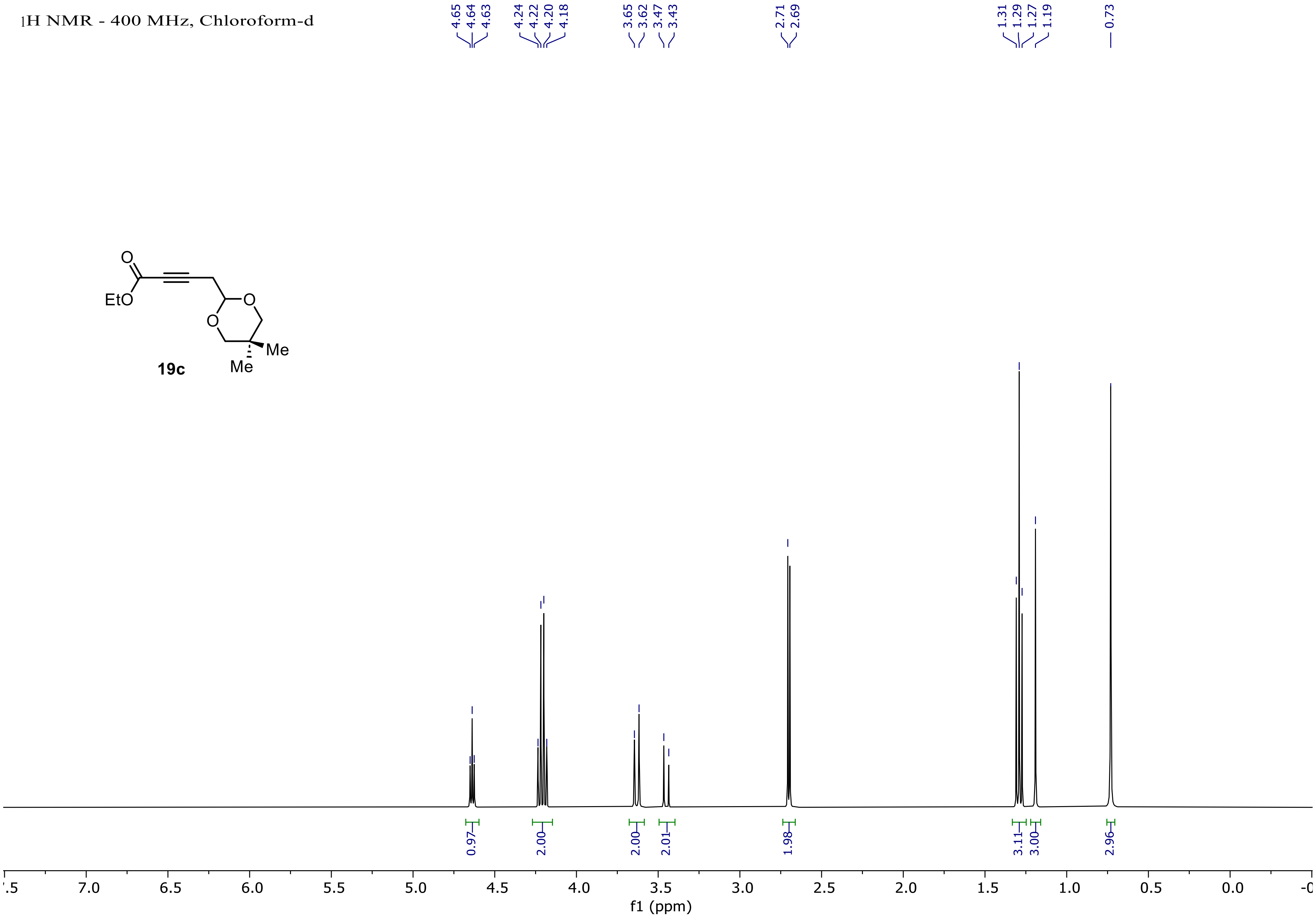

19c

13C{1H} NMR - 101MHz, Chloroform-d

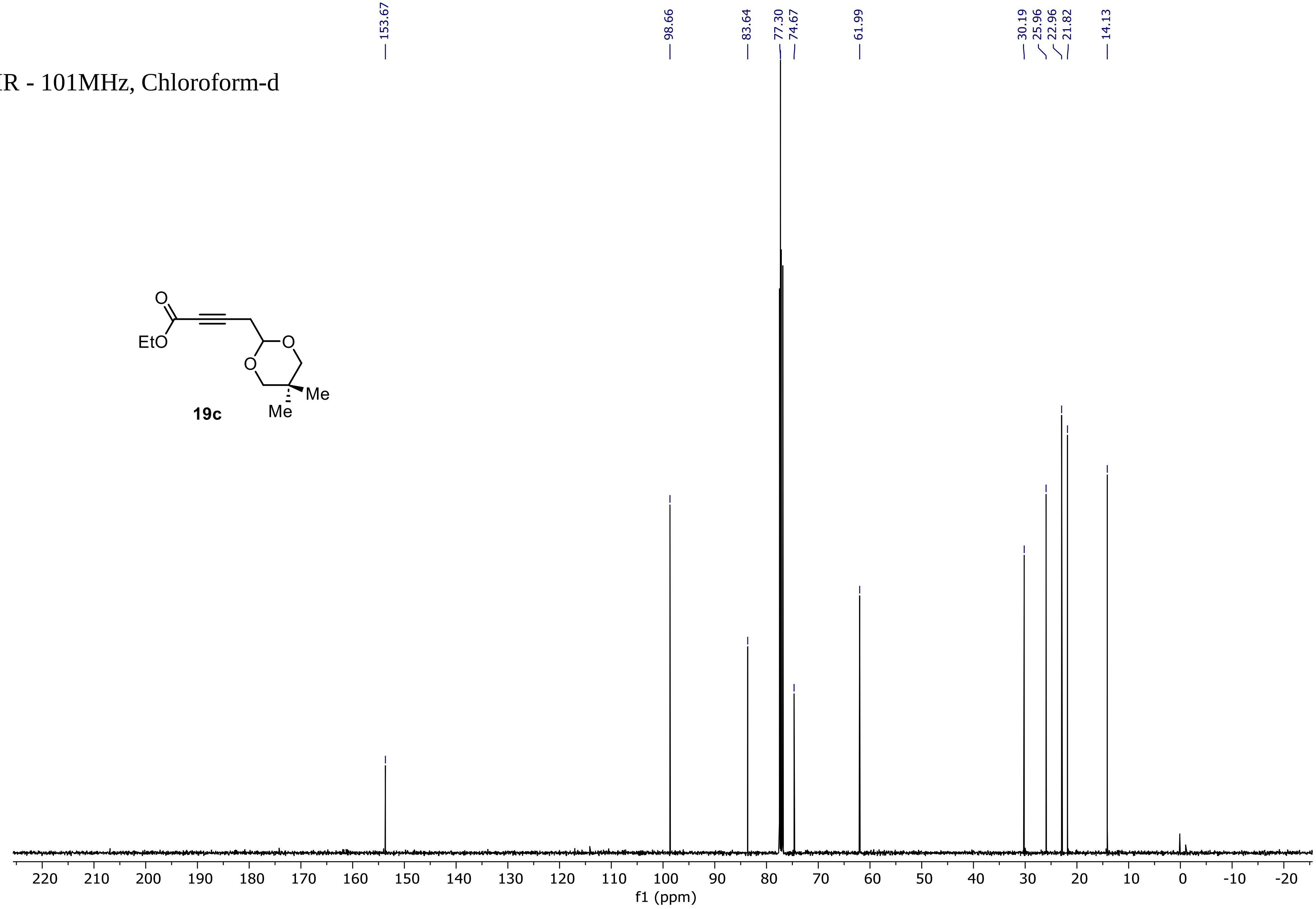

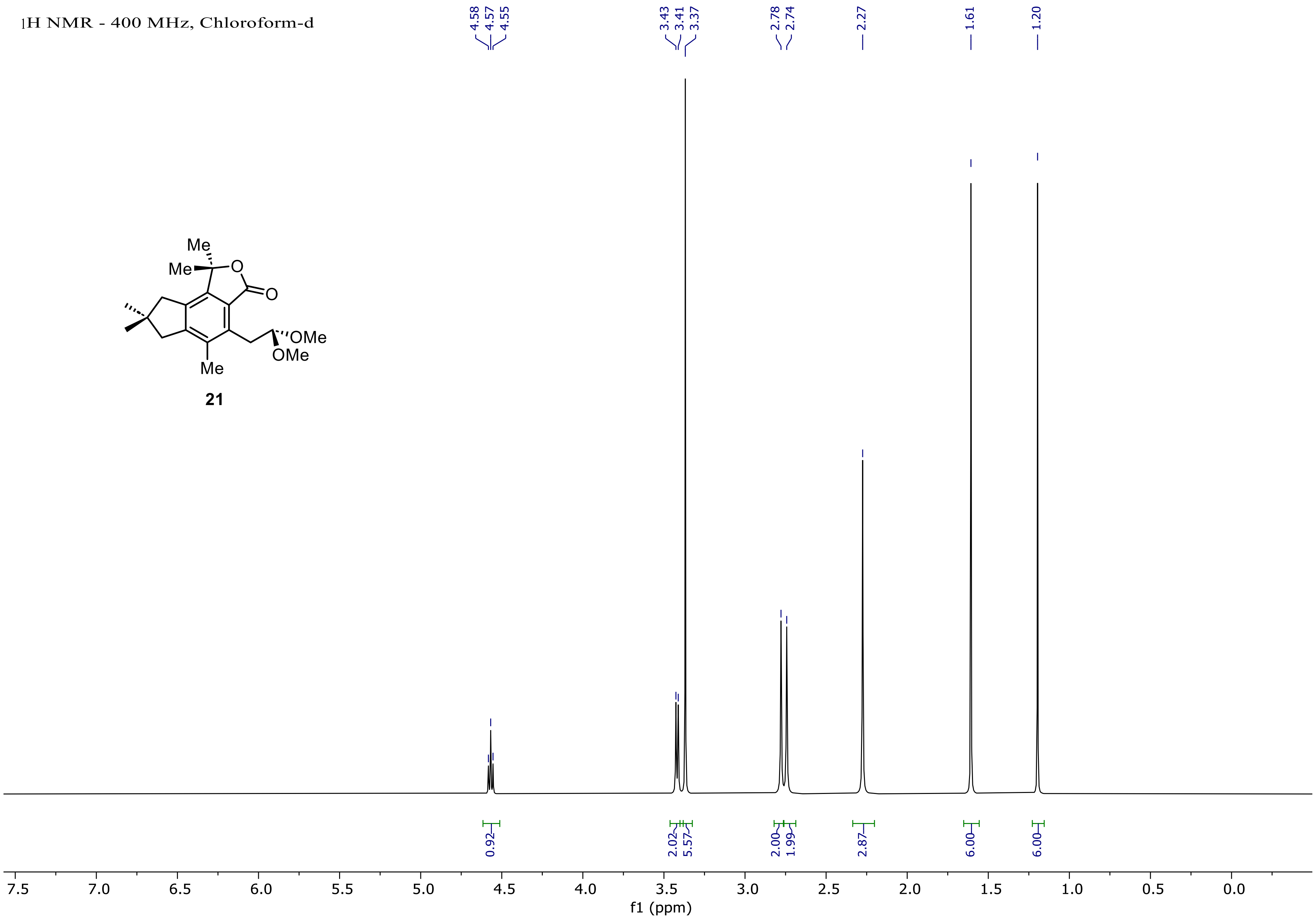
1H NMR - 400 MHz, Chloroform-d
4.58
4.57
4.55
3.43
3.41
3.37
2.78
2.74
2.27
1.61
1.20
Me
Me
O
O
OMe
OMe
Me
21
0.92
2.02
5.57
2.00
1.99
2.87
6.00
6.00
7.5
7.0
6.5
6.0
5.5
5.0
4.5
4.0
3.5
3.0
2.5
2.0
1.5
1.0
0.5
0.0
f1 (ppm)

13C{1H} NMR - 101MHz, Chloroform-d

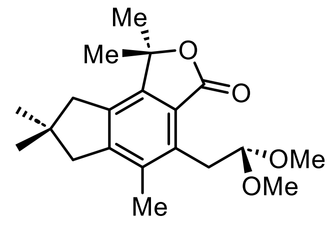

21

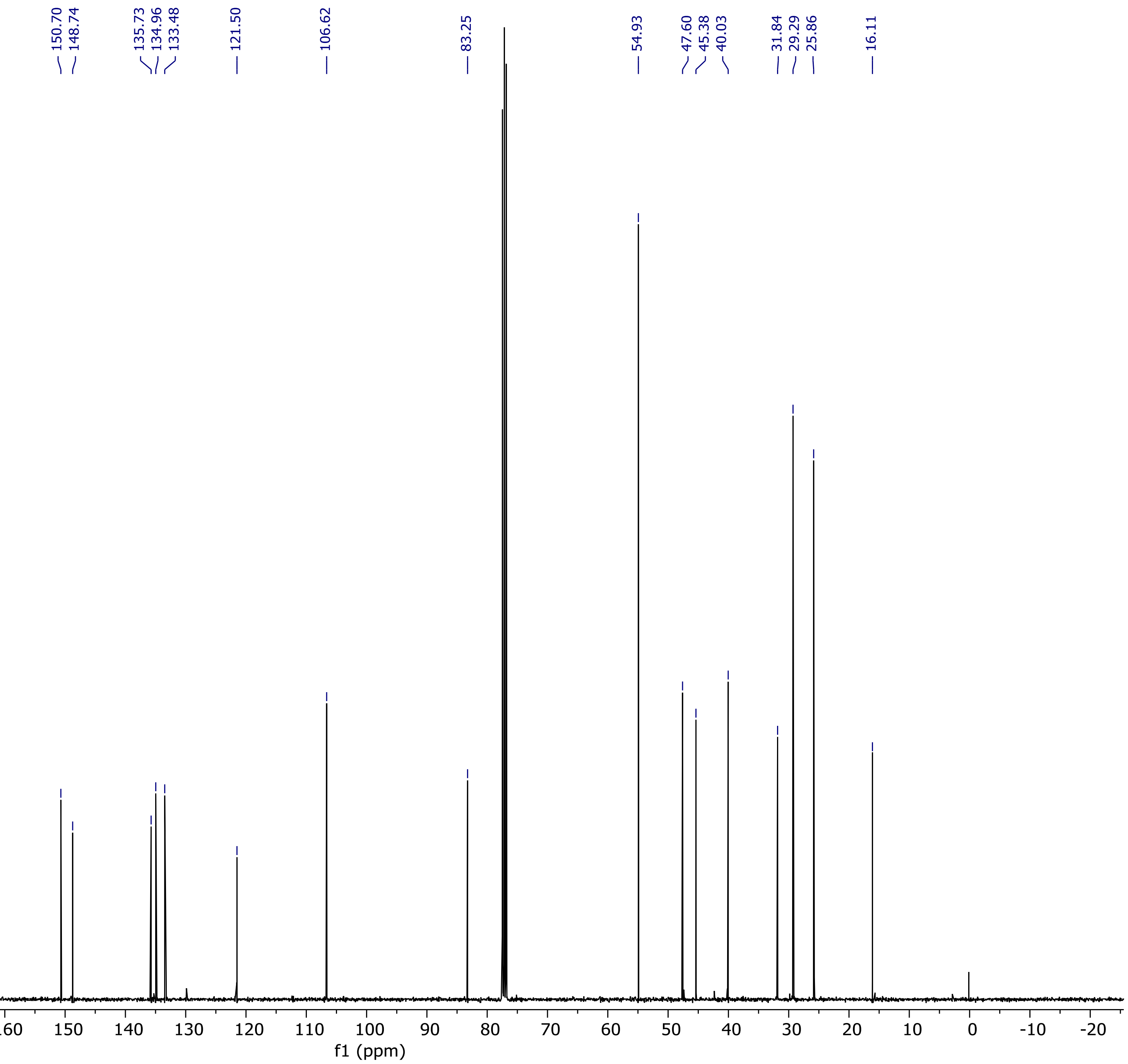

1D NOESY

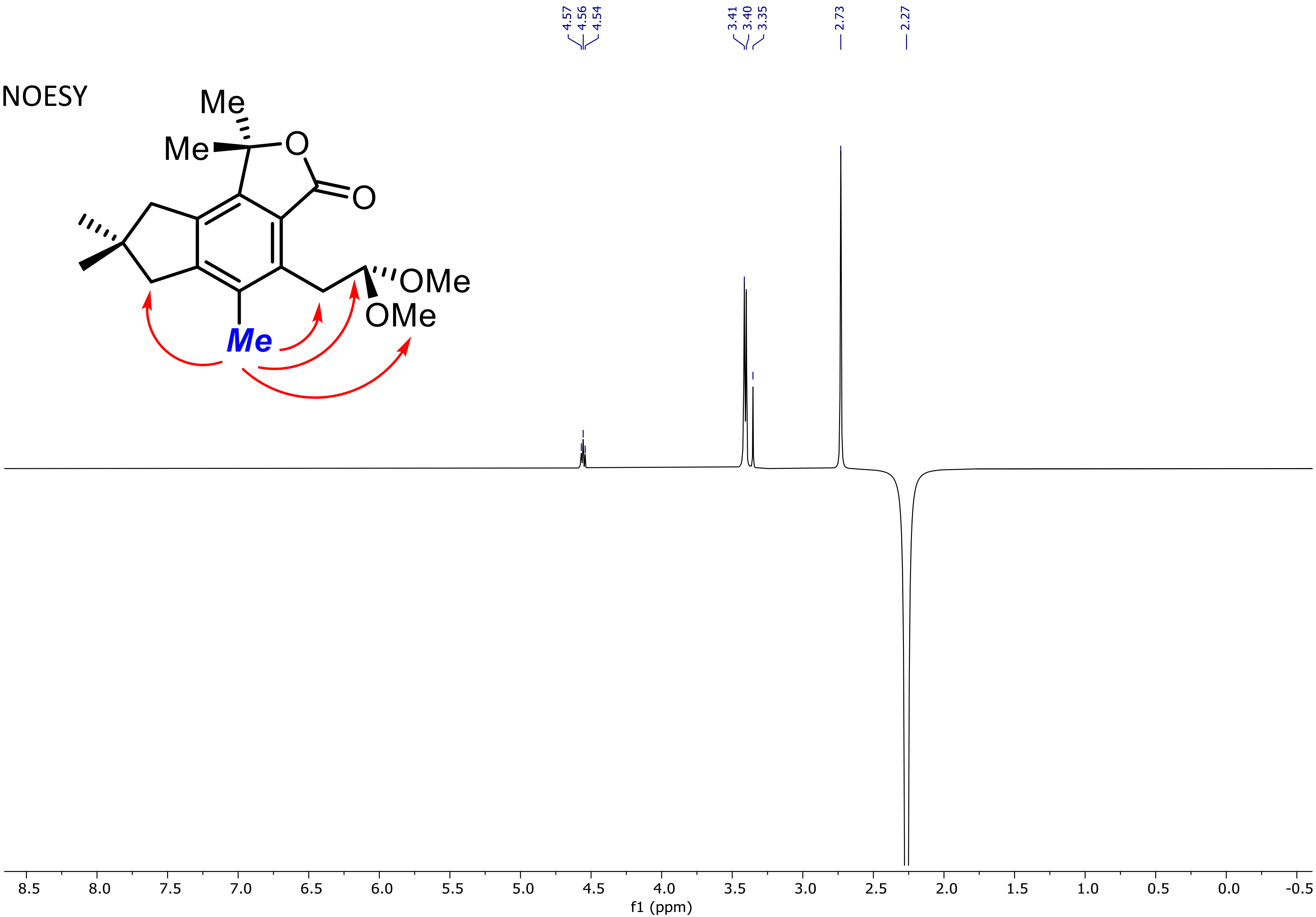

Me
Me
O
O
OMe
OMe
Me
4.57
4.56
4.54
3.41
3.40
3.35
2.73
2.27
8.5
8.0
7.5
7.0
6.5
6.0
5.5
5.0
4.5
4.0
3.5
3.0
2.5
2.0
1.5
1.0
0.5
0.0
-0.5
f1 (ppm)

1H NMR - 400 MHz, Chloroform-d

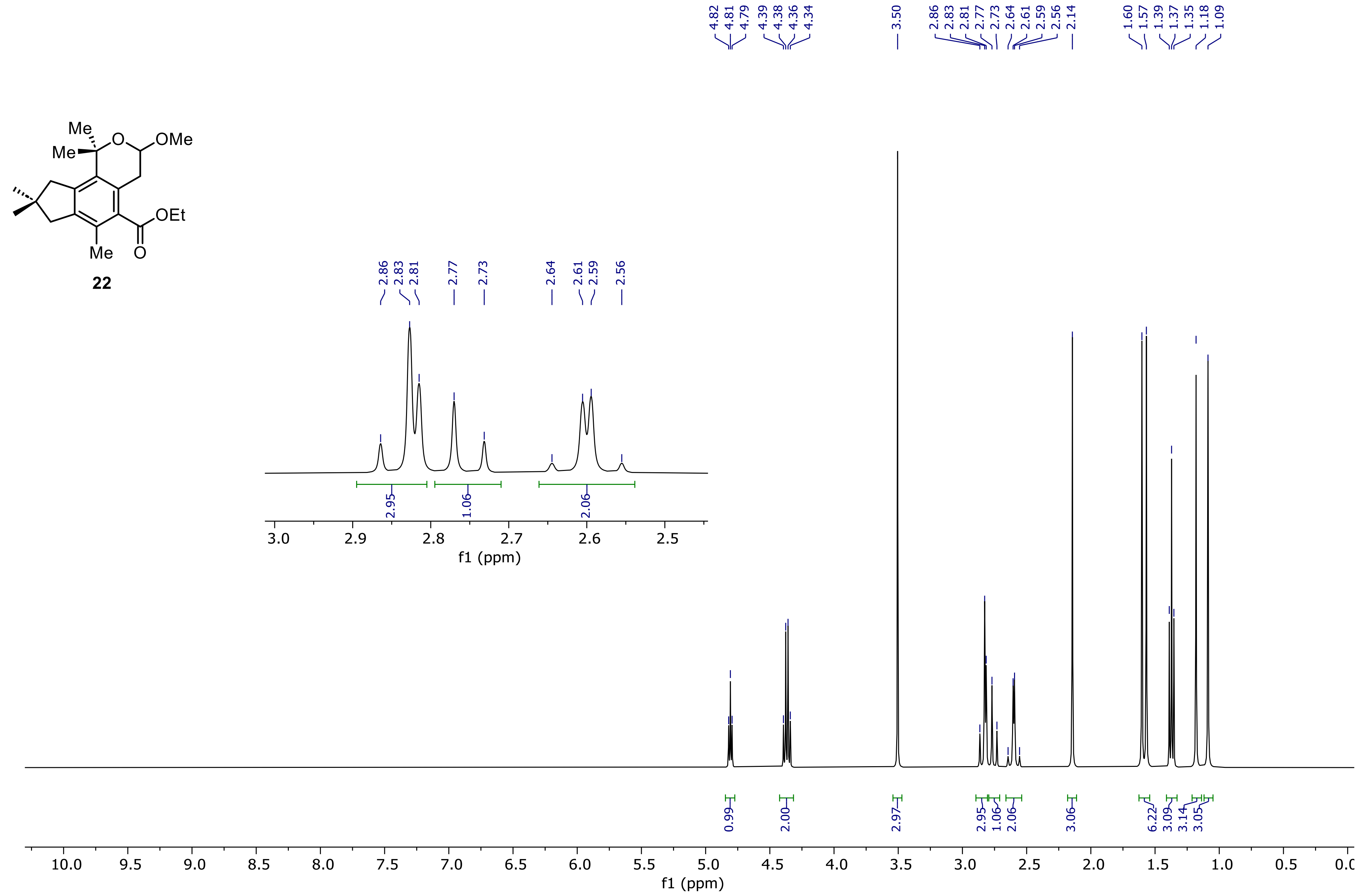

13C{1H} NMR - 101MHz, Chloroform-d

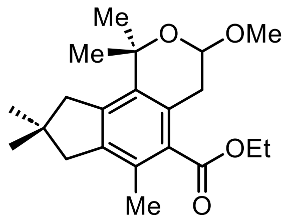
22

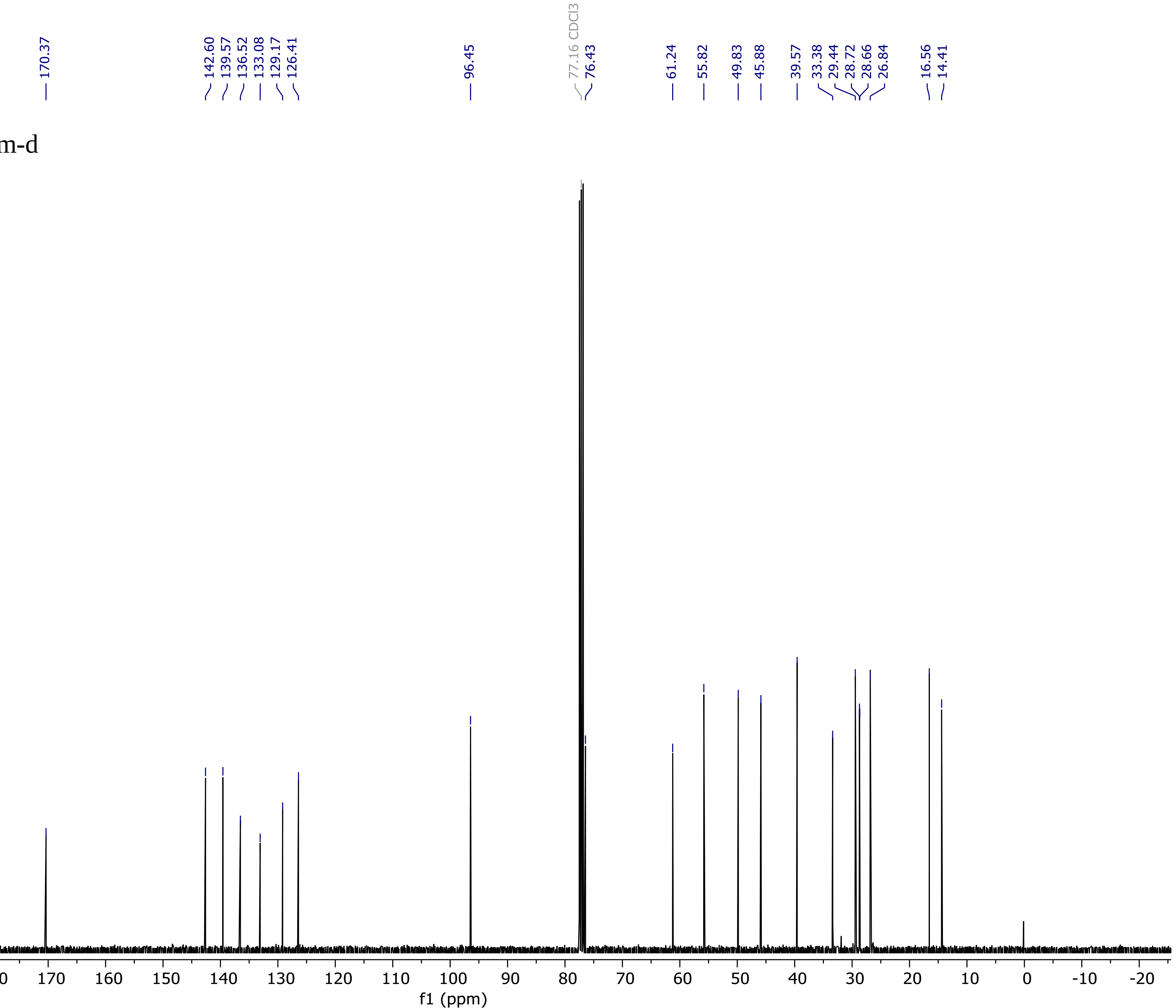

1D NOESY

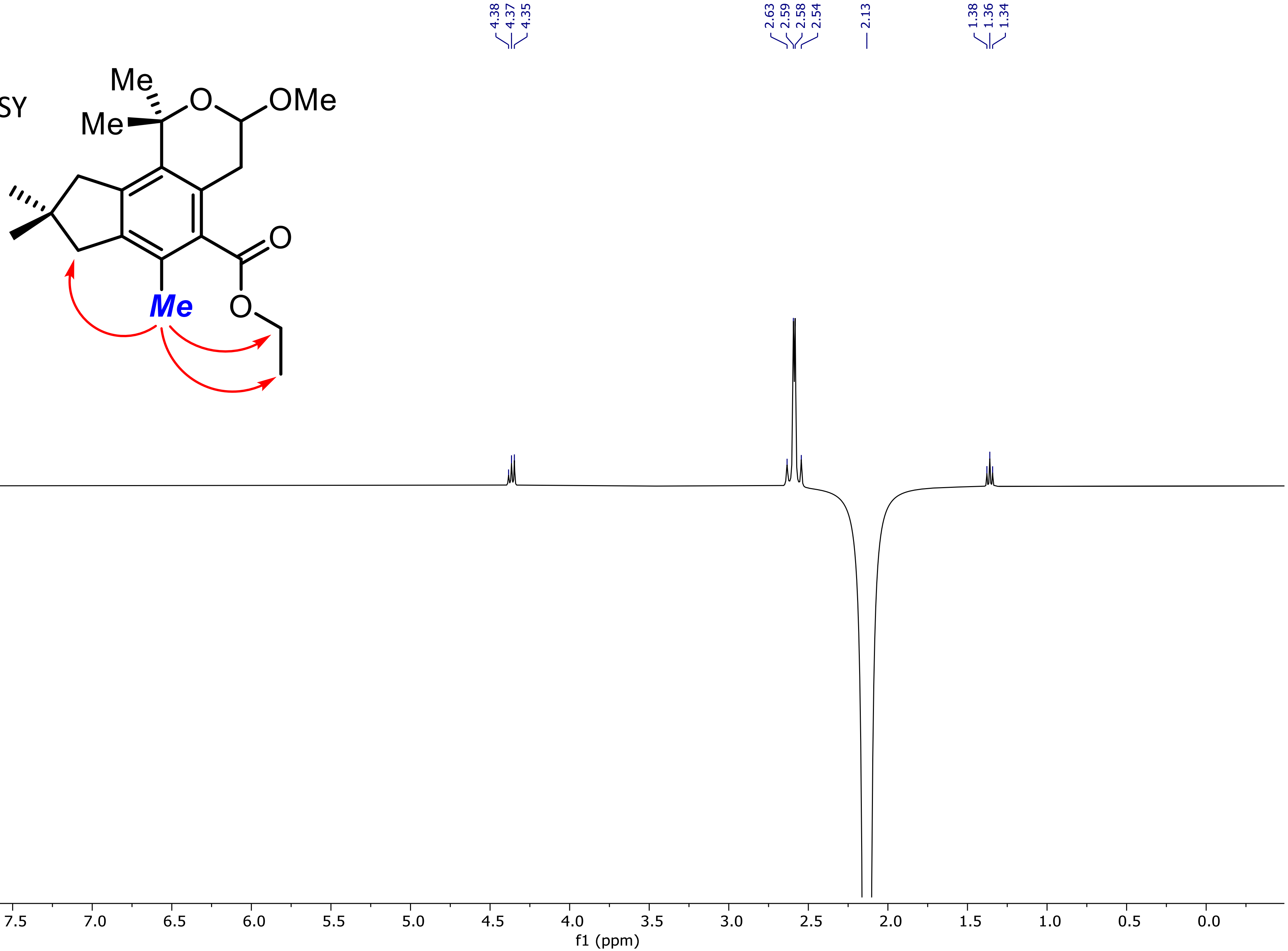
4.38
4.37
4.35
2.63
2.59
2.58
2.54
2.13
1.38
1.36
1.34
8.0
7.5
7.0
6.5
6.0
5.5
5.0
4.5
4.0
3.5
3.0
2.5
2.0
1.5
1.0
0.5
0.0
f1 (ppm)

1D NOESY

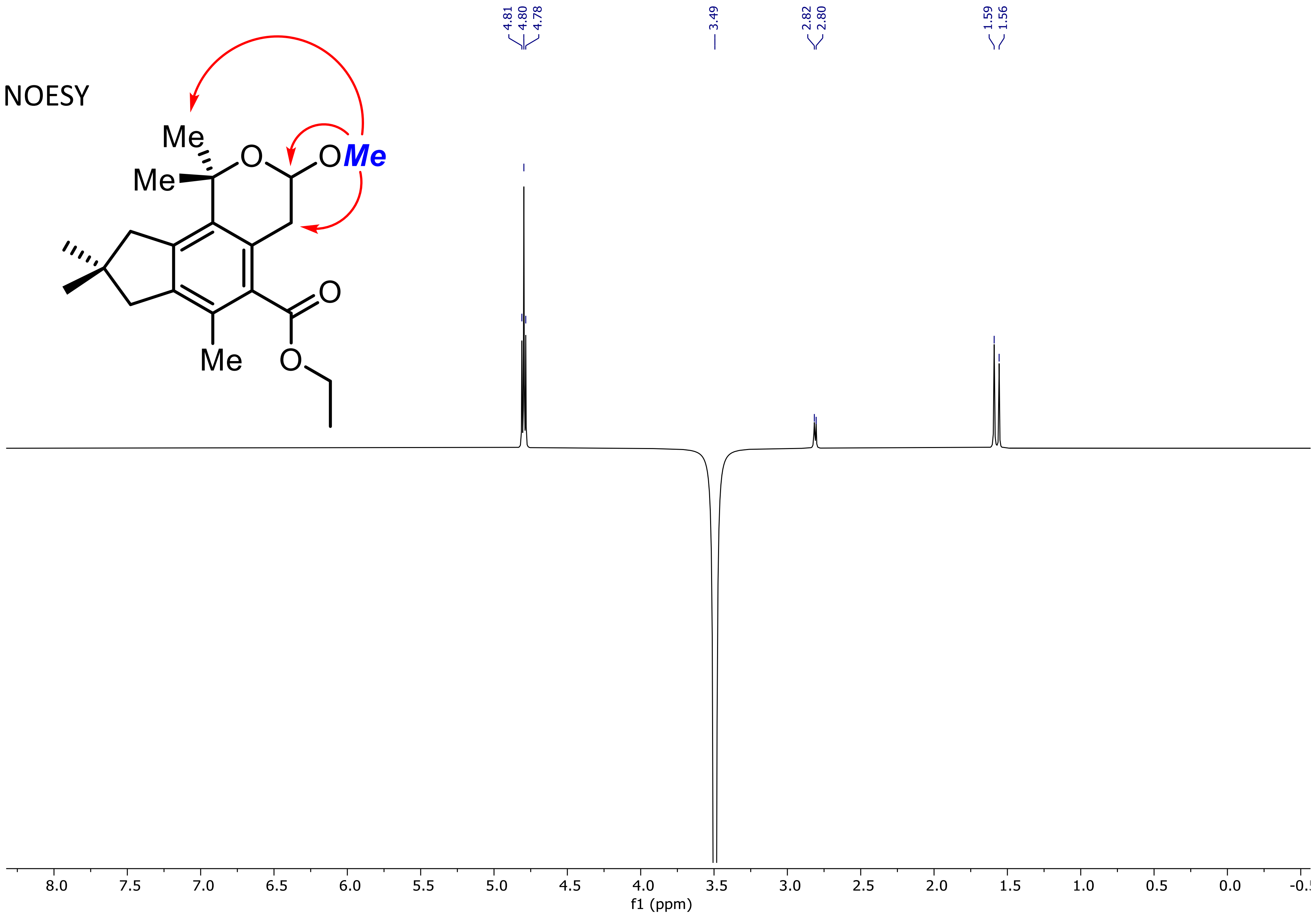
Me
Me
O
OMe
O
O
Me
4.81
4.80
4.78
3.49
2.82
2.80
1.59
1.56
8.0
7.5
7.0
6.5
6.0
5.5
5.0
4.5
4.0
3.5
3.0
2.5
2.0
1.5
1.0
0.5
0.0
f1 (ppm)

1H NMR - 400 MHz, Chloroform-d

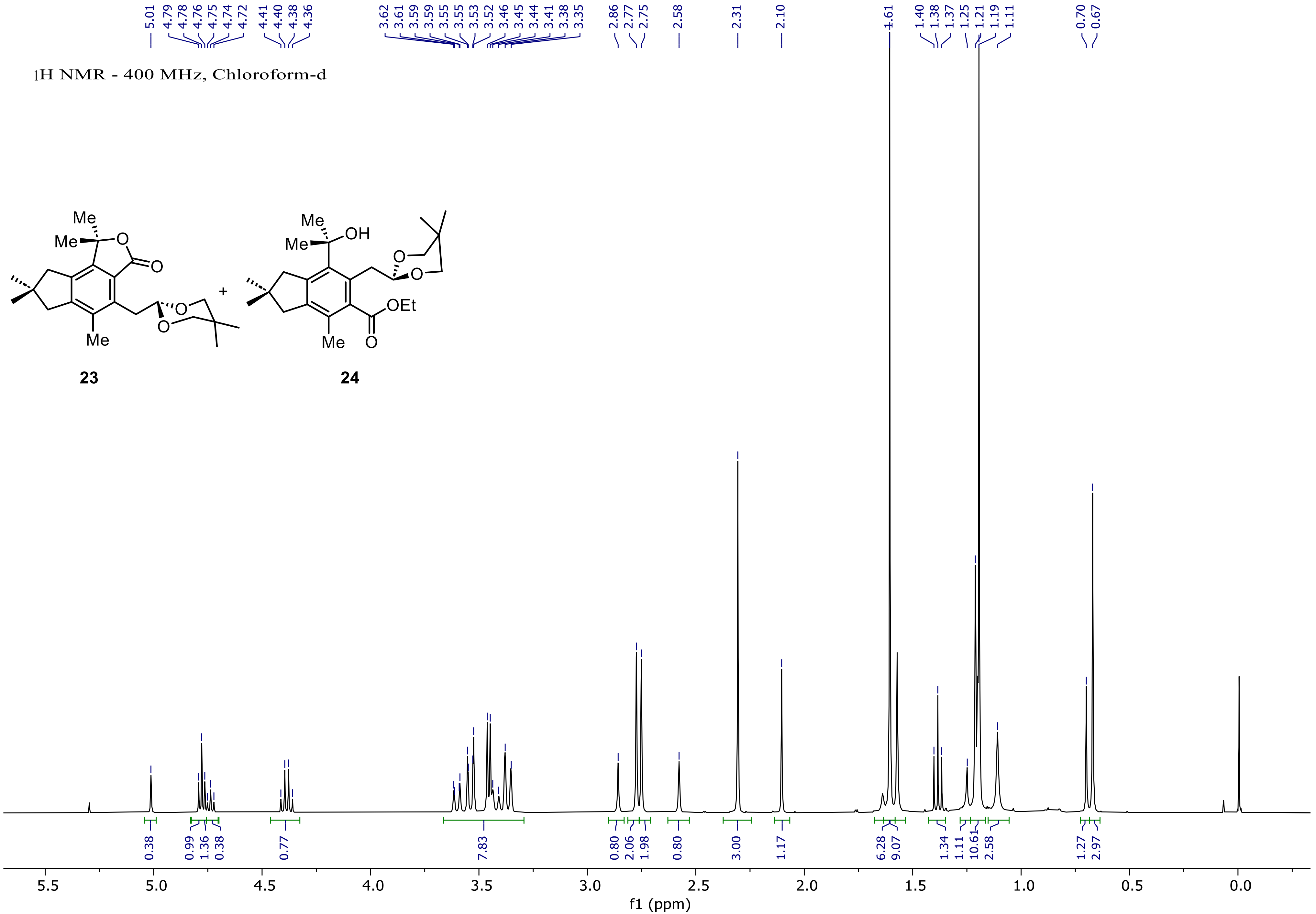

13C{1H} NMR - 101MHz, Chloroform-d

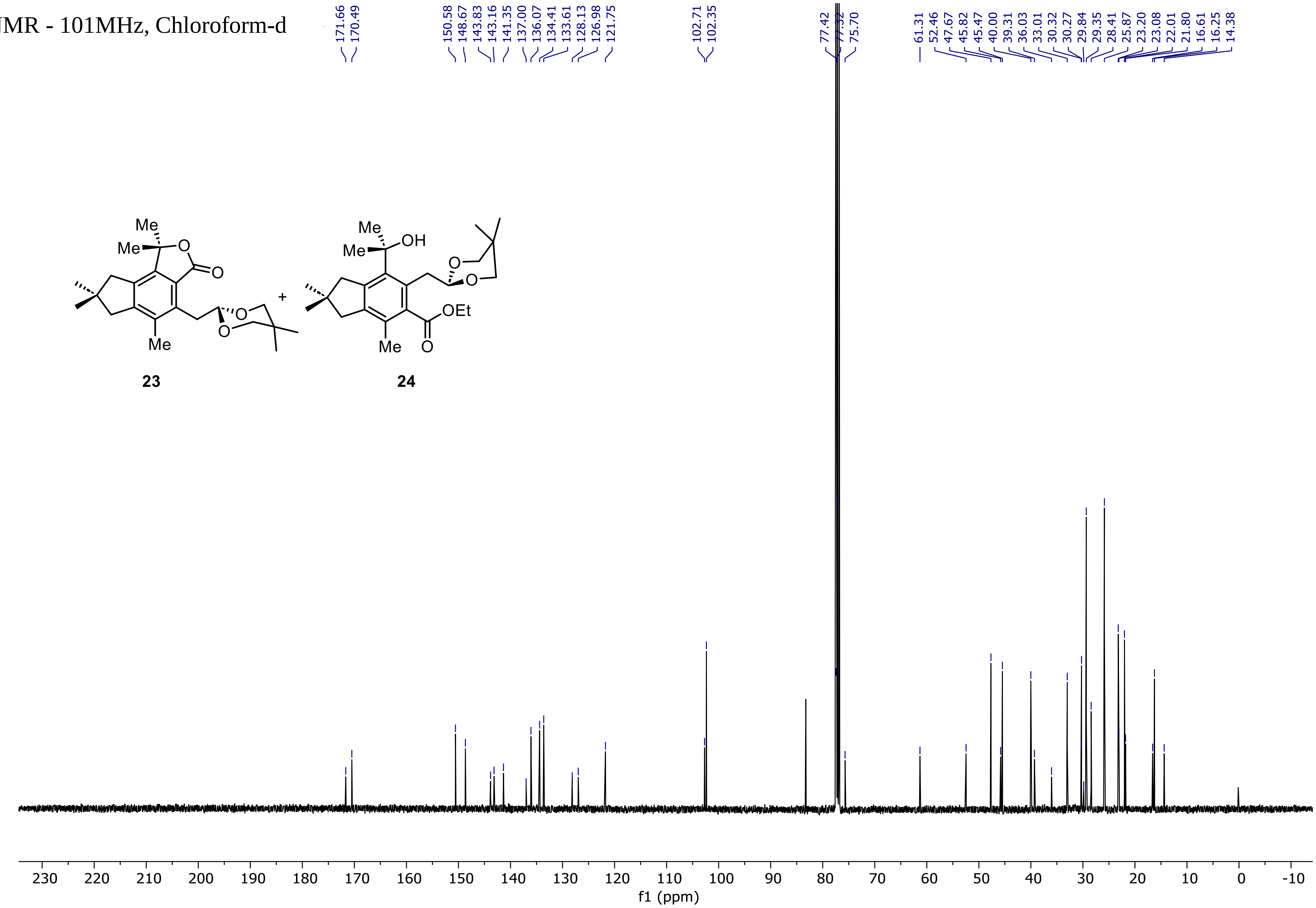

1D NOESY

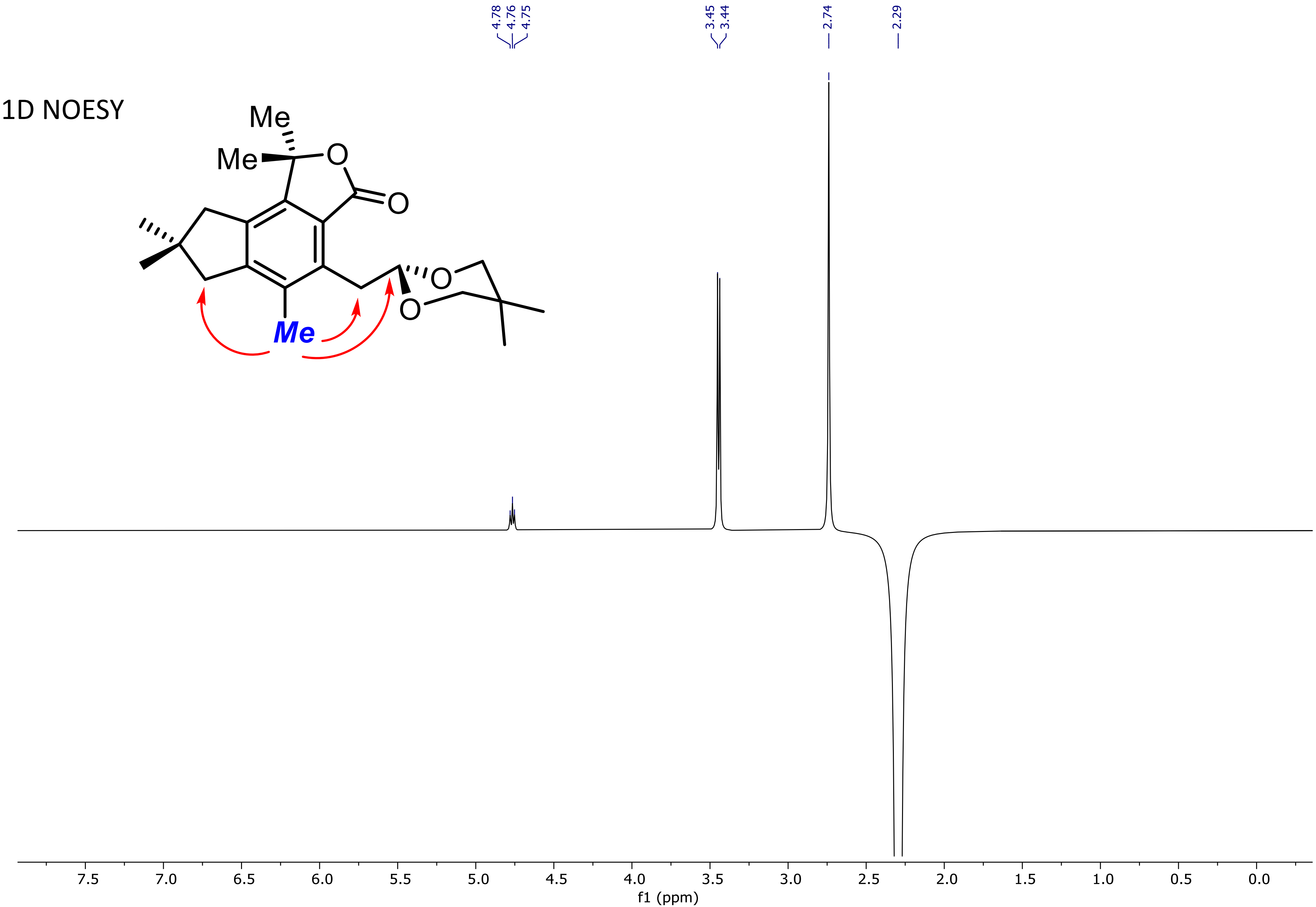
Me
Me
O
O
O
O
Me
4.78
4.76
4.75
3.45
3.44
2.74
2.29
7.5
7.0
6.5
6.0
5.5
5.0
4.5
4.0
3.5
3.0
2.5
2.0
1.5
1.0
0.5
0.0
f1 (ppm)

1D NOESY

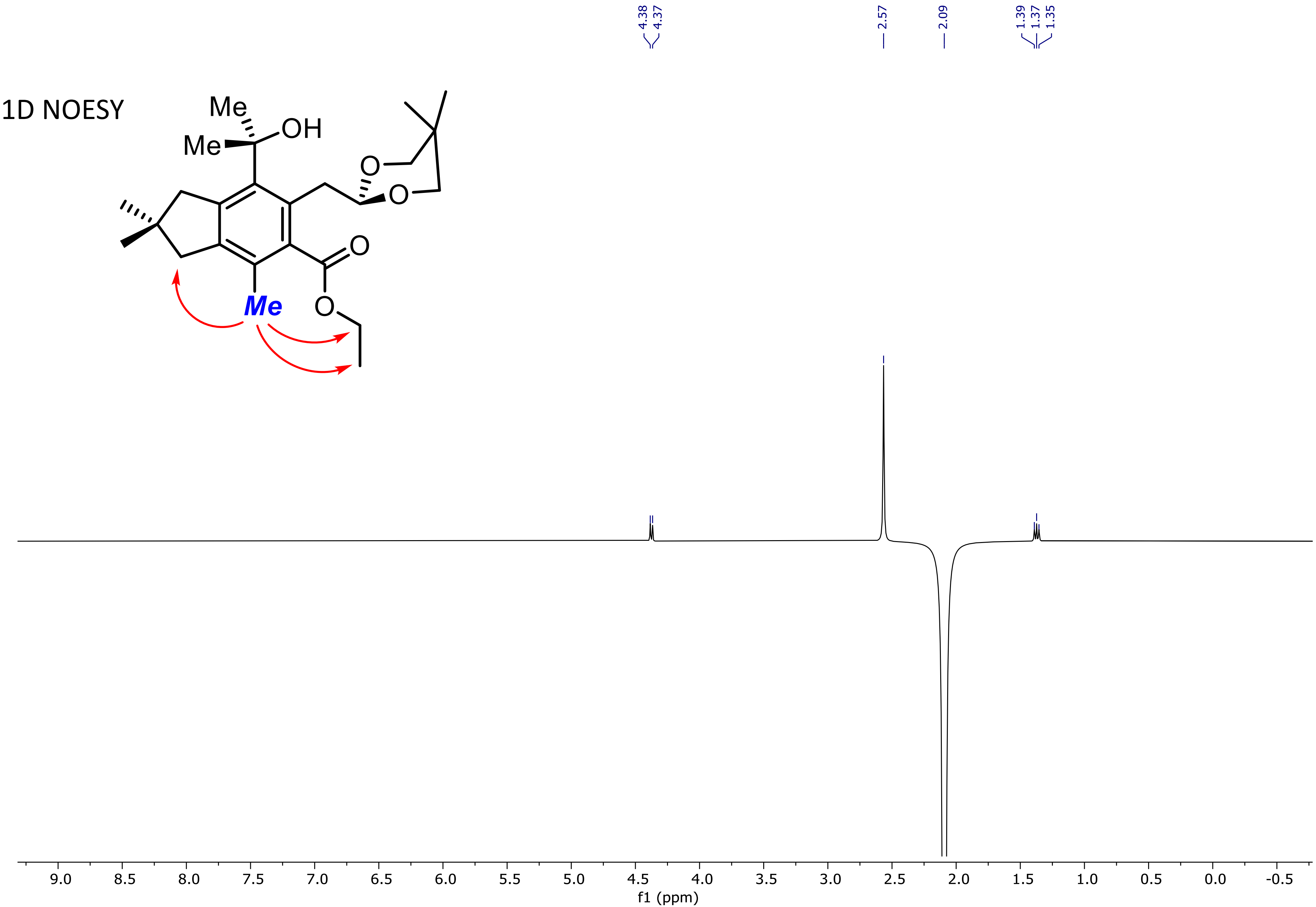

Me
OH
Me
O
O
O
Me
O
4.38
4.37
2.57
2.09
1.39
1.37
1.35
9.0
8.5
8.0
7.5
7.0
6.5
6.0
5.5
5.0
4.5
4.0
3.5
3.0
2.5
2.0
1.5
1.0
0.5
0.0
-0.5
f1 (ppm)

1H NMR - 400 MHz, Chloroform-d

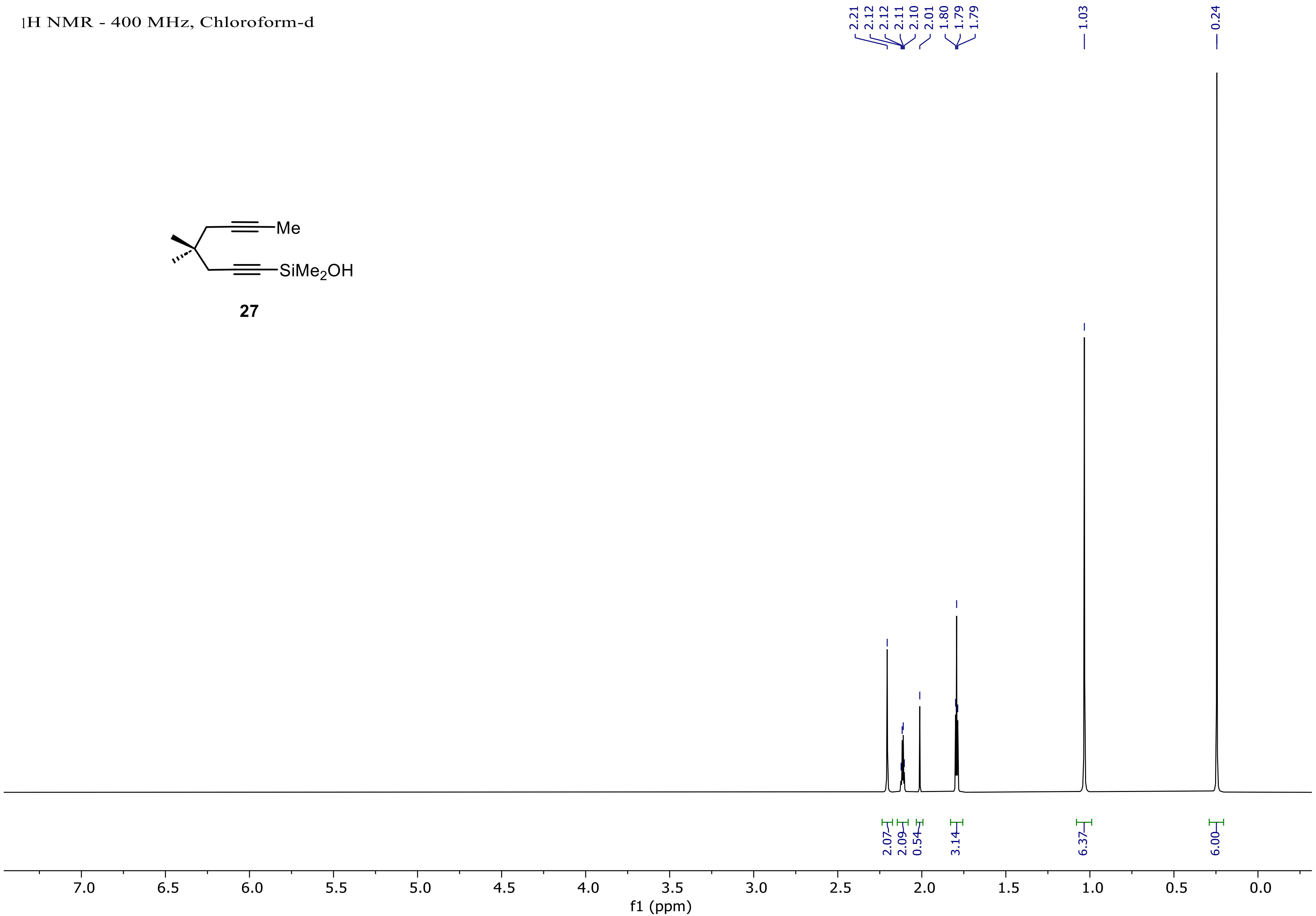

13C{1H} NMR - 101MHz, Chloroform-d

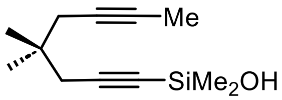

27

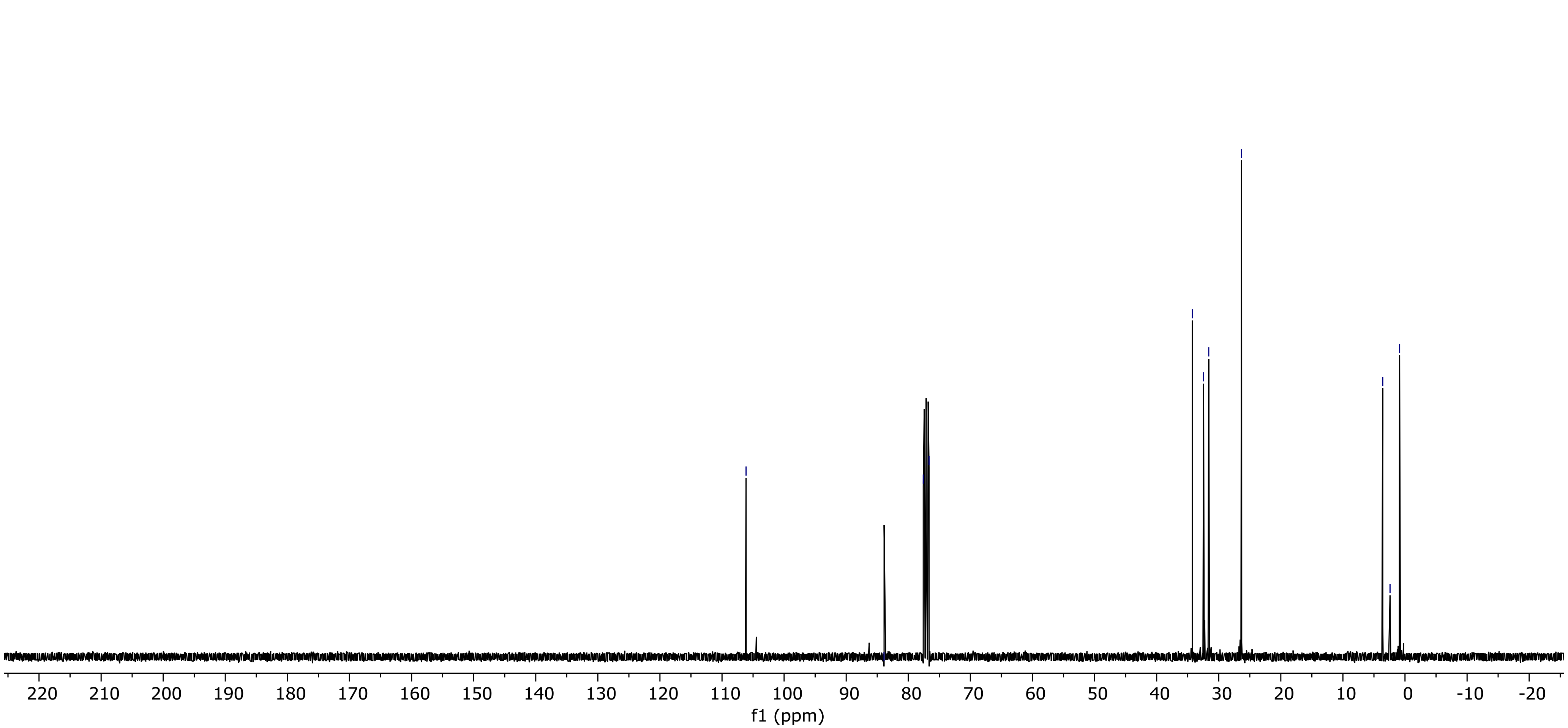

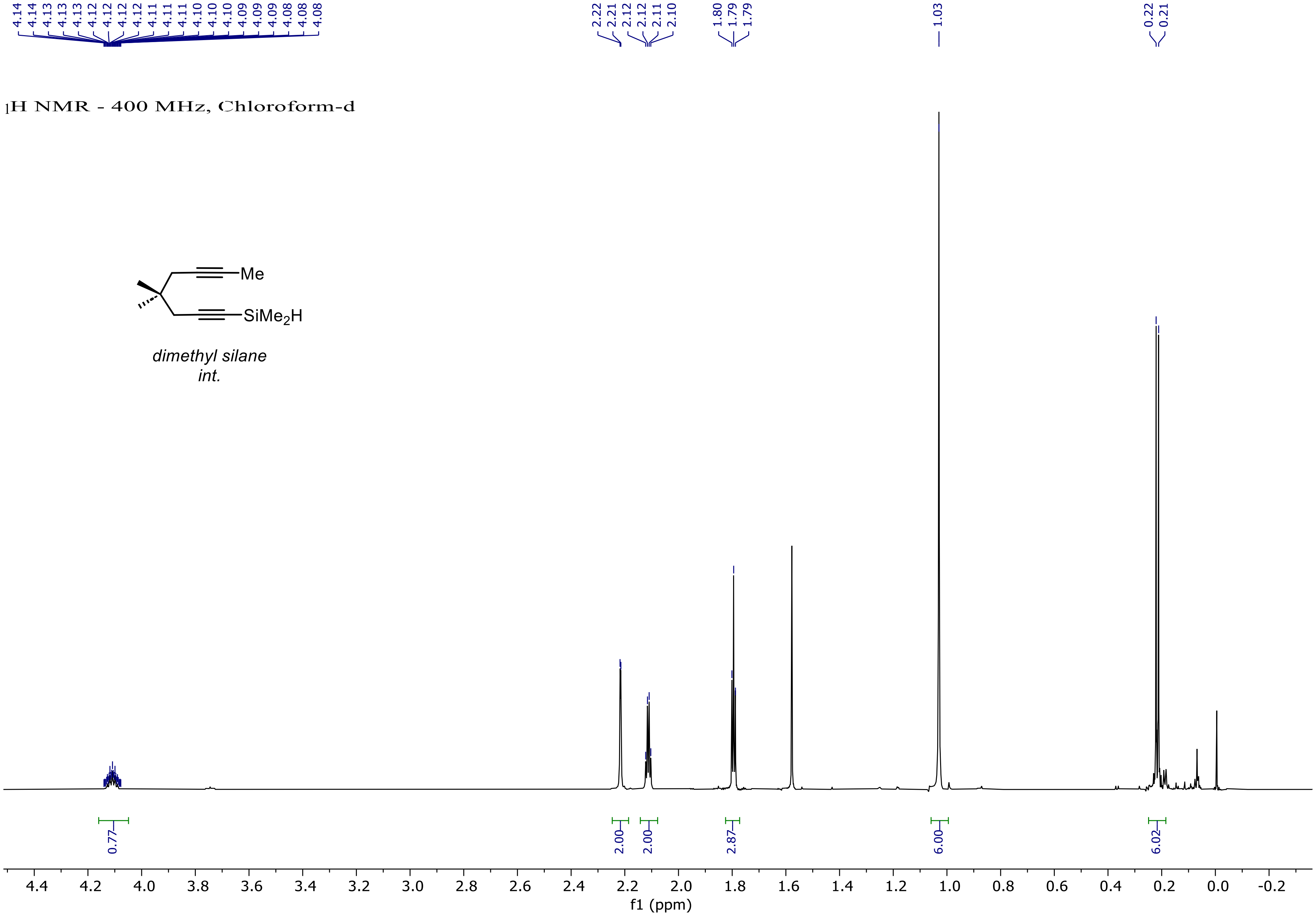
4.14
4.14
4.13
4.13
4.12
4.12
4.12
4.12
4.11
4.11
4.11
4.10
4.10
4.10
4.09
4.09
4.09
4.08
4.08
4.08
2.22
2.21
2.12
2.12
2.11
2.10
1.80
1.79
1.79
1.03
0.22
0.21
1H NMR - 400 MHz, Chloroform-d
Me
SiMe2H
dimethyl silane
int.
0.77
2.00
2.00
2.87
6.00
6.02
4.4
4.2
4.0
3.8
3.6
3.4
3.2
3.0
2.8
2.6
2.4
2.2
2.0
1.8
1.6
1.4
1.2
1.0
0.8
0.6
0.4
0.2
0.0
-0.2
f1 (ppm)

1H NMR - 400 MHz, Chloroform-d

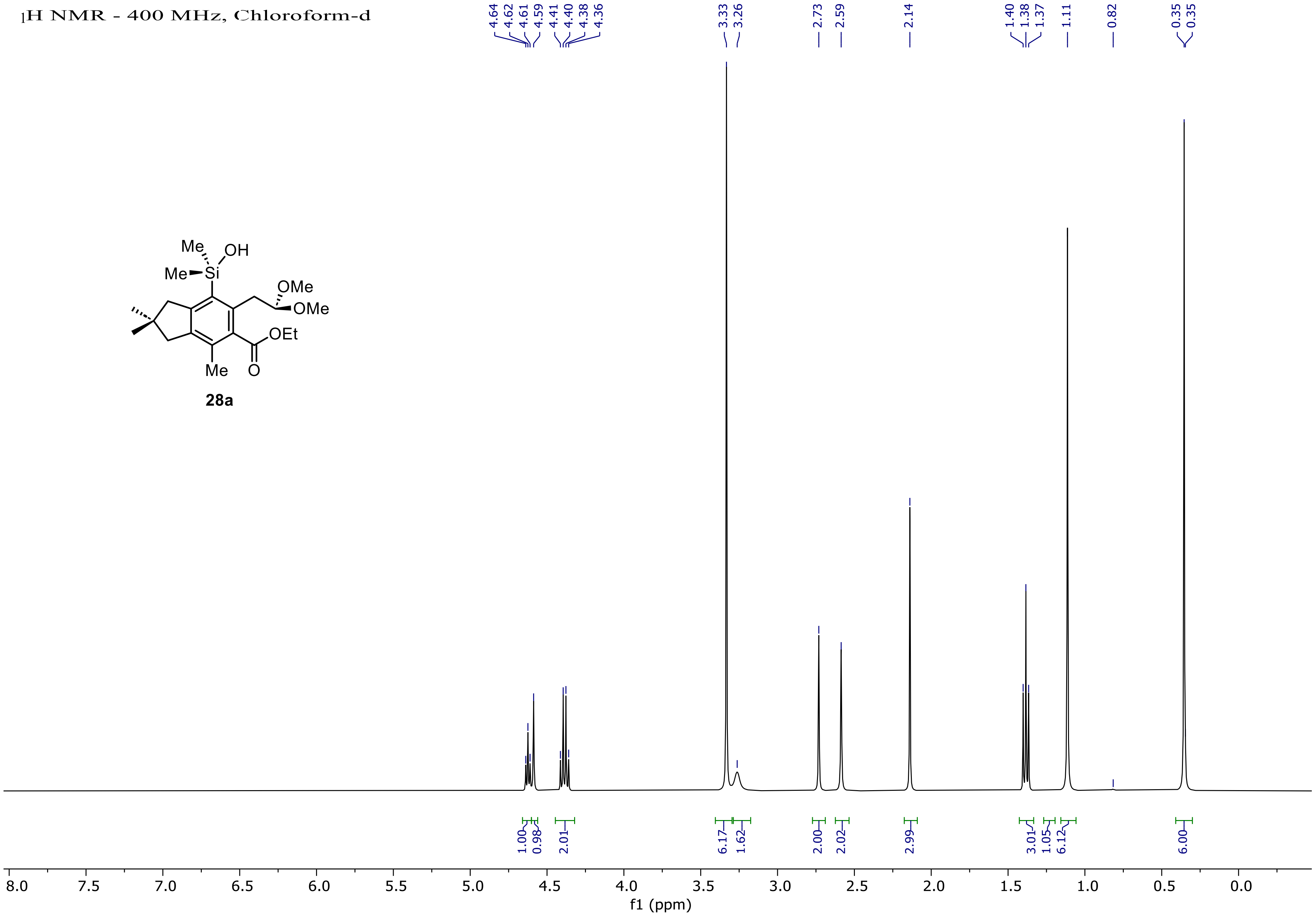

28a

13C{1H} NMR - 101MHz, Chloroform-d

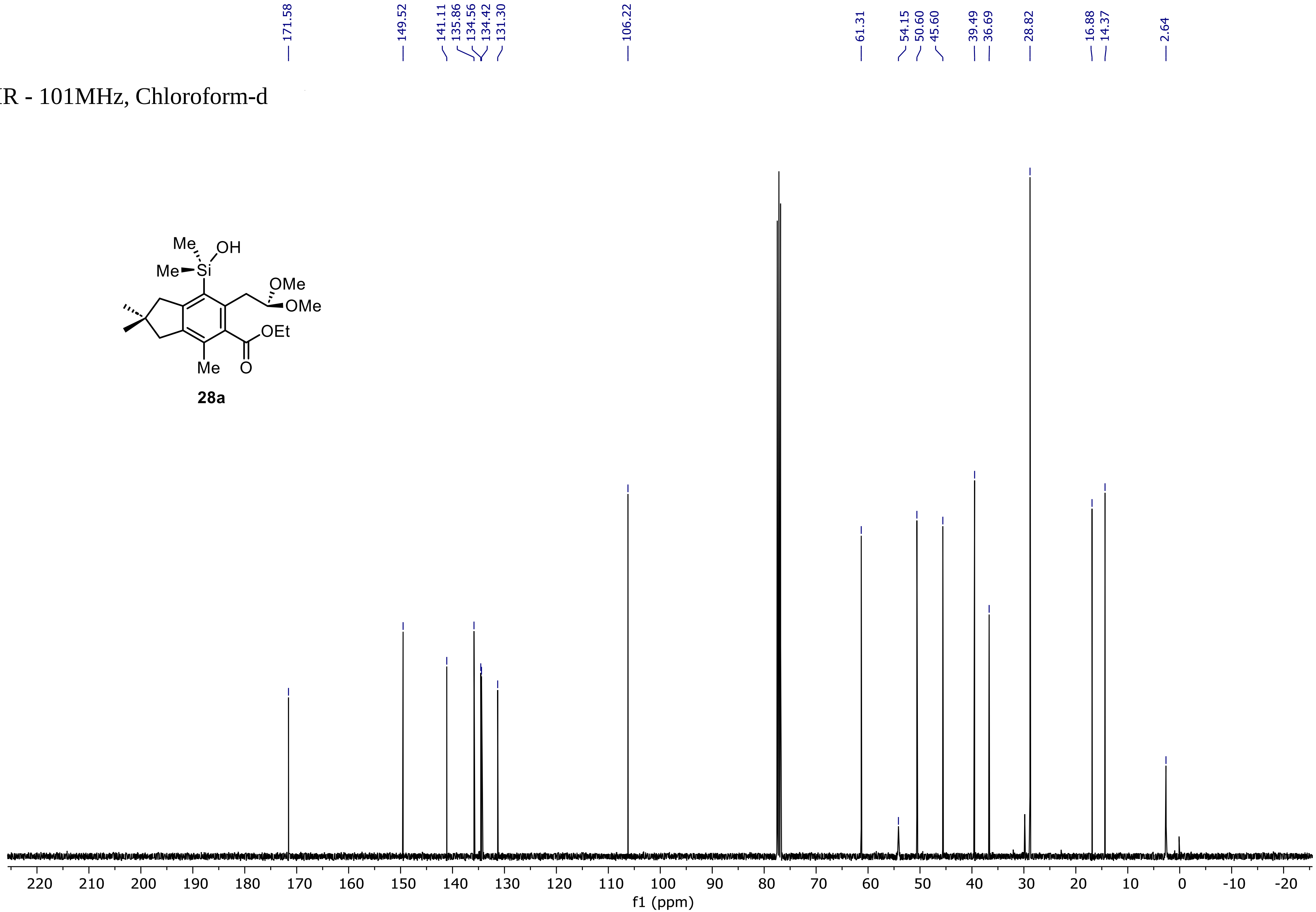

28a

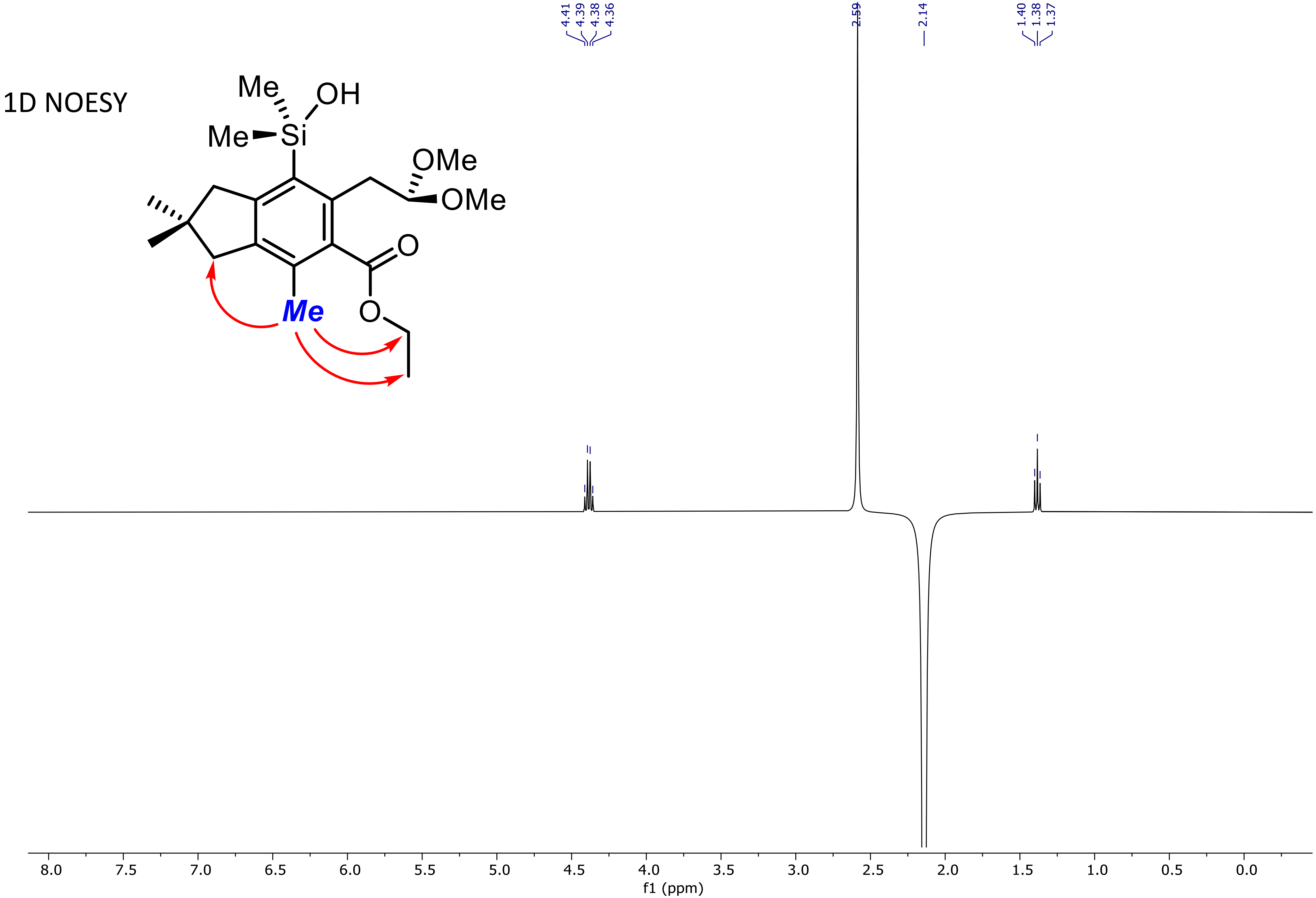

1D NOESY
Me
OH
Me
Si
OMe
OMe
O
Me
O
4.41
4.39
4.38
4.36
2.59
2.14
1.40
1.38
1.37
8.0
7.5
7.0
6.5
6.0
5.5
5.0
4.5
4.0
3.5
3.0
2.5
2.0
1.5
1.0
0.5
0.0
f1 (ppm)

1D NOESY

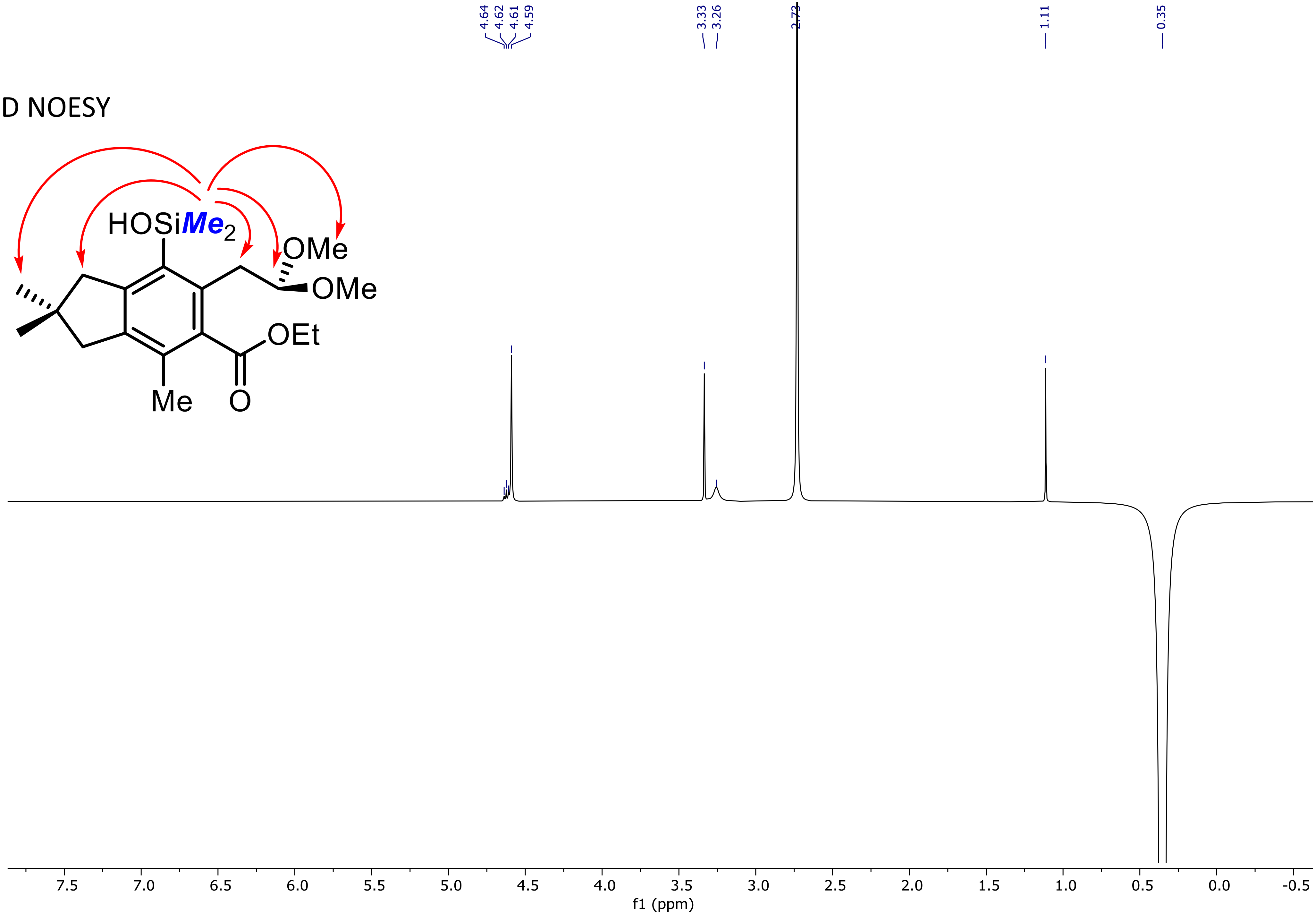

HOSiMe2
OMe
OMe
OEt
Me
O
4.64
4.62
4.61
4.59
3.33
3.26
2.73
1.11
0.35
7.5
7.0
6.5
6.0
5.5
5.0
4.5
4.0
3.5
3.0
2.5
2.0
1.5
1.0
0.5
0.0
-0.5
f1 (ppm)

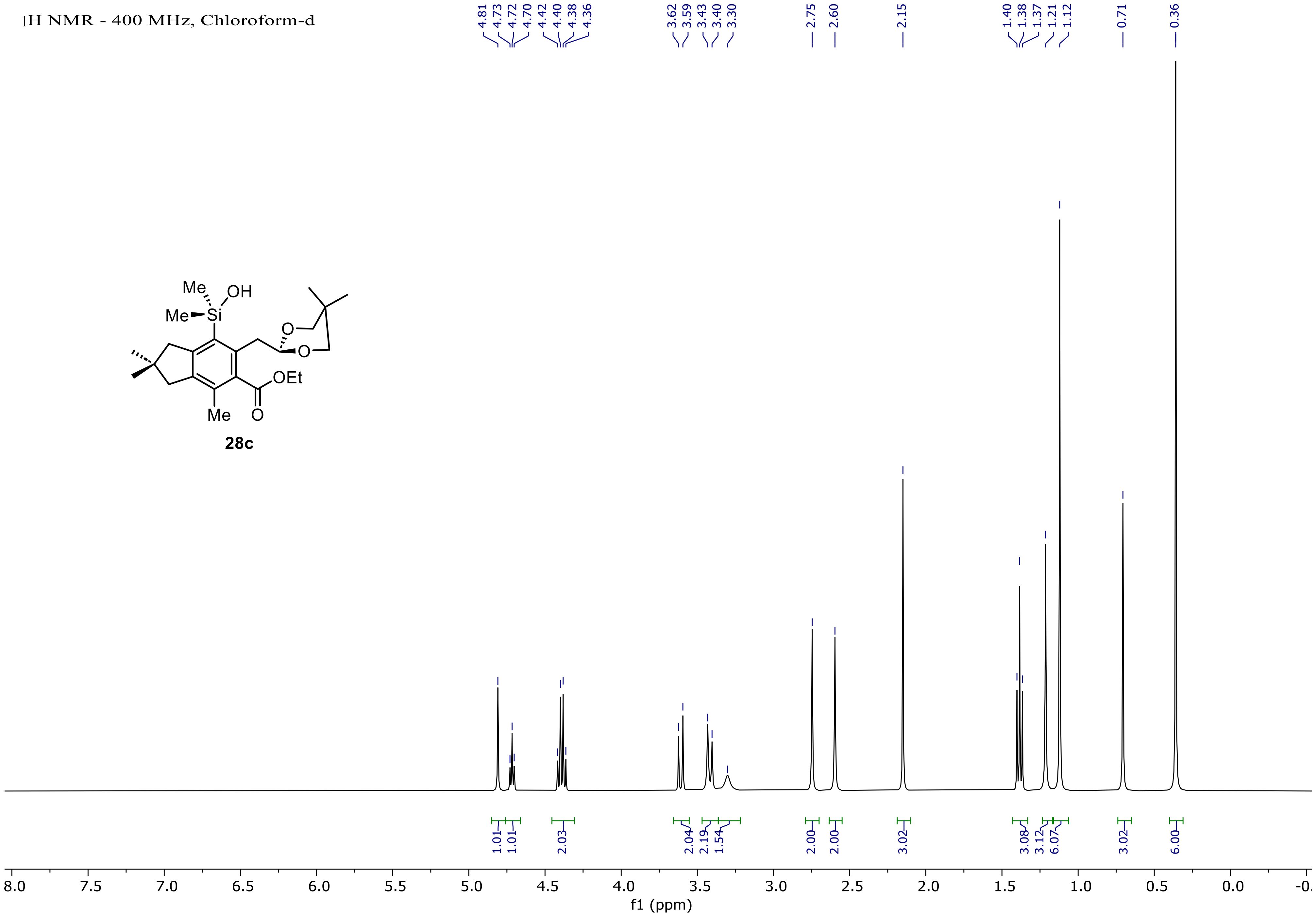
1H NMR - 400 MHz, Chloroform-d
4.81 4.73 4.72 4.70 4.42 4.40 4.38 4.36 3.62 3.59 3.43 3.40 3.30 2.75 2.60 2.15 1.40 1.38 1.37 1.21 1.12 0.71 0.36
Me
OH
Me
Si
O
O
OEt
Me
O
28c
1.01 1.01 2.03 2.04 2.19 1.54 2.00 2.00 3.02 3.08 3.12 6.07 3.02 6.00
8.0 7.5 7.0 6.5 6.0 5.5 5.0 4.5 4.0 3.5 3.0 2.5 2.0 1.5 1.0 0.5 0.0
f1 (ppm)

13C{1H} NMR - 101MHz, Chloroform-d

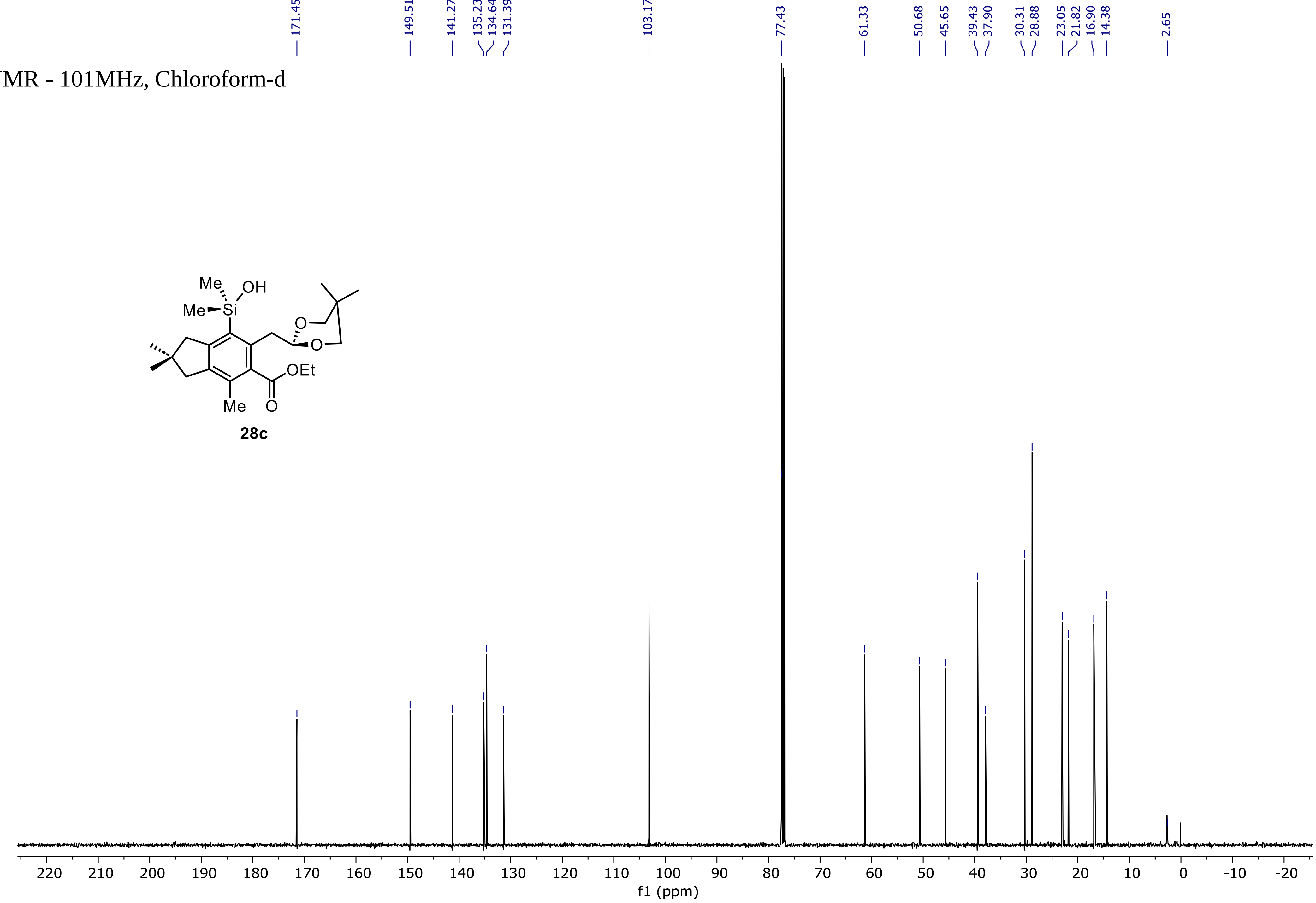

28c

1D NOESY

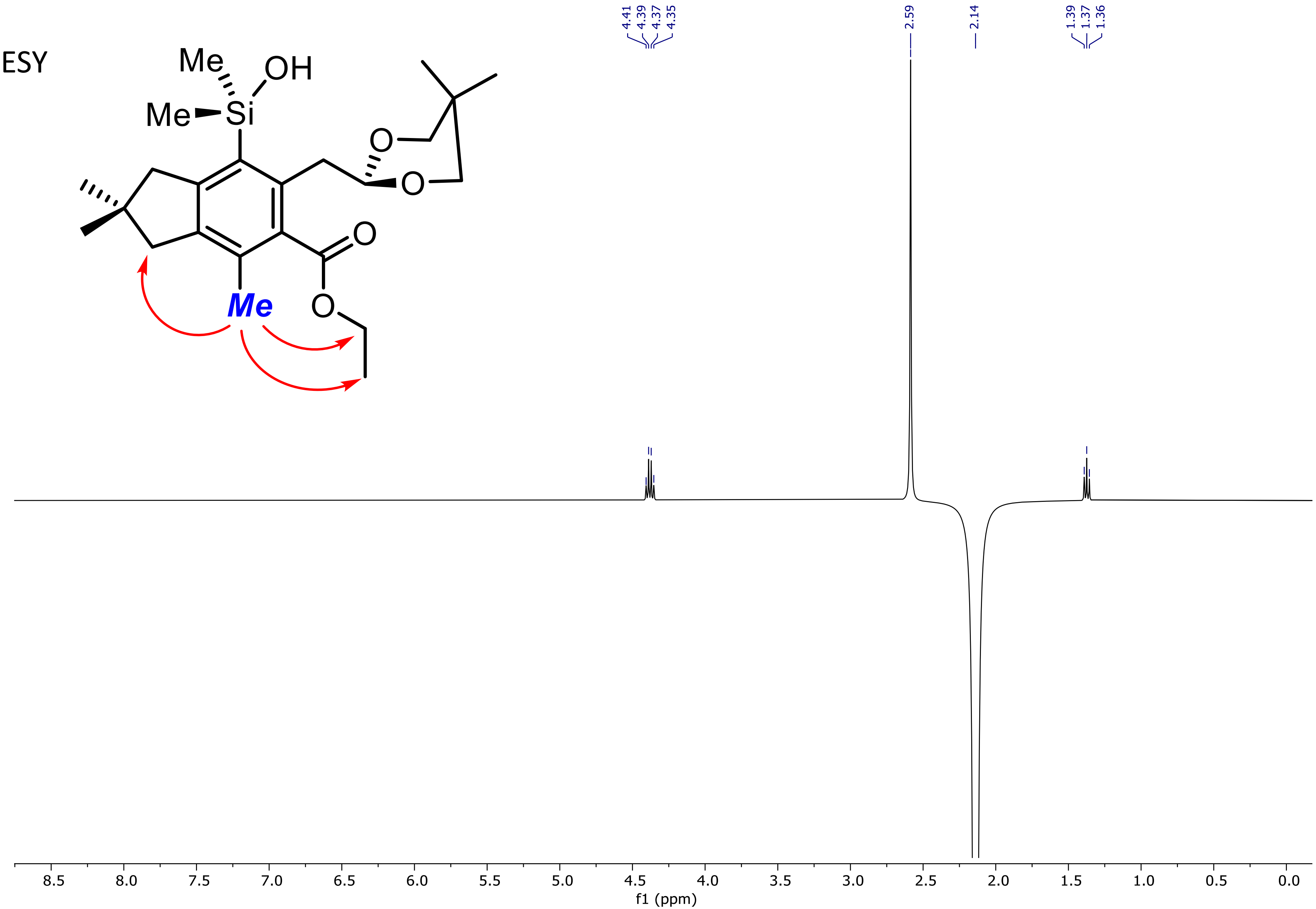
Me
OH
Me
Si
O
O
O
Me
O
4.41
4.39
4.37
4.35
2.59
2.14
1.39
1.37
1.36
8.5
8.0
7.5
7.0
6.5
6.0
5.5
5.0
4.5
4.0
3.5
3.0
2.5
2.0
1.5
1.0
0.5
0.0
f1 (ppm)

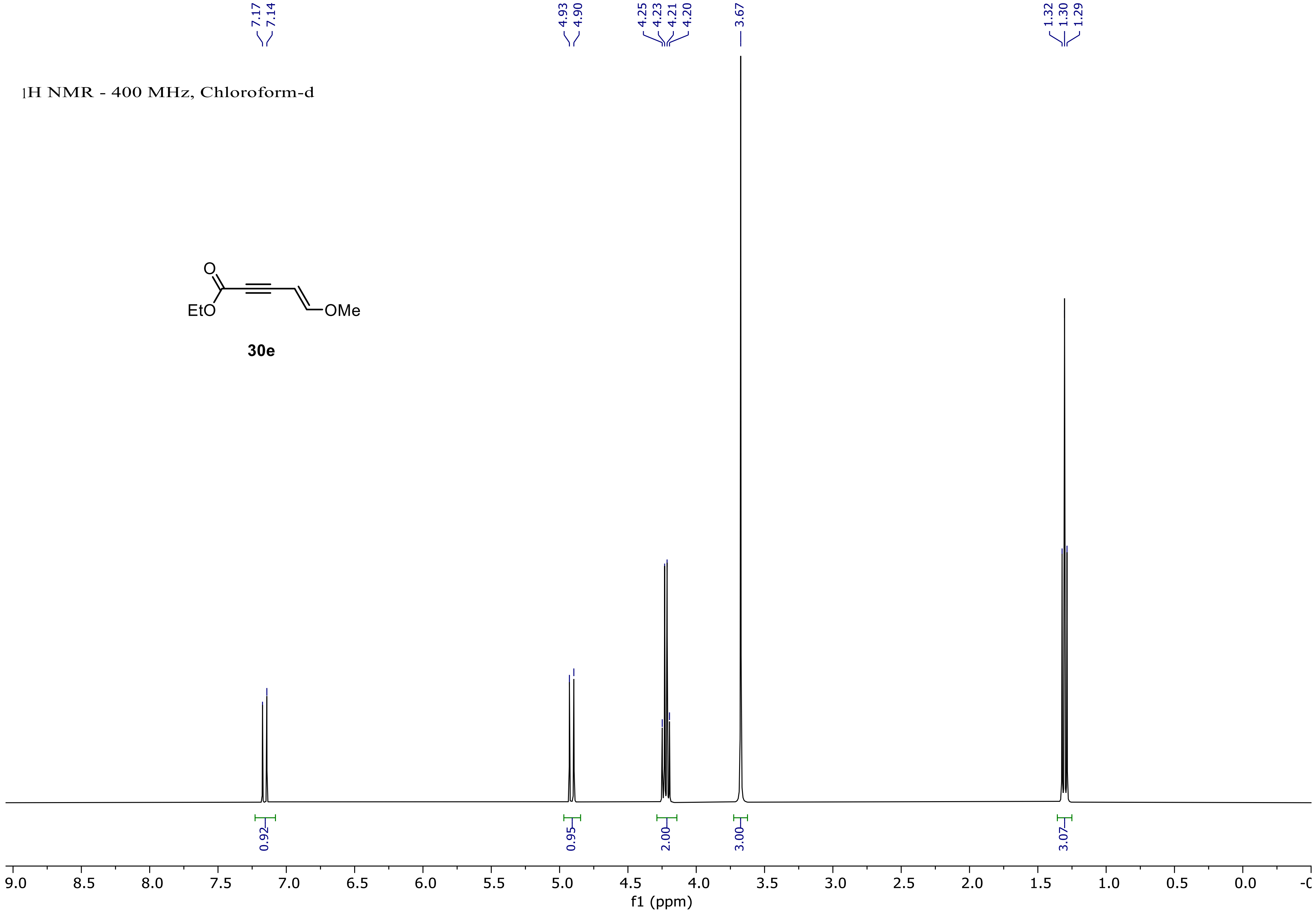
7.17
7.14
4.93
4.90
4.25
4.23
4.21
4.20
3.67
1.32
1.30
1.29
1H NMR - 400 MHz, Chloroform-d
O
EtO
OMe
30e
0.92
0.95
2.00
3.00
3.07
9.0 8.5 8.0 7.5 7.0 6.5 6.0 5.5 5.0 4.5 4.0 3.5 3.0 2.5 2.0 1.5 1.0 0.5 0.0
f1 (ppm)

13C{1H} NMR - 101MHz, Chloroform-d

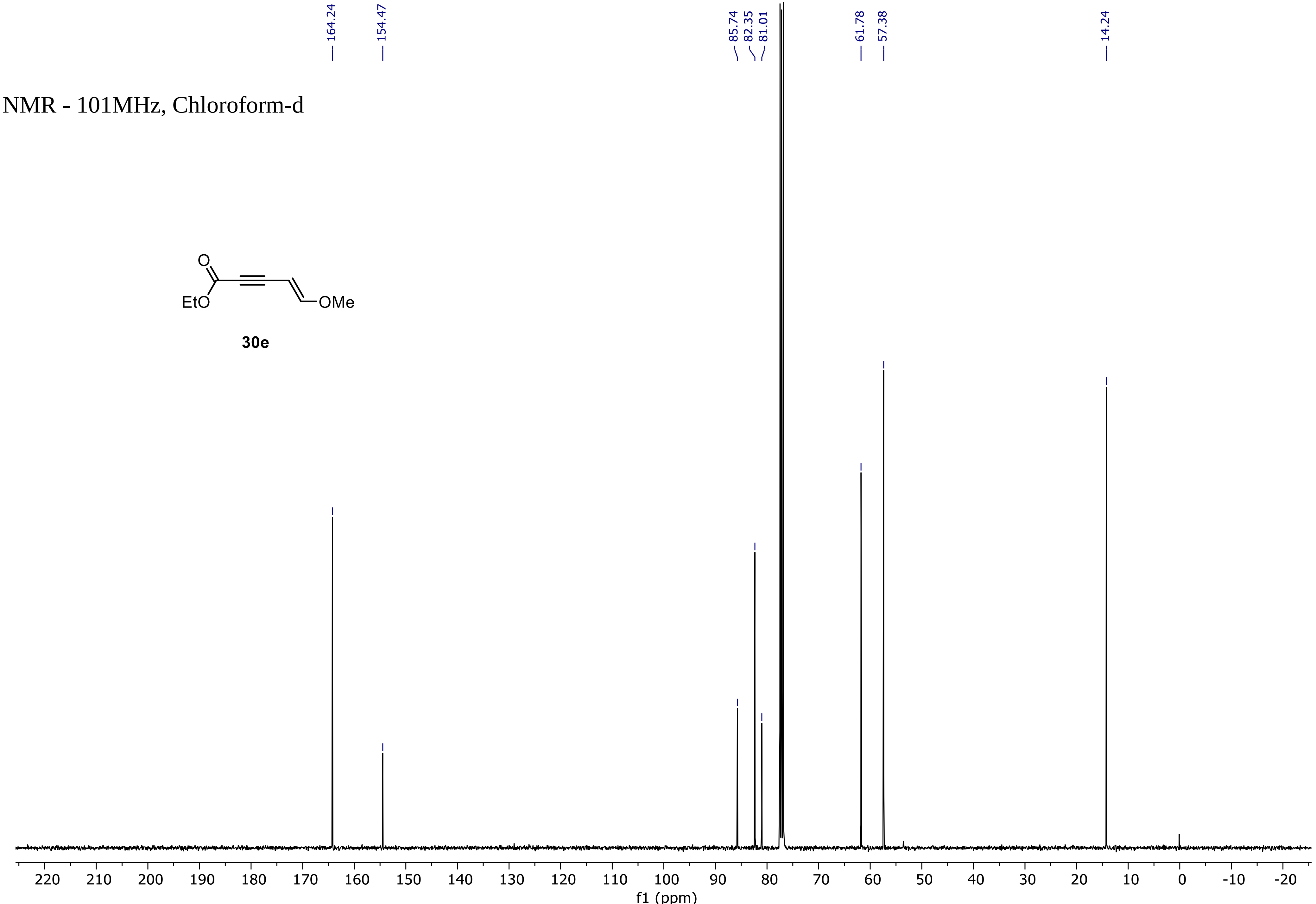

30e

1H NMR - 400 MHz, Chloroform-d

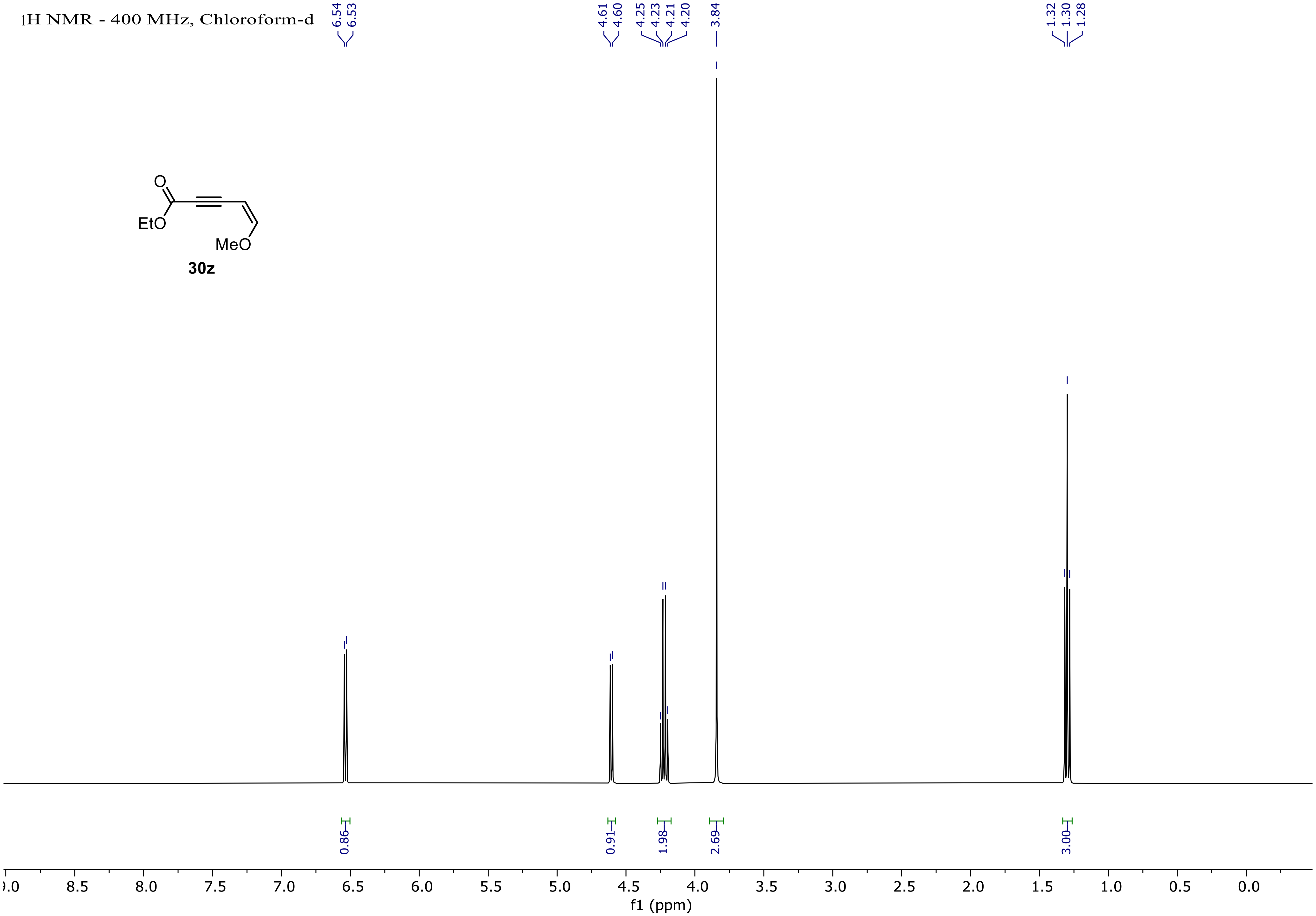

13C{1H} NMR - 101MHz, Chloroform-d

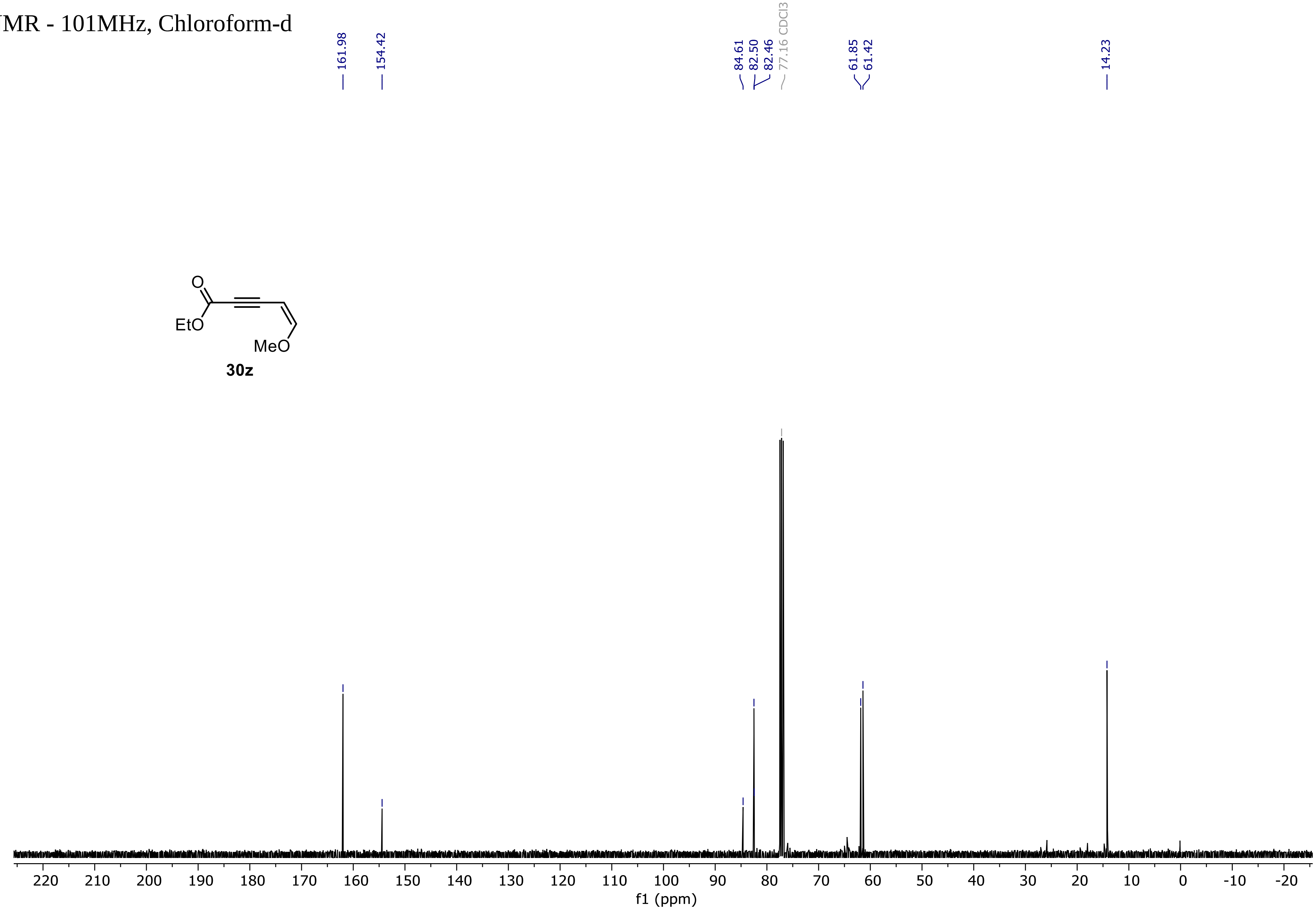

30z

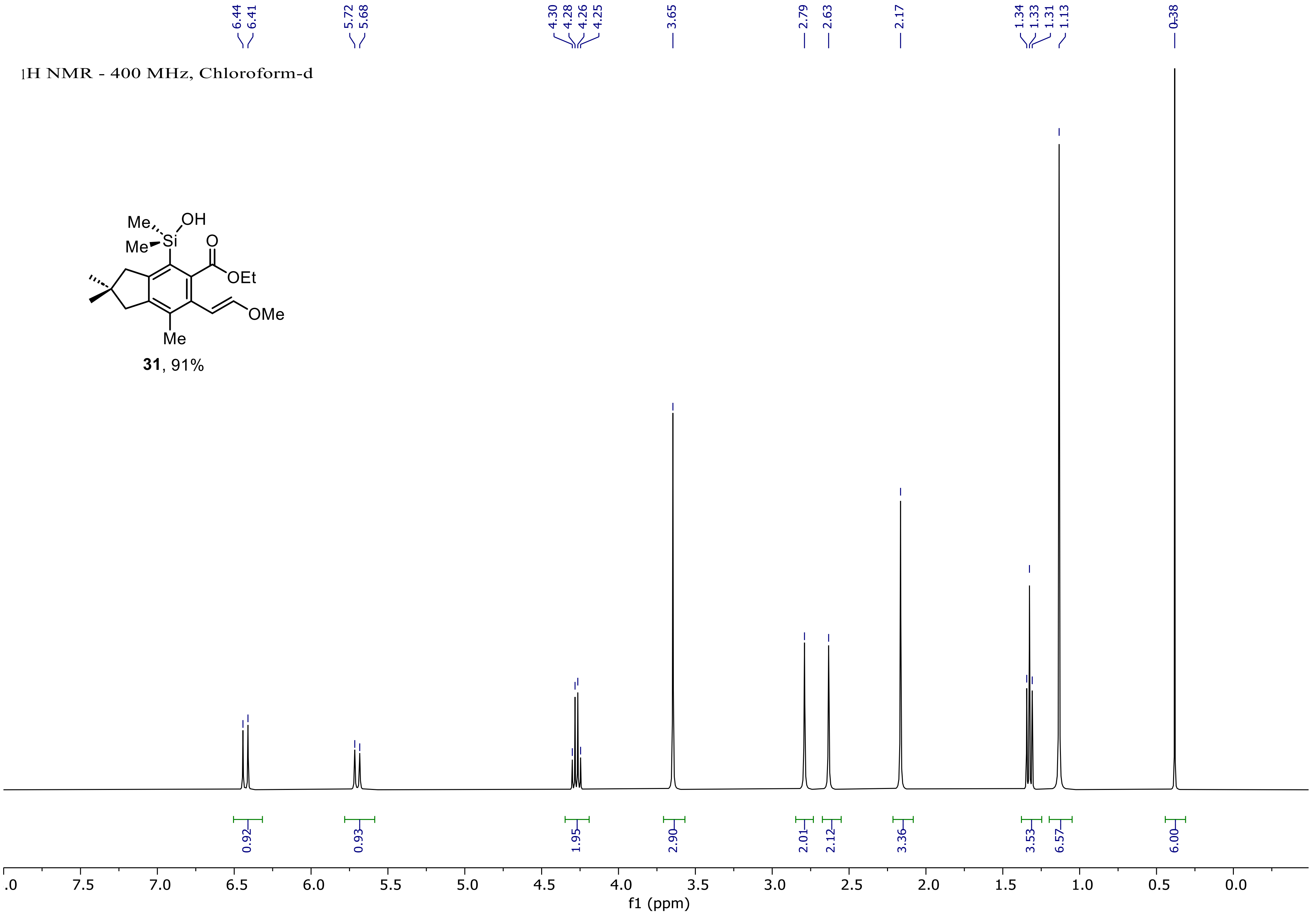
1H NMR - 400 MHz, Chloroform-d
Me
OH
Me
Si
O
OEt
OMe
Me
31, 91%
6.44
6.41
5.72
5.68
4.30
4.28
4.26
4.25
3.65
2.79
2.63
2.17
1.34
1.33
1.31
1.13
0.38
0.92
0.93
1.95
2.90
2.01
2.12
3.36
3.53
6.57
6.00
f1 (ppm)

13C{1H} NMR - 101MHz, Chloroform-d

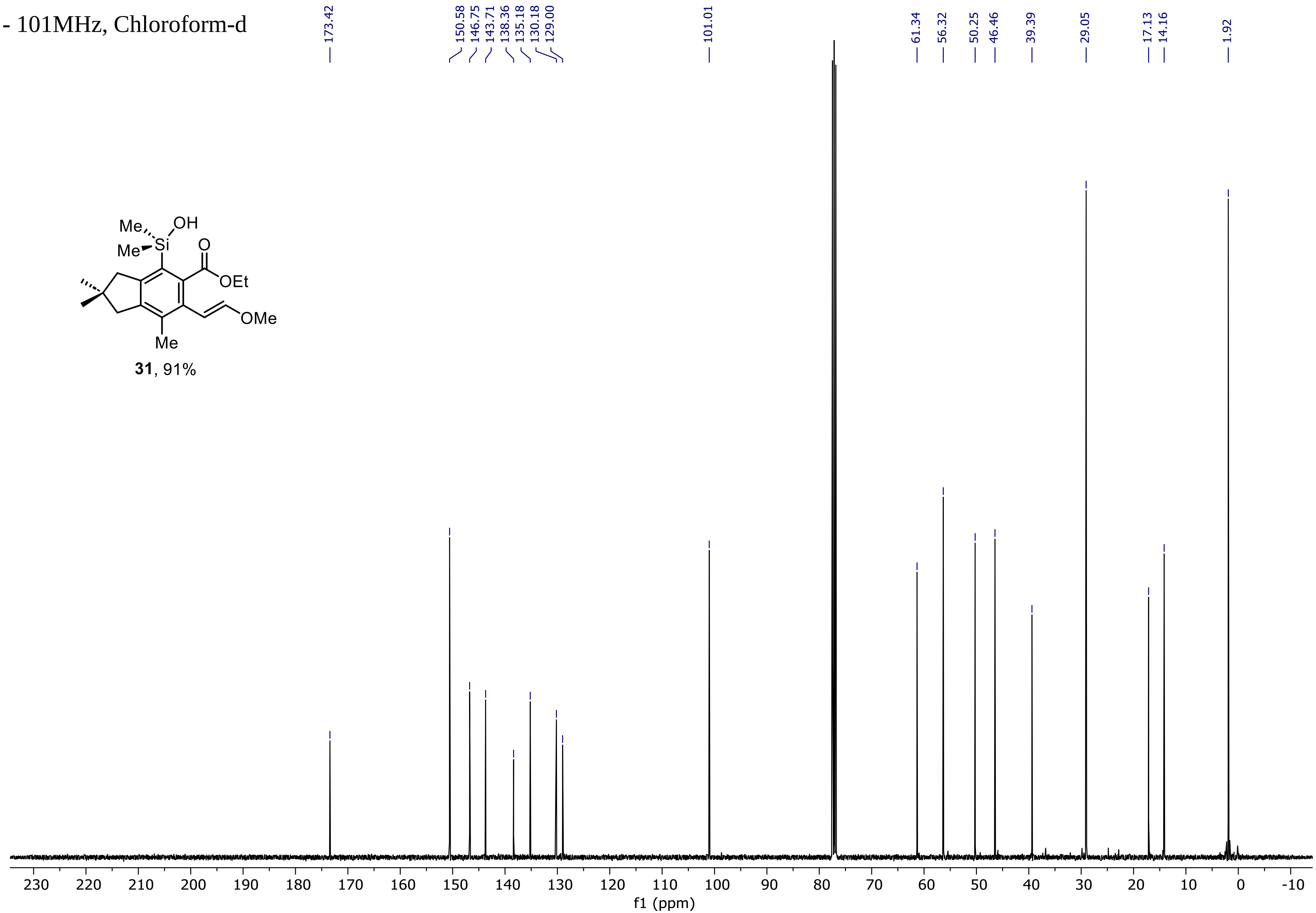

31, 91%

1D NOESY

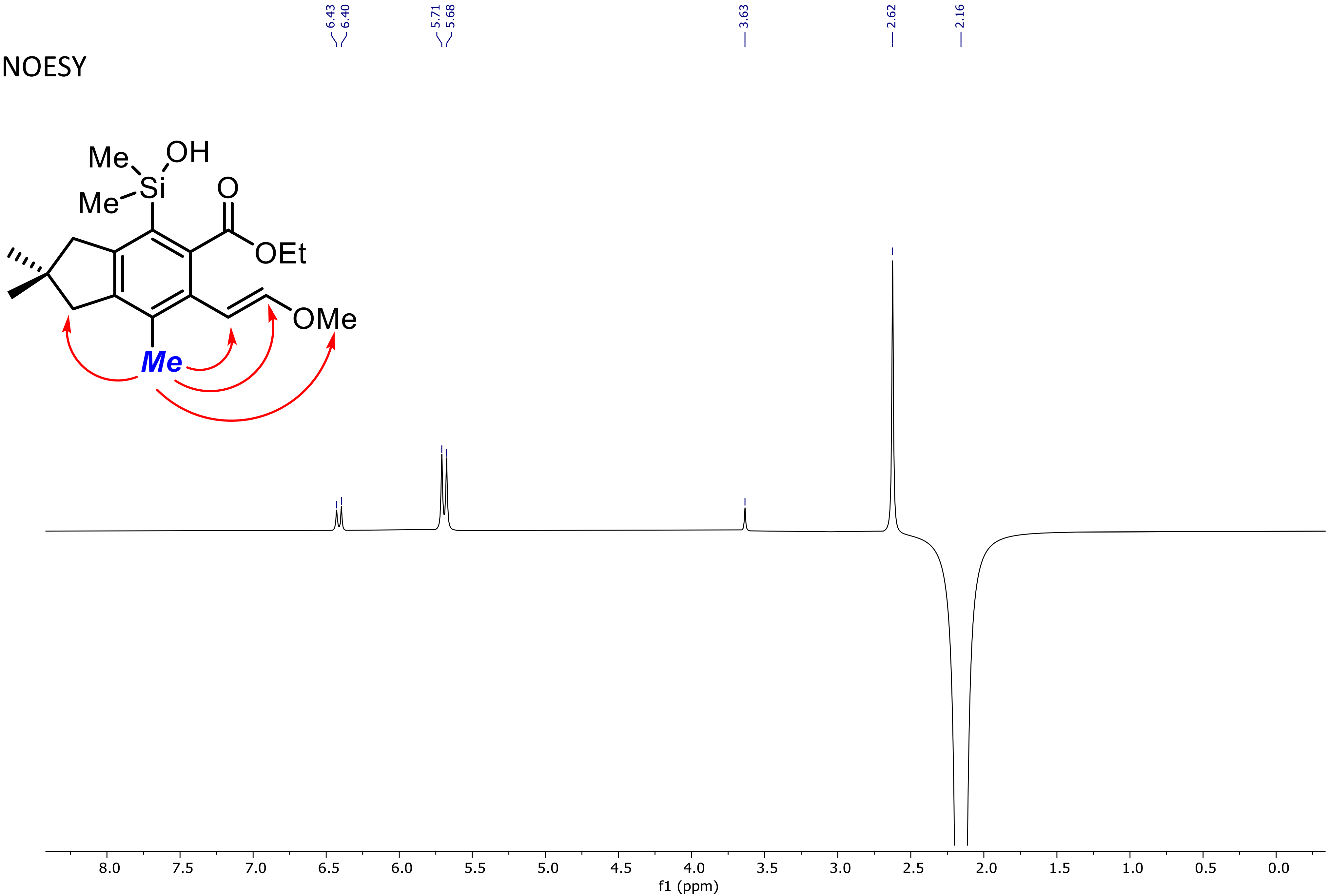
Me
OH
Si
Me
O
OEt
OMe
Me
6.43
6.40
5.71
5.68
3.63
2.62
2.16
8.0
7.5
7.0
6.5
6.0
5.5
5.0
4.5
4.0
3.5
3.0
2.5
2.0
1.5
1.0
0.5
0.0
f1 (ppm)

1H NMR - 400 MHz, Chloroform-d

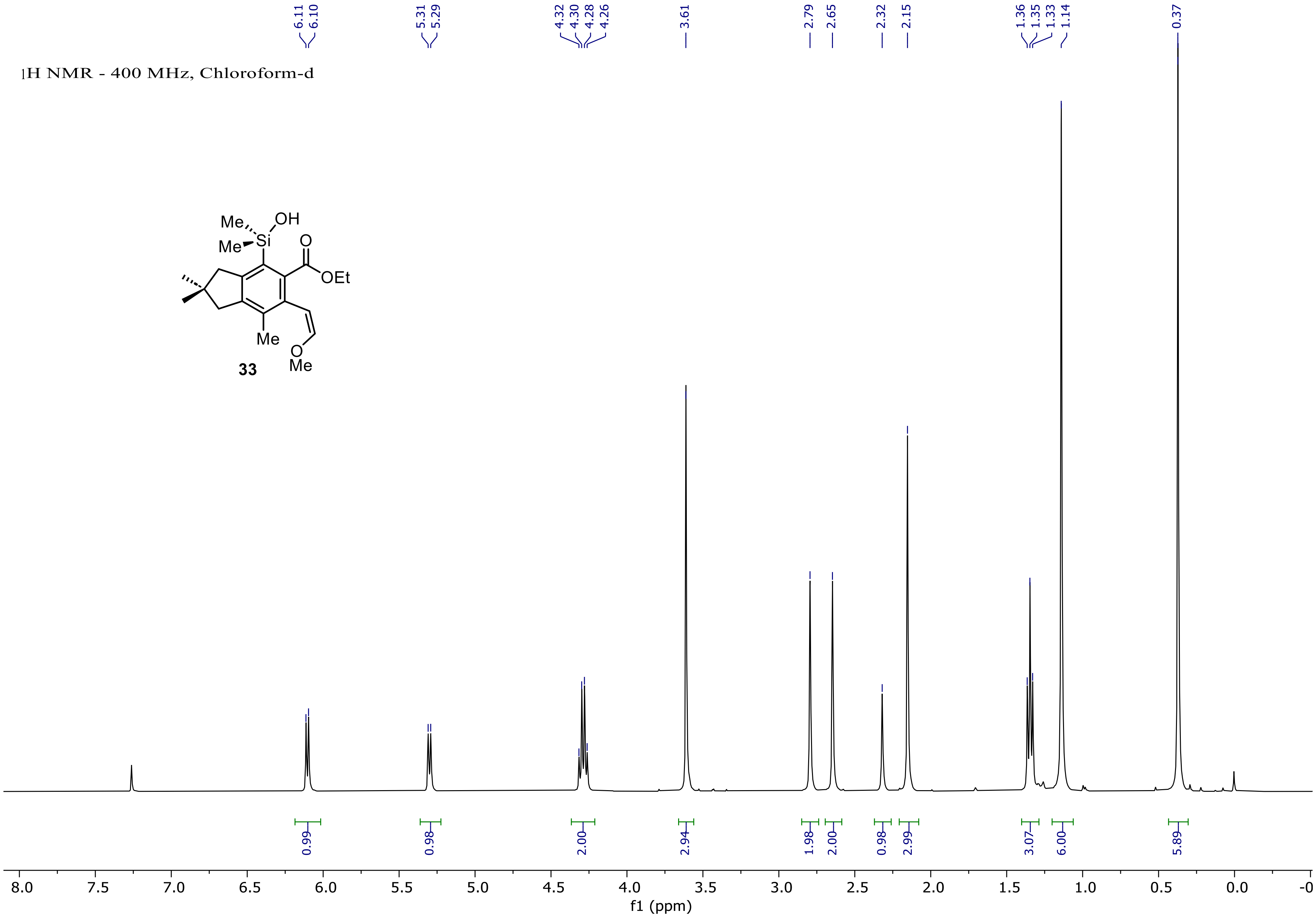

13C{1H} NMR - 101MHz, Chloroform-d

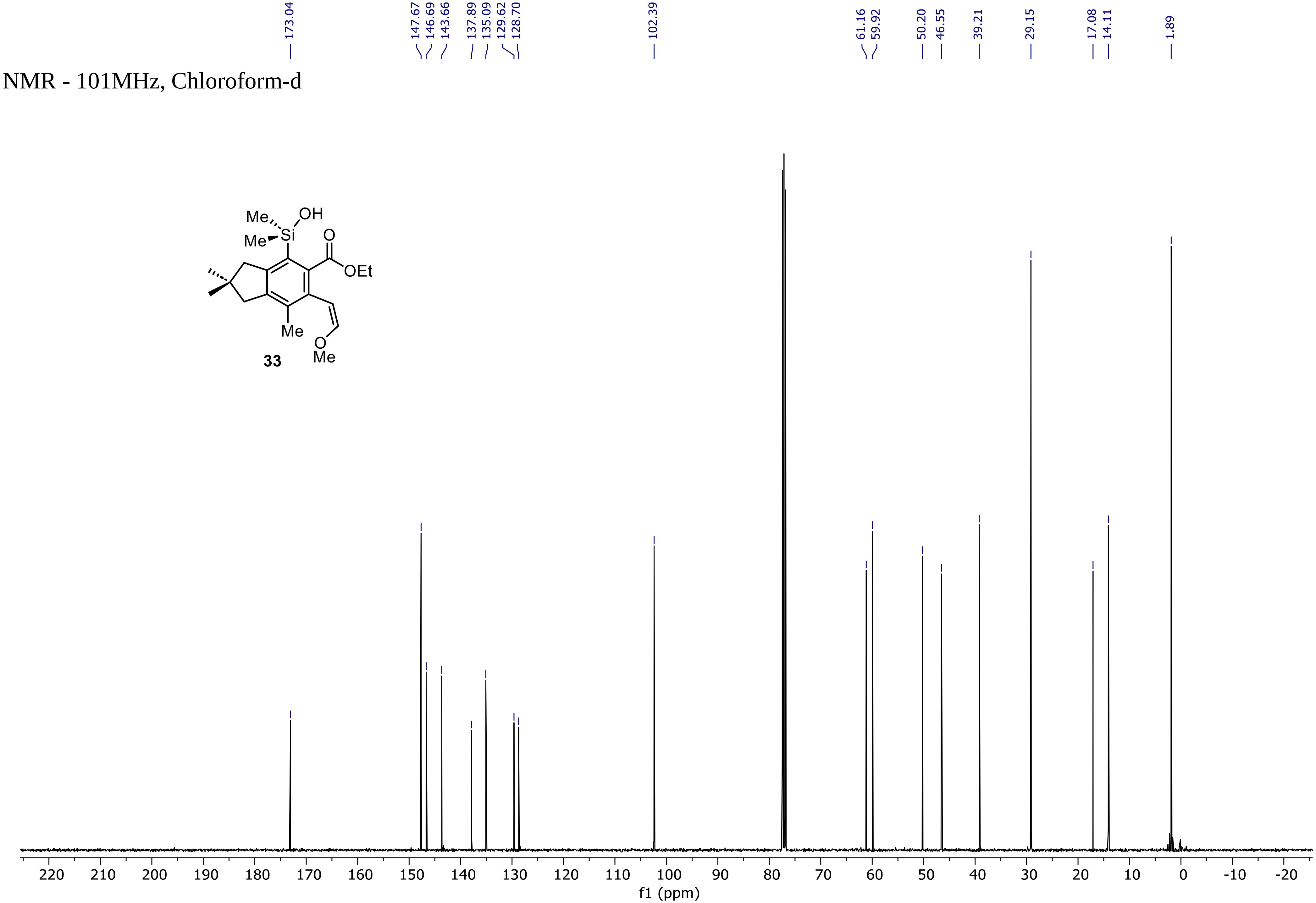

1D NOESY

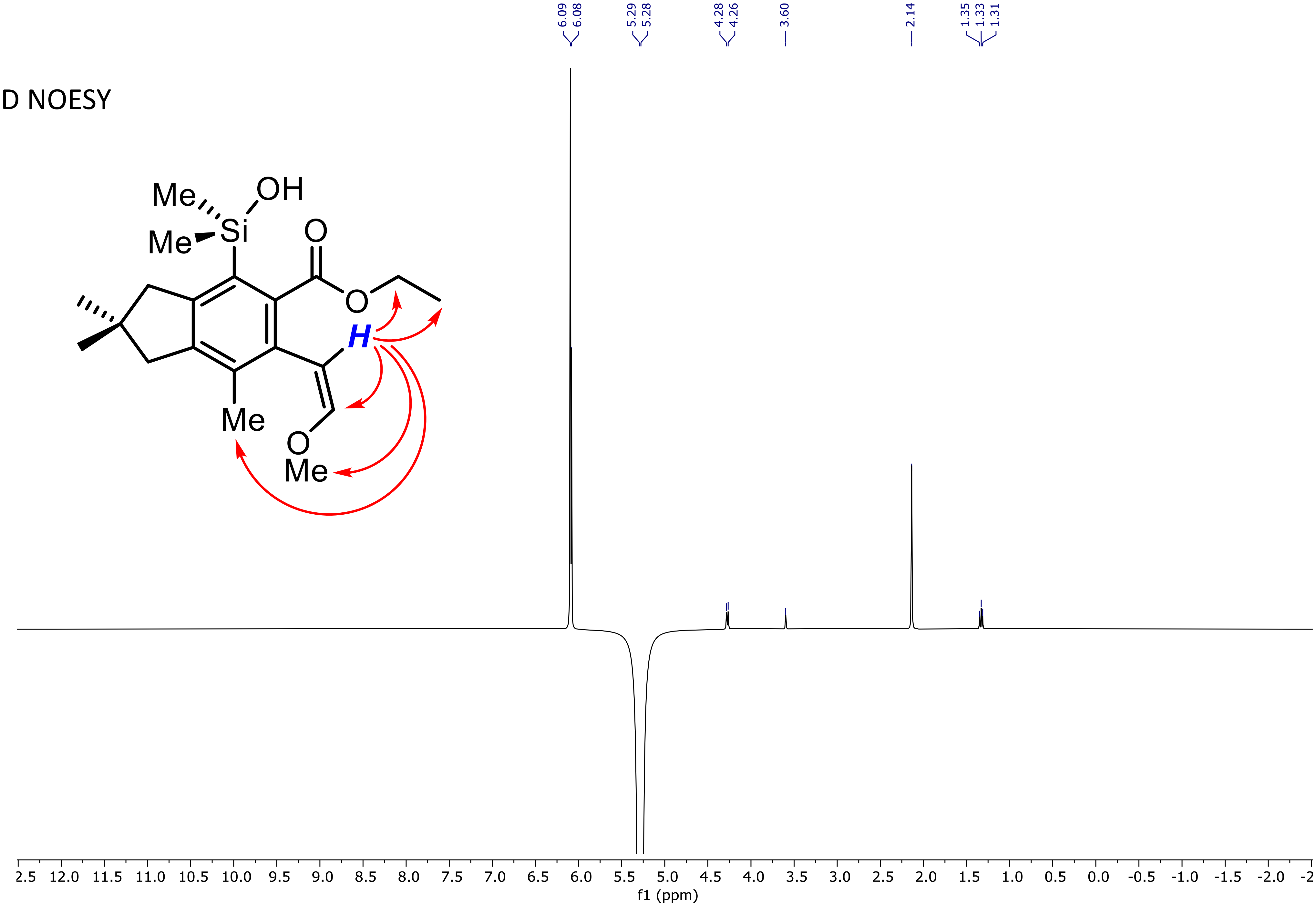

6.09
6.08
5.29
5.28
4.28
4.26
3.60
2.14
1.35
1.33
1.31
Me
OH
Si
Me
O
O
H
Me
O
Me
2.5 12.0 11.5 11.0 10.5 10.0 9.5 9.0 8.5 8.0 7.5 7.0 6.5 6.0 5.5 5.0 4.5 4.0 3.5 3.0 2.5 2.0 1.5 1.0 0.5 0.0 -0.5 -1.0 -1.5 -2.0 -2
f1 (ppm)

1D NOESY

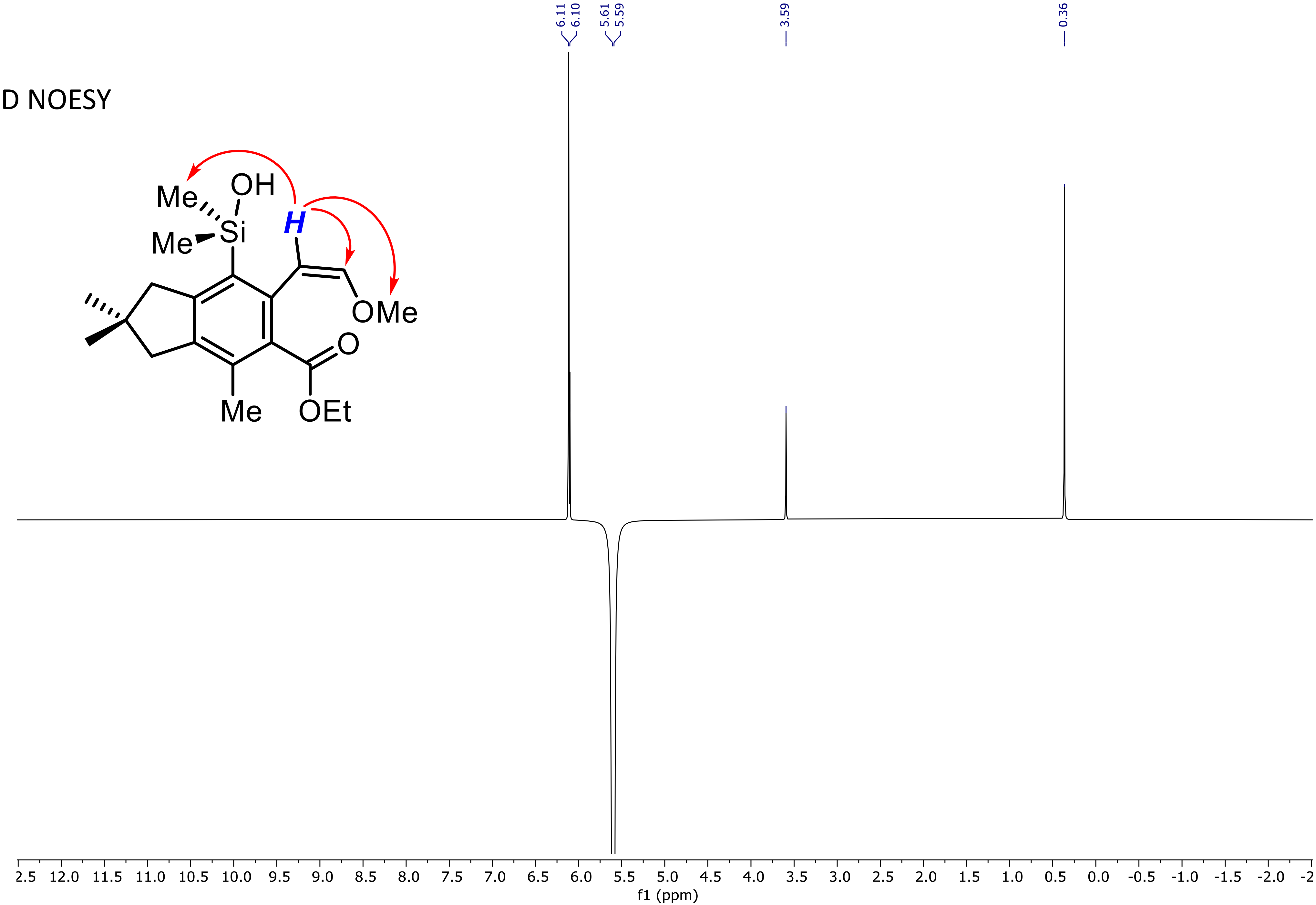

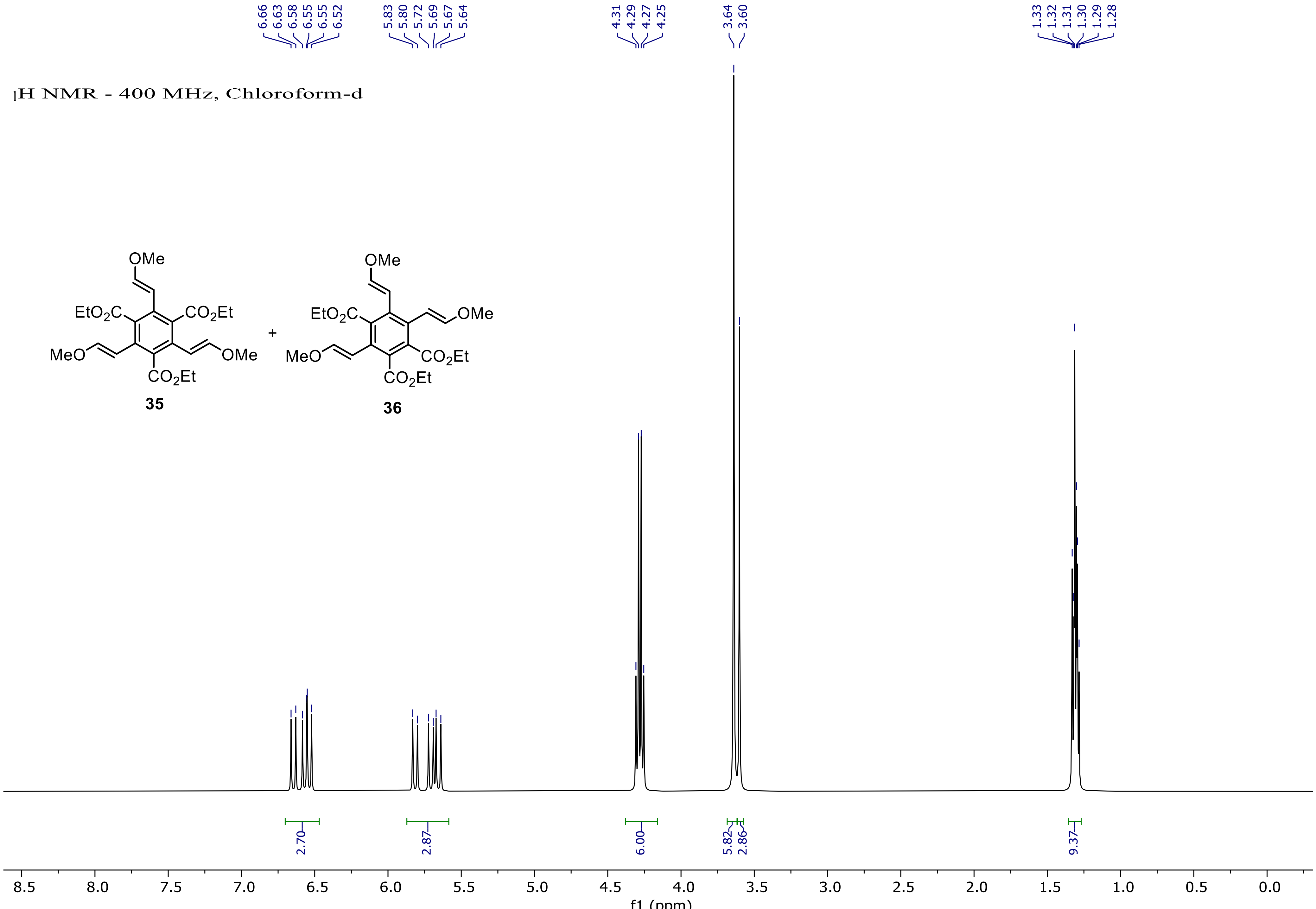
1H NMR - 400 MHz, Chloroform-d
6.66 6.63 6.58 6.55 6.55 6.52
5.83 5.80 5.72 5.69 5.67 5.64
4.31 4.29 4.27 4.25
3.64 3.60
1.33 1.32 1.31 1.30 1.29 1.28
OMe
EtO2C
CO2Et
MeO
OMe
CO2Et
35
+
OMe
EtO2C
OMe
MeO
CO2Et
CO2Et
36
2.70
2.87
6.00
5.82
2.86
9.37
8.5 8.0 7.5 7.0 6.5 6.0 5.5 5.0 4.5 4.0 3.5 3.0 2.5 2.0 1.5 1.0 0.5 0.0
f1 (ppm)

13C{1H} NMR - 101MHz, Chloroform-d

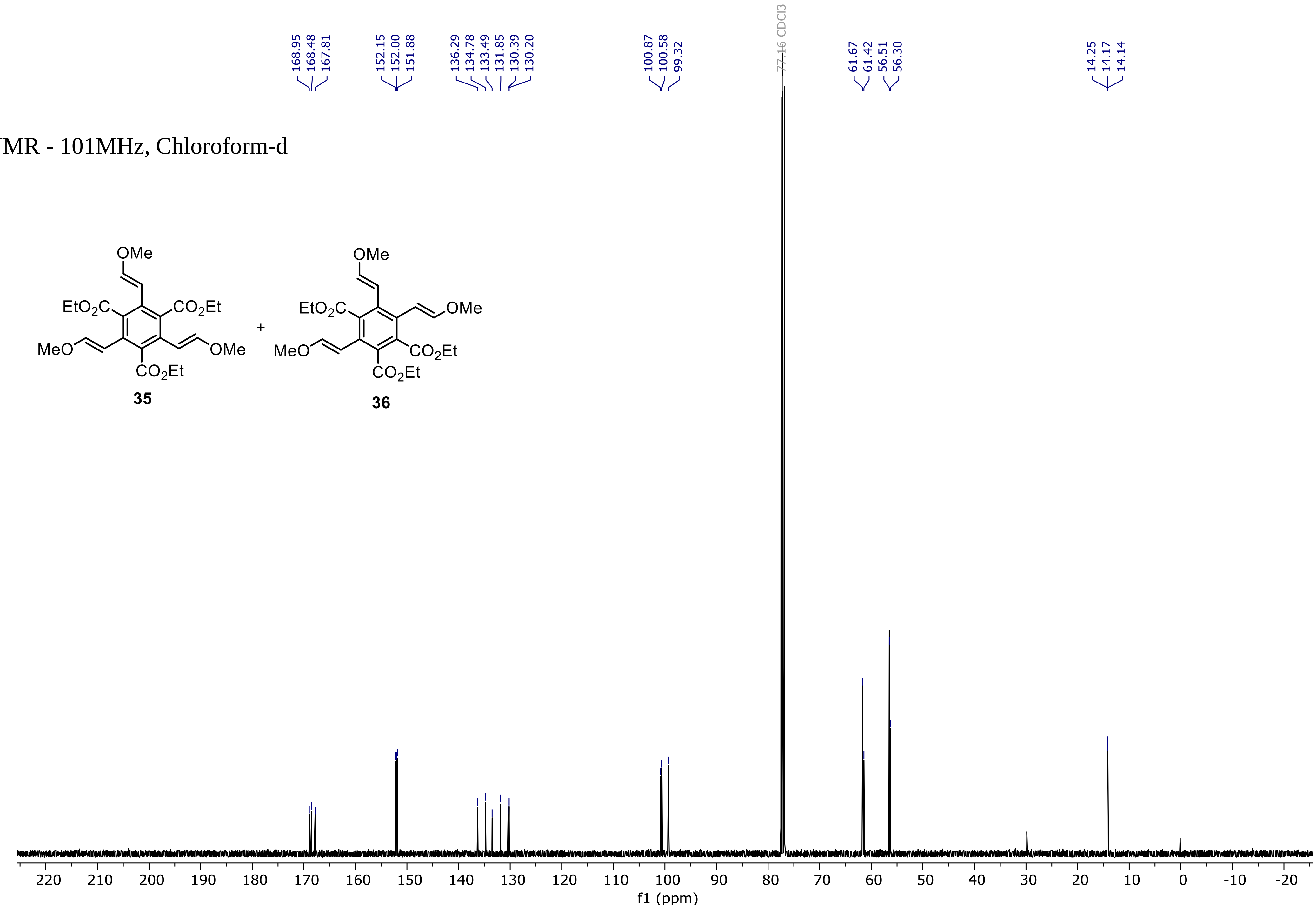

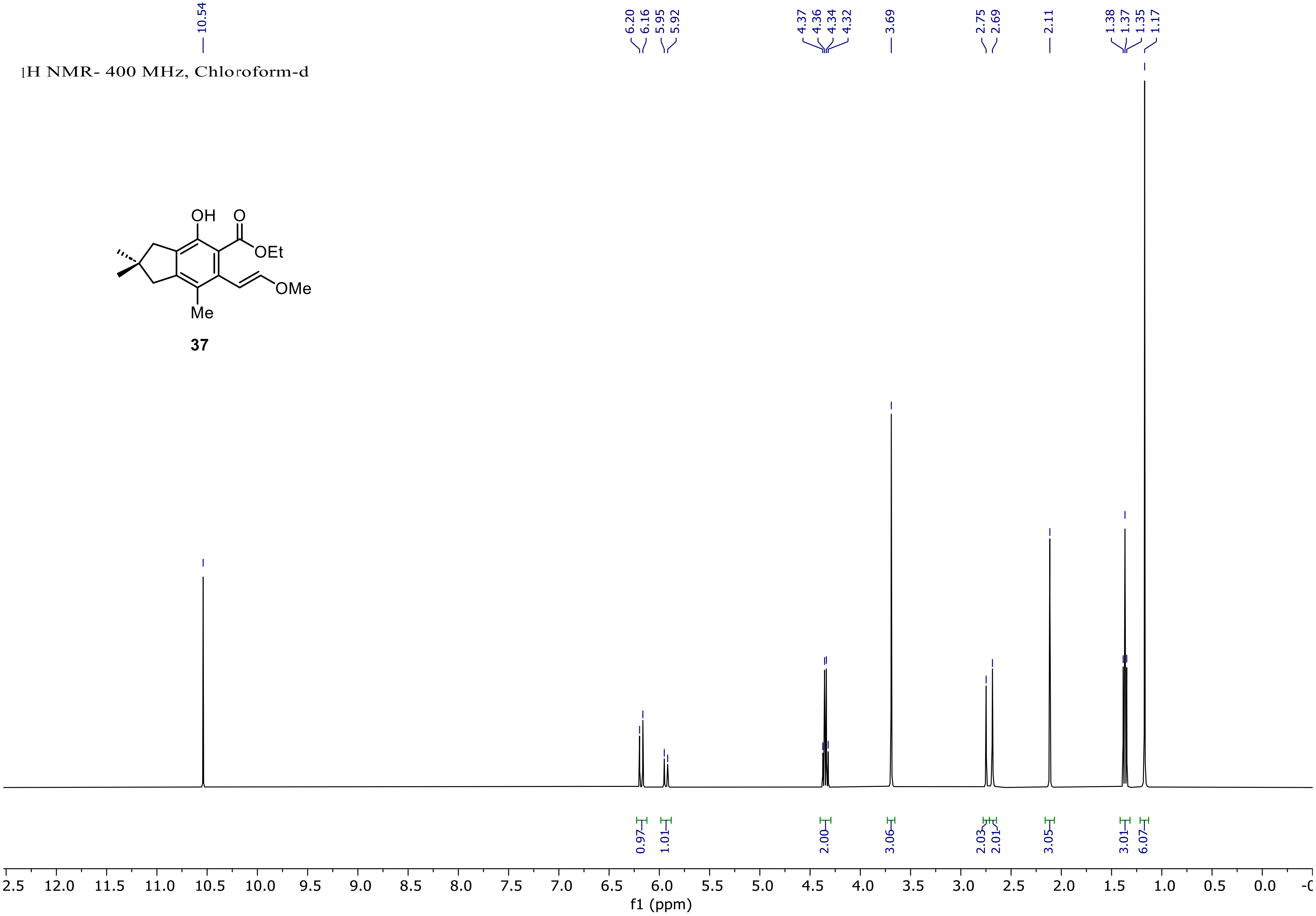

1H NMR- 400 MHz, Chloroform-d
10.54
6.20
6.16
5.95
5.92
4.37
4.36
4.34
4.32
3.69
2.75
2.69
2.11
1.38
1.37
1.35
1.17
OH
O
OEt
OMe
Me
37
0.97
1.01
2.00
3.06
2.03
2.01
3.05
3.01
6.07
f1 (ppm)

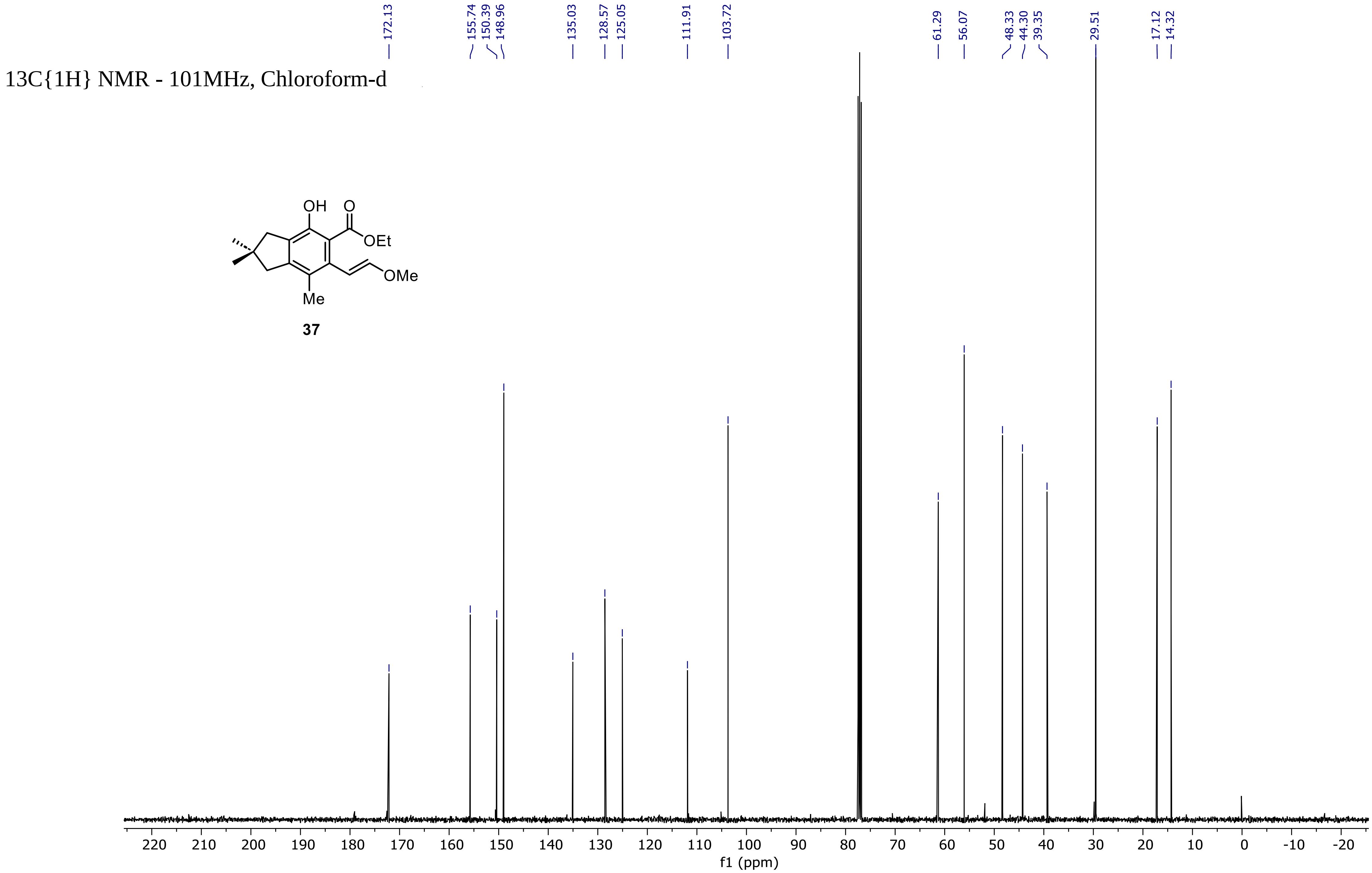
13C{1H} NMR - 101MHz, Chloroform-d
172.13
155.74
150.39
148.96
135.03
128.57
125.05
111.91
103.72
61.29
56.07
48.33
44.30
39.35
29.51
17.12
14.32
OH
O
OEt
OMe
Me
37
220
210
200
190
180
170
160
150
140
130
120
110
100
90
80
70
60
50
40
30
20
10
0
-10
-20
f1 (ppm)

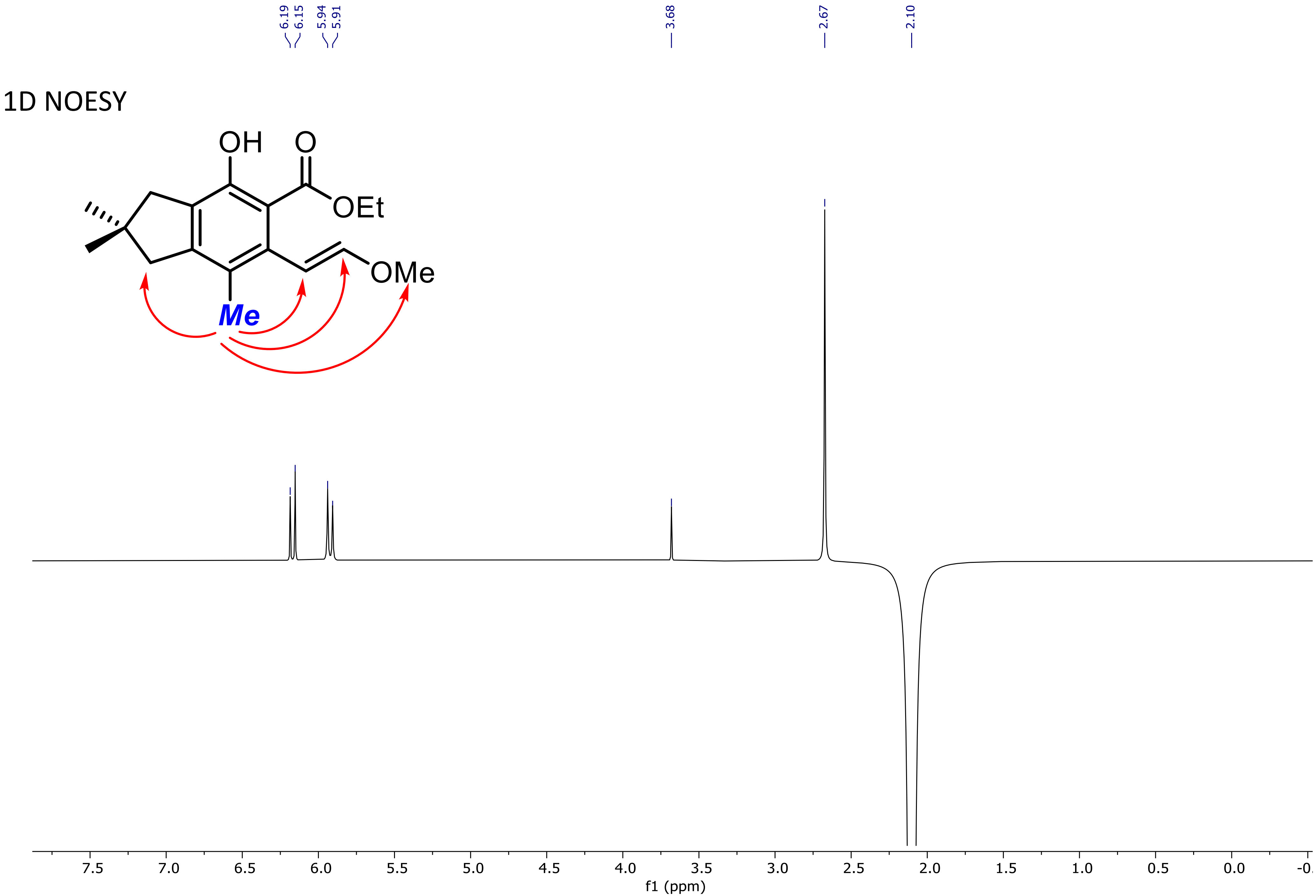
6.19
6.15
5.94
5.91
3.68
2.67
2.10
1D NOESY
OH
O
OEt
OMe
Me
7.5
7.0
6.5
6.0
5.5
5.0
4.5
4.0
3.5
3.0
2.5
2.0
1.5
1.0
0.5
0.0
f1 (ppm)

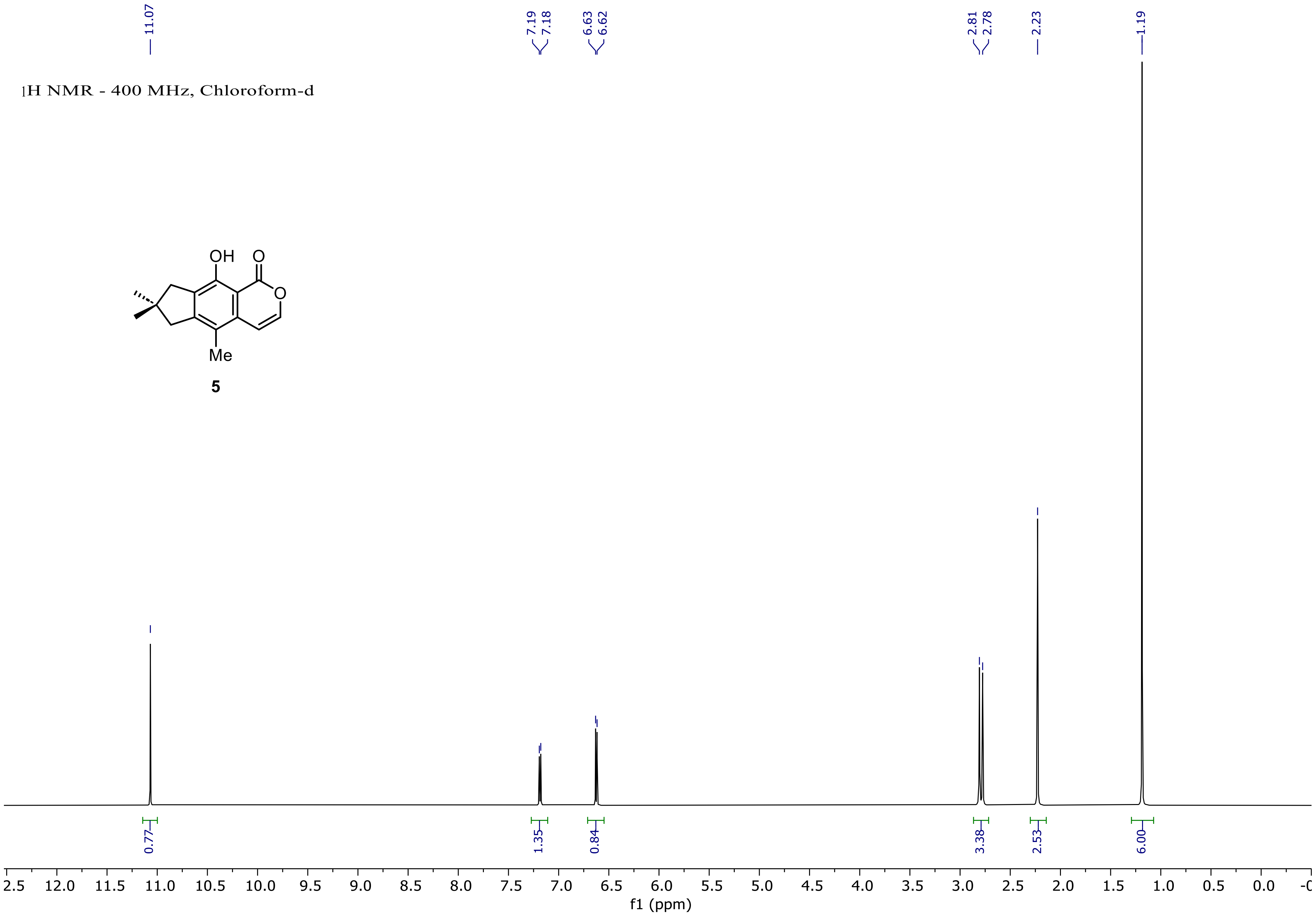
1H NMR - 400 MHz, Chloroform-d
11.07
7.19
7.18
6.63
6.62
2.81
2.78
2.23
1.19
OH
O
O
Me
5
0.77
1.35
0.84
3.38
2.53
6.00
f1 (ppm)

13C{1H} NMR - 101MHz, Chloroform-d

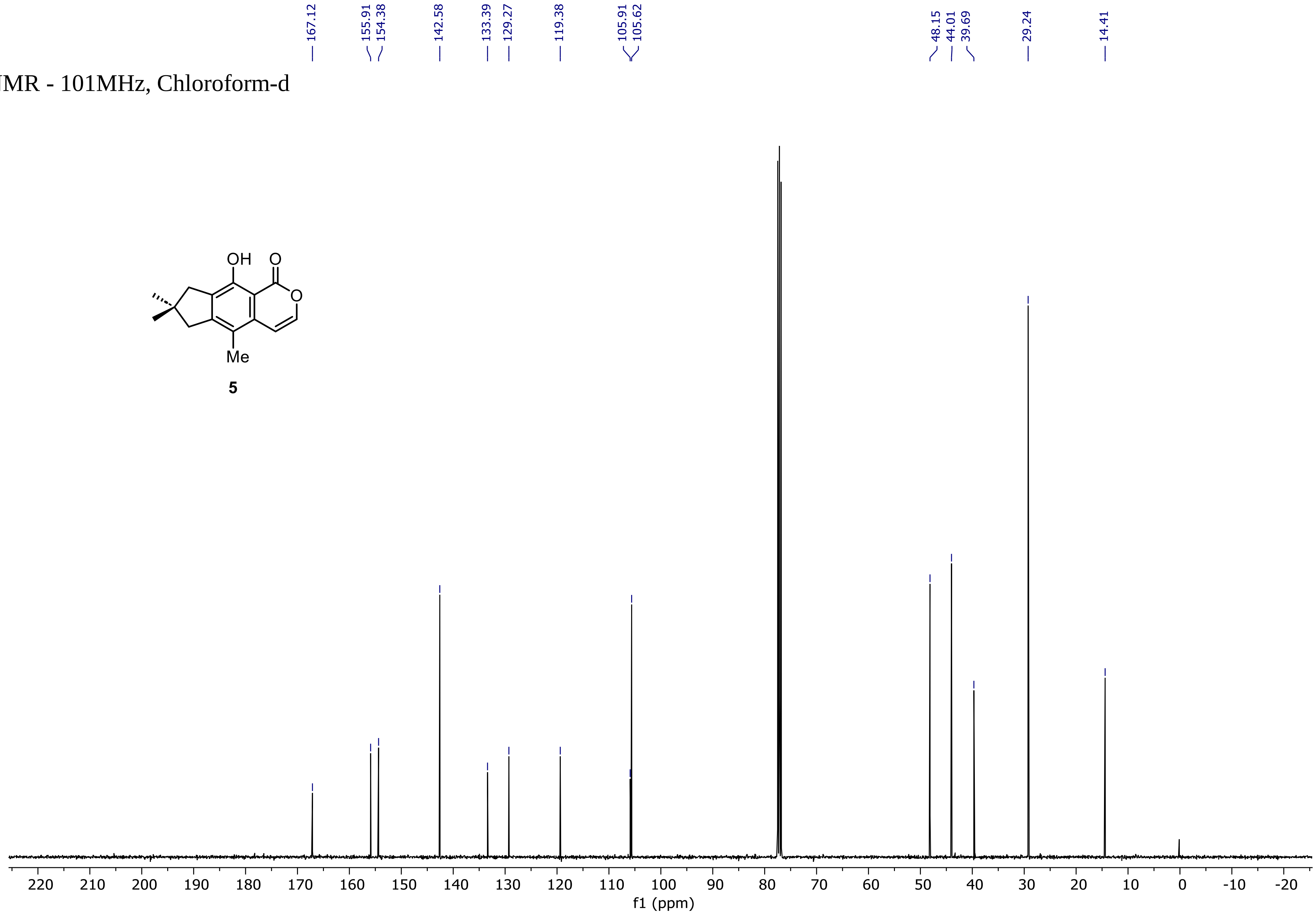

1D NOESY

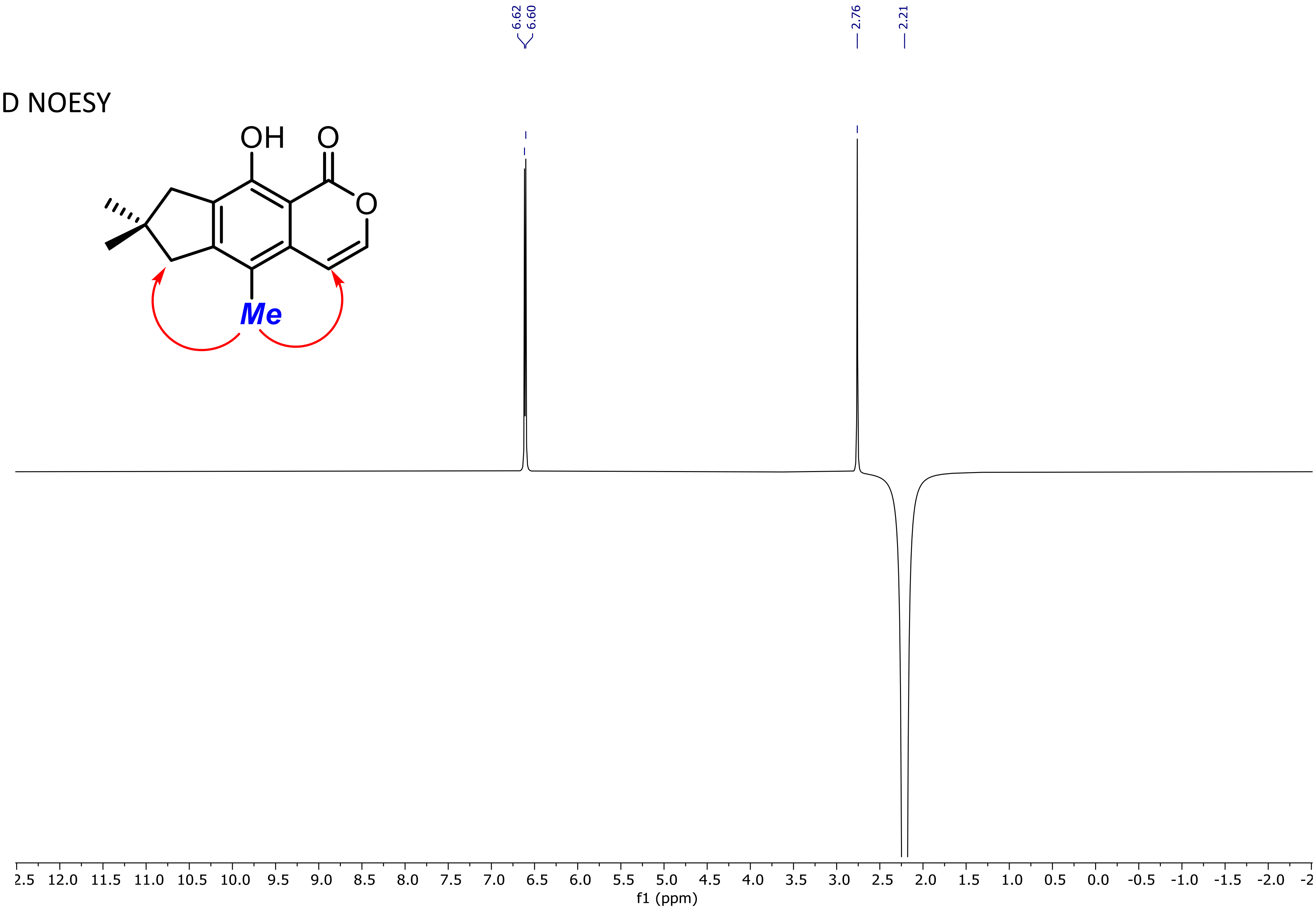

OH
O
O
Me
6.62
6.60
2.76
2.21
2.5 12.0 11.5 11.0 10.5 10.0 9.5 9.0 8.5 8.0 7.5 7.0 6.5 6.0 5.5 5.0 4.5 4.0 3.5 3.0 2.5 2.0 1.5 1.0 0.5 0.0 -0.5 -1.0 -1.5 -2.0 -2
f1 (ppm)

1H NMR - 400 MHz, Chloroform-d

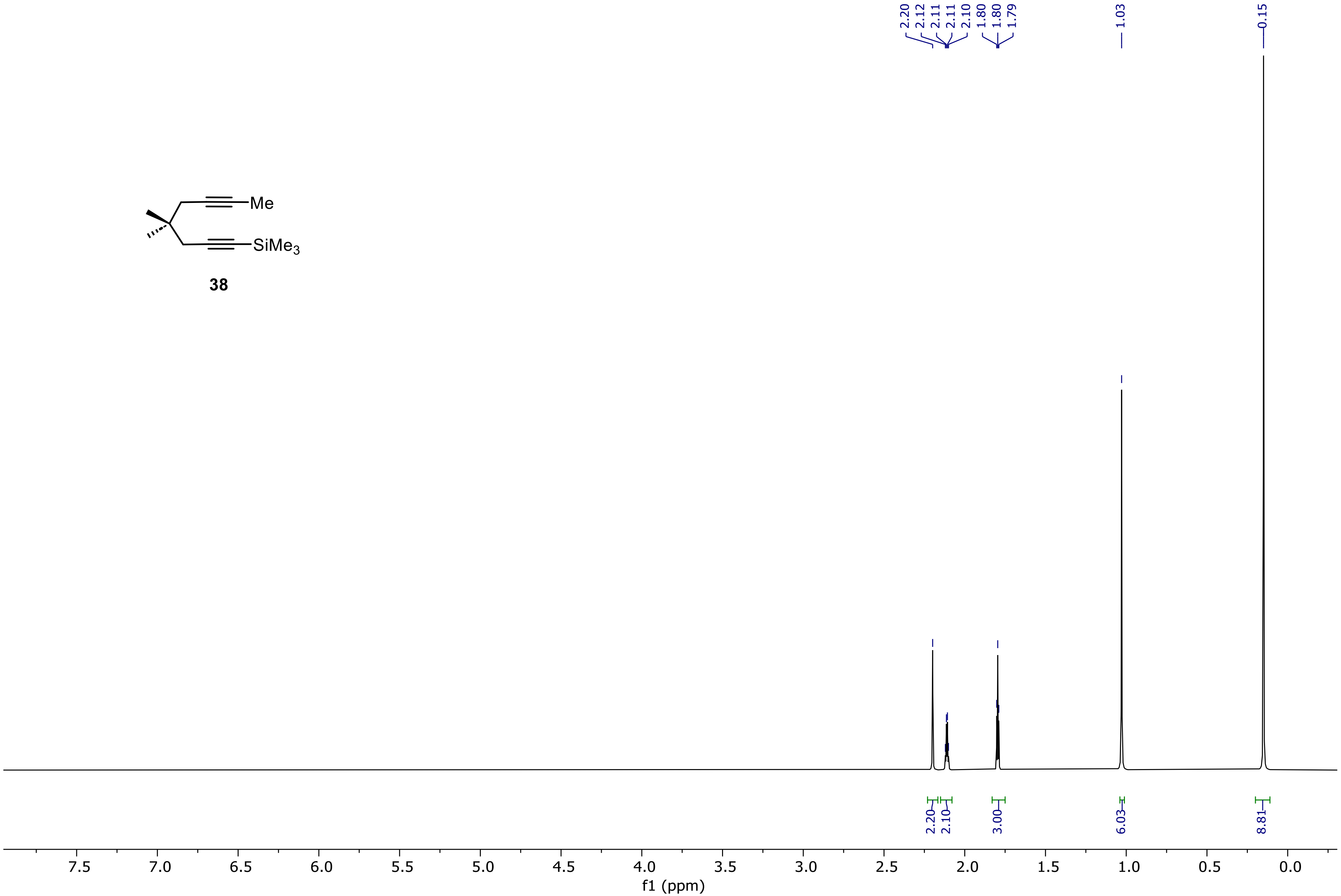

13C{1H} NMR - 101MHz, Chloroform-d

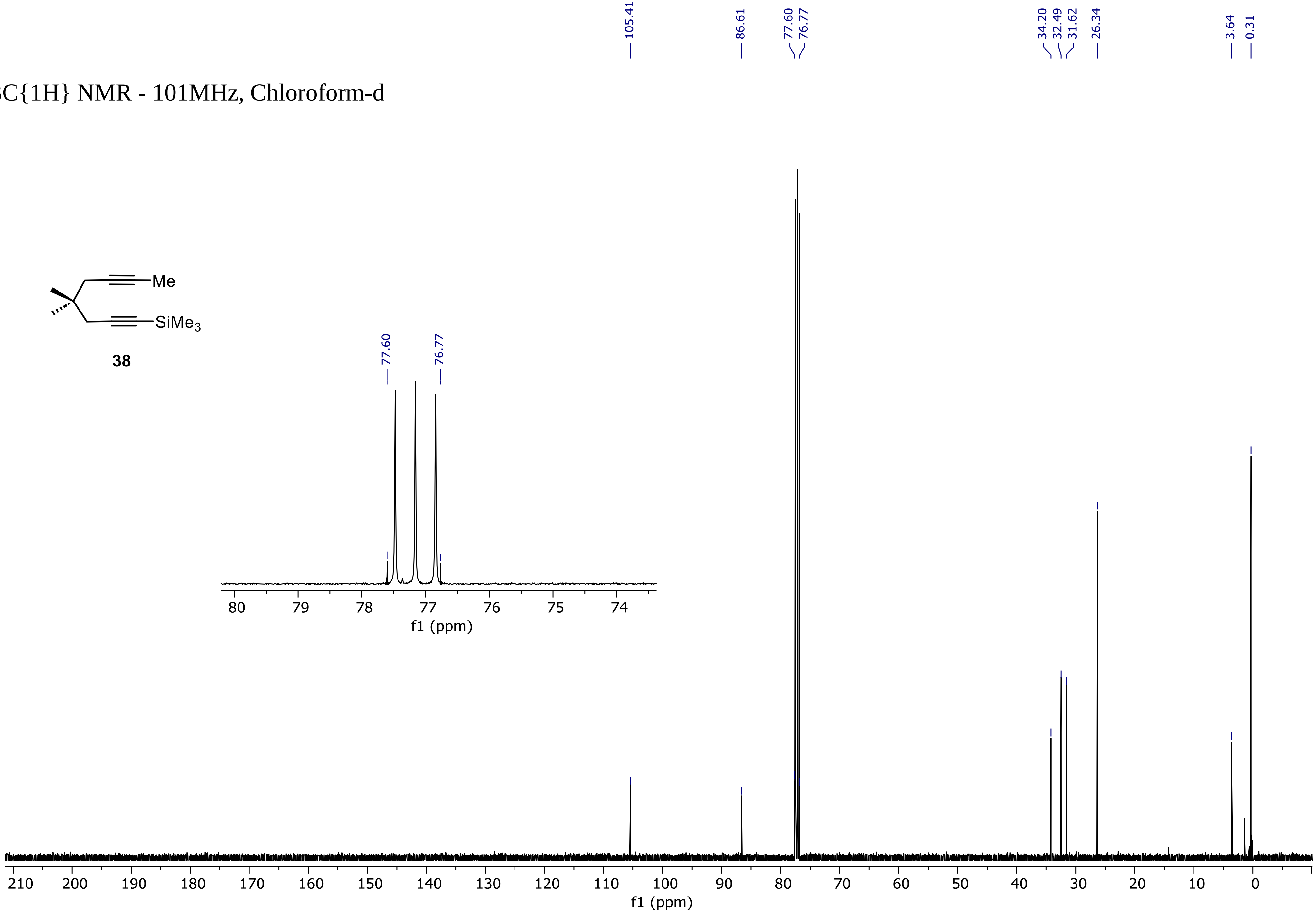

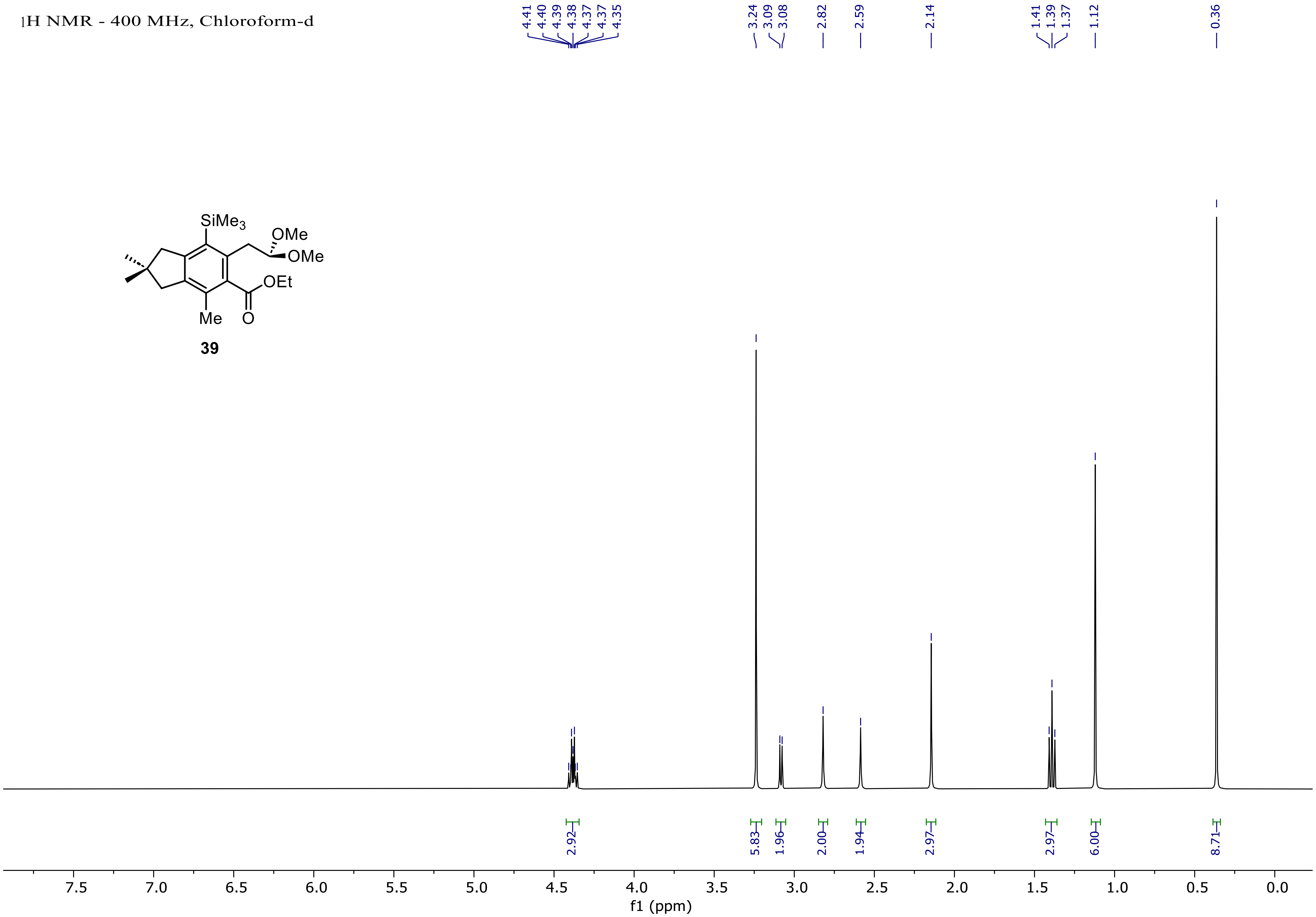
1H NMR - 400 MHz, Chloroform-d
4.41 4.40 4.39 4.38 4.37 4.37 4.35
3.24 3.09 3.08
2.82
2.59
2.14
1.41 1.39 1.37
1.12
0.36
SiMe3
OMe
OMe
OEt
Me
O
39
2.92
5.83
1.96
2.00
1.94
2.97
2.97
6.00
8.71
7.5 7.0 6.5 6.0 5.5 5.0 4.5 4.0 3.5 3.0 2.5 2.0 1.5 1.0 0.5 0.0
f1 (ppm)

13C{1H} NMR - 101MHz, Chloroform-d

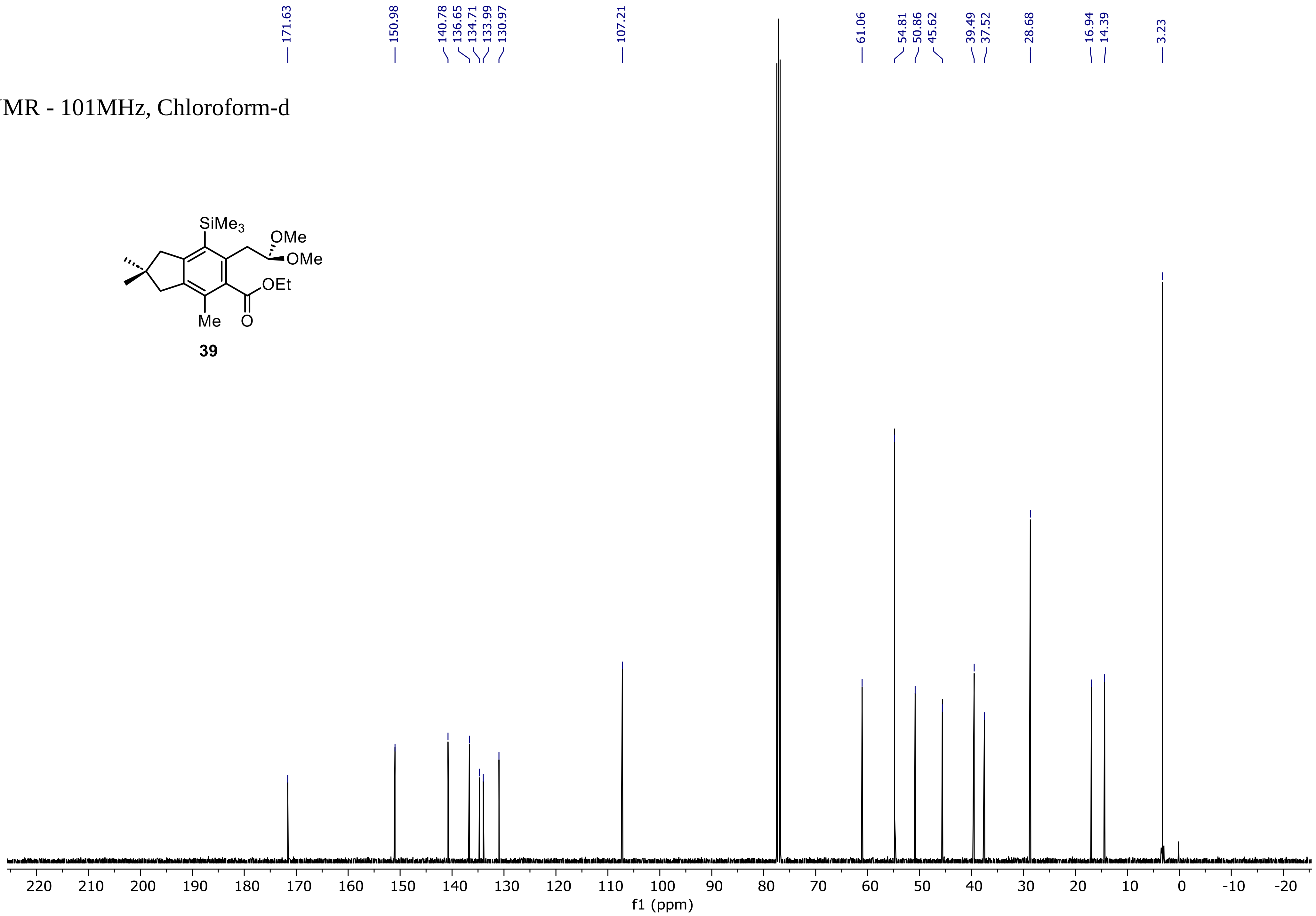

1D NOESY

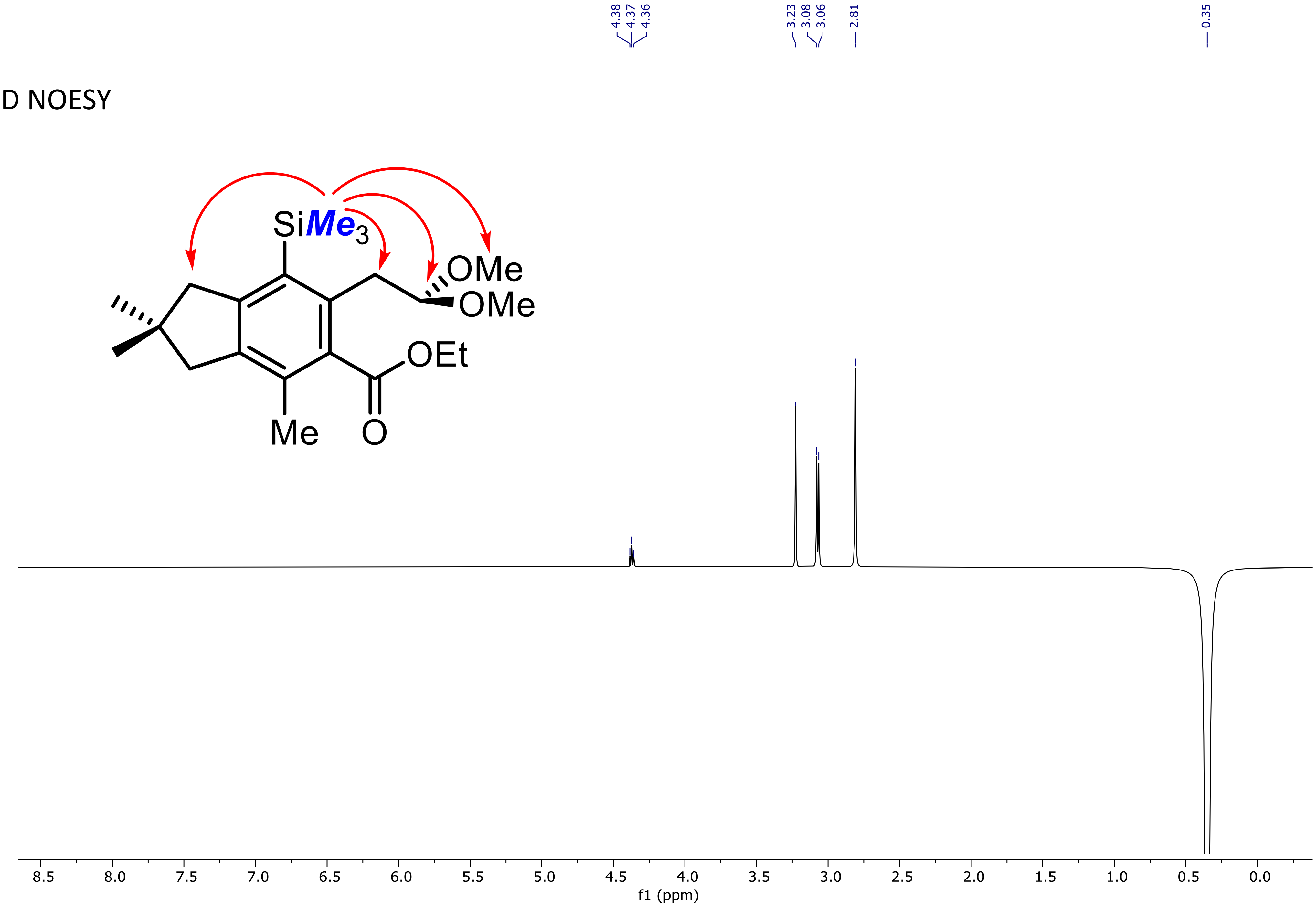
SiMe3
OMe
OMe
OEt
Me
O
4.38
4.37
4.36
3.23
3.08
3.06
2.81
0.35
8.5 8.0 7.5 7.0 6.5 6.0 5.5 5.0 4.5 4.0 3.5 3.0 2.5 2.0 1.5 1.0 0.5 0.0
f1 (ppm)

1H NMR - 400 MHz, Chloroform-d

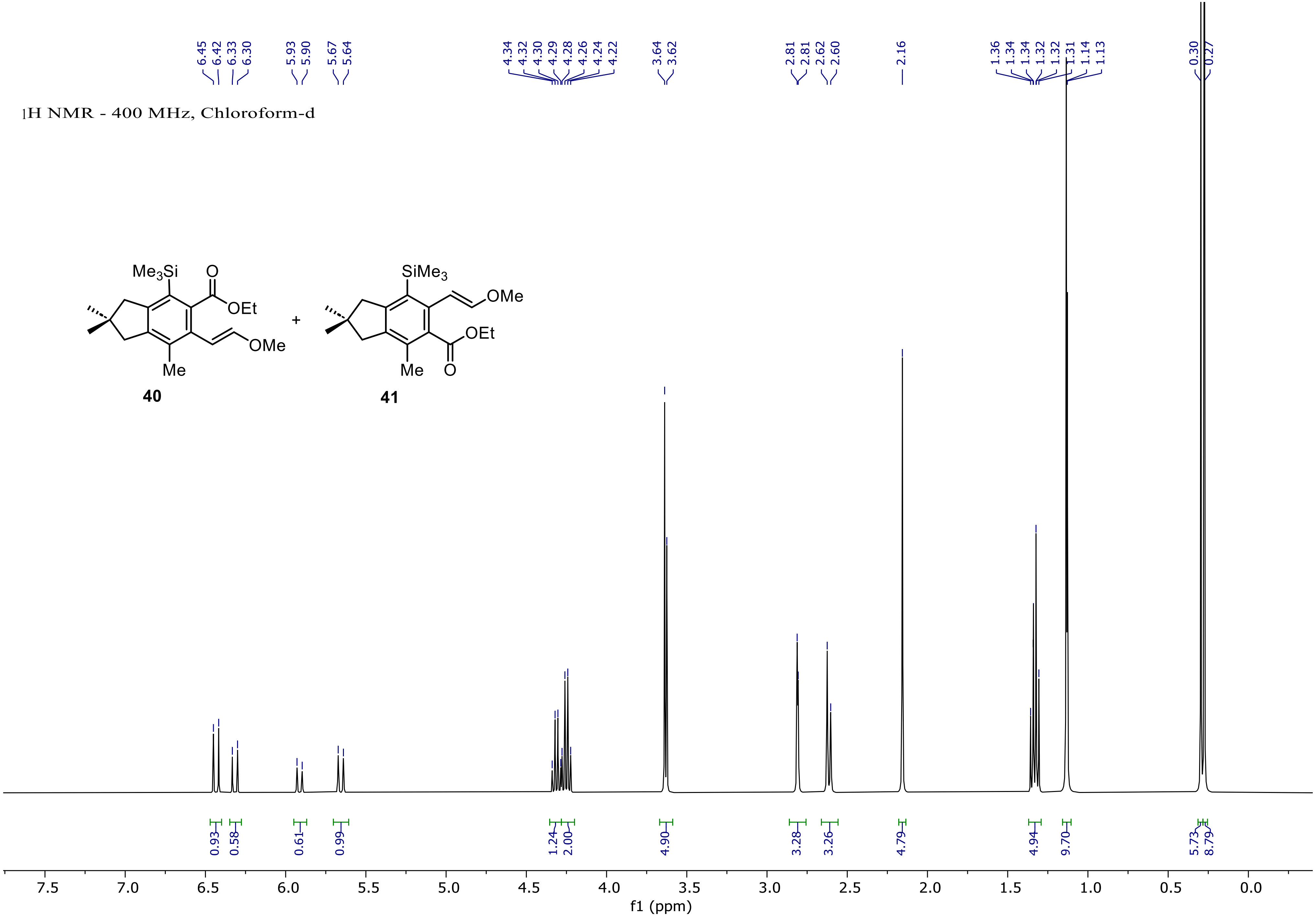

13C{1H} NMR - 101MHz, Chloroform-d

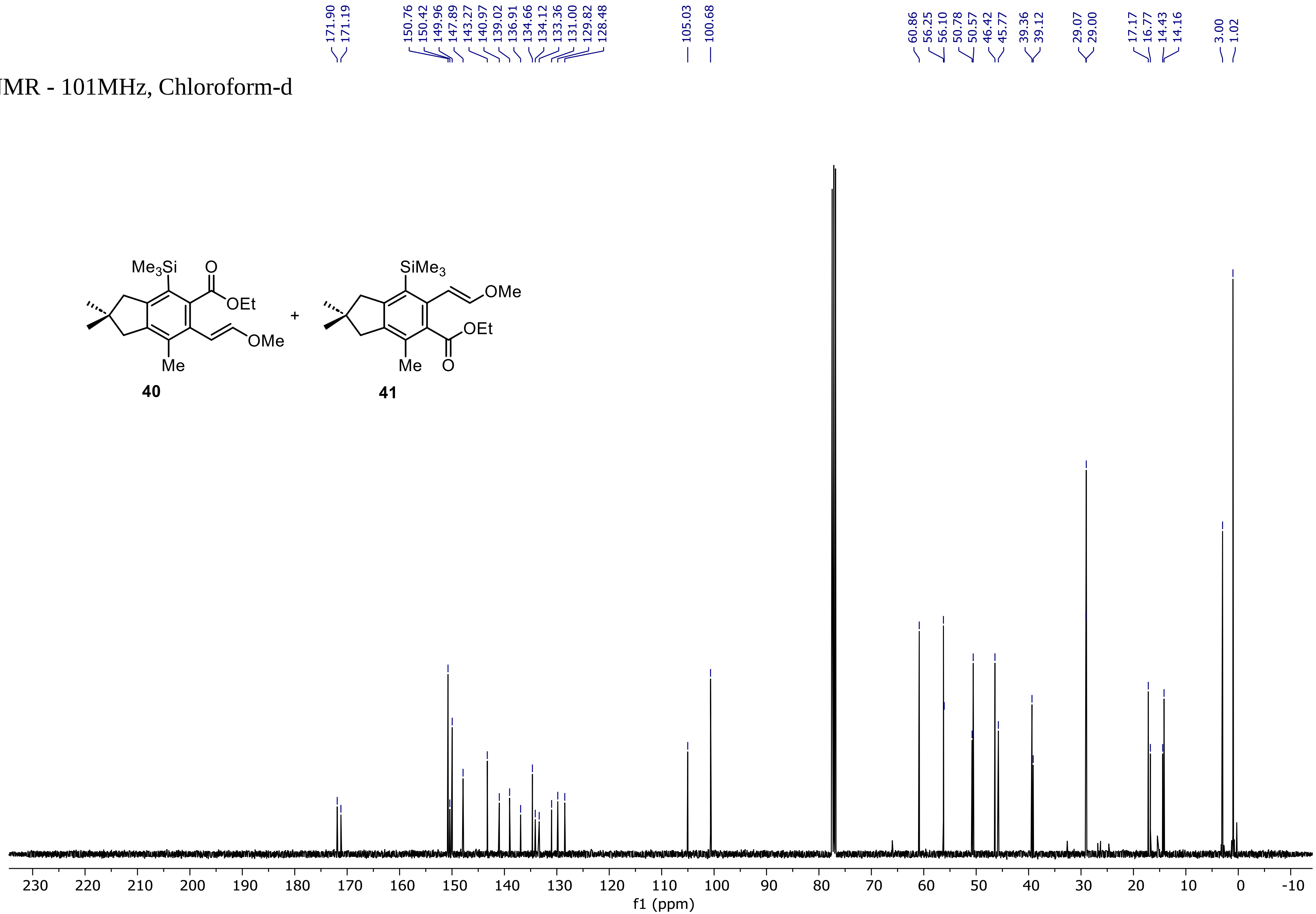

1D NOESY

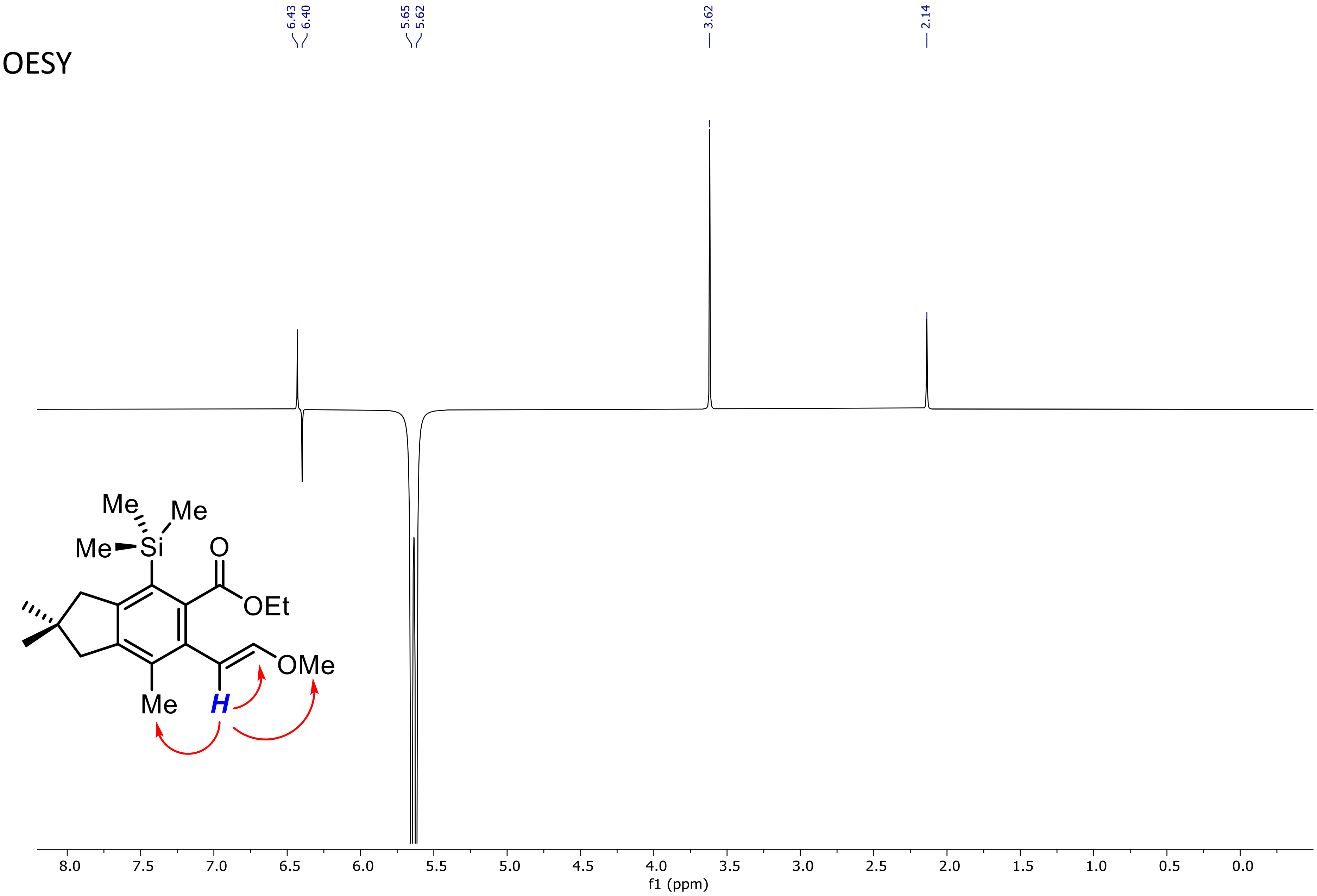
6.43
6.40
5.65
5.62
3.62
2.14
Me
Me
Me
Si
O
OEt
OMe
Me
H
8.0
7.5
7.0
6.5
6.0
5.5
5.0
4.5
4.0
3.5
3.0
2.5
2.0
1.5
1.0
0.5
0.0
f1 (ppm)

1D NOESY

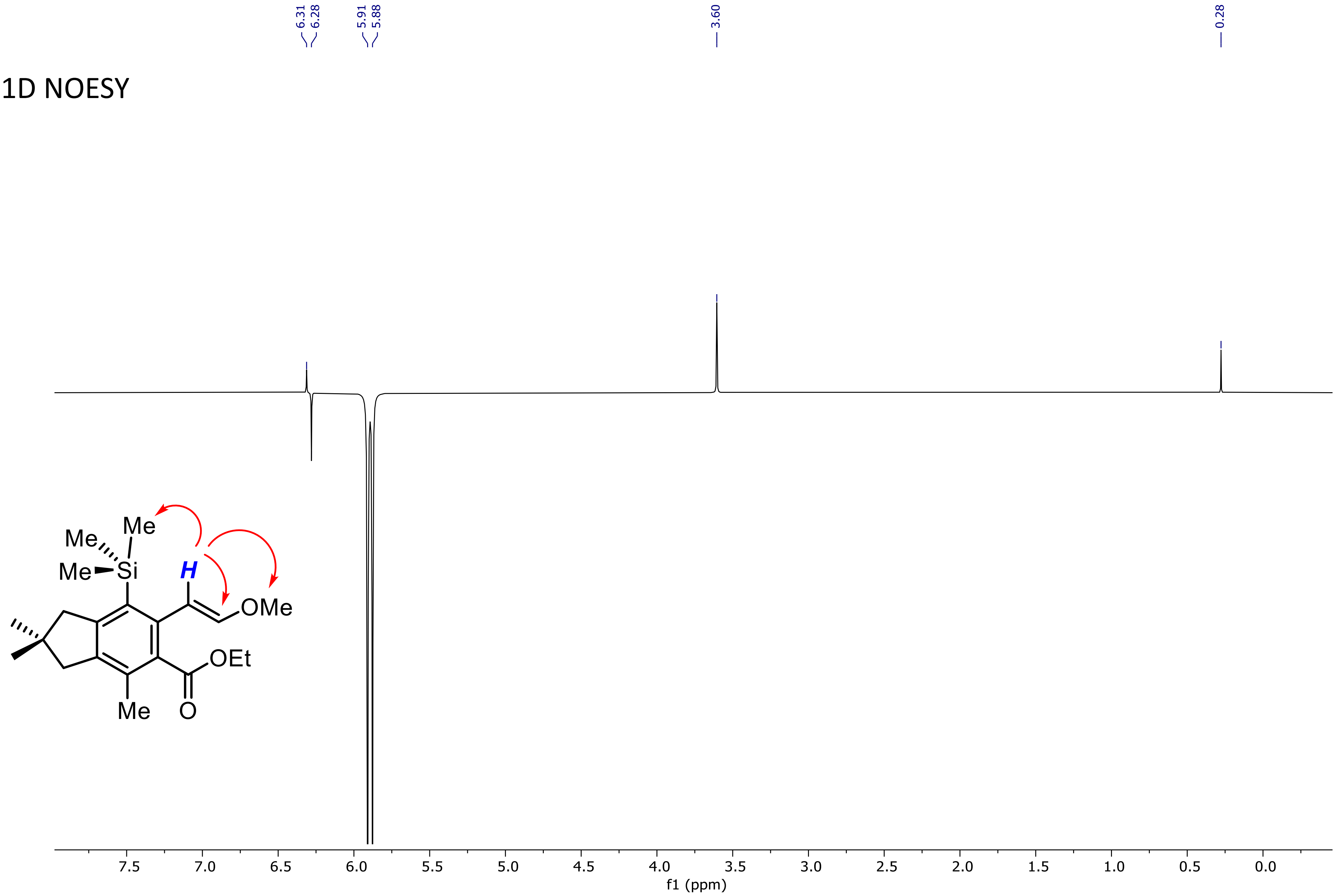

6.31
6.28
5.91
5.88
3.60
0.28
7.5
7.0
6.5
6.0
5.5
5.0
4.5
4.0
3.5
3.0
2.5
2.0
1.5
1.0
0.5
0.0
f1 (ppm)
Me
Me
Me
Si
H
OMe
OEt
Me
O

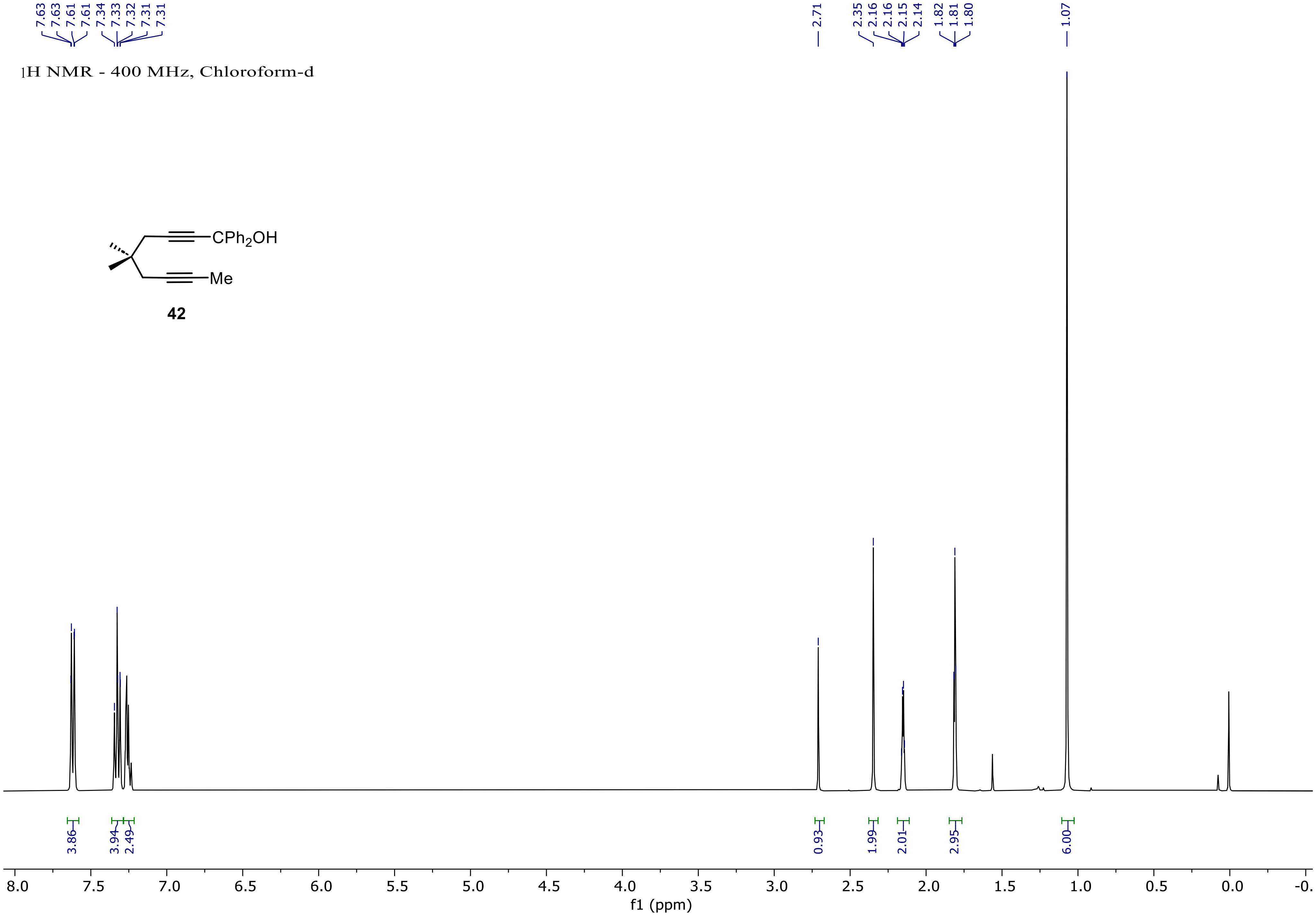
7.63
7.63
7.61
7.61
7.34
7.33
7.32
7.31
7.31
2.71
2.35
2.16
2.16
2.15
2.14
1.82
1.81
1.80
1.07
1H NMR - 400 MHz, Chloroform-d
CPh2OH
Me
42
3.86
3.94
2.49
0.93
1.99
2.01
2.95
6.00
8.0
7.5
7.0
6.5
6.0
5.5
5.0
4.5
4.0
3.5
3.0
2.5
2.0
1.5
1.0
0.5
0.0
-0.
f1 (ppm)

13C{1H} NMR - 101MHz, Chloroform-d

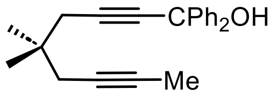

42

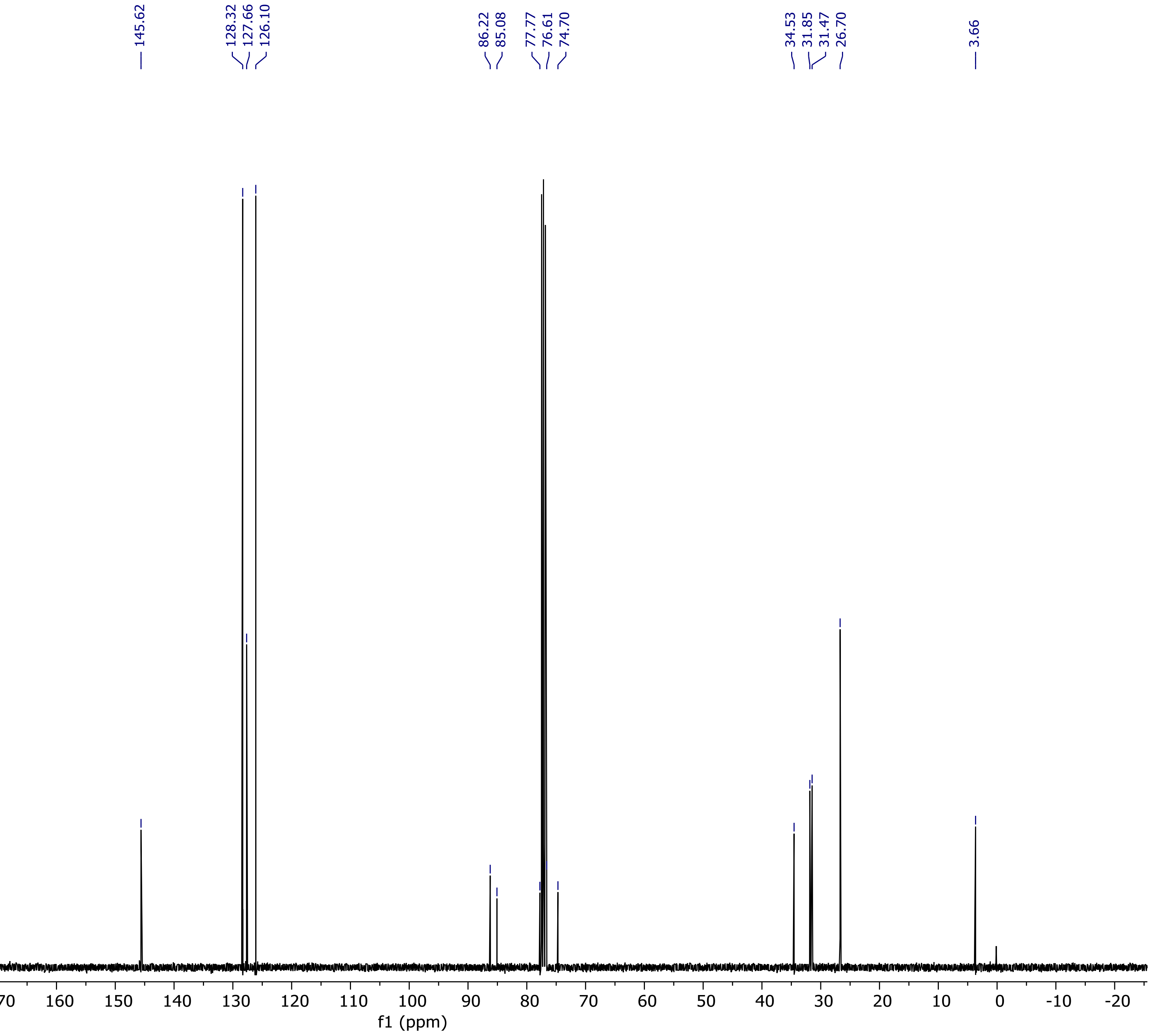

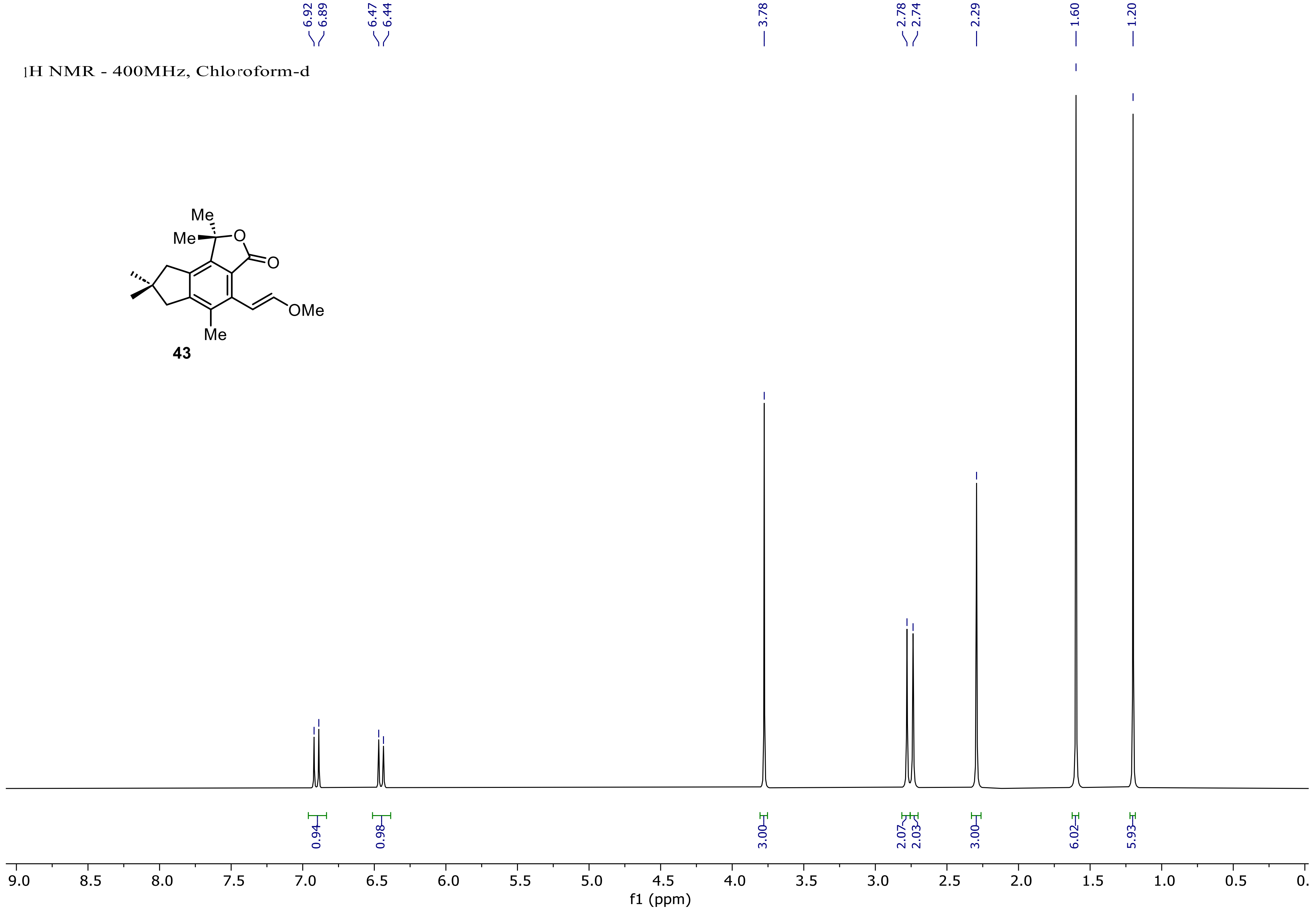
1H NMR - 400MHz, Chloroform-d
6.92
6.89
6.47
6.44
3.78
2.78
2.74
2.29
1.60
1.20
Me
Me
O
O
OMe
Me
43
0.94
0.98
3.00
2.07
2.03
3.00
6.02
5.93
9.0 8.5 8.0 7.5 7.0 6.5 6.0 5.5 5.0 4.5 4.0 3.5 3.0 2.5 2.0 1.5 1.0 0.5 0.
f1 (ppm)

13C{1H} NMR - 101MHz, Chloroform-d

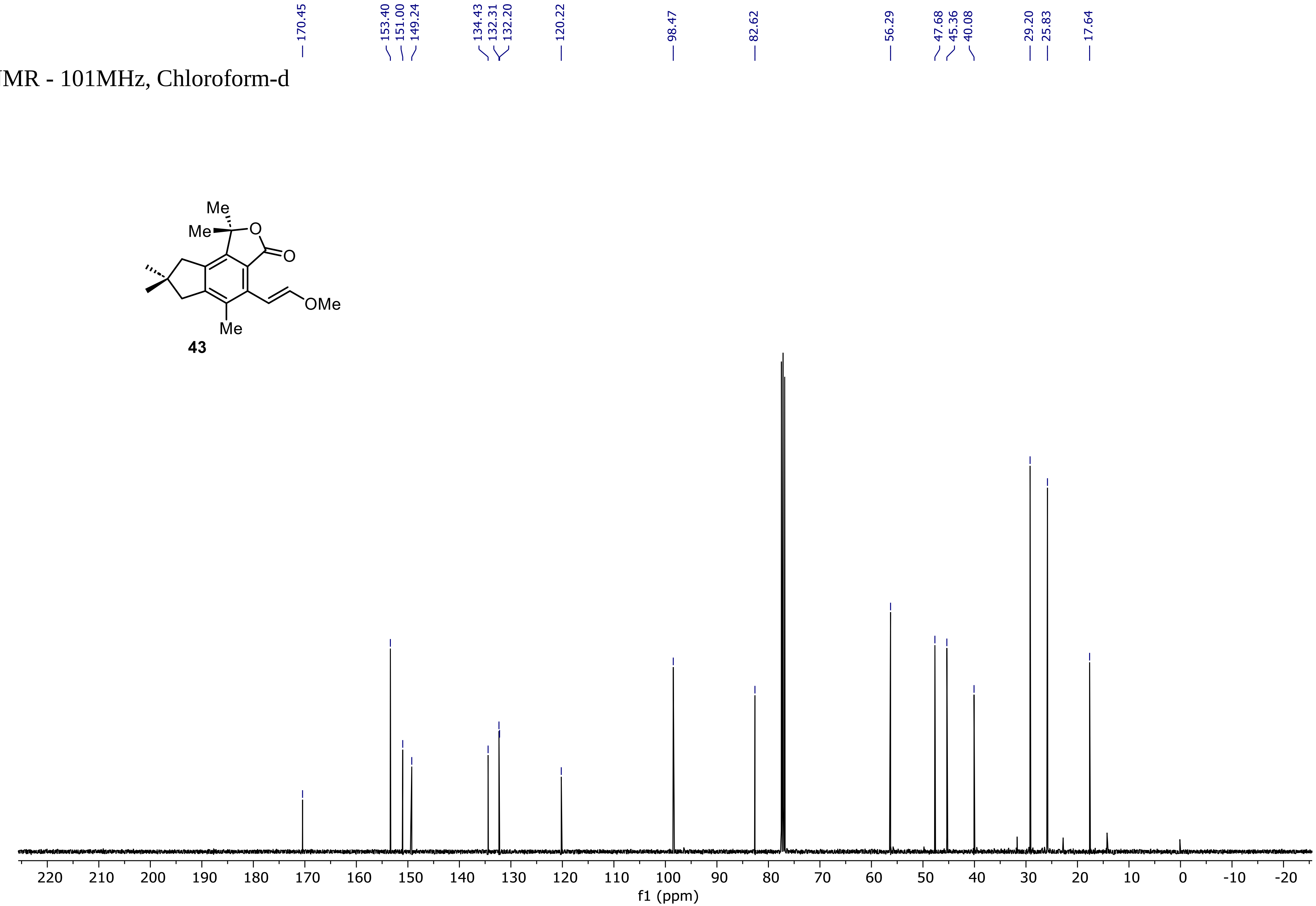

1D NOESY

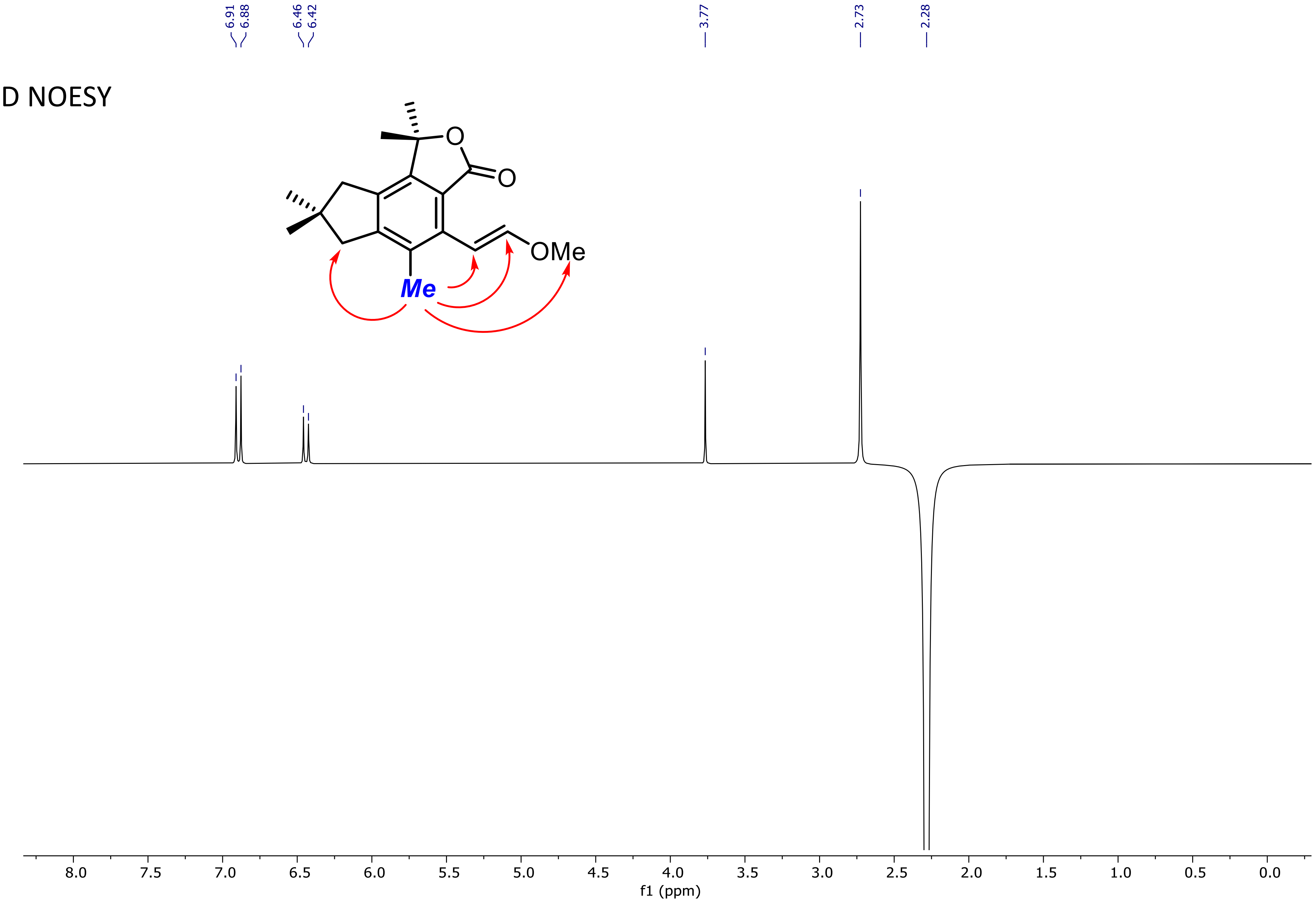
6.91
6.88
6.46
6.42
3.77
2.73
2.28
O
O
OMe
Me
8.0
7.5
7.0
6.5
6.0
5.5
5.0
4.5
4.0
3.5
3.0
2.5
2.0
1.5
1.0
0.5
0.0
f1 (ppm)

1H NMR - 400 MHz, Chloroform-d

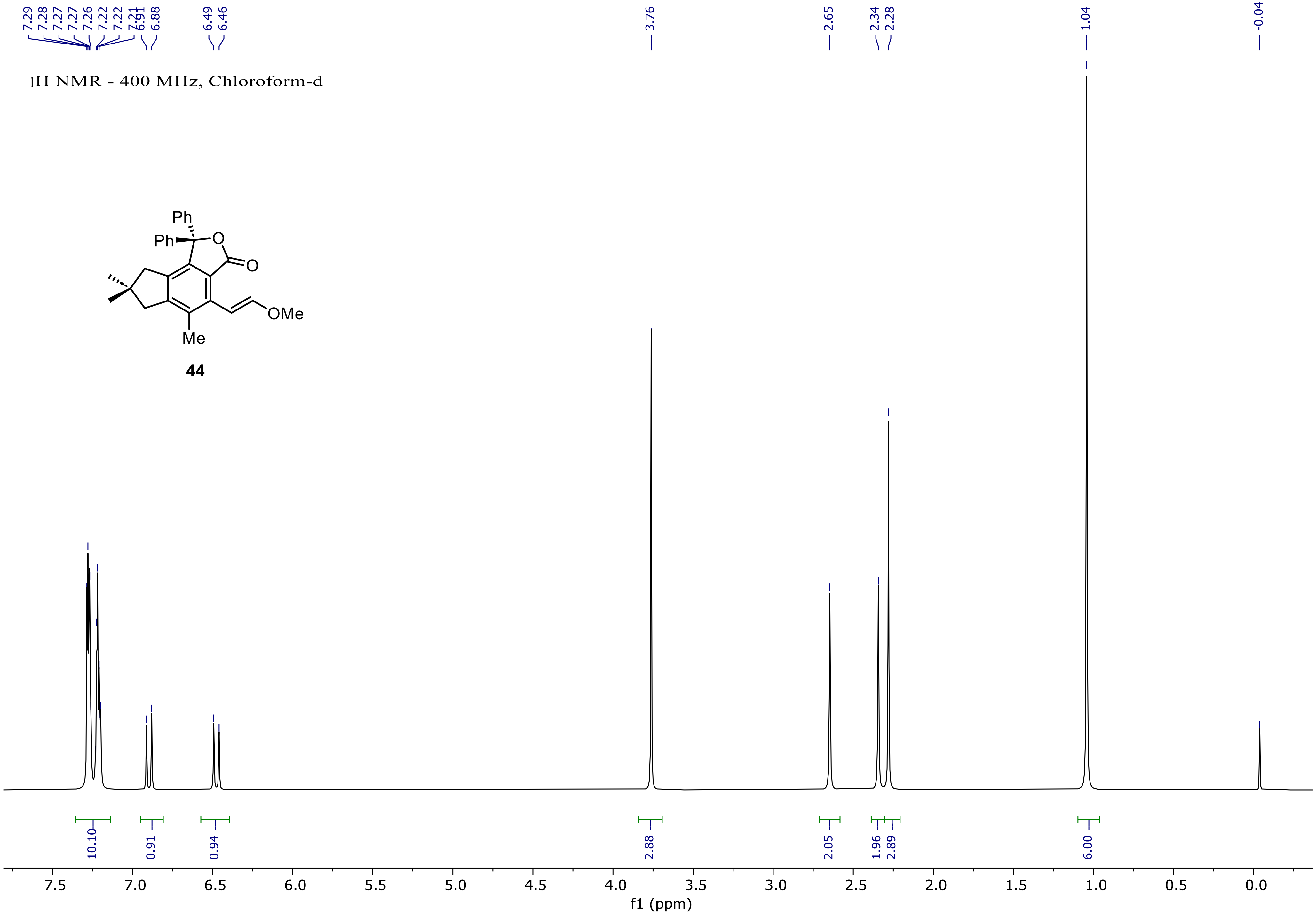

13C{1H} NMR - 101MHz, Chloroform-d

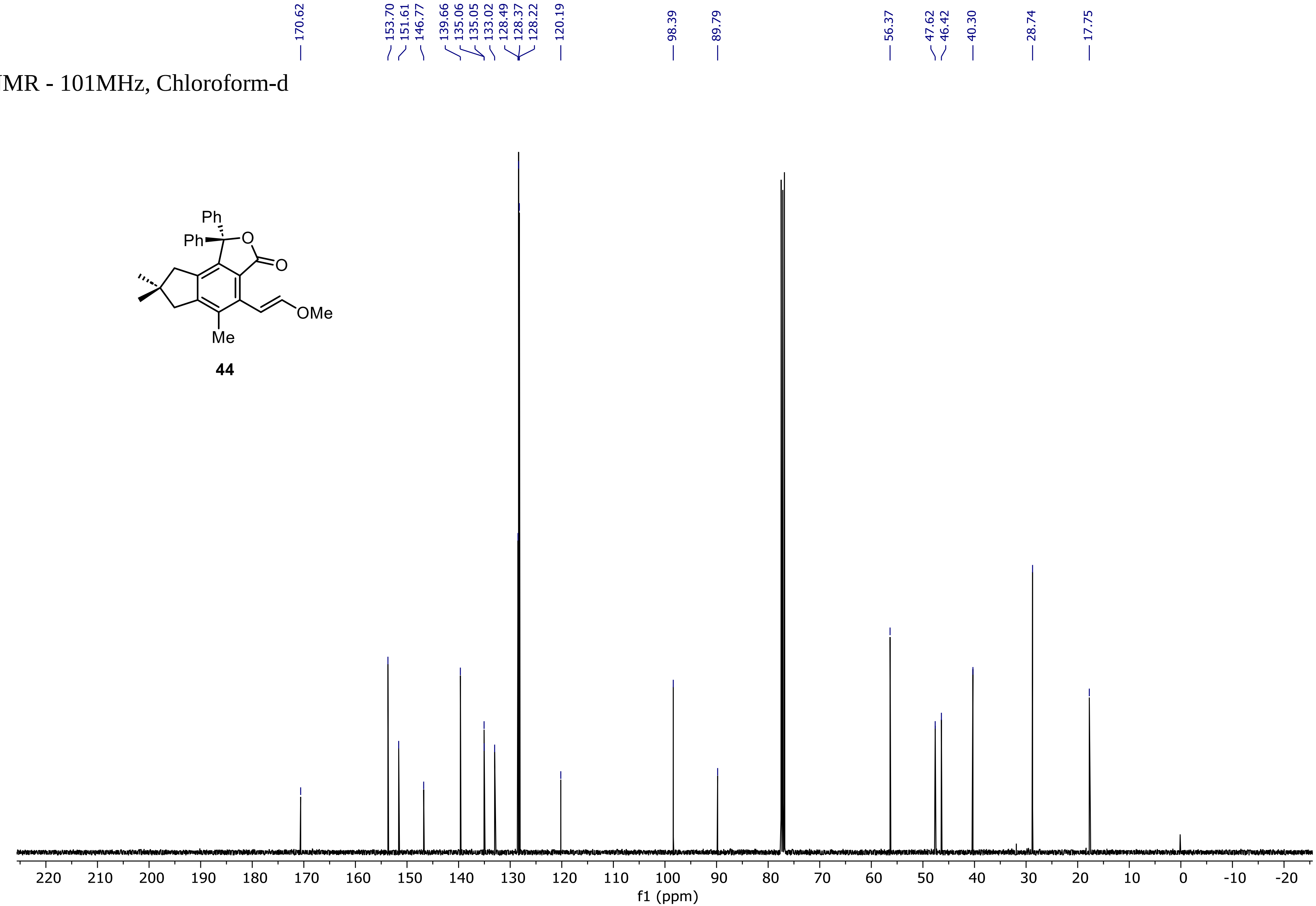

44

1D NOESY

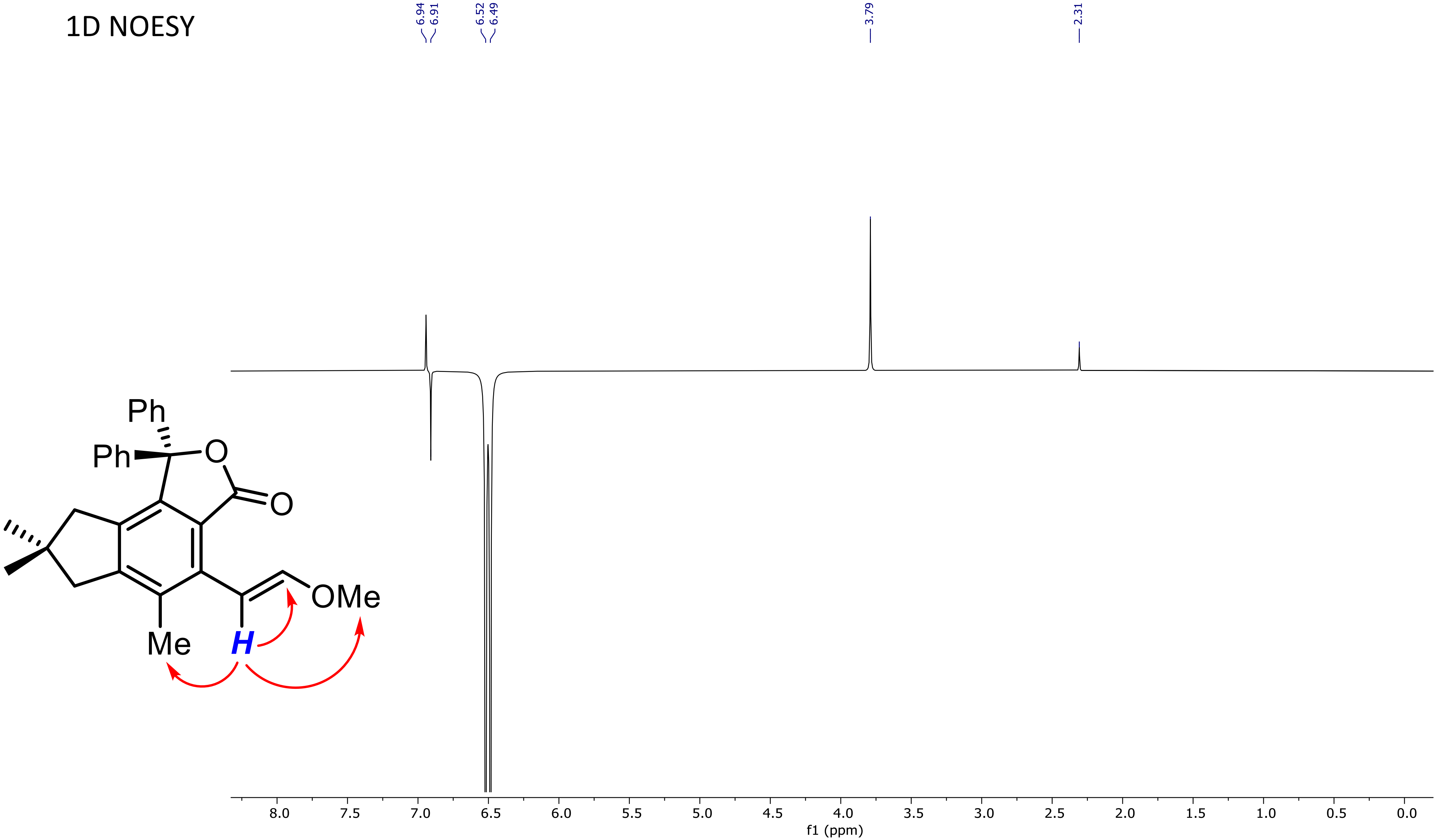
6.94
6.91
6.52
6.49
3.79
2.31
8.0
7.5
7.0
6.5
6.0
5.5
5.0
4.5
4.0
3.5
3.0
2.5
2.0
1.5
1.0
0.5
0.0
f1 (ppm)
Ph
Ph
O
O
OMe
Me
H

1H NMR - 400 MHz, Chloroform-d

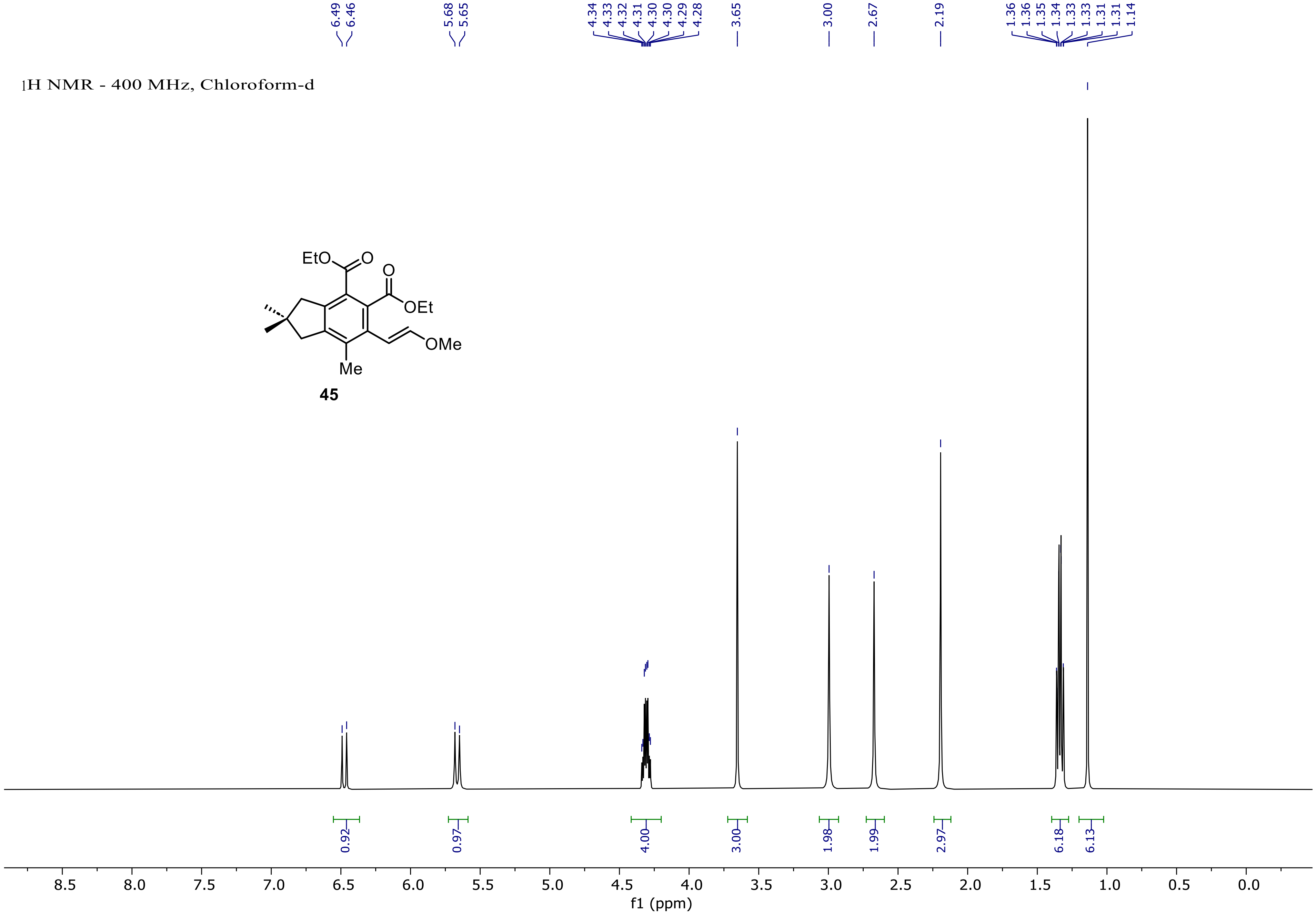

13C{1H} NMR - 101MHz, Chloroform-d

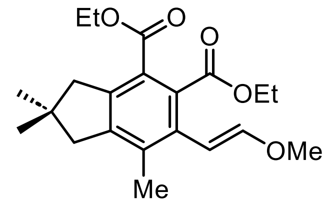

45

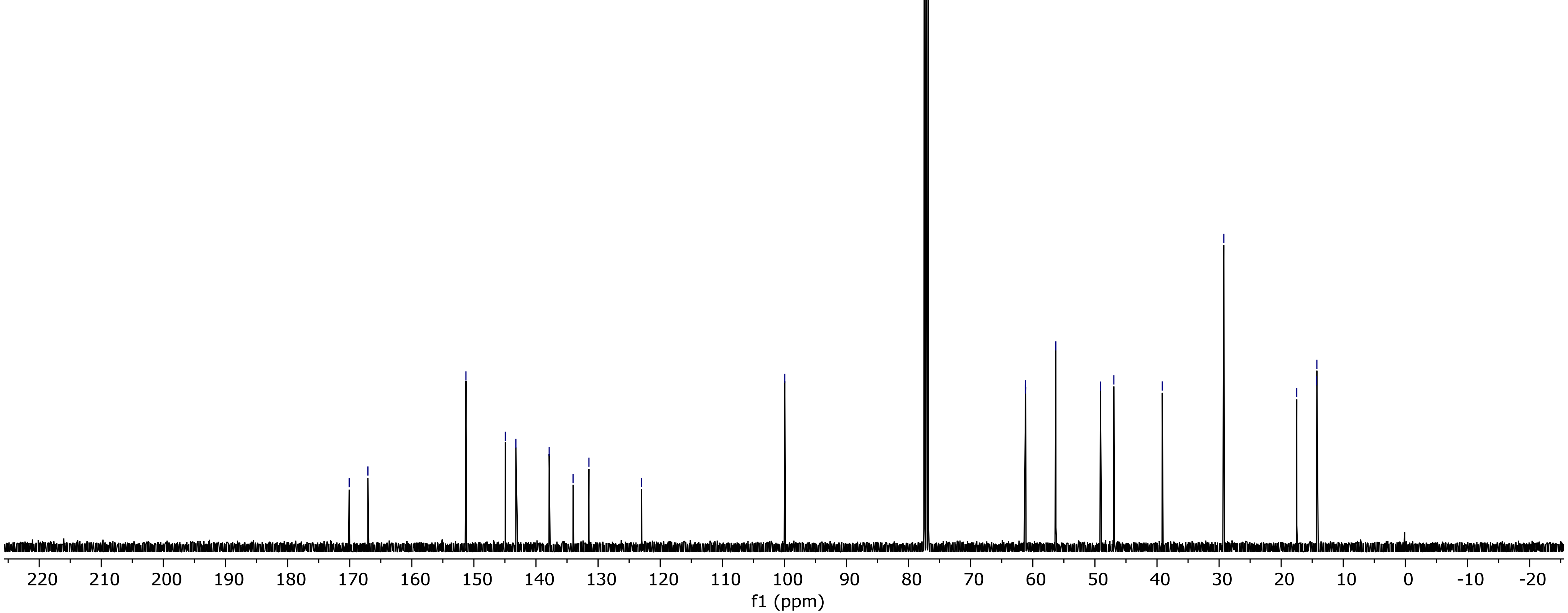

1D NOESY

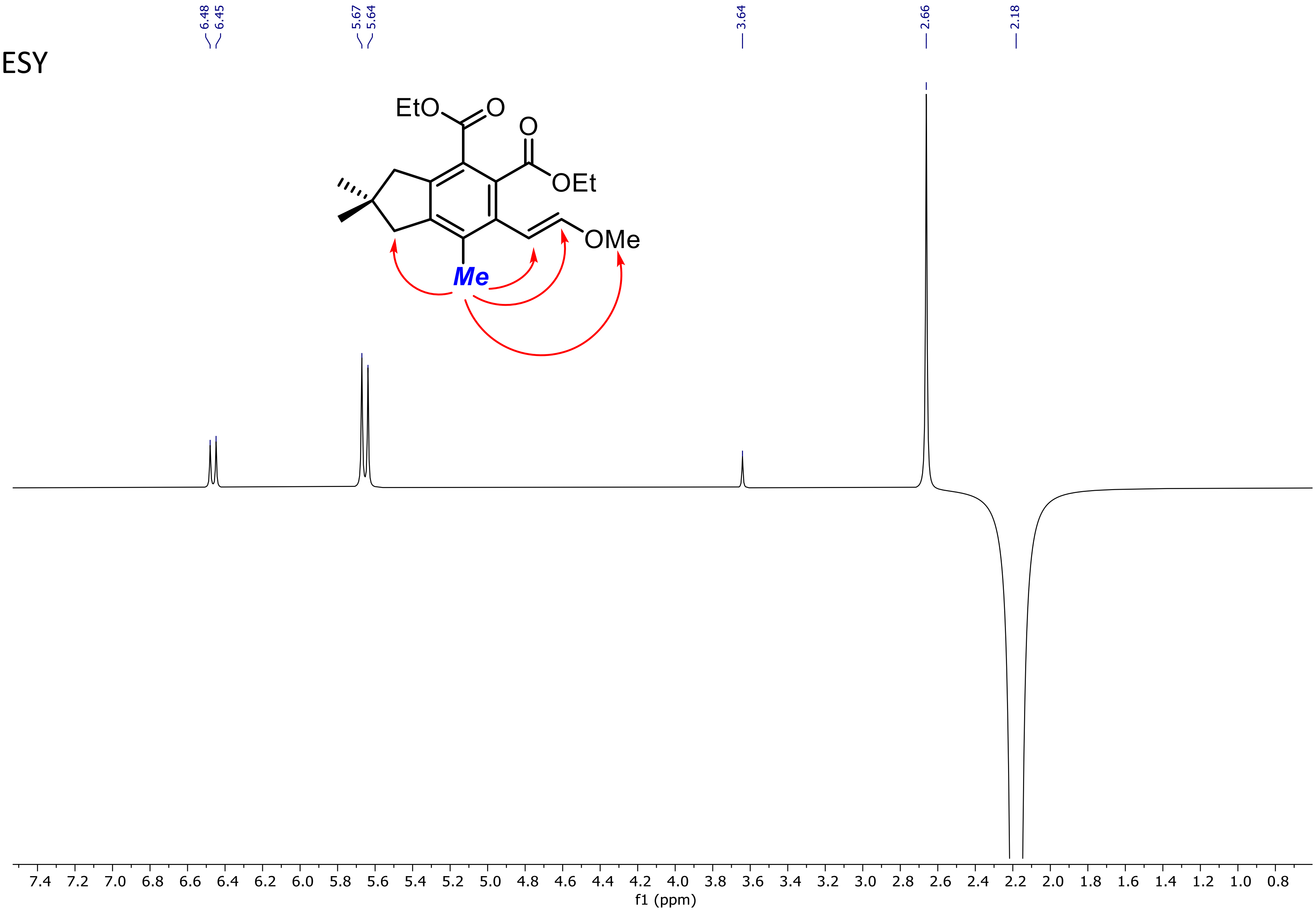
6.48
6.45
5.67
5.64
3.64
2.66
2.18
EtO
O
O
OEt
OMe
Me
7.4 7.2 7.0 6.8 6.6 6.4 6.2 6.0 5.8 5.6 5.4 5.2 5.0 4.8 4.6 4.4 4.2 4.0 3.8 3.6 3.4 3.2 3.0 2.8 2.6 2.4 2.2 2.0 1.8 1.6 1.4 1.2 1.0 0.8
f1 (ppm)

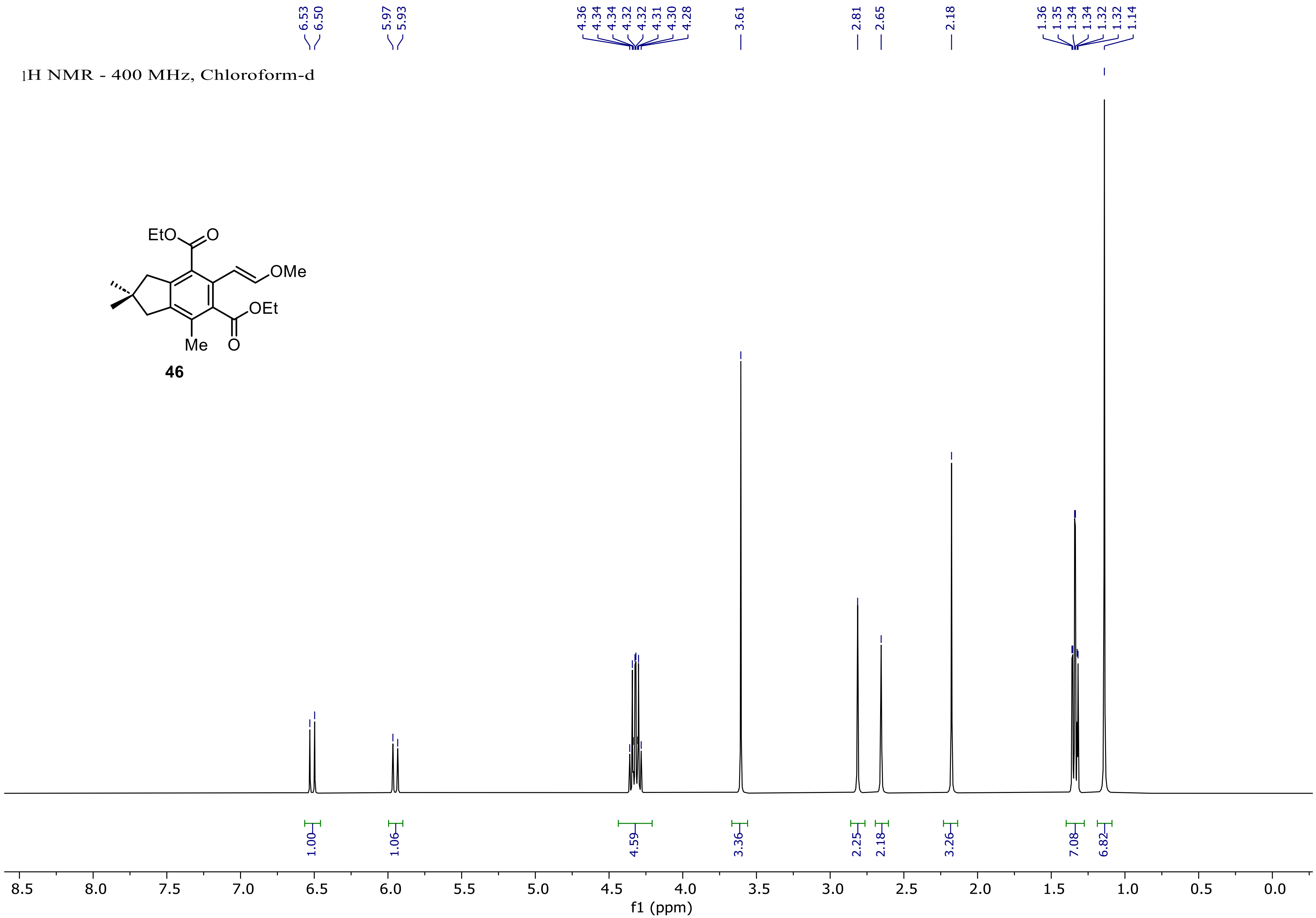
1H NMR - 400 MHz, Chloroform-d
6.53
6.50
5.97
5.93
4.36
4.34
4.34
4.32
4.32
4.31
4.30
4.28
3.61
2.81
2.65
2.18
1.36
1.35
1.34
1.34
1.32
1.32
1.14
EtO
O
OMe
OEt
Me
O
46
1.00
1.06
4.59
3.36
2.25
2.18
3.26
7.08
6.82
8.5 8.0 7.5 7.0 6.5 6.0 5.5 5.0 4.5 4.0 3.5 3.0 2.5 2.0 1.5 1.0 0.5 0.0
f1 (ppm)

13C{1H} NMR - 101MHz, Chloroform-d

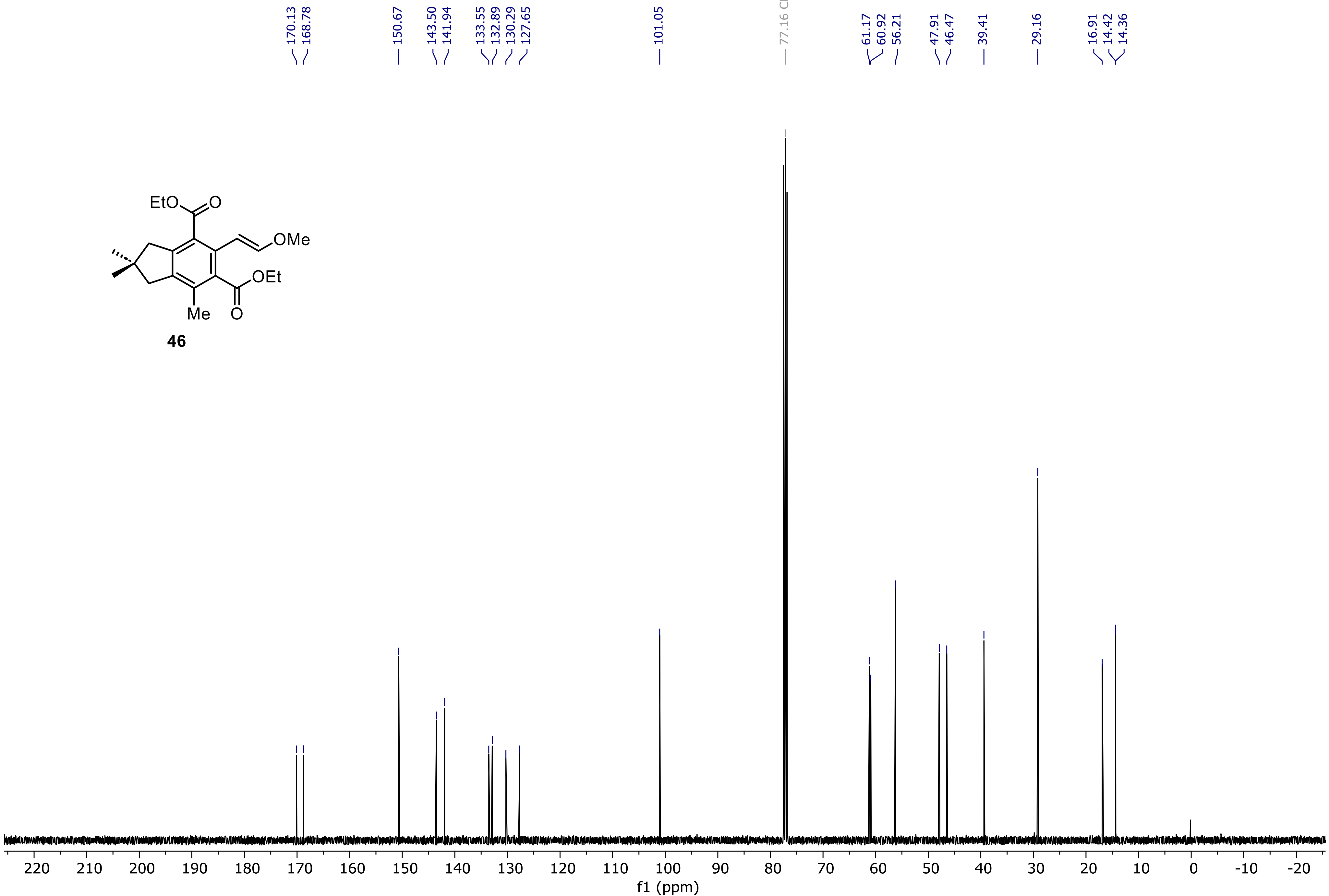

1D NOESY

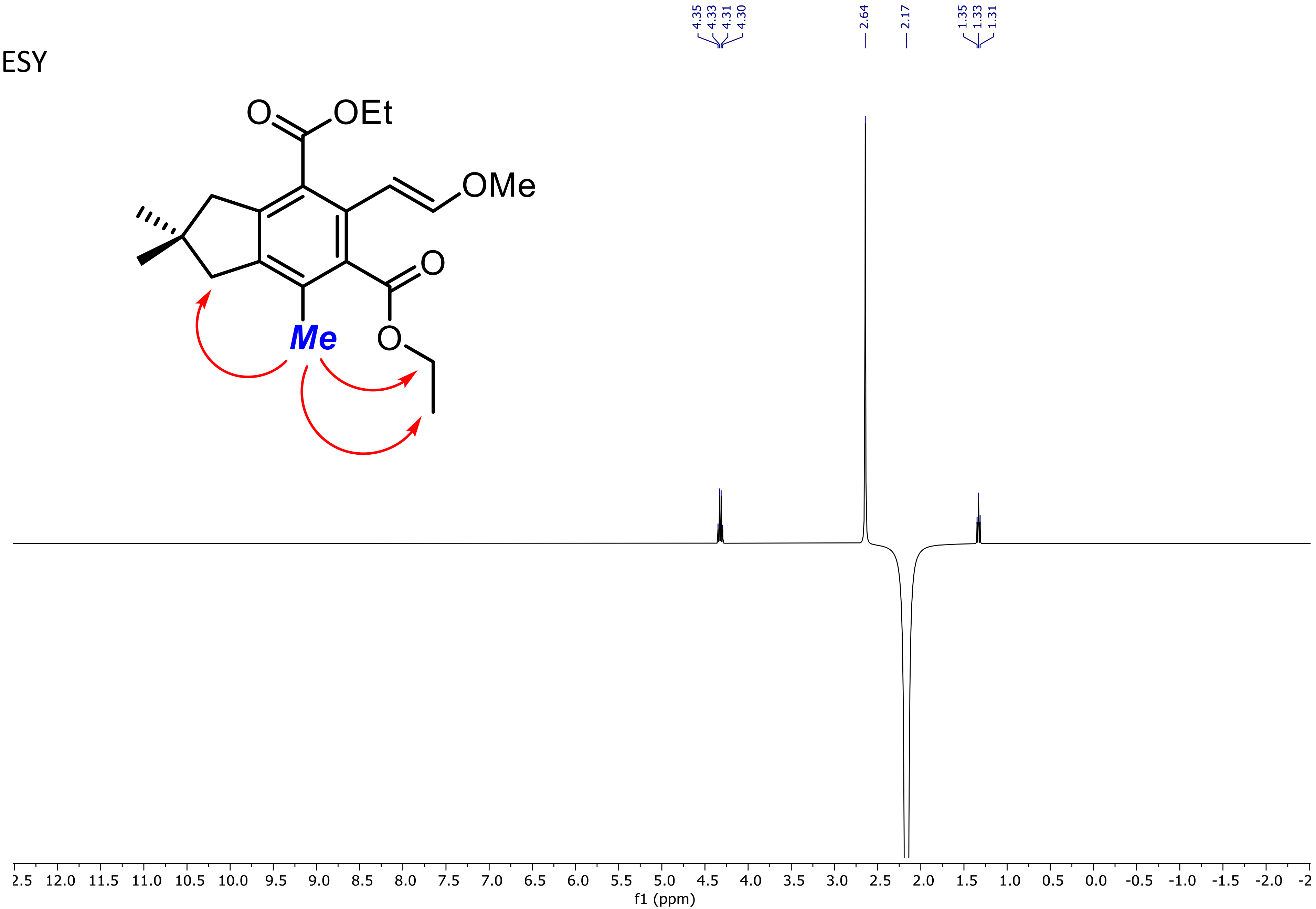

OEt
O
OMe
O
O
Me
4.35
4.33
4.31
4.30
2.64
2.17
1.35
1.33
1.31
2.5 12.0 11.5 11.0 10.5 10.0 9.5 9.0 8.5 8.0 7.5 7.0 6.5 6.0 5.5 5.0 4.5 4.0 3.5 3.0 2.5 2.0 1.5 1.0 0.5 0.0 -0.5 -1.0 -1.5 -2.0 -2
f1 (ppm)

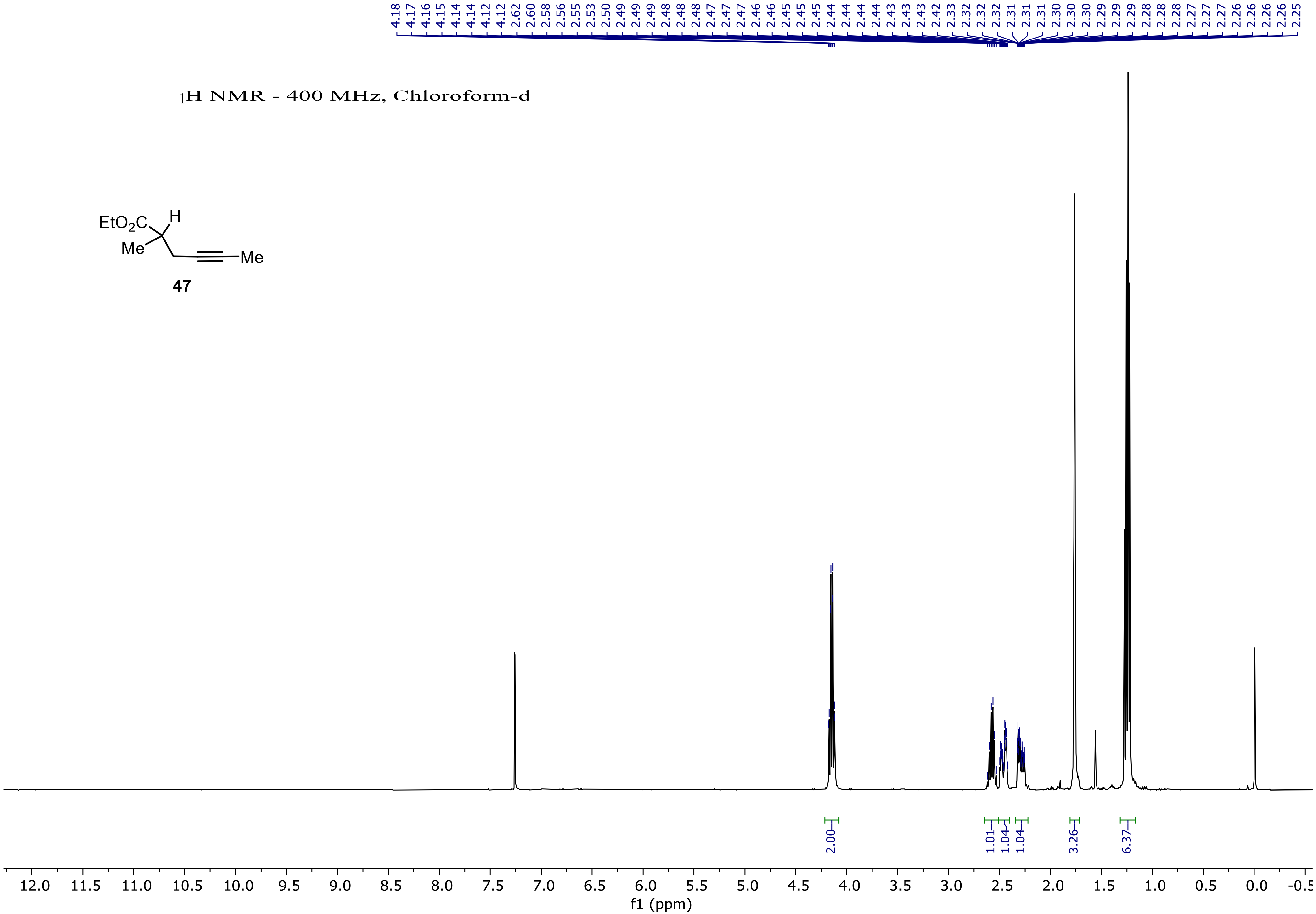
4.18 4.17 4.16 4.15 4.14 4.14 4.12 4.12 2.62 2.60 2.58 2.56 2.55 2.53 2.50 2.49 2.49 2.49 2.48 2.48 2.48 2.47 2.47 2.47 2.46 2.46 2.45 2.45 2.45 2.44 2.44 2.44 2.44 2.43 2.43 2.43 2.42 2.42 2.33 2.32 2.32 2.32 2.31 2.31 2.31 2.30 2.30 2.30 2.29 2.29 2.29 2.28 2.28 2.28 2.27 2.27 2.27 2.26 2.26 2.26 2.26 2.25
1H NMR - 400 MHz, Chloroform-d
EtO2C
H
Me
Me
47
2.00
1.01
1.04
1.04
3.26
6.37
12.0 11.5 11.0 10.5 10.0 9.5 9.0 8.5 8.0 7.5 7.0 6.5 6.0 5.5 5.0 4.5 4.0 3.5 3.0 2.5 2.0 1.5 1.0 0.5 0.0 -0.5
f1 (ppm)

1H NMR - 400 MHz, Chloroform-d

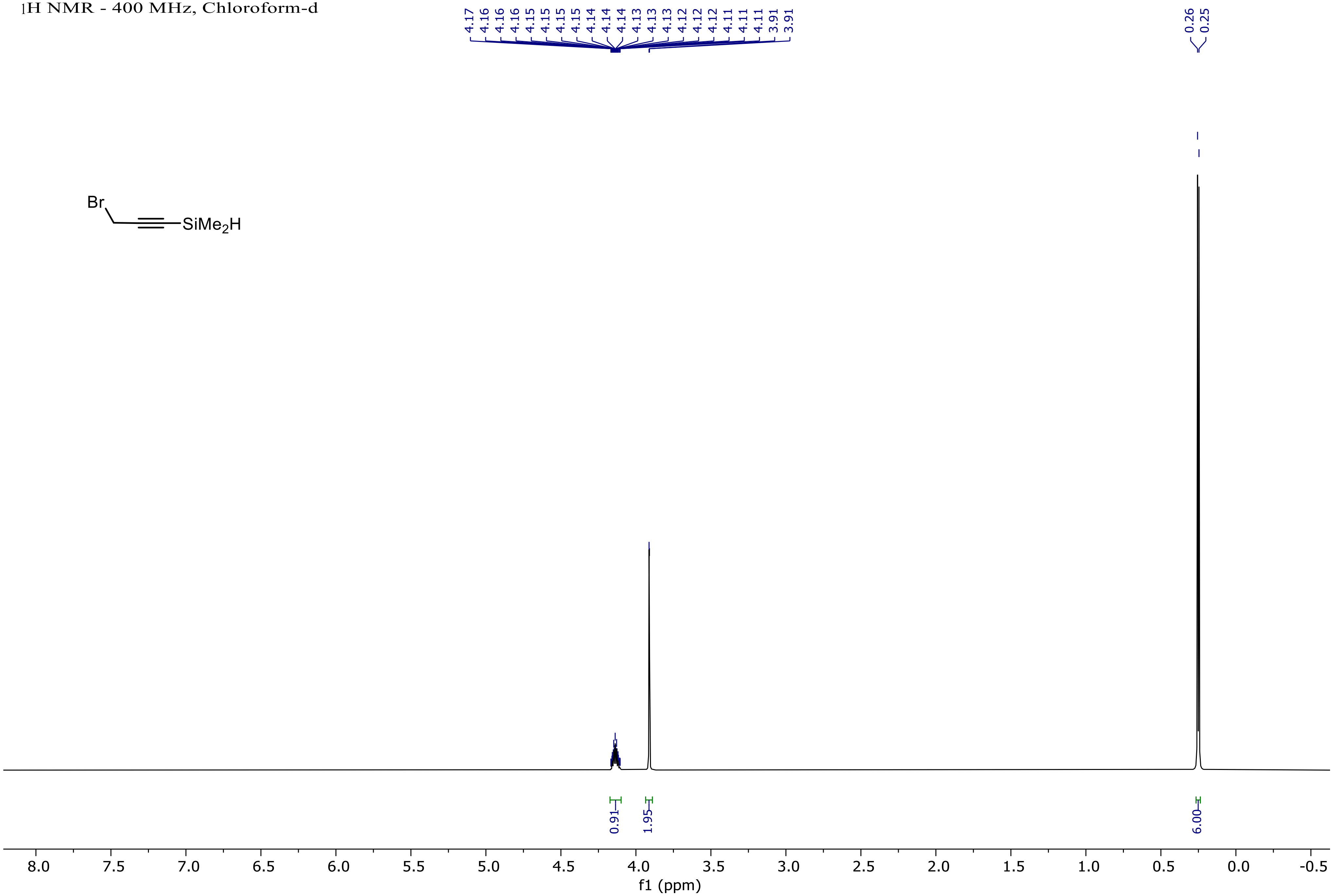

13C{1H} NMR - 101MHz, Chloroform-d

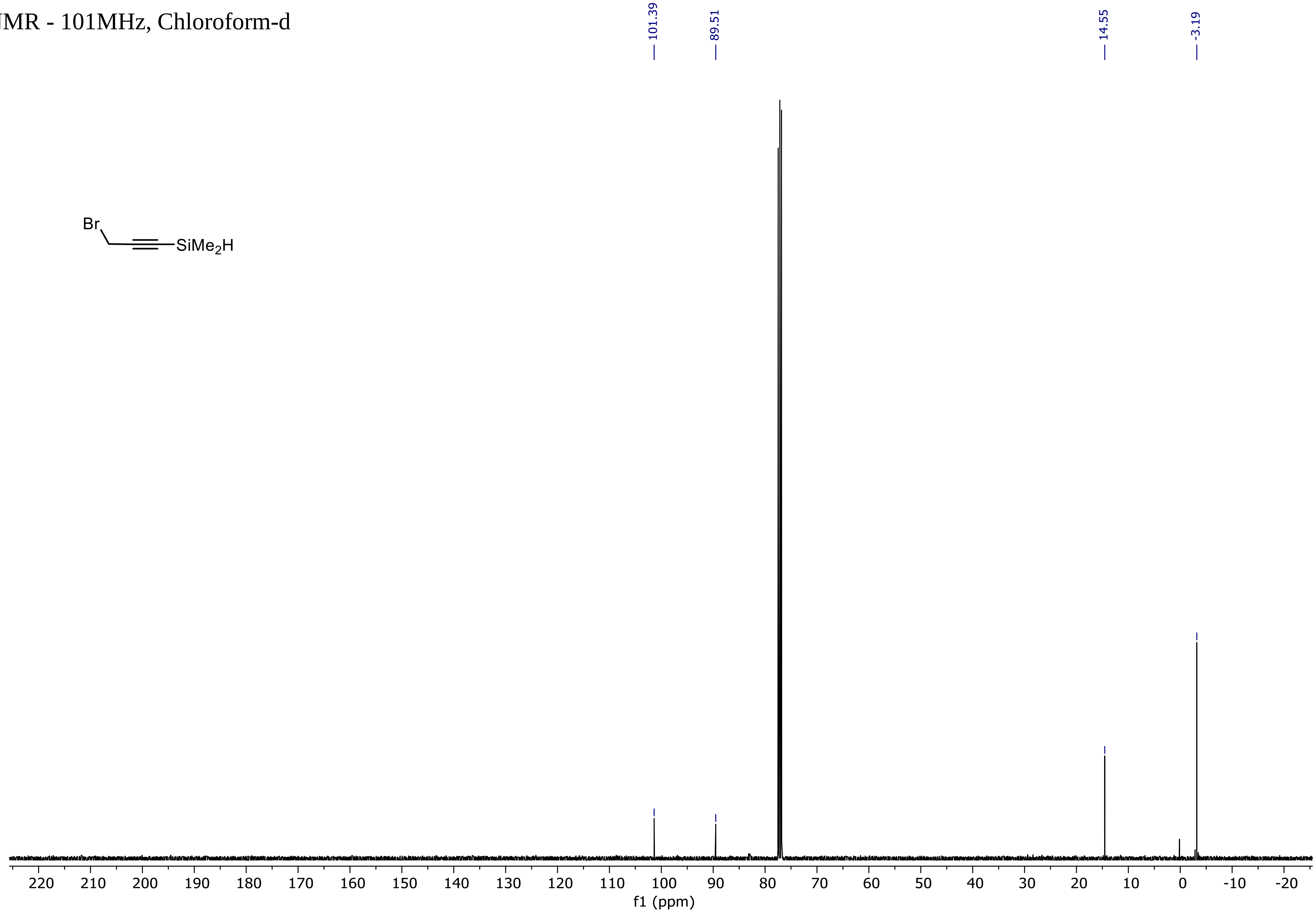

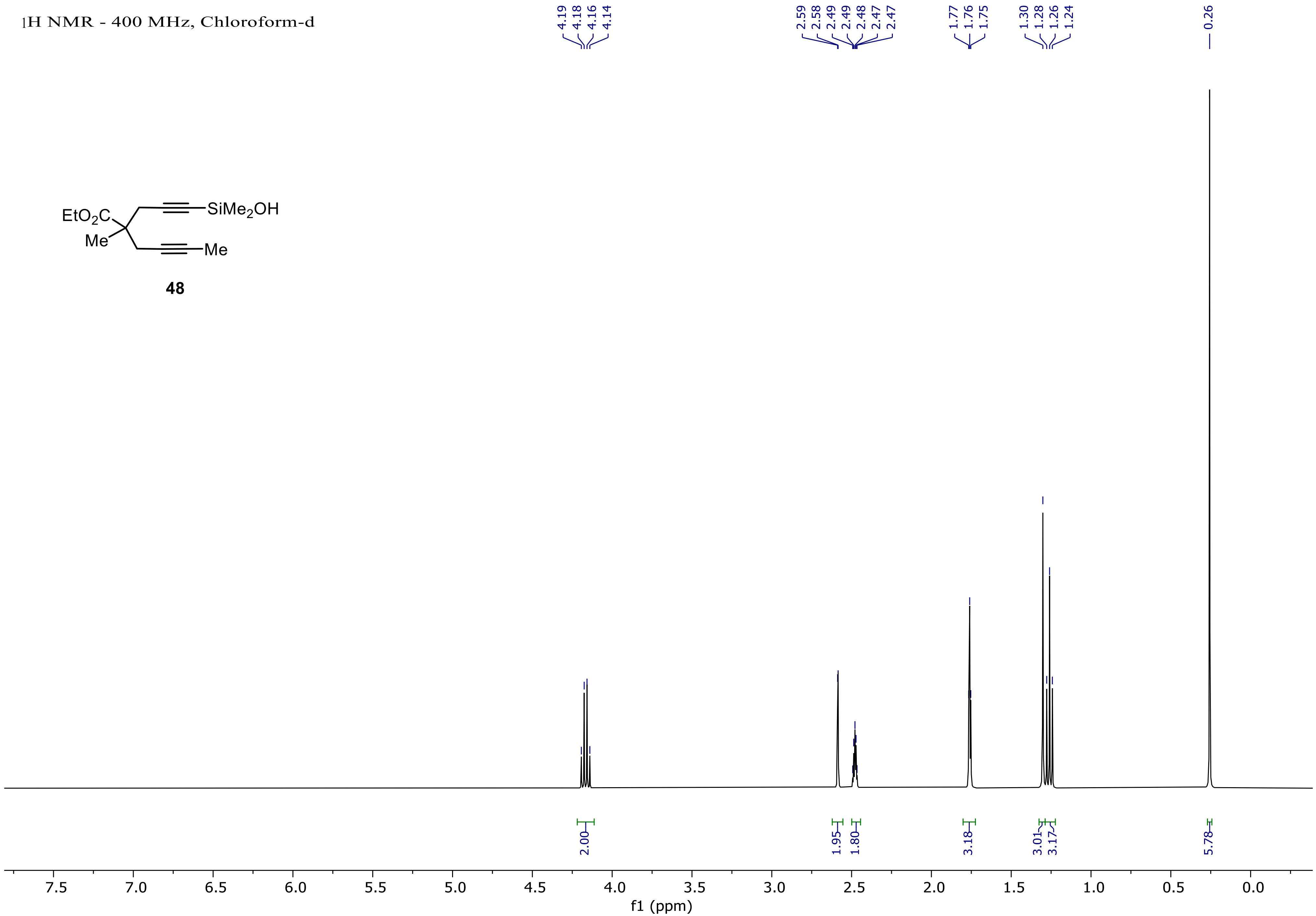
1H NMR - 400 MHz, Chloroform-d
4.19 4.18 4.16 4.14
2.59 2.58 2.49 2.49 2.48 2.47 2.47
1.77 1.76 1.75
1.30 1.28 1.26 1.24
0.26
EtO2C
SiMe2OH
Me
Me
48
2.00
1.95
1.80
3.18
3.01
3.17
5.78
7.5 7.0 6.5 6.0 5.5 5.0 4.5 4.0 3.5 3.0 2.5 2.0 1.5 1.0 0.5 0.0
f1 (ppm)

13C{1H} NMR - 101MHz, Chloroform-d

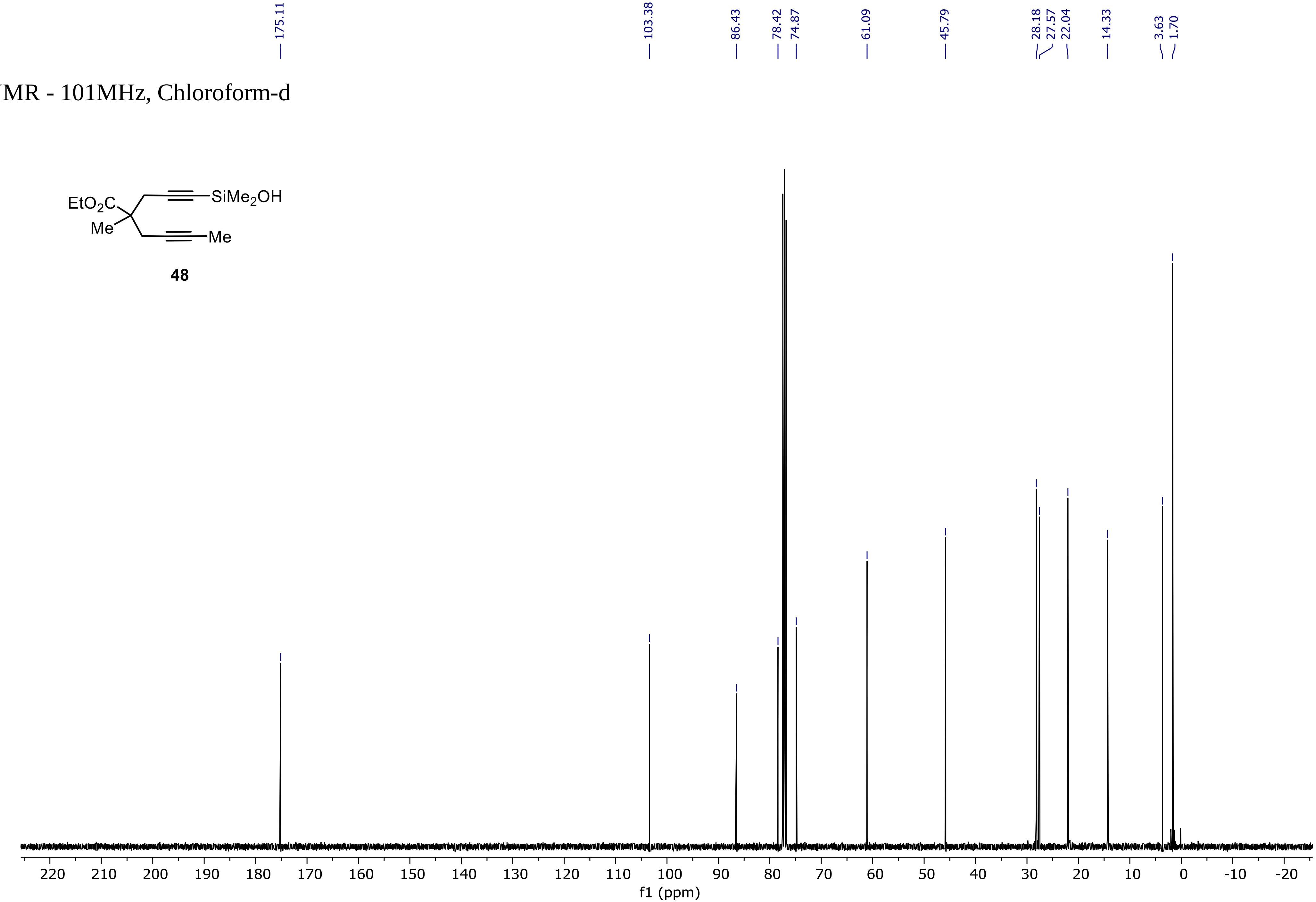

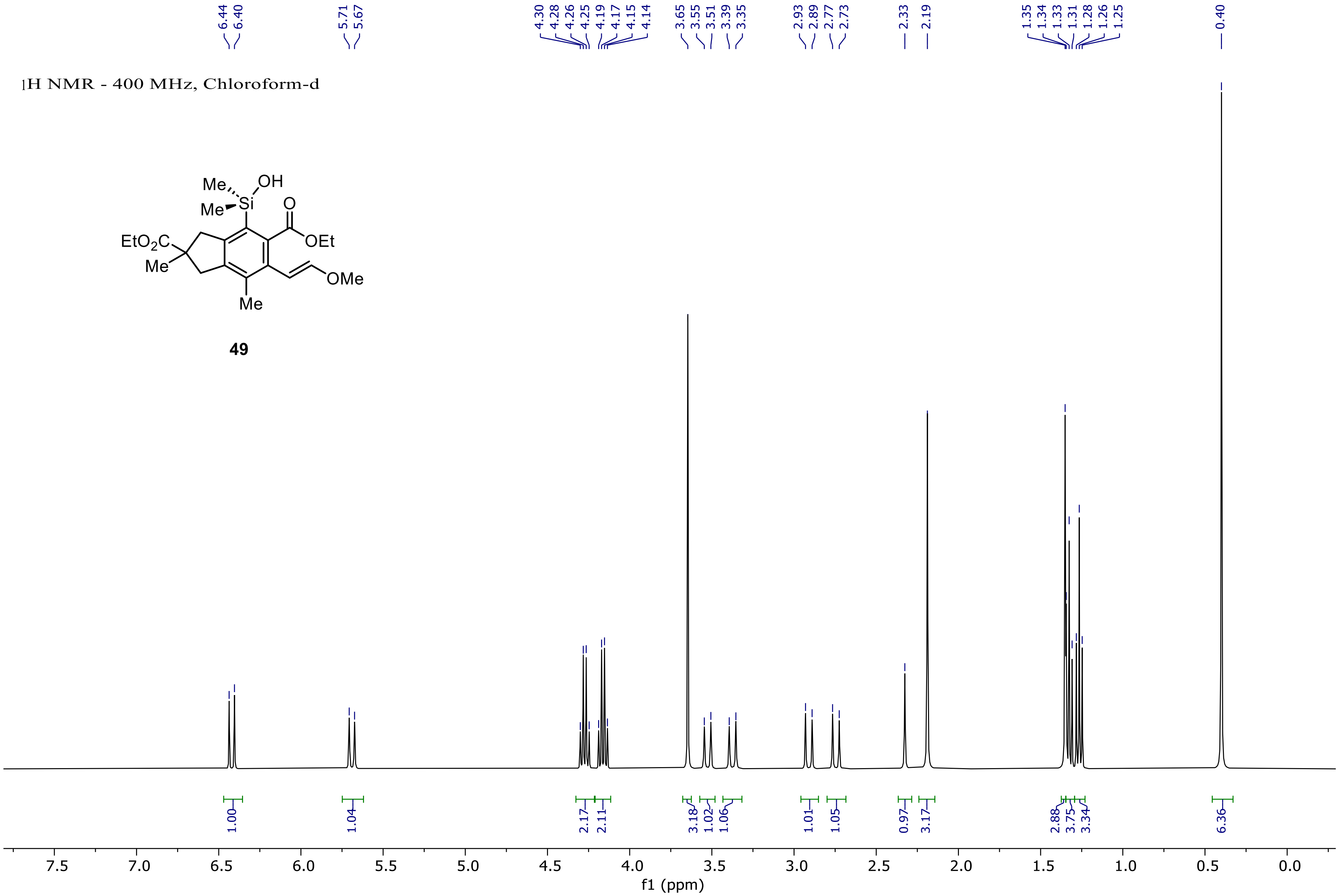
1H NMR - 400 MHz, Chloroform-d
6.44
6.40
5.71
5.67
4.30
4.28
4.26
4.25
4.19
4.17
4.15
4.14
3.65
3.55
3.51
3.39
3.35
2.93
2.89
2.77
2.73
2.33
2.19
1.35
1.34
1.33
1.31
1.28
1.26
1.25
0.40
Me
OH
Si
Me
O
OEt
EtO_2C
Me
OMe
Me
49
1.00
1.04
2.17
2.11
3.18
1.02
1.06
1.01
1.05
0.97
3.17
2.88
3.75
3.34
6.36
7.5
7.0
6.5
6.0
5.5
5.0
4.5
4.0
3.5
3.0
2.5
2.0
1.5
1.0
0.5
0.0
f1 (ppm)

13C{1H} NMR - 101MHz, Chloroform-d

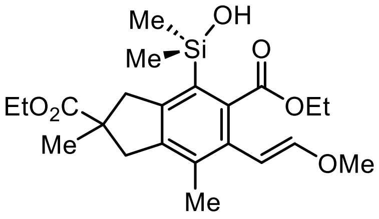

49

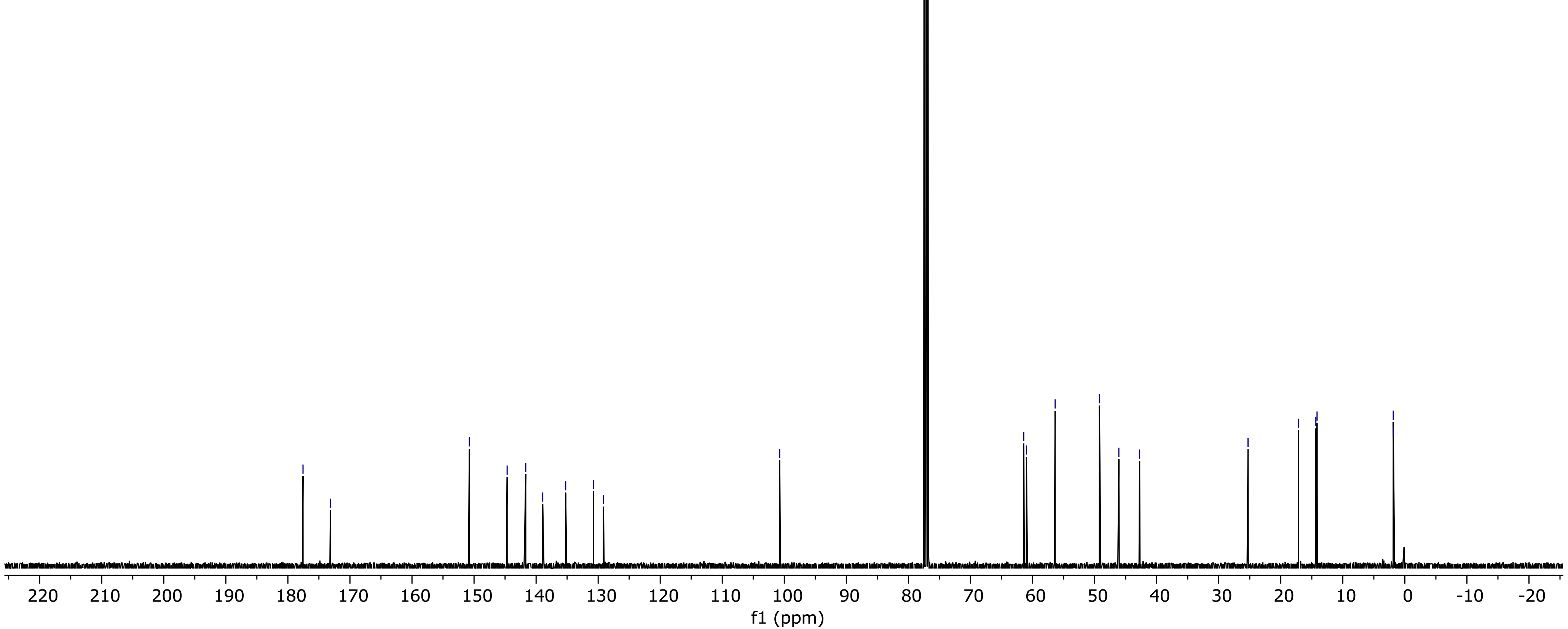

1D NOESY

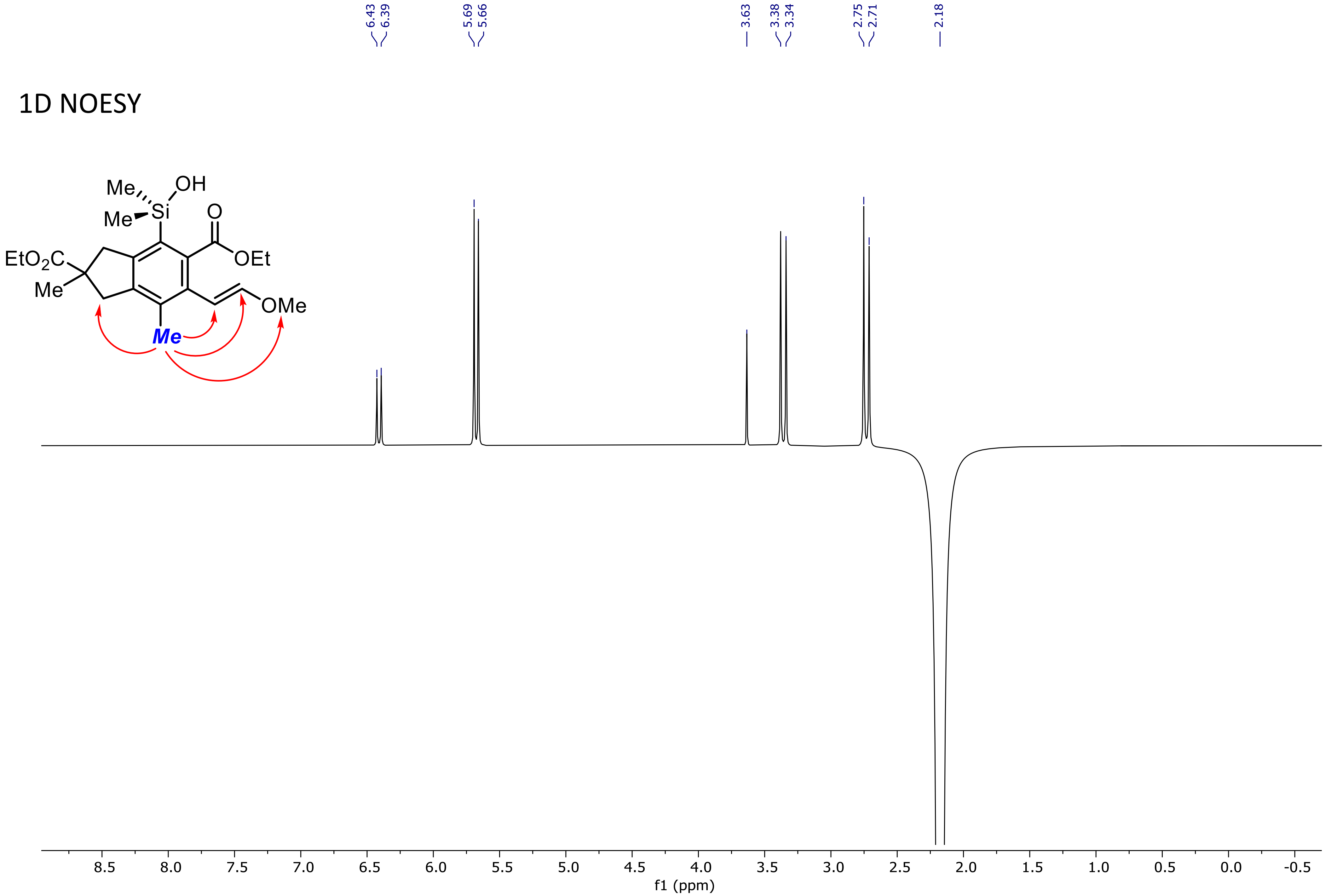

Me
OH
Me
Si
O
EtO2C
OEt
Me
OMe
Me
6.43
6.39
5.69
5.66
3.63
3.38
3.34
2.75
2.71
2.18
8.5
8.0
7.5
7.0
6.5
6.0
5.5
5.0
4.5
4.0
3.5
3.0
2.5
2.0
1.5
1.0
0.5
0.0
-0.5
f1 (ppm)

1H NMR - 400 MHz, Chloroform-d

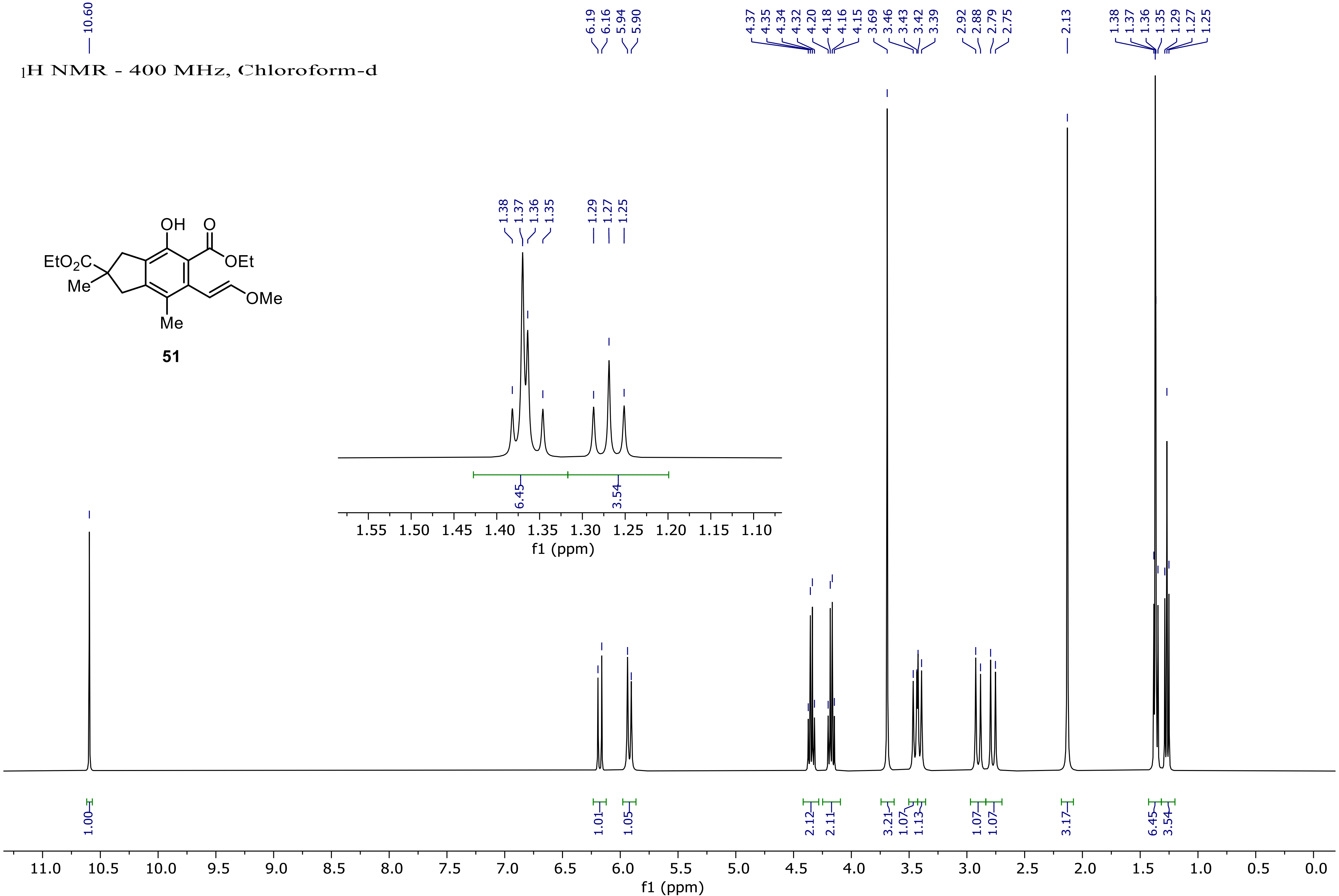

13C{1H} NMR - 101MHz, Chloroform-d

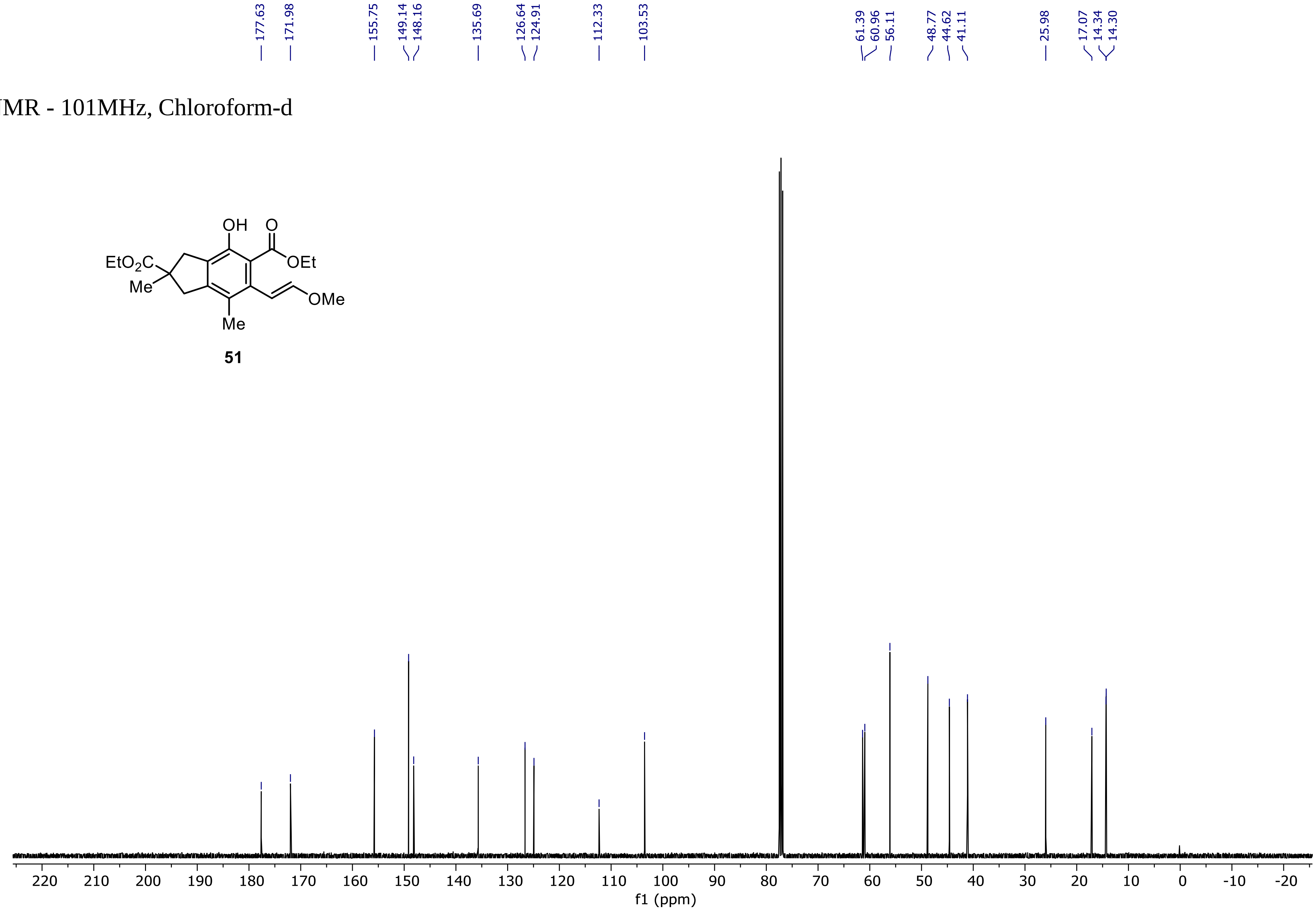

1D NOESY

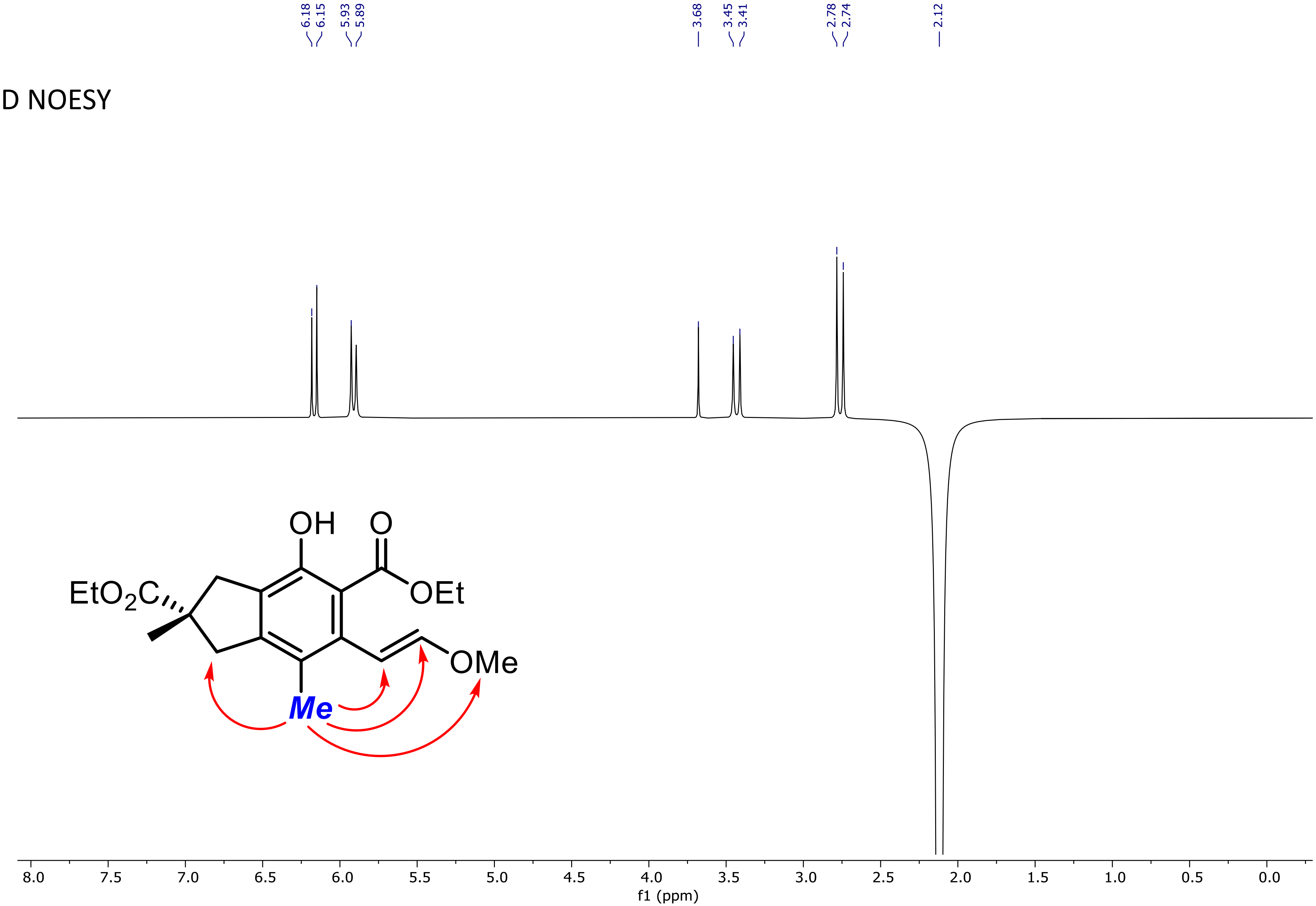

6.18
6.15
5.93
5.89
3.68
3.45
3.41
2.78
2.74
2.12
8.0 7.5 7.0 6.5 6.0 5.5 5.0 4.5 4.0 3.5 3.0 2.5 2.0 1.5 1.0 0.5 0.0
f1 (ppm)
OH
O
OEt
EtO_2C
OMe
Me

1H NMR - 400 MHz, Chloroform-d

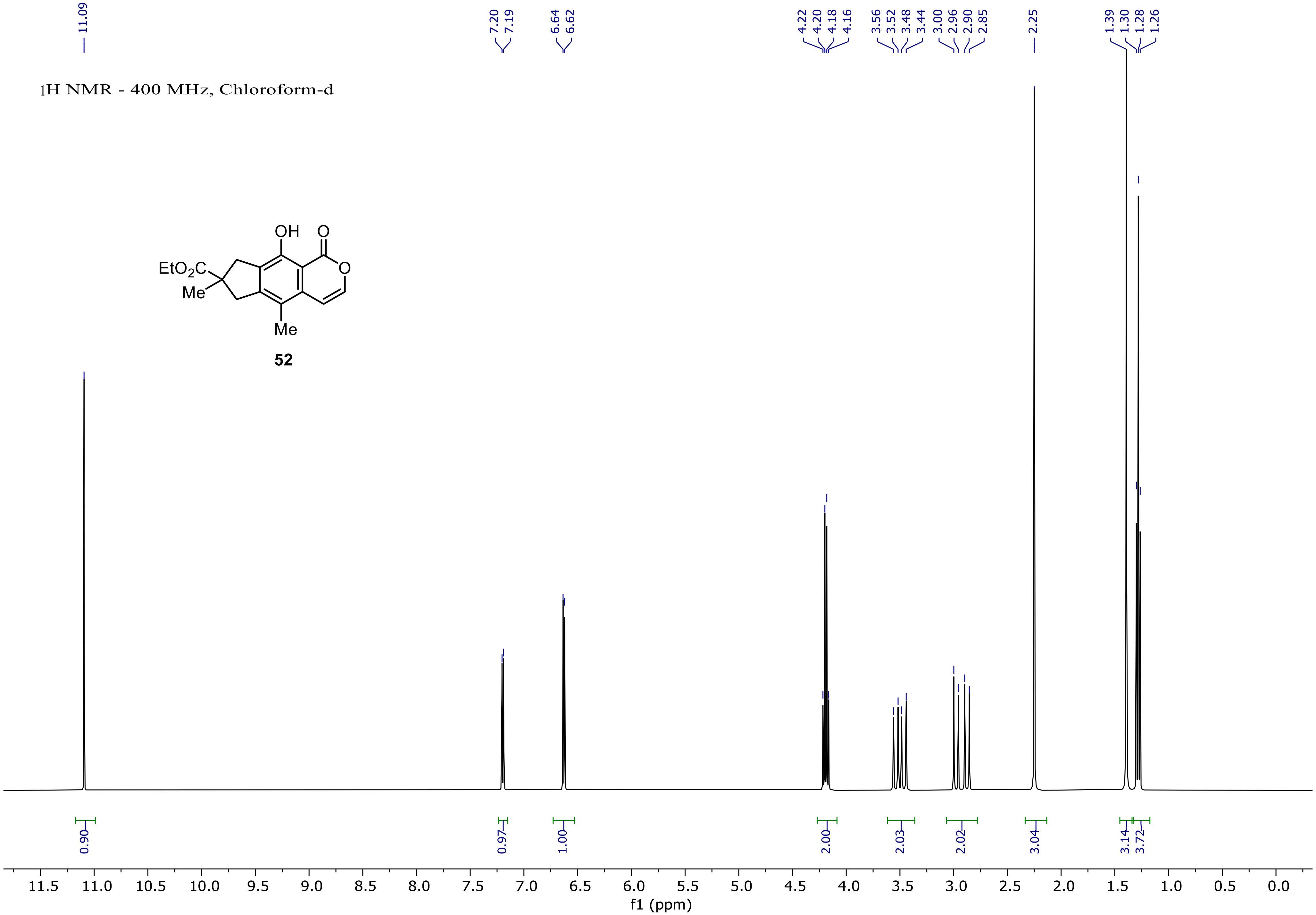

13C{1H} NMR - 101MHz, Chloroform-d

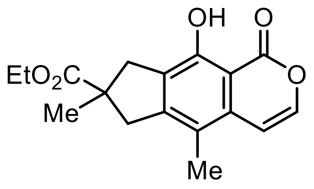
52

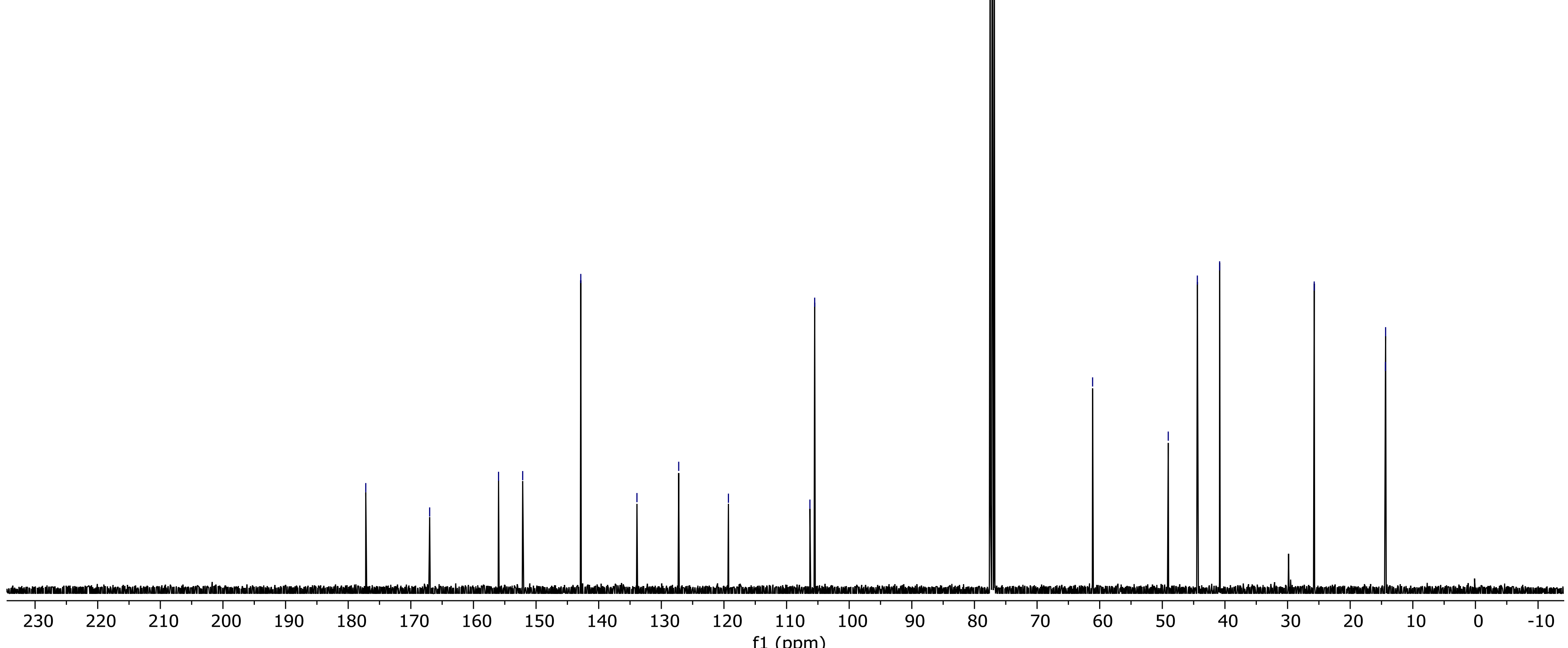

1D NOESY

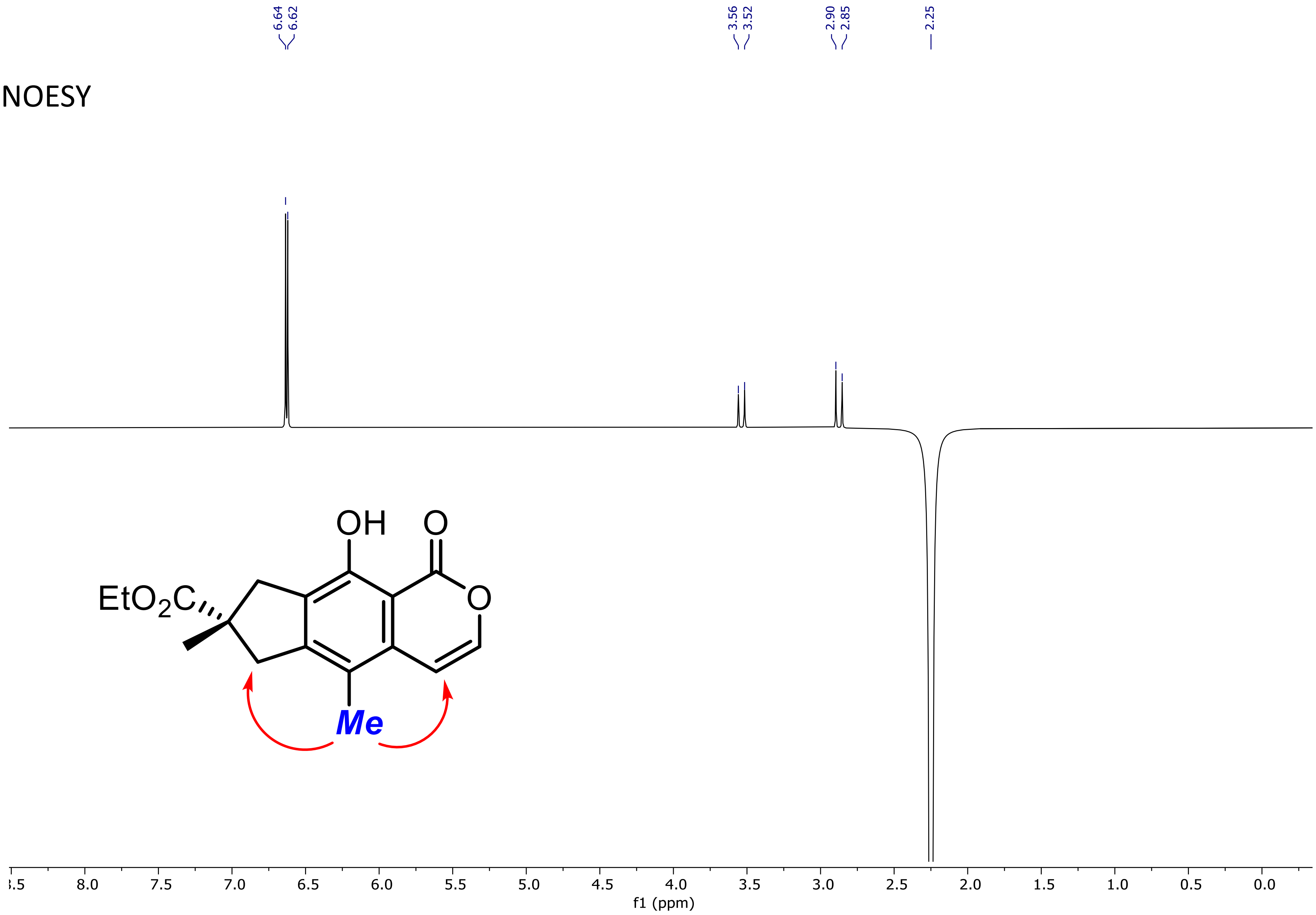

6.64
6.62
3.56
3.52
2.90
2.85
2.25
OH
O
EtO2C
O
Me
8.0
7.5
7.0
6.5
6.0
5.5
5.0
4.5
4.0
3.5
3.0
2.5
2.0
1.5
1.0
0.5
0.0
f1 (ppm)

^{1}H NMR, 400 MHz, DMSO-d_6

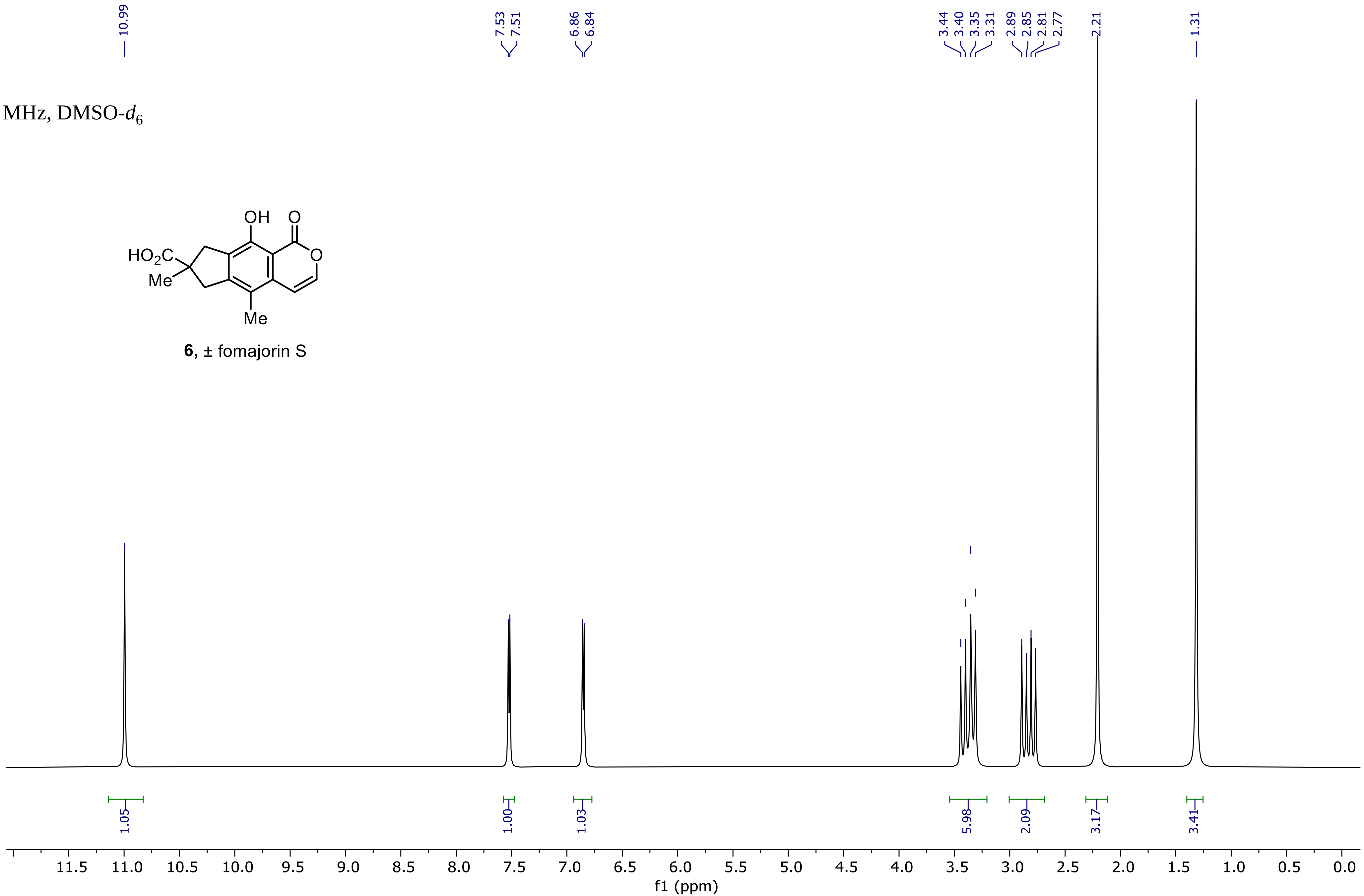

6, ± fomajorin S

13C{1H} NMR - 101MHz, DMSO-d6

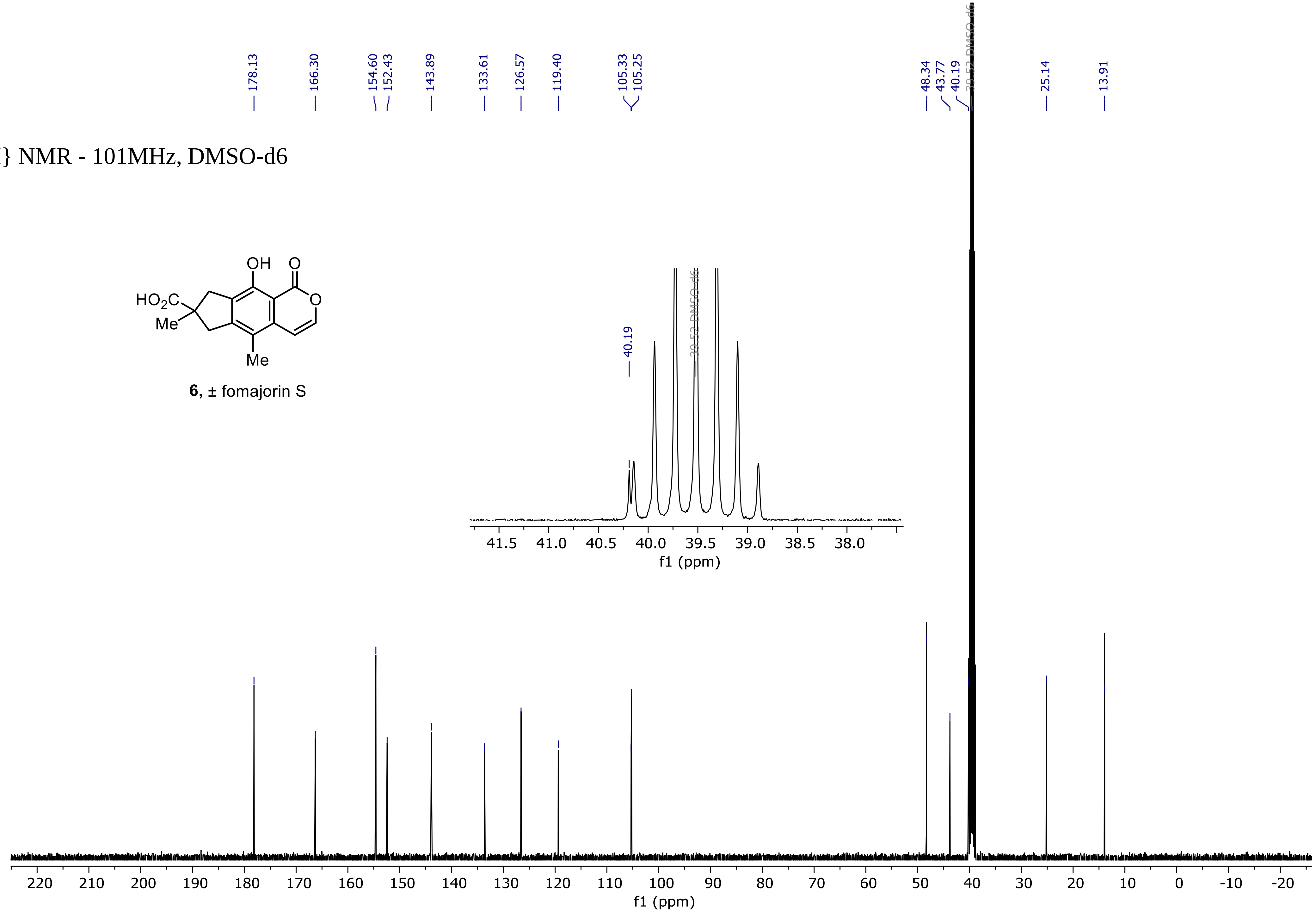

6, ± fomajorin S

1D NOESY

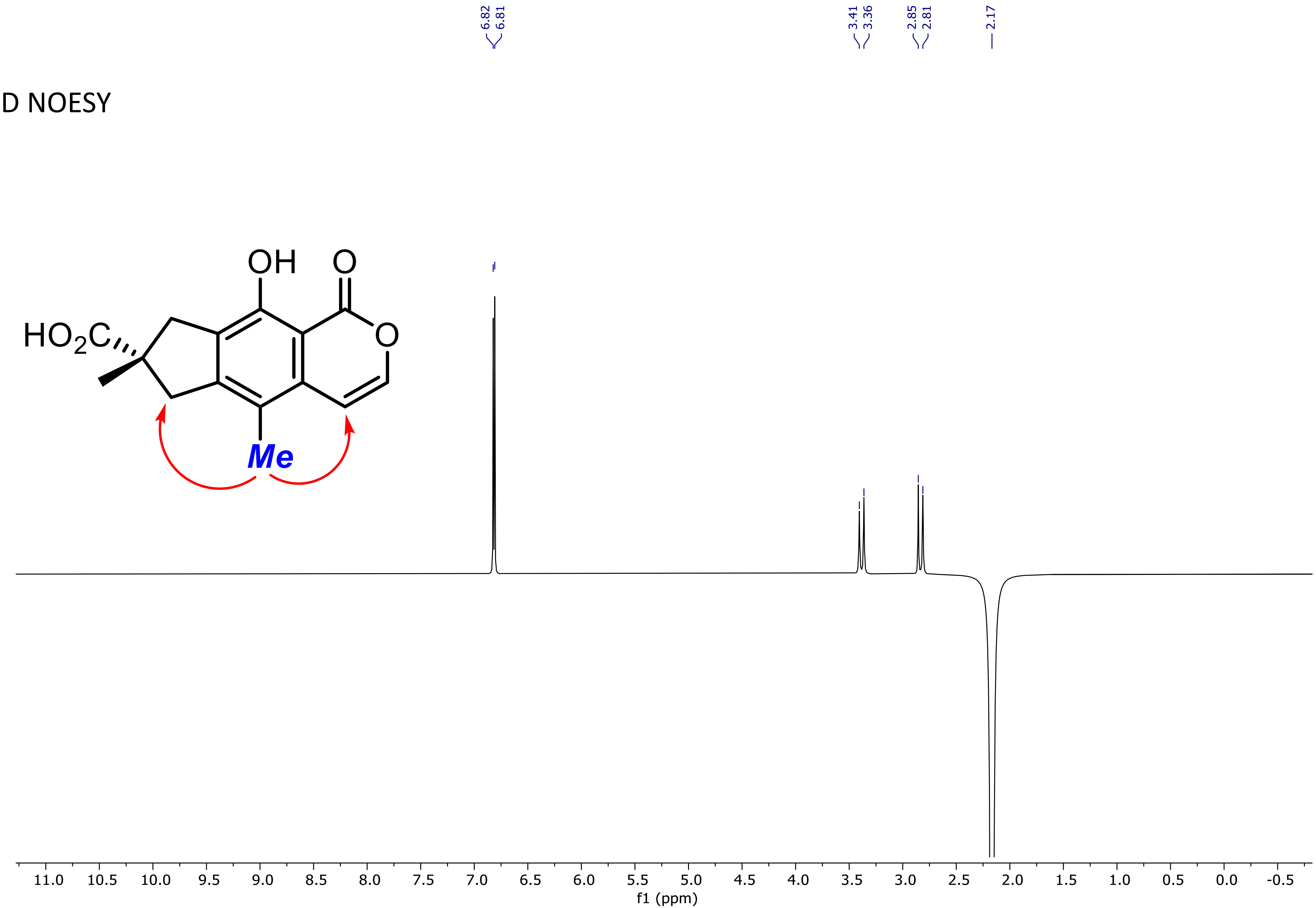

6.82
6.81
3.41
3.36
2.85
2.81
2.17
OH
O
HO2C
O
Me
11.0
10.5
10.0
9.5
9.0
8.5
8.0
7.5
7.0
6.5
6.0
5.5
5.0
4.5
4.0
3.5
3.0
2.5
2.0
1.5
1.0
0.5
0.0
-0.5
f1 (ppm)
